# New Zosteropenillines and Pallidopenillines from the Seagrass-Derived Fungus *Penicillium yezoense* KMM 4679

**DOI:** 10.3390/md22070317

**Published:** 2024-07-17

**Authors:** Elena V. Leshchenko, Ekaterina A. Chingizova, Alexandr S. Antonov, Nadezhda P. Shlyk, Gleb V. Borkunov, Dmitrii V. Berdyshev, Viktoria E. Chausova, Natalya N. Kirichuk, Yuliya V. Khudyakova, Artur R. Chingizov, Anatoly I. Kalinovsky, Roman S. Popov, Natalya Yu. Kim, Ksenia A. Chadova, Ekaterina A. Yurchenko, Marina P. Isaeva, Anton N. Yurchenko

**Affiliations:** 1 G.B. Elyakov Paсific Institute of Bioorganic Chemistry, Far Eastern Branch of the Russian Academy of Sciences, 159 Prospect 100-letiya Vladivostoka, Vladivostok 690022, Russia; 2 Institute of High Technologies and Advanced Materials, Far Eastern Federal University, Vladivostok 690922, Russia; 3 A.V. Zhirmunsky National Scientific Center of Marine Biology, Far Eastern Branch of Russian Academy of Sciences, Vladivostok 690041, Russia

**Keywords:** *Penicillium yezoense*, *Zostera marina*, decalin polyketides, ECD spectral calculations, cytoprotective activity, cytotoxic activity

## Abstract

Ten new decalin polyketides, zosteropenilline M (**1**), 11-*epi*-8-hydroxyzosteropenilline M (**2**), zosteropenilline N (**3**), 8-hydroxyzosteropenilline G (**4**), zosteropenilline O (**5**), zosteropenilline P (**6**), zosteropenilline Q (**7**), 13-dehydroxypallidopenilline A (**8**), zosteropenilline R (**9**) and zosteropenilline S (**10**), together with known zosteropenillines G (**11**) and J (**12**), pallidopenilline A (**13**) and 1-acetylpallidopenilline A (**14**), were isolated from the ethyl acetate extract of the fungus *Penicillium yezoense* KMM 4679 associated with the seagrass *Zostera marina*. The structures of isolated compounds were established based on spectroscopic methods. The absolute configurations of zosteropenilline Q (**7**) and zosteropenilline S (**10**) were determined using a combination of the modified Mosher’s method and ROESY data. The absolute configurations of zosteropenilline M (**1)** and zosteropenilline N (**3**) were determined using time-dependent density functional theory (TD-DFT) calculations of the ECD spectra. A biogenetic pathway for compounds **1**–**14** is proposed. The antimicrobial, cytotoxic and cytoprotective activities of the isolated compounds were also studied. The significant cytoprotective effects of the new zosteropenilline M and zosteropenillines O and R were found in a cobalt chloride (II) mimic in in vitro hypoxia in HEK-293 cells. 1-Acetylpallidopenilline A (**14**) exhibited high inhibition of human breast cancer MCF-7 cell colony formation with IC_50_ of 0.66 µM and its anticancer effect was reduced when MCF-7 cells were pretreated with 4-hydroxitamoxifen. Thus, we propose 1-acetylpallidopenilline A as a new xenoestrogen with significant activity against breast cancer.

## 1. Introduction

Marine-derived fungi still hold a leading position in terms of the number of newly isolated compounds each year [1]. *Penicillium yezoense* belongs to the *Aspergilloides* section and the *Thomiorum* series, which includes the 12 species, *P. fusisporum*, *P. aurantioviolaceum*, *P. valentinum*, *P. roseoviride*, *P. cartierense*, *P. thomii*, *P. yezoense*, *P. contaminatum*, *P. austroafricanum*, *P. crocicola*, *P. grevilleicola* and *P. jejuense*. Earlier, *P. yezoense* was treated as a synonym of *P. thomii,* and then they were accepted as distinct species [2]. Six species in this series were described relatively recently, in 2014–2015, including *P. jejuense*, which was found in marine habitats [2,3]. Representatives of the *Thomiorum* series in terrestrial environments are most often associated with substrates of plant origin and are also found in soils [4]. For example, *P. thomii* is known as a destructor of sphagnum mosses in peat bogs, where it is one of the mass species of micromycetes [5]. In the sea, representatives of this series are also associated with plants and are present in the soil where they grow [6,7]. *P. thomii* is known as a phosphorus-solubilizing microorganism [8] because it was reported that *P. thomii* isolate can solubilize the inorganic phosphorus and the mineralizate of organic phosphorus in volcanic soils [9]. This property of the fungus makes it very significant for symbiosis with plants.

A literature search revealed a lack of data on the isolation of secondary metabolites from these fungi, except for *P. thomii—*the most well-known species of this series. Various secondary metabolites have been isolated from marine *P. thomii* fungi, such as a novel xanthone dimer derivative [10], naphthoquinone derivatives [11], various chromones [12,13] and terpenoids [14,15]. A number of promising *P. thomii* strains from the Collection of Marine Microorganisms (KMM 4645, KMM 4667, KMM 4674 and KMM 4675), producing bioactive meroterpenoids [16], decalin-type [17,18] and spiroketal [19] polyketides, eudesmane-type [20,21] and guaiane-type [21] sesquiterpenes, and a prenylated indole alkaloid [22], were discovered by our scientific group. All of these strains were isolated from marine plants, such as the seagrass *Zostera marina*, brown algae *Sargassum miyabei* and *S. pallidum* collected in the Sea of Japan. 

Among the secondary metabolites of *P. thomii*, the decalin polyketides pallidopenillines and zosteropenillines have attracted particular attention. At this time, more than 200 decalin polyketides have been reported [23,24]. The most well-known decalin polyketides are lovastatin and other statins, which target the 3-hydroxy-3-methylglutaryl-CoA (HMG-CoA) reductase enzyme involved in cholesterol biosynthesis [25]. For other decalin polyketides, various bioactivities, including antimicrobial and cytotoxic activities, were found, which make their producers promising candidates for comprehensive study.

**Figure 1:**
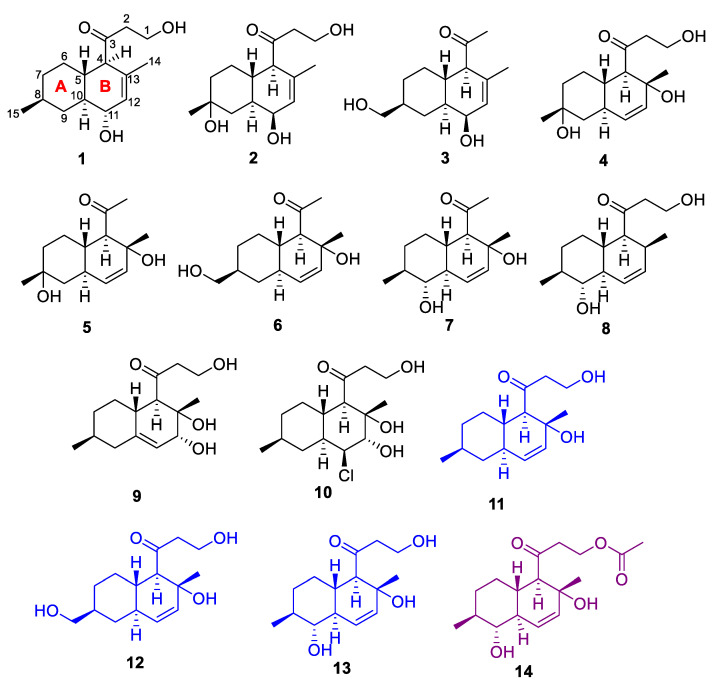
Metabolites isolated from the fungus *Penicillium yezoense* KMM 4679 (black—new compounds; blue—known compounds; purple—known bioactive compound).

To continue the search for new natural compounds from marine-derived fungi belonging to the *Thomiorum* series, we investigated a new strain, *Penicillium yezoense* KMM 4679, isolated from the seagrass *Z. marina*. Herein, we report the isolation and structural elucidation of 14 decalin polyketides, including new compounds **1**–**10** and known related compounds **11**–**14** (Figure 1) produced by this fungus and their biological activities.

## 2. Results

### 2.1. Molecular Identification of the Fungal Strain/Identification of Penicillium yezoense KMM 4679

**Figure 2:**
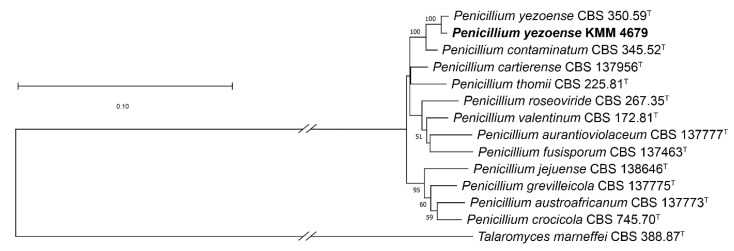
ML tree based on concatenated *ITS-BenA-CaM-RPB2* sequences showing the phylogenetic position of the strain KMM 4679 among members of the genus *Penicillium* section *Aspergilloides* series *Thomiorum*. Bootstrap values (%) of 1000 replications with confidence values greater than 50% are indicated in the nodes. The scale bars represent 0.10 nucleotide substitutions per site.

The strain KMM 4679 was identified using molecular markers such as the *ITS* region and partial *BenA*, *CaM* and *RPB2* genes. Approximately 600 bp fragments of the *ITS* region, about 500 bp fragments of the partial BenA gene, about 600 bp fragments of the partial *CaM* gene and about 1200 bp fragments of the partial *RPB2* gene were amplified and sequenced. According to the results of BLAST analysis, the sequence of the *ITS* region was 100% identical to the sequence of the ex-type strain *Penicillium yezoense* CBS 350.59T, while the *BenA* and *RPB2* gene sequences were more than 99% identical, and the *CaM* gene sequence was more than 98% identical. The phylogenetic Maximum Likelihood (ML) tree of the concatenated *ITS-BenA-CaM-RPB2* sequences clearly showed that the strain KMM 4679 clustered with the ex-type strain *Penicillium yezoense* CBS 350.59^T^ (Figure 2).

### 2.2. Structural Elucidation

**Figure 3:**
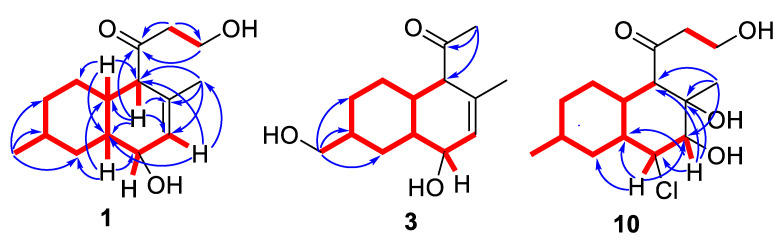
Key HMBC (blue arrows) and ^1^H-^1^H COSY (bold lines) correlations of **1, 3** and **10**.

The molecular formula of **1** was established to be C_15_H_24_O_3_ by the HRESIMS peak at *m*/*z* 275.1611 [M + Na]^+^ (Figure S1) and ^13^C NMR analyses. A close inspection of ^1^H and ^13^C NMR, DEPT and HSQC data for **1** (Table 1 and Table 2) revealed the presence of two methyl (δ_H_ 0.92, δ_C_ 22.5 and δ_H_ 1.60, δ_C_ 21.1) groups, five methylenes (δ_C_ 31.4, δ_C_ 34.3, δ_C_ 38.5, δ_C_ 43.1) including one oxygen-bearing (δ_H_ 3.85, δ_C_ 57.8), and five *sp*^3^-methines (δ_H_ 1.40, δ_C_ 31.7; δ_H_ 1.52, δ_C_ 38.9; δ_H_ 1.16, δ_C_ 45.3; δ_H_ 2.83, δ_C_ 62.2) including one oxygen-bearing (δ_H_ 3.88, δ_C_ 72.6), one *sp*^2^-methine (δ_H_ 5.59, δ_C_ 129.8) and two quaternary *sp*^2^-carbon (δ_C_ 132.0, δ_C_ 214.6). These data and four degrees of unsaturation from the molecular formula suggested that **1** possessed two rings, one double bond and one carbonyl group. ^1^H-^1^H COSY data and HMBC correlations (Figure 3) H_2_-1 (δ_H_ 3.85)/C-3 (δ_C_ 214.6); H_2_-2 (δ_H_ 2.70, 2.64)/C-1 (δ_C_ 57.8) and C-3; H-4 (δ_H_ 2.83)/C-5 (δ_C_ 38.9), C-10 (δ_C_ 45.3), C-13 (δ_C_ 132.0) and C-12 (δ_C_ 129.8); H-5 (δ_H_ 1.52)/C-4 (δ_C_ 62.3), C-6 (δ_C_ 31.3), C-10 and C-13; H_2_-6 (δ_H_ 1.65, 1.14)/C-5, C-7 (δ_C_ 34.3), C-8 (δ_C_ 31.7) and C-10; H-7a (δ_H_ 1.69)/C-5, C-6 and C-15 (δ_C_ 22.5); H-7b (δ_H_ 0.89)/C-9 (δ_C_ 38.5); H-8 (δ_H_ 1.40)/C-6, C-7, C-9, C-10 and C-15; H_3_-15 (δ_H_ 0.92)/C-7, C-8 and C-9; H_2_-9 (δ_H_ 2.21, 0.70)/C-5, C-7, C-8, C-10, C-11 (δ_C_ 72.6) and C-15; H-10 (δ_H_ 1.16)/C-5, C-9 and C-11; H-12 (δ_H_ 5.59)/C-10, C-4 and C-14 (δ_C_ 21.1); and H_3_-14 (δ_H_ 1.60)/C-4, C-13 and C-12 (δ_C_ 129.8) revealed the presence of a decalin moiety and established a Δ^12^ double bond**,** the location of methyl groups at C-8 and C-13, the location of a hydroxy group at C-11, and the location of a 3-hydroxy-1-oxopropyl side chain at C-4 in **1**. Thus, the chemical structure of **1** was determined as a decalin-type polyketide belonging to zosteropenilline and pallidopenilline series previously reported from the *Penicillium thomii* KMM 4674 [17,18]. 

**Figure 4:**
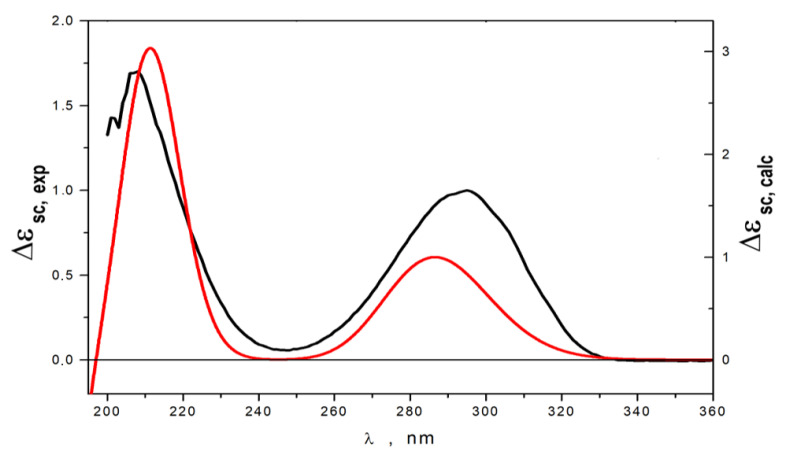
The experimental (black) and calculated (red) ECD spectra of **1**.

The relative configurations of **1** were assigned based on a ROESY experiment and ^1^H-^1^H coupling constants (Table 2). Observed ROESY correlations between H-4 and H-10, between H-5 and H-11, H_a_-2, and the magnitudes of the vicinal coupling constants H-4 (*J* = 10.0 Hz), H-5 (*J* = 11.5, 4.0 Hz) and H-11 (*J* = 9.0 Hz), indicated a *trans*-fusion of the A and B rings, an *α*-orientation of the 11-OH group and a *β*-orientation of the side chain at C-4. The orientation of H_3_-15 was determined to be *β* based on key ROESY correlations between H-8 and H-10 and biogenetic relationships with previously isolated polyketides [17,18]. The absolute stereochemistry of **1** was established based on quantum chemical calculations of the ECD spectrum. The conformational analysis of **1** was performed at the B3LYP/6-31G(d)_PCM level of theory. The internal rotations of the substituents at C-4 and C-11, as well as of the alkyl substituent at C-3, were considered. We found that the rotameric forms of **1** containing intramolecular hydrogen bonds were the most stable conformations. All other conformations lost to them by more than 3 kcal/mol (Figure S114). Conformations with a dihedral angle θ1 = ∠O−C(3)−C(4) −H(4) ≈ 180° were the most stable (Figure S114), and ECD spectra corresponding to them contained a very intensive positive band at λ ≈ 210 nm. The minor conformations, in which a carbonyl group stayed with a dihedral angle θ1 ≈ 0°, generated ECD spectra, in which the bands at λ ≈ 210 nm and at λ ≈ 290 nm had similar intensities (Figure S115). The experimental ECD spectrum of **1** contained two positive bands in the region λ ≥ 200 nm. Figure 4 presents a comparison of this spectrum with the calculated statistically averaged spectrum for 4*R*,5*S*,8*S*,10*S*,11*S*-**1**. A comparison of the features of the calculated and experimental ECD spectra showed good qualitative mutual correspondence. Thus, the absolute configuration of **1** was established to be 4*R*,5*S*,8*S*,10*S*,11*S*. Compound **1** was named zosteropenilline M. It is worth noting that a C-4-methylated derivative of zosteropenilline M (1) viridicatumone A was reported from halotolerant plant-associated *Penicillium viridicatum* [26].

The molecular formula of **2** was established to be C_15_H_24_O_4_ based on the HRESIMS peak at *m*/*z* 291.1542 [M + Na]^+^ (Figure S9) with four degrees of unsaturation. The carbon and proton signals belonging to C-1–C-4 (Table 1 and Table 2) observed for **2** were similar to those observed for **1**. Analysis of the proton and carbon signals in the ^1^H and ^13^C NMR spectra of **2,** including DEPT and HSQC experiments (Table 1 and Table 2), revealed the presence of two methyl (δ_H_ 1.28, δ_C_ 32.0 and δ_H_ 1.63, δ_C_ 21.7) groups, five methylenes (δ_C_ 27.0, δ_C_ 38.0, δ_C_ 40.4, δ_C_ 43.7), including one oxygen-bearing (δ_H_ 3.86, 3.85, δ_C_ 58.0), and four *sp*^3^-methines (δ_H_ 1.68, δ_C_ 33.6; δ_H_ 1.76, δ_C_ 33.6, δ_H_ 2.86, δ_C_ 62.4), including one oxygen-bearing (δ_H_ 3.86, δ_C_ 66.8), one *sp*^2^-methine (δ_H_ 5.89, δ_C_ 127.5), one quaternary *sp*^3^-carbon (δ_C_ 70.0) and two quaternary *sp*^2^-carbon (δ_C_ 135.4, δ_C_ 214.5). ^1^H-^1^H COSY cross-peaks H-9b (δ_H_ 1.59)/H-10 (δ_H_ 1.68), H-10/H-5 (δ_H_ 1.76), H-5/H-6b (δ_H_ 1.51), H-6b/H_2_-7 (δ_H_ 1.61, 1.38) and HMBC correlations H_3_-15 (δ_H_ 1.28)/C-7 (δ_C_ 38.0), C-8 (δ_C_ 70.0) and C-9 (δ_C_ 40.4) (Figure S119) established the structure of the A ring of **2** including the location of hydroxy and methyl groups at C-8. Observed magnitude of the ^3^*J* coupling constant (^3^*J*_H5-H10_ = 12.0 Hz) indicated *trans*-fusion of the A and B rings, ^3^*J* coupling constant (^3^*J*_H4-H5_ = 9.9 Hz) indicated *β*-orientation of the side chain at C-4, and ^3^*J*_H10-H11_ (3.3 Hz) indicated *β*-equatorial orientation of 11-OH group in **2**. The *β*-orientation C-15 was confirmed by the ROESY correlation between H-5 and H_3_-15. Thus, compound **2** was named 11-*epi*-8-hydroxyzosteropenilline M.

**Figure 5:**
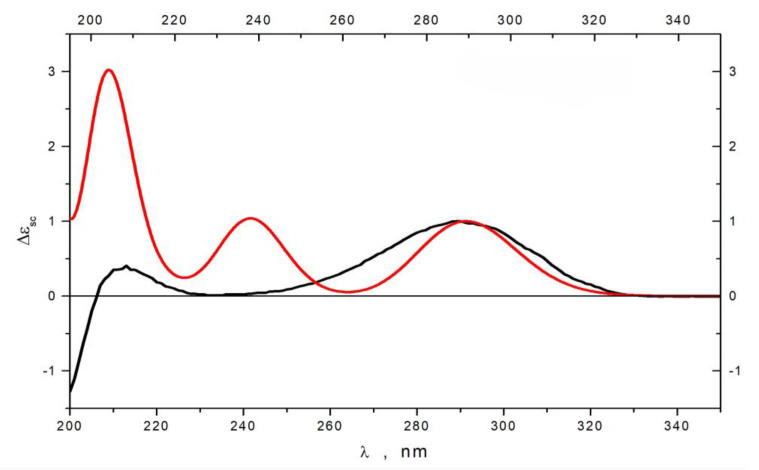
Experimental (black) and calculated (red) ECD spectra of **3**.

The molecular formula of **3** was established to be C_14_H_22_O_3_ by an HRESIMS peak at *m*/*z* 261.1473 [M + Na]^+^ (Figure S17) and corresponded with ^13^C NMR data. The chemical shift values of B ring atoms in the ^1^H and ^13^C NMR spectra of **3** (Table 1 and Table 2) were very similar to those obtained for compound **2.** A close inspection of ^1^H and ^13^C NMR data including DEPT and HSQC experiments of **3** revealed the presence of new methyl group (δ_H_ 2.14, δ_C_ 27.8) together with the absence of C-1 and C-2 methylene groups, and the presence of new one oxygen-bearing methylene group (δ_H_ 3.51, 3.50, δ_C_ 67.0). ^1^H-^1^H COSY data and HMBC correlations (Figure 3) H_2_-15 (δ_H_ 3.51, 3.50)/C-7 (δ_C_ 28.8), C-8 (δ_C_ 40.2), C-9 (δ_C_ 31.3) and H_3_-2 (δ_H_ 2.14)/C-3 (δ_C_ 211.2), and C-4 (δ_C_ 63.4) revealed the location of an oxygen-bearing methylene group at C-8 and acetyl group at C-4 in **3**. The relative configurations were determined based on ^1^H-^1^H coupling constant analysis. The observed magnitudes of the vicinal coupling constants of H-10 (δ_H_ 1.32, *J* = 11.5, 3.3 Hz) and H-11 (δ_H_ 3.92, *J* = 5.6, 3.1 Hz) indicate the *β*-equatorial orientation of the 11-OH group in **3** as in **2**. The absolute configurations of the stereocenters of **3** were established using calculated ECD data (Figure 5). The experimental ECD spectrum of **3** contained an intense negative band in the λ ≤ 207 nm region and two positive bands in the λ ≥ 207 nm region. The band at λ ≈ 290 nm was more intense than that at λ ≈ 212 nm. A comparison of ECD spectra calculated for different conformations of **3** showed that the sign and position of the band at λ ≈ 290 nm were the same for all selected conformations. On the contrary, the shape of the calculated ECD spectrum in the λ ≤ 264 nm region was very conformationally dependent (Figure S116). The shape of the final statistically averaged ECD spectrum depends hardly on the values of calculated statistical weights, which are themselves dependent on the chosen level of theoretical modeling. For **3**, this is a possible cause for why the theoretical ECD spectrum deviates from the experimental one in the λ ≤ 275 nm region (Figure 5). An ECD spectrum of 4*R*,5*S*,8*S*,10*S*,11*R*-**3** was modeled for situations, when total amounts of both types of conformations (dihedral angle 0°:180°) related as 40:60, 60:40 and 80:20 (Figure S117). It is seen that the growth of the number of conformations with a dihedral angle 0° leads to better qualitative correspondence of calculated and experimental ECD spectra. Thus, the absolute stereochemistry of **3** was determined to be 4*R*,5*S*,8*S*,10*S*,11*R*. Compound **3** was named zosteropenilline N. 

The molecular formula of **4** was established to be C_15_H_24_O_4_ by the HRESIMS peak at *m*/*z* 291.1579 [M + Na]^+^ (Figure S25), which was confirmed by ^13^C NMR analyses. The ^1^H and ^13^C NMR spectra (Table 1 and Table 2) for compound **4** were very similar to those obtained for zosteropenilline G (**11**), previously isolated from the *Penicillium thomii* KMM 4674 [18], except for the C-7–C-10 and C-15 carbon signals and peaurantiogriseol D previously isolated from the *Penicillium aurantiogriseum* [27], except the lacking methyl at C-4. ROESY correlation of H_3_-14 (δ_H_ 1.20)/H-5 (δ_H_ 1.50) confirmed the *β*-orientation of the methyl group at C-13. Moreover, the ROESY correlation H-4 (δ_H_ 2.95)/H-10 (δ_H_ 2.31), together with the observed magnitude of the vicinal coupling constant H-4 (*J* = 11.6 Hz), confirmed the *trans*-fusion of the A and B rings and the *β*-orientation of the 3-hydroxy-1-oxopropyl residue at C-4. Thus, compound **4** was named 8-hydroxyzosteropenilline G.

The molecular formula of **5** was established to be C_14_H_22_O_3_ by the HRESIMS peak at *m*/*z* 261,1471 [M + Na]^+^ (Figure S32) and was confirmed by ^13^C NMR analyses. The ^1^H and ^13^C NMR data (Table 1 and Table 2) observed for **5** closely resembled those obtained for **4,** except for signals of C-1 and C-2. A close inspection of the ^1^H and ^13^C NMR, DEPT and HSQC data of **5** revealed that the A and B rings were the same as in **4,** and the acetyl group at C-4 was the same as in **3**. ROESY correlation H_3_-14 (δ_H_ 1.19)/H-5 (δ_H_ 1.47) confirmed the *β*-orientation H_3_-14. Thus, the structure of compound **5** was established and named zosteropenilline O. This compound is very similar to libertalide C, which has an additional methyl group at C-4 and was isolated from the coral-derived fungus *Libertasomyces* sp. [28].

The molecular formula of **6** was established to be C_14_H_22_O_3_ based on the HRESIMS peak at *m*/*z* 261.1470 [M + Na]^+^ (Figure S40). A close inspection of the ^1^H and ^13^C NMR, DEPT and HSQC data of **6** (Table 3 and Table 4) revealed the presence of two methyl groups (δ_H_ 1.18, δ_C_ 25.7 and δ_H_ 2.28, δ_C_ 34.8), four methylenes (δ_C_ 28.8, δ_C_ 29.0, δ_C_ 35.3) including one oxygen-bearing (δ_H_ 3.48, 3.45; δ_C_ 68.2), four *sp*^3^ methines (δ_H_ 1.61, δ_C_ 40.6; δ_H_ 1.49, δ_C_ 41.4; δ_H_ 1.86, δ_C_ 41.5; δ_H_ 2.86, δ_C_ 63.7), two *sp*^2^ methines (δ_H_ 5.45, δ_C_ 131.6, δ_H_ 5.45, δ_C_ 134.2), one quaternary *sp*^3^-carbon (δ_C_ 72.6) and an *sp*^2^-carbon (δ_C_ 211.1). These data and four degrees of unsaturation from the molecular formula suggested that compound **6** possessed two rings, one double bond and one keto group. ^1^H-^1^H COSY data and HMBC correlations (Figure S119) revealed the presence of a decalin moiety and established a Δ^11^ double bond**,** the location of a methyl group at C-13, the location of an oxygen-bearing methylene group at C-8 and the location of an acetyl group at C-4 in **6**. ROESY correlation H-5 (δ_H_ 1.49)/H_3_-14 (δ_H_ 1.18) and H_2_-15 (δ_H_ 3.48, 3.45) confirmed the *β*-orientation methyl and methylene groups at C-13 and C-8, accordingly. Thus, compound **6** was named zosteropenilline P.

**Figure 6:**
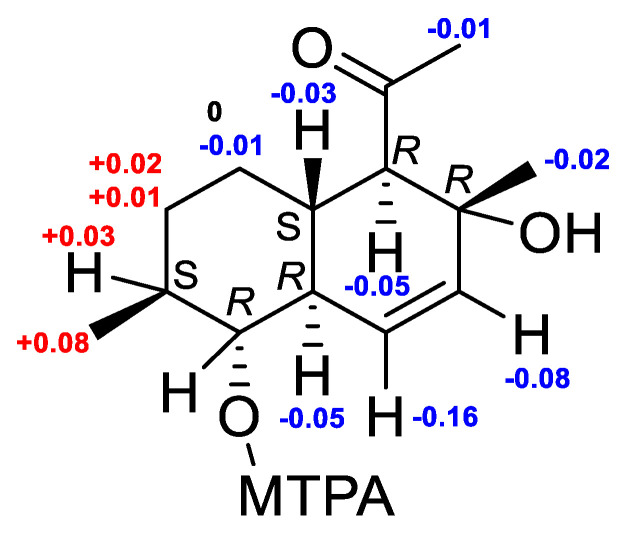
The chemical shift differences Δδ (δ_S_ − δ_R_) (in ppm) for the (*S*)- and (*R*)-MPTA esters of **7**.

The molecular formula of **7** was established to be C_14_H_22_O_3_ based on the HRESIMS peak at *m*/*z* 261.1469 [M + Na]^+^ (Figure S48). The ^1^H and ^13^C NMR spectra for **7** were very similar to **6** except for the С-7 (δ_C_ 32.8), C-9 (δ_C_ 78.1), C-10 (δ_C_ 48.5) and C-15 (δ_C_ 18.4) carbon signals and H-9 (δ_H_ 2.83) and H_3_-15 (δ_H_ 1.03) proton signals. ^1^H-^1^H COSY data and HMBC correlations H_3_-15 (δ_H_ 1.03)/C-7 (δ_C_ 32.8), C-8 (δ_C_ 40.6) and C-9 (δ_C_ 78.8), and H-9/C-10, C-8 and C-15 (Figure S119) established the structure of the A ring of **7** including the location of hydroxyl and methyl groups at C-9 and C-8, respectively. The ROESY correlation H-5 (δ_H_ 1.59)/H_3_-14/H-9 together with observed magnitudes of the vicinal coupling constant of H-9 (*J* = 9.8 Hz) established the α-configuration of the 9-OH group and *β*-orientation H_3_-14. The absolute configuration of C-9 was determined using the modified Mosher’s method [29]. Esterification of **7** with (*R*)- and (*S*)-MTPA chlorides occurred at the C-9 hydroxy group to give (*S*)- and (*R*)-MTPA esters **7b** and **7a**, respectively. The observed chemical shift differences ∆δ (δ*_S_* − δ*_R_*) (Figure 6) indicated a 9*R* configuration. These data and the observed magnitudes of the vicinal coupling constants H-4 (δ_H_ 2.88, *J* = 11.7 Hz), H-9 (δ_H_ 2.83, *J* = 9.8 Hz) and H-10 (δ_H_ 1.80, *J* = 10.0, 2.2 Hz) determined the absolute stereostructure of **7** as 4*R*,5*S*,8*S*,9*R*,10*R*,13*R*, which was the same as that of pallidopenilline A [17]. Thus, compound **7** was named zosteropenilline Q. 

The molecular formula of **8** was established to be C_15_H_24_O_3_ based on the HRESIMS peak at *m*/*z* 275.1592 [M + Na]^+^ (Figure S56). Analysis of ^1^H and ^13^C NMR, DEPT and HSQC data of **8** (Table 3 and Table 4) revealed the presence of a decalin moiety, as well as an established Δ^11^ double bond**,** the location of methyl groups at C-8 and C-13, the location of a hydroxy group at C-9, and the location of a 3-hydroxy-1-oxopropyl residue at C-4 in **8**. The planar structure of compound **8** was similar to pallidopenilline А [17], except for the absence of 13-ОН, and close to craterellone B [30], except for the 3-hydroxy-1-oxopropyl residue at C-4 instead of an acetyl group and the absence of the methyl group at C-4. The observed magnitudes of the vicinal coupling constants of H-4 (*J* = 11.2, 6.0 Hz) and H-13 (*J* = 6.0, 4.0 Hz) established the *β*-configuration of the methyl group at C-13. Thus, compound **8** was named 13-dehydroxypallidopenilline A.

**Figure 7:**
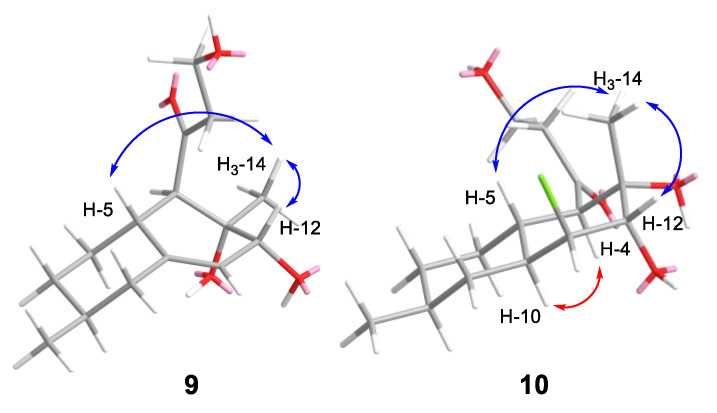
Key ROESY correlations of **9** and **10**.

The molecular formula of **9** was established to be C_15_H_24_O_4_ based on the HRESIMS peak at *m*/*z* 291.1579 [M + Na]^+^ (Figure S64). The ^1^H and ^13^C NMR spectra (Table 3 and Table 4) for compound **9** were very similar to those obtained for zosteropenilline L [18] with the exception of the C-7–C-9, C-12, С-14 and C-15 carbon and proton signals and libertalide M, except for the lack of a methyl at C-4 [28]. ^1^H-^1^H COSY data and HMBC correlations H-11 (δ_H_ 5.50)/C-5 (δ_C_ 39.4), C-10 (δ_C_ 145.3), C-12 (δ_C_ 72.3) and C-13 (δ_C_ 73.4); H-12 (δ_H_ 3.61)/C-4 (δ_C_ 57.9), C-10 and C-11 (δ_C_ 118.2); H-5 (δ_H_ 2.38)/C-4, C-10, C-11 and C-6 (δ_C_ 32.8); and H_3_-15 (δ_H_ 0.90)/C-7 (δ_C_ 33.9), C-8 (δ_C_ 33.6) and C-9 (δ_C_ 43.0) established a decalin moiety and a Δ^10^ double bond**,** the location of methyl groups at C-8 and C-13, and the location of hydroxyl groups at C-12 and C-13. The ROESY correlations H_3_-14 (δ_H_ 1.06)/H-5 and H-12, H-5/H-12 (Figure 7) and the observed magnitude ^3^*J* coupling constant (^3^*J*_H4-H5_ = 10.2 Hz) established the β-orientation of H_3_-14 and α-orientation of 12-OH. 

**Figure 8:**
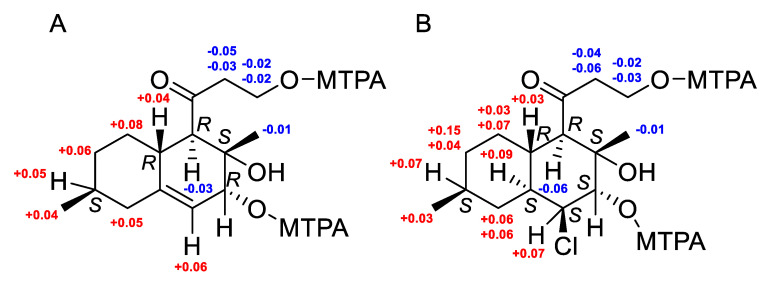
The chemical shift differences Δδ (δ_S_ − δ_R_) (in ppm) for the (*S*)- and (*R*)-MPTA esters of **9** (**A**) and **10** (**B**).

The absolute configuration of C-12 was determined using the modified Mosher’s method [29]. Esterification of **9** with (*R*)- and (*S*)-MTPA chlorides occurred at the C-12 hydroxy group to give the (*S*)- and (*R*)-MTPA esters **9b** and **9a**, respectively. The observed chemical shift differences ∆δ (δ*_S_* − δ*_R_*) (Figure 8A) indicated the 12*R* configuration. Thus, the full absolute stereostructure of **9** was determined as 4*R*,5*R*,8*S*,12*R*,13*S*. Compound **9** was named zosteropenilline R. It should be noted that earlier, the same configuration of C-12 was reported for zosteropenilline L [18], while chemical shift values of C-12 and H-12 were very different in comparison with those for **9**. It is possible that the NOESY data were incorrectly interpreted when determining the stereochemistry of zosteropenilline L.

The molecular formula of **10** was established to be C_15_H_25_ClO_4_ based on the HRESIMS peak at *m*/*z* 327.1346 [M + Na]^+^, the 3:1 isotopic distribution in the mass spectrum was indicative of the presence of one chlorine atom (Figure S72). A close inspection of the ^1^H and ^13^C NMR, DEPT and HSQC data of **10** (Table 3 and Table 4) revealed the presence of two methyl groups (δ_H_ 0.91, δ_C_ 22.4 and δ_H_ 1.42, δ_C_ 24.3), five methylenes (δ_C_ 30.8, δ_C_ 33.9, δ_C_ 38.1, δ_C_ 49.1), including one oxygen-bearing (δ_H_ 3.90, 3.86; δ_C_ 58.3), six *sp*^3^ methines (δ_H_ 1.77, δ_C_ 34.7; δ_H_ 1.43, δ_C_ 32.4; δ_H_ 1.91, δ_C_ 39.4; δ_H_ 2.87, δ_C_ 60.4), including one oxygen-bearing (δ_H_ 3.76, δ_C_ 78.5) and one chlorine-bearing (δ_H_ 4.15, δ_C_ 63.3), one quaternary *sp*^3^ carbon signal (δ_C_ 73.4), and one quaternary *sp*^2^-carbon (δ_C_ 214.5). ^1^H-^1^H COSY data and HMBC correlations (Figure 3) H_3_-14 (δ_H_ 1.42)/C-4 (δ_C_ 60.4), C-13 (δ_C_ 73.4) and C-12 (δ_C_ 78.5); H-12 (δ_H_ 3.76)/C-4, C-13, C-11 (δ_C_ 63.3) and C-10 (δ_C_ 39.4); H-11 (δ_H_ 4.15)/C-5 (δ_C_ 34.7), C-9 (δ_C_ 38.1), C-10, C-12 and C-13; H-10 (δ_H_ 1.91)/C-4, C-5, C-6 and C-8; H-5 (δ_H_ 1.77)/C-4, C-6, C-10 and C-7 (δ_C_ 33.9); H-4 (δ_H_ 2.87)/С-2 (δ_C_ 49.1), C-3 (δ_C_ 213.1), C-5 (δ_C_ 34.7), C-10 (δ_C_ 39.4), C-13 and C-14 (δ_C_ 24.3); and H_3_-15 (δ_H_ 0.91)/C-7, C-8 (δ_C_ 32.4) and C-9 revealed the presence of a decalin moiety and established the location of methyl groups at C-8 and C-13, the location of hydroxy groups at C-12 and C-13, and the location of a 3-hydroxy-1-oxopropyl residue at C-4 in **10**. These data, as well as the chemical shifts of C-11 methine (δ_C_ 63.3, δ_H_ 4.15), indicated the location of the chlorine atom at C-11. The planar structure of **10** was similar to libertalide I except for the lack of a methyl at C-4 [28].The relative configuration of **10** was assigned based on ^1^H-^1^H coupling constants H-4 (δ_H_ 2.87, *J* = 11.3 Hz), H-5 (δ_H_ 1.77, *J* = 11.3, 3.5 Hz), H-10 (δ_H_ 1.91, *J* = 11.3, 3.4 Hz), H-11 (δ_H_ 4.15, *J* = 3.1 Hz) and H-12 (δ_H_ 3.76, *J* = 2.8 Hz), together with ROESY correlations H-4/H-10, H_3_-14/H-5 and H-12 (Figure 8). The absolute configuration of C-12 was determined using the modified Mosher’s method [29]. Esterification of **10** with (*R*)- and (*S*)-MTPA chlorides occurred at the C-12 hydroxy group to give (*S*)- and (*R*)-MTPA esters **10b** and **10a**, respectively. The observed chemical shift differences ∆δ (δ*_S_* − δ*_R_*) (Figure 8B) indicated the 12-*S* configuration. These data determined the absolute stereostructure of **10** to be 4*R*,5*R*,8*S*,10*S*,11*S*,12*S*,13*S*. Thus, compound **10** was named zosteropenilline S. 

In addition to the nine new polyketides **1**–**7**, **9** and **10** related to zosteropenillines and one new pallidopenilline A derivative (**8**), known zosteropenillines G (**11**) and J (**12**) [18], pallidopenilline A (**13**) and 1-acetylpallidopenilline A (**14**) [17] were isolated from the fungus *Penicillium yesoense* KMM 4679. 

### 2.3. Biological Activity of Isolated Compounds

#### 2.3.1. Antimicrobial Activity

The effects of compounds **1**, **2** and **4**–**14** on *Staphylococcus aureus* ATCC 21027, *Escherichia coli* VKPM (B-7935) and *Candida albicans* KMM 455 test strain growth and biofilm formation were investigated, and the data are presented in Table 5. Compound **3** was isolated in insufficient amounts and was not investigated in all experiments.

Thus, the antimicrobial activities of the isolated compounds were weak. None of the tested compounds exhibited antimicrobial activity against *E. coli*. Compounds **1** and **6**–**14** at 100 µM inhibited the growth of *S. aureus* by nearly 15–30%. Compounds **1**, **2**, **4–7**, **9**, **11** and **12** inhibited the growth of *C. albicans* by 6–35% at a concentration of 100 µM. Nevertheless, none of the studied compounds affected the formation of biofilms of the test strains of microorganisms at concentrations up to 100 μM. 

#### 2.3.2. Cytotoxic Activity of Isolated Compounds

The influence of compounds **1**, **2** and **4**–**14** on the viability of normal human kidney HEK 298 cells, as well as human prostate PC-3, cervical HeLa and breast MCF-7 cancer cells, was measured using the MTT assay after 24 h of treatment. The results are presented in Table 6.

None of the investigated compounds showed a significant cytotoxic effect on normal kidney cells after 24 h. Compounds **5**−**7**, only at a concentration of 100 µM, decreased the viability of HEK-298 cells by approximately 15–20%. The cytotoxic effect of the compounds on PC-3 cell viability was weak, excluding that of compound **14**. The concentration of half-maximal inhibition (IC_50_) for **14** was calculated as 94.20 ± 1.13 µM. The effect of these compounds on HeLa cells was more pronounced. The IC_50s_ values calculated for **2**, **5** and **11** were 87.29, 82.49 and 79.68 µM, respectively. Moreover, compound **14** half-maximally inhibited the viability of MCF-7 cells at 71.98 ± 2.48 µM.

#### 2.3.3. Cytoprotective Activity

**Figure 9:**
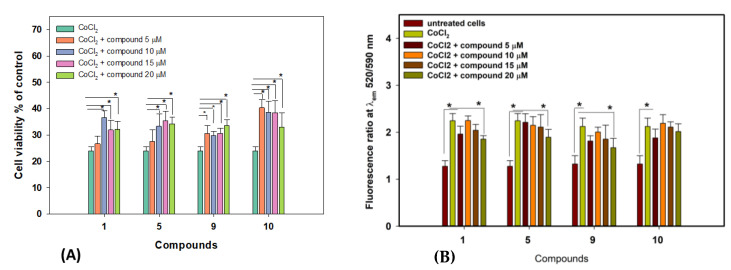
Effects of compounds **1**, **5**, **9** and **10** on CoCl_2_-treated HEK-293 cell viability (**A**) and lipid peroxidation level (**B**). Data are presented as the mean ± SEM. * *p*-value ≤ 0.05 considered significant.

The cytoprotective effects of the isolated compounds were investigated in cobalt (II) chloride-induced HEK-293 cells, and some compounds showed significant effects in this in vitro model. The influence of compounds **1**, **5**, **9** and **10** on the viability and lipid peroxidation levels in CoCl_2_-treated cells is presented in Figure 9.

The treatment with CoCl_2_ at 500 µM for 24 h decreased the viability of HEK-293 cells by 75% (Figure 9A). Zosteropenilline M (**1**), at concentrations of 10–20 µM, increased the viability of CoCl_2_-treated HEK-293 cells by 33.6–53.3%. Zosteropenilline O (**5**), at concentrations of 10–20 µM, increased the viability of these cells by 39.9–48.3%. Zosteropenilline R (**9**), at concentrations of 5–20 µM, increased the viability of these cells by 28.2–40.8%. Zosteropenilline S (**10**), at concentrations of 5–20 µM, increased the viability of these cells by 38.2–69.3%.

The toxic effect of CoCl_2_ resulted in a significant increase in lipid peroxide levels in HEK-293 cells, which was measured using the MitoCLox fluorescence dye. Compounds **1**, **5** and **9** significantly decreased lipid peroxide levels in CoCl_2_-treated HEK-293 cells (Figure 9B). 

#### 2.3.4. Anticancer Activity of 1-Acetylpallidopenilline A (**14**)

In the experiments (Section 2.3.2), only 1-acetylpallidopenilline A (**14**) showed some cytotoxic activity against cancer PC-3, HeLa and MCF-7 cells compared to normal HEK-293 cells. Therefore, various aspects of the anticancer activity of compound **14** were investigated.

**Figure 10:**
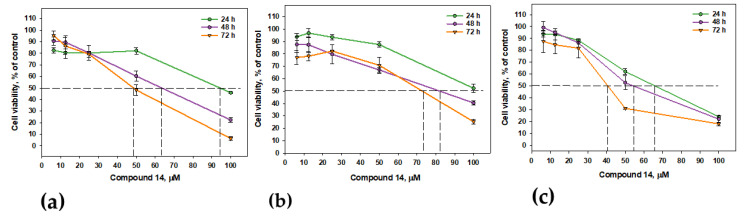
Cytotoxic activity of **14** against PC-3 (**a**), HeLa (**b**) and MCF-7 (**c**) cells after 24, 48 and 72 h. Data are presented as the mean ± SEM.

The exposition of PC-3, HeLa and MCF-7 cells with **14** for 48 h and 72 h resulted in an increase in its cytotoxicity (Figure 10). The IC_50s_ values (for PC-3 cells) were calculated as 63.35 µM and 48.48 µM, respectively. The IC_50s_ values (for HeLa cells) were calculated as 82.71 µM and 73.15 µM, respectively. Finally, The IC_50s_ values (for MCF-7 cells) were calculated as 54.72 µM and 41.03 µM, respectively.

**Figure 11:**
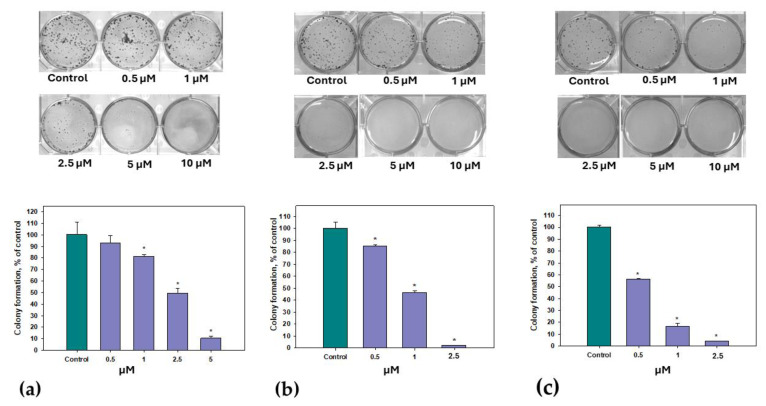
Effect of **14** on the formation of PC-3 (**a**), HeLa (**b**) and MCF-7 (**c**) colonies. Data are presented as the mean ± SEM. * indicates the significant differences from the control with *p*-value ≤ 0.05.

The effects of **14** on PC-3, HeLa and MCF-7 cell colony formation were investigated, and the data are presented in Figure 11. 

The effect of **14** on colony formation was very significant. The half-maximal concentrations of the inhibition of PC-3, HeLa and MCF-7 colony formation was calculated for **14** as 2.48 ± 0.11 µM, 0.96 ± 0.02 µM and 0.66 ± 0.01 µM, respectively.

**Figure 12:**
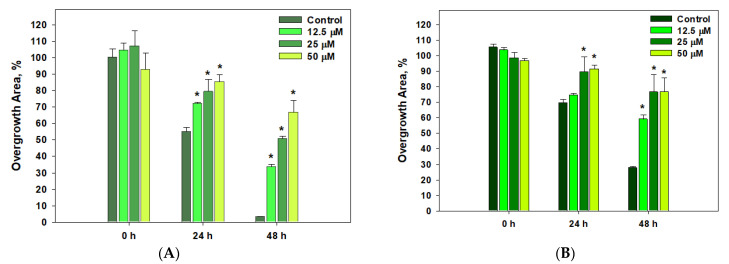
The influence of 1-acetylpallidopenilline A (**14**) on the migration of HeLa (**A**) and MCF-7 (**B**) cells. Data are presented as the mean ± SEM. * indicates the significant differences from the control with *p*-value ≤ 0.05.

The data on the effect of **14** on PC-3, HeLa and MCF-7 cell migration in the scratch assay are presented in Figure 12.

Thus, **14** significantly inhibited the migration of HeLa and MCF-7 cells (Figure 12), but did not affect the migration of PC-3 cells.

**Figure 13:**
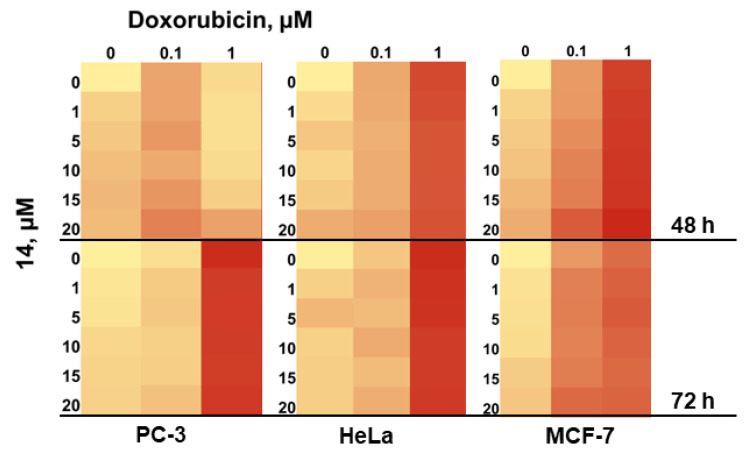
Combined effect of **14** and doxorubicin on the viability of PC-3, HeLa and MCF-7 cells. Data are presented as the mean ± SEM.

Finally, we investigated the combined cytotoxic effects of doxorubicin and **14** added together on the viability of PC-3, HeLa and MCF-7 cells. The data are presented in Figure 13. 

Therefore, doxorubicin at a concentration of 1 µM decreased MCF-7 cell viability by approximately 60%, and the combination of compound **14** with doxorubicin was more toxic. A more significant effect was observed when doxorubicin was administered at 0.1 µM. It decreased MCF-7 viability by only 30%, but the combination of **14** with doxorubicin diminished MCF-7 viability by 50%.

**Figure 14:**
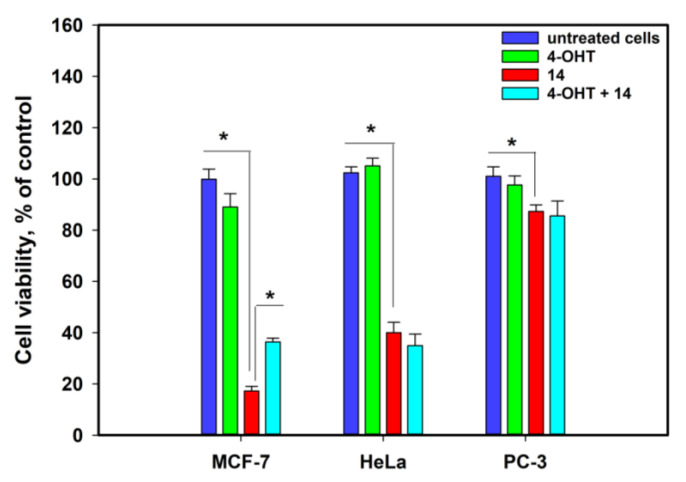
The influence of **14** (at a 75 µM) on cell viability in the presence of 4-OHT (at 10 µM). Data are presented as the mean ± SEM. * *p*-value ≤ 0.05 considered significant.

Thus, the more significant activity of **14** against MCF-7 cells, and to a lesser extent against HeLa cells, in contrast to PC-3, was observed in various experiments. To detect the possible target of **14**, we used 4-hydroxytamoxifen (4-OHT) at a concentration of 10 µM as a known inhibitor of estrogen receptors (ERs), and the data are presented in Figure 14. 

No significant differences were detected between **14** and **14** after pretreatment with 4-OHT in HeLa and PC-3 cells. However, when MCF-7 cells were pretreated with 4-OHT and then treated with **14**, a significant decrease in the cytotoxicity of **14** was observed. This confirmed that ERs are one of the possible targets for this fungal metabolite.

## 3. Discussion

**Figure 15:**
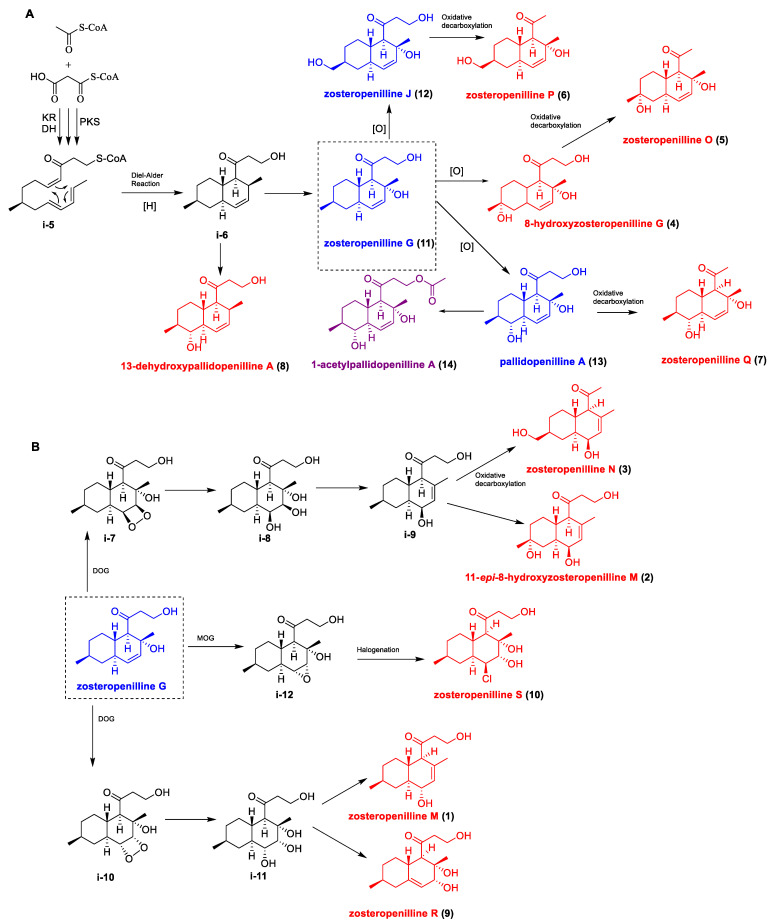
Proposed biosynthetic pathway for compounds 1–14.

Despite the relative occurrence of polyketides similar to isolated compounds **1**–**14** [28,30], the absence of a methyl group at C-3 is a characteristic feature of only two related series of heptaketides: zosteropenillines and pallidopenillines, produced by several strains of *Penicillium* spp. [17,18]. The biosynthesis of such heptaketides has not been reported before, but it is obvious that it occurs similarly to the biosynthesis of the nonaketide derivative lovastatin, which has a very similar decalin moiety [31]. Thus, the decalin framework is formed as a result of the intramolecular Diels–Alder cyclization of the linear heptaketide precursor. It is likely that the C-9 hydroxy derivative **8** and the C-13 hydroxy derivative zosteropenilline G (**11**) are obtained directly from the formed intermediate **i-5** (Figure 15A). According to our suggestion, zosteropenilline G (**11**) is a key intermediate for most other compounds. Compounds **4**, **12** and **13** are simple hydroxy derivatives of zosteropenilline G (**11**). Compounds **5**–**7** with shortened side chains are possible products of the oxidative decarboxylation of the corresponding compounds **4**, **12** and **13** with a “normal” side chain. The action of dioxygenase on the double bond of zosteropenilline G (**11**) with the subsequent hydrolysis of peroxides can lead to the formation of β-cis-diol **i-7** and α-cis-diol **i-10** (Figure 15B). The dehydration of the latter probably leads to the formation of zosteropenilline M (**1**) and with the formation of a double bond Δ^10^ in zosteropenilline R (**9**). Dehydration of **i-7** is realized with the formation of a double bond Δ^12^ in the intermediate **i-8**, followed by oxidation of C-15 and shortening of the side chain, which gives **3**, and oxidation of C-8 to form **2**. The epoxidation of the double bond leads to **i-12**. The action of epoxide hydrolase on **i-12** and the subsequent halogenation of tetraol **i-13** yields zosteropenilline S (**10**).

At present time, selective estrogen receptor downregulators (SERDs) that act as pure antagonists by interfering with the binding of estradiol to estrogen receptors (ERs) are used for hormone receptor-positive (HR+) breast cancer treatment [32]. Fulvestrant is a first-generation steroidal SERD approved by the FDA in 2007 for the treatment of ER-positive metastatic breast cancer [33]. Selective estrogen receptor modulators (SERMs) modulate ER activity by changing the coregulators to which they bind, and nonsteroidal SERMs can be classified on the basis of their structures, such as triphenylethylenes (tamoxifen, toremifene and idoxifene), phenylindoles (bazedoxifene and pipindoxifene), benzothiophenes (raloxifene and arzoxifene), benzopyrans (acolbifene), tetrahydronaphthalenes (lasofoxifene) and dihydrobenzoxathiins [34]. The sources of the reported SERDs and SERMs are plants, fungi and synthetic analogs of natural compounds, including xenoestrogens, which are structurally different from known estrogenic compounds. Examples of this include the p-hydroxyalkyl benzene derivative actinopolymorphol A from the actinomycete *Actinopolymorpha rutilus* [35] and the decalin derivatives fusarielins A, F, G and H from the fungus *Fusarium graminearum* [36]. In the present study, we propose 1-acetylpallidopenilline A as a new mycoestrogen with potent antimetastatic activity against MCF-7 breast cancer cells.

On the other hand, the new possibility of 1-acetylpallidopenilline A was found to increase the cytotoxicity of doxorubicin. Other earlier decalin polyketides zosteropenillines A, C and G were found to be upregulators of autophagy-related cargo protein p62 (which increased levels associated with the inhibition of autophagy flux) in PC-3 cells [18]. 

It is obvious that the presence of a 1-acetyl chain in the structure of pallidopenillines leads to an increase in the antitumor properties of the compounds of these series. It is quite difficult to draw other conclusions about the influence of the structure on cytotoxic activity, since the differences in activity are small.

Moreover, the investigation of the fungal *Penicillium yezoense* KMM 4679 strain resulted in the discovery of the decalin polyketides zosteropenillines O, К and S with protective effects against hypoxia-induced HEK-293 cell damage and induced mitochondrial lipid peroxide oxidation. Previously, it was shown that zosteropenillines B, H and J could downregulate NO levels in LPS-stimulated RAW264.7 murine macrophages, indicating their possible anti-inflammatory activity [18]. New data on the antioxidant activity of zosteropillines may be interesting for future investigations.

## 4. Materials and Methods

### 4.1. General Experimental Procedures

Optical rotations were measured on a Perkin-Elmer 343 polarimeter (Perkin Elmer, Waltham, MA, USA) in MeOH. UV spectra were recorded on a Shimadzu UV-1601PC spectrometer (Shimadzu Corporation, Kyoto, Japan) in MeOH. ECD spectra were measured using a Chirascan-Plus CD Spectrometer (Leatherhead, UK) in MeOH. ^1^H and ^13^C NMR spectra were recorded in aceton-d_6_ on Bruker Avance-500 and Avance III-700 spectrometers (Bruker BioSpin GmbH, Rheinstetten, Germany), operating at 500 MHz and 125 MHz, 700 MHz and 176 MHz, respectively, using TMS as an internal standard. HRESIMS spectra were obtained using a Bruker maXis Impact II mass spectrometer (Bruker Daltonics GmbH, Rheinstetten, Germany) (for compounds **3**–**7**, **9**–**14**, **10**a and **10**b). HPLC-MS/MS analysis was performed on a Shimadzu HPLC system (Kyoto, Japan), LC-30AD pumps and an LC-20AC autosampler connected to a Shimadzu IT-TOF (Kyoto, Japan) with an electrospray ionization (ESI) ion source (for compounds **1**, **2**, **8**, **7a** and **7b**). Pre-column ODS Ascentis Supelguard (2 cm × 2.1 mm), 5 µm (Sigma-Aldrich, St. Louis, MO, USA), was used (Figure S116). 

Plates precoated with Si gel (5–17 μm, 4.5 × 6.0 cm, Imid) and Si gel60 RP-18 F254S (20 × 20 cm; Merck KGaA, Darmstadt, Germany) were used for thin-layer chromatography. Preparative HPLC was carried out on a SepaBean machine with a UV detector combined with an ELSD detector using a Buchi glass column (49 × 230 mm) passed over a Si gel KSK (50/100 μm; Imid Ltd., Moscow, Russia), and on Shimadzu LC-20 (Shimadzu, Kyoto, Japan) and Agilent 1100 (Agilent Technologies, Santa Clara, CA, USA) chromatographs using Shimadzu RID-20A and Agilent 1100 RID detectors and YMC ODS-AM (5 μm, 250 × 10 mm; YMC Co, Kyoto, Japan), Hydro-RP (4 μm, 250 × 10 mm; Phenomenex, Torrance, CA, USA), YMC Chiral NEA (R)-NP (5 μm, 250 × 4.6 mm; YMC Co) and HyperClone ODS (5 μm, 250 × 4.6 mm; Phenomenex) columns.

*S*-(+)- and *R*-(−)-a-methoxy-a-(trifluoromethyl)phenylacetyl chloride (MTPA-Cl) were obtained from Sigma-Aldrich (Merck, Darmstadt, Germany).

### 4.2. Fungal Strain 

The strain of the fungus *Penicillium yezoense* KMM 4679 was isolated from the rhizosphere of the seagrass *Zostera marina* (Sea of Japan). The strain was stored at the Collection of Marine Microorganisms (KMM) of the G.B. Elyakov Pacific Institute of Bioorganic Chemistry (Vladivostok, Russia).

### 4.3. DNA Extraction and Amplification

The detailed methodology is described in the Supplementary Materials (on page 6 and Table S1).

### 4.4. Phylogenetic Analysis

The *ITS* region, the partial *BenA*, *CaM* and *RPB2* gene sequences of the fungal strain KMM 4679, and members of the genus *Penicillium* section *Aspergilloides*, series *Thomiorum*, were aligned by MEGA X software version 11.0.9 [37] using the Clustal W algorithm. A search for *ITS*, *BenA*, *CaM* and *RPB2* sequences of ex-type strains was performed in the GenBank database (http://ncbi.nlm.nih.gov, accessed on 29 May 2024) using the BLASTn algorithm (http://www.ncbi.nlm.nih.gov/BLAST, accessed on 29 May 2024). Multiple alignment of ITS, BenA, CaM and RPB2 sequences of strain KMM 4679 and sequences of ex-type strains of the genus *Penicillium* section *Aspergilloides*, series *Thomiorum*, and their phylogenetic analysis were carried out using MEGA X software (version 11.0.9) [37]. The phylogenetic tree was built based on the aligned combined sequences of *ITS*, *BenA*, *CaM* and *RPB2* using the ML algorithm and the selected optimal evolutionary model: the Kimura 2-parameter model [38]. A bootstrap test (1000 replicates) was used for statistical support. The sequences of the *Talaromyces marneffei* strain CBS 388.87T were used as an outgroup for phylogenetic analysis (Table S1).

### 4.5. Cultivation of P. yezoense KMM 4679

The fungus was grown as described in [39]. 

### 4.6. Extraction and Isolation 

At the end of the incubation period, the mycelia together with the medium was extracted with EtOAc (5 L). The obtained extract was dried in vacuo. The residue was dissolved in H_2_O−EtOH (4:1) (300 mL) and was extracted with *n*-hexane (0.2 L × 3) and EtOAc (0.2 L × 3). After evaporation of the EtOAc layer, the residual material (8.74 g) was passed over a Si gel, which was eluted followed by a step gradient from 100% *n*-hexane to 100% EtOAc (total volume 50 L). Fractions of 15 mL were collected and combined based on TLC analysis (Si gel, toluene–isopropanol, 6:1 and 3:1, *v*/*v*).

The *n*-hexane–EtOAc (60:40) (240 mg) eluate was purified on a YMC ODS-AM column eluting with CH_3_CN–H_2_O (80:20) to yield subfractions Zp-107-2-B-1 (36 mg) and Zp-107-2-B-2 (115.8 mg). 

Subfraction Zp-107-2-B-1 (36 mg) was purified on a Hydro-RP column eluting with CH_3_CN−H_2_O (40:60) to yield **1** (2.5 mg), **8** (1.7 mg) and subfraction Zp-107-2-B-1-1 (15.3 mg), which was purified on a HyperClone column eluting with MeOH−H_2_O (45:55) to yield **5** (3.2 mg), **6** (5.3 mg) and **7** (4.7 mg).

Subfraction Zp-107-2-B-2 (115.8 mg) was separated on a Hydro-RP column eluting with CH_3_CN–H_2_O (70:30) to yield subfraction Zp-107-2-B-2-2 (63.4 mg). Subfraction Zp-107-2-B-2-2 was purified on a Fusion column eluting with CH_3_CN−H_2_O (45:55) to yield **11** (6.5 mg).

The *n*-hexane–EtOAc (40:60) (620 mg) eluate was purified on a YMC ODS-AM column eluting with CH_3_CN–H_2_O (60:40) to yield subfractions Zp-107-2-C-1 (205.7 mg) and Zp-107-2-C-2 (67 mg). 

Subfraction Zp-107-2-C-1 (205.7 mg) was separated on a Hydro-RP column eluting with MeOH−H_2_O (55:45) to yield **2** (2.3 mg) and subfractions Zp-107-2-C-1-2 (15 mg), Zp-107-2-C-1-3 (75 mg) and Zp-107-2-C-1-4 (17 mg). Subfraction Zp-107-2-C-1-2 (15 mg) was purified on a YMC Chiral NEA (R)-NP column eluting with CH_3_CN–H_2_O (70:30) to yield **12** (12 mg). Subfraction Zp-107-2-C-1-3 (75 mg) was purified on a YMC Chiral NEA (R)-NP column eluting with MeOH−H_2_O (30:70) to yield **13** (7.9 mg). Subfraction Zp-107-2-C-1-4 (17 mg) was purified on a YMC Chiral NEA (R)-NP column eluting with MeOH–H_2_O (30:70) to yield **3** (1.3 mg) and **4** (3.9 mg).

Subfraction Zp-107-2-C-2 (67 mg) was separated on a Hydro-RP column eluting with CH_3_CN−H_2_O (60:40) to yield subfractions Zp-107-2-C-2-1 (15.7 mg) and Zp-107-2-C-2-2 (5.6 mg). Subfraction Zp-107-2-C-2-1 (15.7 mg) was purified on a YMC Chiral NEA (R)-NP column eluting with CH_3_CN−H_2_O (25:75) to yield **9** (9.3 mg) and **14** (2.3 mg). Subfraction Zp-107-2-C-2-2 (5.6 mg) was purified on a YMC Chiral NEA (R)-NP column eluting with CH_3_CN−H_2_O (40:60) to yield **10** (3.4 mg).

### 4.7. Spectral Data

Zosteropenilline M (**1**): colorless amorphous; [α]_D_^20^ +161.4 (c 0.32 MeOH); CD (c 3.8 mM, MeOH), λ_max_ (∆ε) 208 (+3.84), 295 (+2.25) nm; ^1^H and ^13^C NMR data, see Table 1 and Table 2, Supplementary Figures S2–S8; HRESIMS [M + Na]^+^ *m/z* 275.1611 (calcd. for C_15_H_24_O_3_Na 272.1617, ∆ –2.4 ppm) (Figure S1).

11-*epi*-8-hydroxyzosteropenilline M (**2**): colorless amorphous; [α]_D_^20^ −57.6 (c 0.066 MeOH); CD (c 2.4 mM, MeOH), λ_max_ (∆ε) 204 (−5.49), 243 (−0.36), 277 (+0.07), 330 (+0.08) nm; ^1^H and ^13^C NMR data, see Table 1 and Table 2, Supplementary Figures S10–S16; HRESIMS [M + Na]^+^ *m/z* 291.1542 (calcd. for C_15_H_24_O_4_Na 291.1577, ∆−8.5 ppm (Figure S1).

Zosteropenilline N (**3**): colorless amorphous; [α]_D_^20^ −60.3 (c 0.058 MeOH); CD (c 2.4 mM, MeOH), λ_max_ (∆ε) 213 (+0.55), 290 (+1.36) nm; ^1^H and ^13^C NMR data, see Table 1 and Table 2, Supplementary Figures S18–S24; HRESIMS [M + Na]^+^ *m/z* 261.1473 (calcd. for C_14_H_22_O_3_Na 261.1468, ∆–4.6 ppm), [M − H]^-^ *m/z* 237.1498 (calcd. for C_14_H_21_O_3_ 237.1496, ∆ –0.8 ppm) (Figure S17).

8-hydroxyzosteropenilline G (**4**): colorless amorphous; [α]_D_^20^ −44.1 (c 0.17 MeOH); CD (c 1.3 mM, MeOH), λ_max_ (∆ε) 213 (+0.40), 229 (−0.10), 295 (−2.16) nm; ^1^H and ^13^C NMR data, see Table 1 and Table 2, Supplementary Figures S26–S31; HRESIMS [M + Na]^+^ *m/z* 291.1579 (calcd. for C_15_H_24_O_4_Na 291.1567, ∆−4.1 ppm), [M − H]^−^ *m/z* 267.1598 (calcd. for C_15_H_23_O_4_ 267.1602, ∆ 1.5 ppm) (Figure S25).

Zosteropenilline O (**5**): colorless amorphous; [α]_D_^20^ −31.5 (c 0.32 MeOH); CD (c 5.8 mM, MeOH), λ_max_ (∆ε) 213 (+0.46), 291 (−1.81) nm; ^1^H and ^13^C NMR data, see Table 1 and Table 2, Supplementary Figures S33–S39; HRESIMS [M + Na]^+^ *m/z* 261.1471 (calcd. for C_14_H_22_O_3_Na 261.1461, ∆−3.8 ppm), [M − H]^−^ *m/z* 237.1496 (calcd. for C_14_H_21_O_3_ 237.1496, ∆ 0.0 ppm) (Figure S32).

Zosteropenilline P (**6**): colorless amorphous; [α]_D_^20^ −54.3 (c 0.53 MeOH); CD (c 4.2 mM, MeOH), λ_max_ (∆ε) 201 (−1.81), 213 (+0.29), 291 (−1.61) nm; ^1^H and ^13^C NMR data, see Table 1 and Table 2, Supplementary Figures S41–S47; HRESIMS [M + Na]^+^ *m/z* 261.1470 (calcd. for C_14_H_22_O_3_Na 261.1461, ∆–3.4 ppm), [M − H]^−^ *m/z* 237.1491 (calcd. for C_14_H_21_O_3_ 237.1496, ∆2.1 ppm) (Figure S40).

Zosteropenilline Q (**7**): colorless amorphous; [α]_D_^20^ −64.0 (c 0.47 MeOH); CD (c 4.2 mM, MeOH), λ_max_ (∆ε) 215 (+0.49), 291 (−1.99) nm; ^1^H and ^13^C NMR data, see Table 1 and Table 2, Supplementary Figures S49–S55; HRESIMS [M + Na]^+^ *m/z* 261.1469 (calcd. for C_14_H_22_O_3_Na 261.1461, ∆−3.1 ppm), [M − H]^−^ *m/z* 237.1495 (calcd. for C_14_H_21_O_3_ 237.1496, ∆0.4 ppm) (Figure S48).

13-dehydroxypallidopenilline A (**8**): colorless amorphous; [α]_D_^20^ +53.6 (c 0.14 MeOH); CD (c 2.2 mM, MeOH), λ_max_ (∆ε) 219 (−0.76), 222 (−0.77), 236 (+1.05), 289 (−0.58), 328 (+0.16) nm; ^1^H and ^13^C NMR data, see Table 1 and Table 2, Supplementary Figures S57–S63; HRESIMS [M + Na]^+^ *m/z* 275.1613 (calcd. for C_15_H_24_O_3_Na 275.1617, ∆−1.4 ppm) (Figure S56).

Zosteropenilline R (**9**): colorless amorphous; [α]_D_^20^ −93.7 (c 0.24 MeOH); CD (c 1.9 mM, MeOH), λ_max_ (∆ε) 208 (+4.00), 230 (−0.09), 293 (−2.00) nm; ^1^H and ^13^C NMR data, see Table 1 and Table 2, Supplementary Figures S65–S71; HRESIMS [M + Na]^+^ *m/z* 291.1579 (calcd. for C_15_H_24_O_4_Na 291.1567, ∆−4.4 ppm), [M − H]^−^ *m/z* 367.1604 (calcd. for C_15_H_23_O_3_ 267.1602, ∆–0.7 ppm) (Figure S64).

Zosteropenilline S (**10**): colorless amorphous; [α]_D_^20^ −91.5 (c 0.042 MeOH); CD (c 1.9 mM, MeOH), λ_max_ (∆ε) 195 (+0.99), 208 (−0.39), 295 (−2.87) nm; ^1^H and ^13^C NMR data, see Table 1 and Table 2, Supplementary Figures S73–S79; HRESIMS [M + Na]^+^ *m/z* 327.1346 (calcd. for C_15_H_25_ClO_4_Na 327.1334, ∆–4.7 ppm), [M − H]^−^ *m/z* 303.1371 (calcd. for C_15_H_24_ClO_4_ 303.1369, ∆−0.7 ppm) (Figure S72).

Zosteropenilline G (**11**): white solid; [α]_D_^20^ −64.7 (c 0.65 MeOH); CD (c 4.6 mM, MeOH), λ_max_ (∆ε) 213 (+0.55), 295 (−2.59) nm; ^1^H and ^13^C NMR as previously published [18] (Figures S81 and S82); HRESIMS [M + Na]^+^ *m/z* 275.1621 (calcd. for C_15_H_24_O_3_Na 275.1611, ∆ 1.21 ppm) (Figure S80).

Zosteropenilline J (**12**): colorless crystals; [α]_D_^20^ −51.8 (c 0.078 MeOH); ^1^H and ^13^C NMR as previously published [18] (Figures S84 and S85); HRESIMS [M + Na]^+^ *m/z* 291.1578 (calcd. for C_15_H_24_O_4_Na 291.1567, ∆−3.8 ppm), [M − H]^−^ *m/z* 267.1603 (calcd. for C_15_H_23_O_4_ 267.1602, ∆−0.4 ppm) (Figure S83).

Pallidopenilline A (**13**): colorless crystals; [α]_D_^20^ −148.6 (c 0.037 MeOH); ^1^H and ^13^C NMR as previously published [18] (Figures S87 and S88); ^13^C NMR spectrum (176 MHz; СDCl_3_; δ, ppm) 213.8 (C-3), 134.6 (C-12), 127.1 (C-11), 78.1 (C-9), 72.4 (C-13), 63.2 (C-4), 58.1 (C-1), 49.2 (C-2), 48.5 (C-10), 40.6 (C-8), 39.3 (C-5), 32.8 (C-7), 28.7 (C-6), 25.8 (C-14), 18.4 (C-15) (Figure S88); HRESIMS [M + Na]^+^ *m/z* 291.1571 (calcd. for C_15_H_24_O_4_Na 291.1567, ∆−1.4 ppm), [M − H]^−^ *m/z* 267.1605 (calcd. for C_15_H_23_O_4_ 267.1602, ∆−1.1 ppm) (Figure S86).

1-acetylpallidopenilline A (**14**): colorless crystals; [α]_D_^20^ −81.4 (c 0.059 MeOH); ^1^H NMR spectrum (700 MHz; СDCl_3_; δ, ppm; *J* in Hz) 6.03 (1H, dd, *J* = 10.1, 1.8, H-11), 5.53 (1H, dd, *J* = 10.1, 2.8, H-12), 4.35 (1H, dd, *J* = 12.5, 6.1 H_a_-1), 4.35 (1H, d, *J* = 12.5, 6.1, H_b_-1), 3.13 (1H, dt, *J* = 17.8, 6.4, H_a_-2), 2.88 (1H, d, *J* = 11.7, H-4), 2.83 (1H, t, *J* = 9.9, H-9), 2.73 (1H, dt, *J* = 17.8, 6.4, H_a_-2), 2.02 (3H, s, H_3_-2’), 1.80 (1H, tt, *J* = 10.3, 2.3, H-10), 1.72 (1H, dq, *J* = 13.8, 3.5, H_a_-7), 1.68 (1H, dq, *J* = 12.3, 3.2, H_a_-6), 1.63 (1H, qd, *J* = 11.5, 2.7, H-5), 1.38 (1H, m, H-8), 1.16 (3H, s, H_3_-14), 1.02 (3H, s, *J* = 6.5, H_3_-15), 1.12 (1H, m, H_b_-7), 0.91 (1H, qd, *J* = 12.4, 3.3, H_b_-6) (Figure S90); ^13^C NMR spectrum (176 MHz; СDCl_3_; δ, ppm) 209.6 (C-3), 170.8 (C-1’), 134.6 (C-12), 127.1 (C-11), 78.1 (C-9), 72.2 (C-13), 63.2 (C-4), 59.2 (C-1), 48.5 (C-10), 45.6 (C-2), 40.6 (C-8), 39.3 (C-5), 32.8 (C-7), 28.7 (C-6), 25.8 (C-14), 20.9 (C-2’), 18.4 (C-15) (Figure S91); HRESIMS [M + Na]^+^ *m/z* 333.1687 (calcd. for C_17_H_26_O_5_Na 333.1672, ∆−4.5 ppm), [M − H]^−^ *m/z* 3093.1708 (calcd. for C_17_H_25_O_5_ 309.1707, ∆−0.3 ppm) (Figure S89).

### 4.8. Preparation of (S)-MTPA and (R)-MTPA Esters of Zosteropenillines Q (***7***), R (***9***) and S (***10***)

To a solution of zosteropenillines Q (**7**), R (**9**) and S (**10**) (1 mg) in pyridine were added 4-dimethylaminopyridine (a few crystals) and (*R*)-MTPA-Cl (12 μL) at room temperature, and the mixture was stirred for 1.5 h. After evaporation of the solvent, the residue was purified by HPLC on a YMC C-18 column eluting with CH_3_CN:H_2_O (70:30) to afford the (*S*)-MTPA esters of zosteropenillines Q (**7**), R (**9**) and S (**10**), (**7b, 9b** and **10b**). The (*R*)-MTPA esters were prepared in a similar manner using (*S*)-MTPA-Cl. 

NMR data of (*R*,*S*)-MTPA esters of **7** (Figures S102 and S103). HRESIMS (**7b**) *m/z* 477.1844 [M + Na]^+^ (calcd. for C_24_H_29_F_3_O_5_Na, 477.1859) (Figure S104); HRESIMS (**7a**) *m/z* 477.1821 [M + Na]^+^ (calcd. for C_24_H_29_F_3_O_5_Na, 477.1859) (Figure S105).

NMR data of (*R*,*S*)-MTPA esters of **9** (Figures S106 and S107). HRESIMS (**9b**) *m/z* 723.2375 [M + Na]^+^ (calcd. for C_35_H_38_F_6_O_8_Na, 723.2363), *m/z* 735.2157 [M + Cl]^−^ (calcd. for C_35_H_38_СlF_6_O_8_, 735.2165) (Figure S118); HRESIMS (**9a**) *m/z* 723.2383 [M + Na]^+^ (calcd. for C_35_H_38_F_6_O_8_Na, 723.2363), *m/z* 735.2153 [M + Cl]^−^ (calcd. for C_35_H_38_СlF_6_O_8_, 735.2165) (Figure S119).

NMR data of (*R*,*S*)-MTPA esters of **7** (Figures S109–S111). HRESIMS (**10b**) *m/z* 759.2157 [M + Na]^+^ (calcd. for C_35_H_39_СlF_6_O_8_Na, 759.2130); *m/z* 771.1933 [M + Cl]^-^ (calcd. for C_35_H_39_Сl_2_F_6_O_8_, 771.1932); (Figure S112); HRESIMS (1**0a**) *m/z* 759.2162 [M + Na]^+^ (calcd. for C_35_H_39_СlF_6_O_8_Na, 759.2130); *m/z* 771.1924 [M + Cl]^−^ (calcd. for C_35_H_39_Сl_2_F_6_O_8_, 771.1932) (Figure S113).

### 4.9. Antimicrobial Activity

The yeast-like fungus *Candida albicans* KMM 455 and bacterial strains *Staphylococcus aureus* ATCC 21027 and *Escherichia coli* VKPM (B-7935) (Collection of Marine Microorganisms PIBOC FEB RAS) were cultured on solid-medium Mueller–Hinton broth with agar (16.0 g/L) in a Petri dish at 37 °C for 24 h.

The assays were performed in 96-well microplates in an appropriate Mueller–Hinton broth. Each well contained 90 µL of a bacterial or yeast-like fungal suspension (106 CFU/mL). Then, 10 µL was added of a compound diluted at concentrations from 1.5 µM to 100.0 µM using twofold dilution (DMSO concentration < 1%). Culture plates were incubated overnight at 37 °C, and the OD620 was measured using a Multiskan FC spectrophotometer (Thermo Scientific Inc., Beverly, MA, USA). The antibiotic gentamicin and antifungal agent nitrofungin were used as positive controls at 1 mg/mL; 1% DMSO in PBS served as a negative control. Examination was performed twice and in triplicate. The results were calculated as a percentage of the control data using SigmaPlot 14.0 software.

### 4.10. Biofilm Formation

The inhibition of the biofilm formation was assessed using the MTT test, as described in [40]. Mueller–Hinton broth was inoculated with 10^6^ CFU/mL of microorganisms: *C. albicans*, *S. aureus* and *E. coli* overnight cultures. A total of 90 µL of this cell suspension was then dispensed into 96-well microtiter plates containing 10 µL of compound diluted at concentrations from 1.5 µM to 100.0 µM using twofold dilution (DMSO concentration < 1%). After 24 h growth at 37 °C, the plates were washed with PBS to remove unbound cells. Next, 10 µL of MTT solution in PBS (5 mg/mL; Sigma-Aldrich, Munich, Germany) was added to each well and incubated for 2–4 h. Then, the media were carefully aspirated, and the plates were dried for 2 h. Then, 100 µL/well of DMSO was added to each well to dissolve formazan crystals, and the absorbance was measured using a plate reader according to the manufacture’s protocol. The results were reported as percentage inhibition normalized to the untreated control. The antibiotic gentamicin and antifungal agent nitrofungin were used as positive controls at 1 mg/mL; 1% DMSO in PBS served as a negative control. Examination was performed twice and in triplicate. 

### 4.11. Cell Culture

The human prostate cancer cells PC-3 and CRL-1435 and the human embryonic kidney cells HEK-293 and CRL-1573TM were purchased from the American Type Culture Collection (ATCC; Manassas, VA, USA). PC-3 and HEK-293 cells were cultured in DMEM medium containing 10% fetal bovine serum (Biolot, St. Petersburg, Russia) and 1% penicillin/streptomycin (Biolot, St. Petersburg, Russia) at 37 °C in a humidified atmosphere with 5% (*v*/*v*) CO_2_. The cells were incubated in cultural flasks until sub-confluent (~80%).

### 4.12. Cytotoxic Activity (MTT Assay)

PC-3 cells (5 × 10^3^ cells/well) and HEK 293 cells (8 × 10^3^ cells/well) were seeded in a 96-well plate and incubated overnight. Then, compounds were added at concentrations of 1–100 μM and incubated for another 24 h. Cell viability was then determined by the MTT (3-(4,5-dimethylthiazol-2-yl)-2,5-diphenyltetrazolium bromide) method according to the manufacturer’s instructions (Sigma-Aldrich, St. Louis, MO, USA). The absorbance of the converted formazan was measured using a Multiskan FC microplate photometer (Thermo Scientific, Waltham, MA, USA) at λ = 570 nm. The results are presented as percentages of the control data. 

### 4.13. Colony Formation Assay

The influence of compounds on colony formation by PC-3 cells was tested by the clonogenic assay [41]. The concentration of PC-3 cells was 0.33 × 10^3^/mL. The cells were incubated for 10 days, and then they were fixed with methanol (25 min), staining with 0.5% solution of crystal violet (25 min) and washing with PBS. The counting of grown colonies was carried out using a BIO-PRINT-Cx4 Edge-Fixed Pad-Container (Vilber, Collegien, France) using Bio-Vision Software user and service manual-v18.01 (Vilber, Collegien, France). The results are presented as colony inhibition in comparison with the control.

### 4.14. Migration Assay 

Silicone 2-well inserts (Ibidi^®^, Gräfelfing, Germany) were used for formation of a free cell zone in the PC-3 cells’ monolayer. After removing the insert, the gap between the cells was 500 ± 50 μm. The cells were washed twice with PBS after removing the inserts and staining with (5,6)-carboxyfluorescein succinimidyl ester (CFDA SE) (Lumiprobe, Moscow, Russia) fluorescence dye. Then, cells were treated with compound or culture medium only (vehicle control) for 24 h. Cell migration into the wound area was observed under a fluorescence microscope (MIB-2-FL; LOMO, St. Petersburg, Russia) with objective 10× magnification.

### 4.15. Drug Combination Study

Experiments were performed as previously reported [42]. To study the synergistic cytotoxic effect of the compound in combination with the clinically used anticancer drug doxorubicin, the PC-3 cells (5 × 10^3^ cells/well in 96-well plates) were simultaneously treated with compound and doxorubicin at different concentrations. After 24 h of incubation, the cell viability was determined using the MTT assay described above.

### 4.16. Hypoxia

The HEK-293 cells were exposed to cobalt chloride (II) at a 500 µM for 1 h and then the compounds were added. After 24 h, the viability of the cells was measured via MTT assay as described earlier.

### 4.17. Lipid Peroxidation Level

The fluorescence probe MitoCLox (Lumiprobe, Moscow, Russia) was used for the detection of the lipid peroxidation level in CoCl_2_-treated cells. It was added in the cells for 1 h at a concentration of 200 nM. After 1 h, the cells were washed with PBS and fluorescence was measured in ratiometric mode with λem = 520/590 nm using a PHERAStar FS plate reader (BMG Labtech, Offenburg, Germany). The data were processed with MARS Data Analysis v. 3.01R2 (BMG Labtech, Offenburg, Germany) and calculated as a 520/590 ratio.

### 4.18. Statistical Data Evaluation

All the data were obtained in three independent replicates, and the calculated values are expressed as the mean ± standard error of the mean (SEM). A Student’s *t*-test was performed using SigmaPlot 14.0 (Systat Software Inc., San Jose, CA, USA) to determine the statistical significance. The differences were considered statistically significant at *p* < 0.05.

### 4.19. Quantum Chemical Modeling

The B3LYP exchange-correlation functional, the polarization continuum model (PCM) and 6-311G(d) basis set implemented in the Gaussian 16 package of programs were used for all quantum chemical calculations [43]. For compounds **1** and **3**, conformational analysis was performed, and the significant (most stable) conformations were selected for further modeling of ECD spectra.

The statistical weights (*g_im_*) of individual conformations were calculated according to the following equation:

(1) gim=e−ΔGim/RT∑ie−ΔGim/RT

where Δ*G_im_* = *G_i_* − *G_m_* are the relative Gibbs free energies and index “*m*” denotes the most stable conformation. 

The ECD spectra were calculated using time-dependent density functional theory (TDDFT), the B3LYP functional, the PCM model and the 6-311G(d) basis set for conformations, of which relative Gibbs free energies satisfied to relation ΔG*_im_* ≤ 4 kcal/mol. Gauss-type functions were used for simulating the individual bands in theoretical spectra. The bandwidths ζ = 0.36 eV for **1** and ζ = 0.24 eV for **3** were used. The UV shifts Δλ = +4 nm were used to obtain the best correspondence between the experimental and calculated spectra for **1** and **3**. 

The scaled theoretical and experimental ECD spectra were obtained according to the following equation:

(2) Δεsc(λ)=Δε(λ)Δε(λpeak)

where the denominator |Δε(λ_peak)_| is a modulo of the peak value for the positive characteristic band at λ ≈ 290 nm in the corresponding ECD spectrum.

## 5. Conclusions

Ten new polyketides, named zosteropenilline M (**1**), 11-*epi*-8-hydroxyzosteropenilline M (**2**), zosteropenilline N (**3**), 8-hydroxyzosteropenilline G (**4**), zosteropenilline O (**5**), zosteropenilline P (**6**), zosteropenilline Q (**7**), 13-dehydroxypallidopenilline A (**8**), zosteropenilline R (**9**) and zosteropenilline S (**10**), were isolated from the ethyl acetate extract of the marine-derived fungus *Penicillium yezoense* KMM 4679. The absolute configurations of **1** and **3** were determined by time-dependent density functional theory (TD-DFT) calculations of the ECD spectra. The absolute configurations of **7, 9** and **10** were determined by a combination of the modified Mosher’s method and ROESY data together with biogenetic relationships. A biogenetic pathway for **1**−**14** was proposed. Zosteropenillines O, К and S diminished cobalt chloride (II)-induced HEK-293 cell damage and recovered the high lipid peroxide oxidation level in the mitochondria of these cells. 1-Acetylpallidopenilline A exhibited strong inhibition of human breast cancer MCF-7 cell colony formation and may be interesting for future studies as a new estrogen receptor modulator.

## Figures and Tables

**Table 1:** ^13^С NMR spectroscopic data (**δ_С_** in ppm**,** type) for compounds **1**–**5**.

No.	1 *	2 *	3 *	4 **	5 *
1	57.8, CH_2_	58.0, CH_2_	–	58.1, CH_2_	–
2	43.1, CH_2_	43.7, CH_2_	28.0, CH_3_	49.2, CH_2_	34.7, CH_3_
3	214.6, C	214.5, C	211.2, C	214.1, C	211.1, C
4	62.2, CH	62.4, CH	63.4, CH	63.2, CH	63.5, CH
5	38.9, CH	33.6, CH	34.1, CH	41.0, CH	40.8, CH
6	31.4, CH_2_	27.0, CH_2_	31.3, CH_2_	24.9, CH_2_	24.9, CH_2_
7	34.3, CH_2_	38.0, CH_2_	28.8, CH_2_	38.7, CH_2_	38.7, CH_2_
8	31.7, CH	70.0, C	40.2, C	69.8, C	69.8, C
9	38.5, CH_2_	40.4, CH_2_	31.3, CH_2_	44.6, CH_2_	44.7, CH_2_
10	45.3, CH	33.6, CH	41.4, CH	37.0, CH	37.0, CH
11	72.6, CH	66.8, CH	68.6, CH	131.6, CH	131.5, CH
12	129.8, CH	127.5, CH	127.1, CH	134.2, CH	134.4, CH
13	132.0, C	135.4, C	135.7, C	73.0, C	72.7, C
14	21.1, CH_3_	21.7, CH_3_	21.6, CH_3_	26.0, CH_3_	25.7, CH_3_
15	22.5, CH_3_	32.0, CH_3_	67.0, CH_2_	31.6, CH_3_	31.6, CH_2_

* Chemical shifts were measured at 176.04 MHz in CDCl_3_; ** Chemical shifts were measured at 125.77 MHz in CDCl_3_.

**Table 2:** ^1^H NMR spectroscopic data δ_H_, mult. (*J* in Hz) for compounds **1**–**5**.

Position	1 *	2 *	3 *	4 **	5 *
1	3.85, brs (2H)	3.86, d (5.5) 3.85, d (5.5)	–	3.92, ddd (11.0, 7.3, 3.7) 3.86, ddd (10.5, 6.7, 3.8)	–
2	2.70, ddd (18.1, 6.5, 4.6) 2.64, ddd (18.1, 6.2, 4.5)	2.79, ddd (18.3, 10.8, 5.4) 2.68, ddd (18.3, 10.7, 5.3)	2.14, s	3.07, ddd (17.9, 6.7, 3.7) 2.62, ddd (18.1, 7.1, 3.8)	2.29, s
4	2.83, brd (10.0)	2.86, brd (9.9)	2.74, brd (9.9)	2.95, d (11.6)	2.90, d (11.6)
5	1.52, qd (11.5, 4.0)	1.76, m	1.76, m	1.50, m	1.47, m
6	1.65, dq (13.1, 3.4) 1.14, qd (13.0, 3.7)	1.55, m 1.51, m	1.73–1.82, m 1.13, m	1.58, m 1.27, m	1.62, tq (16.7, 3.0) 1.26, m
7	1.69, dq (13.1, 3.1) 0.89, qd (13.1, 3.4)	1.61, m 1.38, td (13.4, 5.0)	1.79, m 0.96, m	1.64, dq (14.0, 3.1) 1.44, qd (13.6, 4.1)	1.64, m 1.45, m
8	1.40, m	–	1.60, m	–	–
9	2.21, dq (12.8, 3.4) 0.70, dd (12.7, 12.0)	1.65, m 1.59, m	1.73–1.82, m 1.27, m	1.70, dt (13.5, 3.0) 1.19 (13.3)	1.70, dt (13.5, 2.9) 1.20, t (13.3)
10	1.16, m	1.68, tt (12.0, 3.4)	1.32, tt (11.5, 3.3)	2.31, m	2.29, m
11	3.88, brd (9.0)	3.86, dd (5.5, 3.3)	3.92, dd (5.6, 3.1)	5.41, dd (9.9, 1.7)	5.40, dd (9.9, 1.5)
12	5.59, q (1.6)	5.89, dt (5.8, 1.6)	5.88, brd (5.8)	5.46, dd (9.9, 2.9)	5.48, dd (9.9, 2.9)
14	1.60, s	1.63, brs	1.63, brs	1.20, s	1.19, s
15	0.92, d (6.5)	1.28, s	3.51, dd (5.7, 1.2) 3.50, dd (5.7, 1.3)	1.24, s	1.24, s

* Chemical shifts were measured at 700 MHz in CDCl_3_; ** Chemical shifts were measured at 500 MHz in CDCl_3_^.^.

**Table 3:** ^13^С NMR spectroscopic data (**δ_С_** in ppm, type) for compounds **6**–**10**.

No.	6 *	7 *	8 *	9 *	10 *
1	–	–	57.8, CH_2_	58.1, CH_2_	58.3, CH_2_
2	34.8, CH_3_	34.7, CH_3_	45.2, CH_2_	49.4, CH_2_	49.1, CH_2_
3	211.1, C	210.9, C	213.1, C	214.5, C	214.5, C
4	63.7, CH	63.5, CH	55.6, CH	57.9, CH	60.4, CH
5	41.4, CH	39.3, CH	33.9, CH	39.4, CH	34.7, CH
6	28.8, CH_2_	28.7, CH_2_	28.8, CH_2_	32.8, CH_2_	30.8, CH_2_
7	29.0, CH_2_	32.8, CH_2_	33.1, CH_2_	33.9, CH_2_	33.9, CH_2_
8	40.6, CH	40.6, CH	40.6, CH	33.6, CH	32.4, CH
9	35.3, CH_2_	78.1, CH	78.6, CH	43.0, CH_2_	38.1, CH_2_
10	41.5, CH	48.5, CH	48.4, CH	145.3, C	39.4, CH
11	131.6, CH	127.0, CH	126.1, CH	118.2, CH	63.3, CH
12	134.2, CH	134.8, CH	131.7, CH	72.3, CH	78.5, CH
13	72.6, C	72.1, C	31.5, CH	73.4, CH	73.4, C
14	25.7, CH_3_	25.5, CH_3_	17.4, CH_3_	19.9, CH_3_	24.3, CH_3_
15	68.2, CH_2_	18.4, CH_3_	18.5, CH_3_	22.3, CH_3_	22.4, CH_3_

* Chemical shifts were measured at 176.04 MHz in CDCl_3_.

**Table 4:** ^1^H NMR spectroscopic data δ_H_, Mult. (*J* in Hz) for compounds **6**–**10**.

Position	6 *	7 *	8 *	9 *	10 *
1			3.88, ddd (11.2, 6.8, 3.8) 3.84, ddd (11.2, 6.8, 3.8)	3.91, ddd (11.2, 7.0, 3.6) 3.86, ddd (11.2, 7.0, 3.6)	3.90, ddd (11.0, 7.5, 3.6) 3.86, ddd (10.5, 6.5, 3.6)
2	2.28, s	2.29, s	2.78, ddd (18.1, 6.8, 3.8) 2.55, ddd (18.1, 6.8, 3.8)	3.08, ddd (18.0, 6.8, 3.8) 2.61, ddd (18.0, 6.8, 3.8)	3.05, ddd (17.8, 7.5, 3.6) 2.54, ddd (17.8, 6.4, 3.6)
4	2.86, d (11.7)	2.88, d (11.8)	2.82, dd (11.2, 6.0)	2.90, d (10.2)	2.87, d (11.3)
5	1.49, qd (11.7, 3.0)	1.59, m	1.59, qd (11.2, 3.0)	2.38, td (10.2, 5.0)	1.77, qd (11.3, 3.5)
6	1.83, m 0.94, qd (12.1, 3.4)	1.71, m 0.94, m	1.85, dq (12.6, 3.1) 0.84, qd (12.6, 3.5)	1.67, m 1.02, m	1.66, m 0.90, m
7	1.80, m 1.03, qd (12.5, 4.0)	1.73, m 1.13, m	1.75, dq (13.8, 3.6) 1.17, qd (13.3, 3.9)	1.65, m 1.03, m	1.61, m 0.90, m
8	1.61, m	1.39, m	1.39, m	1.43, m	1.43, m
9	1.87, m 0.82, q (12.5)	2.83, t (9.8)	2.86, t (9.9)	2.22, m 1.65, m	1.50, dq (13.1, 3.1) 1.22, m
10	1.86, m	1.80, tt (10.0, 2.2)	1.71, tq (10.6, 2.3)	–	1.91, tt (11.3, 3.4)
11	5.45, s	6.02, dd (10.0, 1.4)	5.99, brd (10.0)	5.50, dt (5.8, 2.1)	4.15, t (3.1)
12	5.45, s	5.54, dd (10.0, 2.7)	5.67, ddd (10.0, 4.4, 2.7)	3.61, dd (4.8, 0.9)	3.76, dd (2.8)
13	–	–	2.57, dd (6.0, 4.0)	–	–
14	1.18, s	1.18, s	0.83, d (7.2)	1.06, s	1.42, s
15	3.48, dd (10.5, 6.9) 3.45, dd (10.5, 6.6)	1.03, d (6.4)	1.04, d (6.4)	0.90, d (6.5)	0.91, d (6.5)

* Chemical shifts were measured at 700 MHz in CDCl_3_.

**Table 5:** Antimicrobial activity of isolated compounds ^1^.

Compound	Inhibition of Microbial Growth, % of Control		
	*S. aureus*	*E. coli*	*C. albicans*
**1**	30.08 ± 2.42	0	8.32 ± 0.62
**2**	0	0	6.12 ± 1.89
**4**	0	0	10.98 ± 2.31
**5**	0	0	35.31 ± 1.16
**6**	28.99 ± 1.66	0	17.34 ± 2.55
**7**	29.95 ± 4.50	0	19.21 ± 3.12
**8**	19.86 ± 1.82	0	0
**9**	11.66 ± 1.71	0	12.03 ± 1.14
**10**	15.73 ± 0.36	0	0
**11**	16.95 ± 4.12	0	18.10 ± 2.58
**12**	20.31 ± 1.20	0	6.61 ± 1.12
**13**	15.49 ± 1.35	0	0
**14**	31.65 ± 7.80	0	0
Gentamicin/nitrofungin	98.61 ± 1.15	97.56 ± 2.10	98.13 ± 0.69

^1^ The concentration of each compound was 100 µM.

**Table 6:** Cytotoxic activity of isolated compounds ^1^.

Compound	Cell Viability, % of Control			
	НЕК-298	РС-3	HeLa	MCF-7
**1**	95.16 ± 4.01	89.25 ± 0.44	66.49 ± 6.19	86.42 ± 1.76
**2**	90.98 ± 2.82	90.98 ± 2.82	44.12 ± 5.13	57.46 ± 1.82
**4**	94.71 ± 1.53	94.71 ± 1.53	81.80 ± 6.76	81.92 ± 3.92
**5**	85.64 ± 1.85	91.20 ± 3.37	37.12 ± 3.85	77.40 ± 2.73
**6**	83.26 ± 3.78	83.15 ± 1.72	71.44 ± 3.99	82.05 ± 1.31
**7**	80.00 ± 0.99	88.70 ± 1.10	80.80 ± 3.25	76.23 ± 4.53
**8**	86.10 ± 2.24	83.60 ± 1.81	72.66 ± 2.49	81.21 ± 5.56
**9**	96.45 ± 7.64	96.44 ± 7.63	79.14 ± 6.36	79.64 ± 1.67
**10**	98.10 ± 1.15	85.98 ± 2.13	68.69 ± 6.21	84.85 ± 4.90
**11**	93.97 ± 2.36	93.97 ± 2.36	34.35 ± 1.90	81.00 ± 2.38
**12**	87.01 ± 2.71	87.01 ± 2.71	66.70 ± 6.64	82.77 ± 1.36
**13**	89.76 ± 3.31	89.76 ± 3.31	71.85 ± 3.02	95.05 ± 1.51
**14**	96.92 ± 4.83	45.91 ± 0.67	53.44 ± 2.14	23.71 ± 1.05

^1^ The concentration of substances was 100 µM.

## Data Availability

The original data presented in this study are included in the article/Supplementary Materials; further inquiries can be directed to the corresponding author.

## Supplementary Materials

The following supporting information can be downloaded at: https://www.mdpi.com/article/10.3390/md22070317/s1, Table S1. The strains of the species used in multi-locus phylogenetic analysis and GenBank accession numbers; Figures S1, S9, S17, S25, S32, S40, S48, S56, S64, S72, S80, S83, S86 and S89 HRESIMS for **1**–**14**; Figures S2–S8. NMR spectra of **1**; Figures S10–S16. NMR spectra of **2**; Figures S18–S24. NMR spectrum of **3**; Figures S26–S31. NMR spectra of **4**; Figures S33–S39. NMR spectra of **5**; Figures S41–S47. NMR spectra of **6**; Figures S49–S55. NMR spectra of **7**. Figures S57–S63. NMR spectra of **8**; Figures S65–S71. NMR spectra of **9**. Figures S73–S79. NMR spectra of **10**; Figures S81 and S82. NMR spectra of **11**; Figures S84 and S85. NMR spectra of **12**. Figures S87 and S88. NMR spectra of **13**; Figures S90 and S91. NMR spectra of **14**; Figures S92–S101. UV and CD data for **1**–**10**; Figures S102, S103 and S106–S111. NMR spectra of ((*S*)- and (*R*) MTPA esters); Figures S104, S105, S112, S113, S118, S119, S120 and S121. HRESIMS for ((*S*)- and (*R*) MTPA esters); Figure S106. ^1^H NMR spectrum of **9b** ((S)-MTPA ester); Figure S114. The main conformations of **1**. Figure S115. ECD spectra, calculated for different conformations of **1**. Figure S116. ECD spectra, calculated for different conformations of **3**; Figure S117. ECD spectra, simulated for different relative amounts of conformations with dihedral angle θ1 = ∠O−C−C−H ≈ 180° and θ1 = ∠O−C−C−H ≈ 0°; Figure S118. HPLC-MS/MS Analysis; Figure S119. Key HMBC (blue arrows) and ^1^H-1H COSY (bold lines) correlations of **1**–**10**.
