# Supplementary material for: New Zosteropenillines and Pallidopenillines from the Seagrass-Derived Fungus Penicillium yezoense KMM 4679
Source: Mar Drugs. 2024 Jul 17;22(7):317. doi: 10.3390/md22070317 (PMC11277992; doi:10.3390/md22070317)
Supplement: Supplementary file 1 [file marinedrugs-22-00317-s001.zip › marinedrugs-3102332-supplementary.pdf]

# Supporting Information

# New Zosteropenillines and Pallidopenillines from the Seagrass-Derived Fungus *Penicillium yezoense* KMM 4679

**Elena V. Leshchenko [1,2,*,+], Ekaterina A. Chingizova [1,+], Alexandr S. Antonov [1], Nadezhda P. Shlyk [2], Gleb V. Borkunov [1,2], Dmitrii V. Berdyshev [1], Viktoria E. Chausova [1], Natalya N. Kirichuk [1], Yuliya V. Khudyakova [1], Artur R. Chingizov [1], Anatoly I. Kalinovskiy [1], Roman S. Popov [1], Natalya Yu. Kim [1], Ksenia A. Chadova [3], Ekaterina A. Yurchenko [1], Marina P. Isaeva [1] and Anton N. Yurchenko [1]**

Contains

*DNA Extraction and Amplification* ........ 6

Table S1. The strains of the species used in multi-locus phylogenetic analysis and GenBank accession numbers. ........ 6

Figure S1 HRESIMS for 1 ........ 8

Figure S2. $^1$H NMR spectrum of 1 measured at 700 MHz in $CDCl_3$ ........ 9

Figure S3. $^{13}$C NMR spectrum of 1 measured at 176 MHz in $CDCl_3$ ........ 10

Figure S4. DEPT-135 spectrum of 1 measured at 176 MHz in $CDCl_3$ ........ 11

Figure S5. HSQC spectrum of 1 measured in $CDCl_3$ ........ 12

Figure S6. COSY spectrum of 1 measured in $CDCl_3$ ........ 13

Figure S7. HMBC spectrum of 1 measured in $CDCl_3$ ........ 14

Figure S8. ROESY spectrum of 1 measured in $CDCl_3$ ........ 15

Figure S9. HRESIMS for 2 ........ 16

Figure S10. $^1$H NMR spectrum of 2 measured at 700 MHz in $CDCl_3$ ........ 17

Figure S11. $^{13}$C NMR spectrum of 2 measured at 176 MHz in $CDCl_3$ ........ 18

Figure S12. DEPT-135 spectrum of 2 measured at 176 MHz in $CDCl_3$ ........ 19

**Figure S13. HSQC spectrum of 2 measured in $CDCl_3$** ........ 20
**Figure S14. COSY spectrum of 2 measured in $CDCl_3$** ........ 21
**Figure S15. HMBC spectrum of 2 measured in $CDCl_3$** ........ 22
**Figure S16. ROESY spectrum of 2 measured in $CDCl_3$** ........ 23
**Figure S17. HRESIMS for 3** ........ 24
**Figure S18. $^{1}H$ NMR spectrum of 3 measured at 700 MHz in $CDCl_3$** ........ 26
**Figure S19.$^{13}C$ NMR spectrum of 3 measured at 176 MHz in $CDCl_3$** ........ 27
**Figure S20. DEPT-135 spectrum of 3 measured at 176 MHz in $CDCl_3$** ........ 28
**Figure S21. HSQC spectrum of 3 measured in $CDCl_3$** ........ 29
**Figure S22. COSY spectrum of 3 measured in $CDCl_3$** ........ 30
**Figure S23. HMBC spectrum of 3 measured in $CDCl_3$** ........ 31
**Figure S24. ROESY spectrum of 3 measured in $CDCl_3$** ........ 32
**Figure S25. HRESIMS for 4** ........ 33
**Figure S26. $^{1}H$ NMR spectrum of 4 measured at 500 MHz in $CDCl_3$** ........ 35
**Figure S27.$^{13}C$ NMR spectrum of 4 measured at 125 MHz in $CDCl_3$** ........ 36
**Figure S28. HSQC spectrum of 4 measured in $CDCl_3$** ........ 37
**Figure S29. COSY spectrum of 4 measured in $CDCl_3$** ........ 38
**Figure S30. HMBC spectrum of 4 measured in $CDCl_3$** ........ 39
**Figure S31. ROESY spectrum of 4 measured in $CDCl_3$** ........ 40
**Figure S32. HRESIMS for 5** ........ 41
**Figure S33. $^{1}H$ NMR spectrum of 5 measured at 700 MHz in $CDCl_3$** ........ 43
**Figure S34.$^{13}C$ NMR spectrum of 5 measured at 176 MHz in $CDCl_3$** ........ 44
**Figure S35. DEPT-135 spectrum of 5 measured at 176 MHz in $CDCl_3$** ........ 45
**Figure S36. HSQC spectrum of 5 measured in $CDCl_3$** ........ 46
**Figure S37. COSY spectrum of 5measured in $CDCl_3$** ........ 47
**Figure S38. HMBC spectrum of 5 measured in $CDCl_3$** ........ 48
**Figure S39. ROESY spectrum of 5 measured in $CDCl_3$** ........ 49
**Figure S40. HRESIMS for 6** ........ 50
**Figure S41. $^{1}H$ NMR spectrum of 6 measured at 700 MHz in $CDCl_3$** ........ 52
**Figure S42.$^{13}C$ NMR spectrum of 6 measured at 176 MHz in $CDCl_3$** ........ 53
**Figure S43. DEPT-135 spectrum of 6 measured at 176 MHz in $CDCl_3$** ........ 54
**Figure S44. HSQC spectrum of 6 measured in $CDCl_3$** ........ 55
**Figure S45. COSY spectrum of 6 measured in $CDCl_3$** ........ 56
**Figure S46. HMBC spectrum of 6 measured in $CDCl_3$** ........ 57

**Figure S47. ROESY spectrum of 6 measured in $CDCl_3$** ........ 58
**Figure S48. HRESIMS for 7** ........ 59
**Figure S49. $^1H$ NMR spectrum of 7 measured at 700 MHz in $CDCl_3$** ........ 61
**Figure S50.$^{13}C$ NMR spectrum of 7 measured at 176 MHz in $CDCl_3$** ........ 62
**Figure S51. DEPT-135 spectrum of 7 measured at 176 MHz in $CDCl_3$** ........ 63
**Figure S52. HSQC spectrum of 7 measured in $CDCl_3$** ........ 64
**Figure S53. COSY spectrum of 7 measured in $CDCl_3$** ........ 65
**Figure S54. HMBC spectrum of 7 measured in $CDCl_3$** ........ 66
**Figure S55. ROESY spectrum of 7 measured in $CDCl_3$** ........ 67
**Figure S56. HRESIMS for 8** ........ 68
**Figure S57. $^1H$ NMR spectrum of 8 measured at 700 MHz in $CDCl_3$** ........ 69
**Figure S58.$^{13}C$ NMR spectrum of 8 measured at 176 MHz in $CDCl_3$** ........ 70
**Figure S59. DEPT-135 spectrum of 8 measured at 176 MHz in $CDCl_3$** ........ 71
**Figure S60. HSQC spectrum of 8 measured in $CDCl_3$** ........ 72
**Figure S61. COSY spectrum of 8 measured in $CDCl_3$** ........ 73
**Figure S62. HMBC spectrum of 8 measured in $CDCl_3$** ........ 74
**Figure S63. ROESY spectrum of 8 measured in $CDCl_3$** ........ 75
**Figure S64. HRESIMS for 9** ........ 76
**Figure S65. $^1H$ NMR spectrum of 9 measured at 700 MHz in $CDCl_3$** ........ 78
**Figure S66.$^{13}C$ NMR spectrum of 9 measured at 176 MHz in $CDCl_3$** ........ 79
**Figure S67. DEPT-135 spectrum of 9 measured at 176 MHz in $CDCl_3$** ........ 80
**Figure S68. HSQC spectrum of 9 measured in $CDCl_3$** ........ 81
**Figure S69. COSY spectrum of 9 measured in $CDCl_3$** ........ 82
**Figure S70. HMBC spectrum of 9 measured in $CDCl_3$** ........ 83
**Figure S71. ROESY spectrum of 9 measured in $CDCl_3$** ........ 84
**Figure S72. HRESIMS for 10** ........ 84
**Figure S73. $^1H$ NMR spectrum of 10 measured at 700 MHz in $CDCl_3$** ........ 89
**Figure S74.$^{13}C$ NMR spectrum of 10 measured at 176 MHz in $CDCl_3$** ........ 90
**Figure S75. DEPT-135 spectrum of 10 measured at 176 MHz in $CDCl_3$** ........ 91
**Figure S76. HSQC spectrum of 10 measured in $CDCl_3$** ........ 92
**Figure S77. COSY spectrum of 10 measured in $CDCl_3$** ........ 93
**Figure S78. HMBC spectrum of 10 measured in $CDCl_3$** ........ 94
**Figure S79. ROESY spectrum of 10 measured in $CDCl_3$** ........ 95
**Figure S80. HRESIMS for 11** ........ 96

Figure S81. $^1$H NMR spectrum of 11 measured at 700 MHz in $CDCl_3$ ....97
Figure S82.$^{13}$C NMR spectrum of 11 measured at 176 MHz in $CDCl_3$....98
Figure S83. HRESIMS for 12....98
Figure S84. $^1$H NMR spectrum of 12 measured at 700 MHz in $CDCl_3$ ....101
Figure S85.$^{13}$C NMR spectrum of 12 measured at 176 MHz in $CDCl_3$....102
Figure S86. HRESIMS for 13....103
Figure S87. $^1$H NMR spectrum of 13 measured at 700 MHz in $CDCl_3$ ....105
Figure S88.$^{13}$C NMR spectrum of 13 measured at 176 MHz in $CDCl_3$....106
Figure S89. HRESIMS for 14....107
Figure S90. $^1$H NMR spectrum of 14 measured at 700 MHz in $CDCl_3$ ....109
Figure S91.$^{13}$C NMR spectrum of 14 measured at 176 MHz in $CDCl_3$....110
Figure S92. UV and CD data for 1 in MeOH....111
Figure S93. UV and CD data for 2 in MeOH....113
Figure S94. UV and CD data for 3 in MeOH....115
Figure S95. UV and CD data for 4 in MeOH....117
Figure S96. UV and CD data for 5 in MeOH....119
Figure S97. UV and CD data for 6 in MeOH....121
Figure S98. UV and CD data for 7 in MeOH....123
Figure S99. UV and CD data for 8 in MeOH....125
Figure S100. UV and CD data for 9 in MeOH....127
Figure S101. UV and CD data for 10 in MeOH ....129
Figure S102. $^1$H NMR spectrum of 7b ((S)-MTPA ester) measured at 700 MHz in $CDCl_3$ ....131
Figure S103. $^1$H NMR spectrum of 7a ((R)-MTPA ester) measured at 700 MHz in $CDCl_3$ ....132
Figure S104. HRESIMS for 7b ((S)-MTPA ester) ....133
Figure S105. HRESIMS for 7a ((R)-MTPA ester) ....133
Figure S106. $^1$H NMR spectrum of 9b ((S)-MTPA ester) measured at 700 MHz in $CDCl_3$ ....134
Figure S107. $^1$H NMR spectrum of 9a ((R)-MTPA ester) measured at 700 MHz in $CDCl_3$ ....135
Figure S108. $^1$H NMR spectrum of 10b ((S)-MTPA ester) measured at 700 MHz in $CDCl_3$ ...136
Figure S109. HSQC spectrum of 10b ((S)-MTPA ester) measured at 700 MHz in $CDCl_3$....137
Figure S110. $^1$H NMR spectrum of 10a ((R)-MTPA ester) measured at 700 MHz in $CDCl_3$ ...138
Figure S111. HSQC spectrum of 10a ((R)-MTPA ester) measured at 700 MHz in $CDCl_3$....139
Figure S112. HRESIMS for 10b ((S)-MTPA ester)....140
Figure S113. HRESIMS for 10a ((R)-MTPA ester)....142
Figure S114. The main conformations of 1 ....144

**Figure S115. ECD spectra, calculated for different conformations of 1.** .......... 146

**Figure S116. ECD spectra, calculated for different conformations of 3.** .......... 147

**Figure S117. ECD spectra, simulated for different relative amounts of conformations with dihedral angle θ1 = ∠O–C–C–H ≈ 180° and θ1 = ∠O–C–C–H ≈ 0°** .......... 148

**Figure S118. HPLC-MS/MS Analysis** .......... 148

**Figure S119. Key HMBC (blue arrows) and 1H-1H COSY (bold lines) correlations of 1–10.** .......... 149

**Figure S120. HRESIMS for 9b ((S)-MTPA ester)** .......... 150

**Figure S121. HRESIMS for 9a ((R)-MTPA ester)** .......... 152

.

*DNA Extraction and Amplification*

Genomic DNA was isolated from fungal mycelium grown on MEA (malt extract agar) at 25 °C for 7 days using the MagJET Plant Genomic DNA Kit (Thermo Fisher Scientific, Waltham, MA, USA) according to the manufacturer's protocol. PCR was conducted using GoTaq Flexi DNA Polymerase (Promega, Madison, WI, USA). For amplification of the *ITS* region, the standard primer pair, ITS1 and ITS4 was used [1]. The reaction profile was 95 °C for 300 s; 35 cycles of 95 °C for 30 s, 55 °C for 45 s, 72 °C for 90 s; and, finally, 72 °C for 300 s. For amplification of the partial BenA gene, the primer pair, tub_P/A_F [2] and Bt-2b [3] was used. The reaction profile was 95 °C for 300 s; 35 cycles of 95 °C for 30 s, 55 °C for 45 s, and 72 °C for 90 s; and, finally, 72 °C for 300 s. For amplification of the partial CaM gene, the degenerate primer pair cal_P/A_F and cal_P/A_R was used [2]. The reaction profile was 95 °C for 300 s; 35 cycles of 95 °C for 30 s, 60 °C for 45 s, and 72 °C for 90 s; and, finally, 72 °C for 300 s. For amplification of the partial RPB2 gene, the standard primer pair, 5Feur and 7CReur was used [4]. The reaction profile used as described in [5]. The amplified *ITS, BenA, CaM*, and *RPB2* genes were purified with the ExoSAP-IT™ PCR Product Cleanup Reagent (Thermo Fisher Scientific, Waltham, MA, USA). Sequencing was bidirectional performed with the same primers on an Applied Biosystems SeqStudio Genetic Analyzer (Thermo Fisher Scientific, Waltham, MA, USA) using the Big Dye Terminator reagent kit, version 3.1. Gene sequences were deposited in GenBank under accession numbers PP854452 for *ITS*, PP861099 for the partial *BenA*, PP861100 for the partial *CaM*, and PP861101 for the partial *RPB2* gene (Table S1).


1. White, T.J.; Bruns, T.; Lee, S.; Taylor, J. Amplification and direct sequencing of fungal ribosomal RNA genes for phylogenetics. In PCR protocols: a guide to methods and applications, London, 1990; Vol. 38, pp 315-322.

2. Yurchenko, A.N.; Zhuravleva, O.I.; Khmel, O.O.; Oleynikova, G.K.; Antonov, A.S.; Kirichuk, N.N.; Chausova, V.E.; Kalinovsky, A.I.; Berdyshev, D.V.; Kim, N.Y., et al. New cyclopiane diterpenes and polyketide derivatives from marine sediment-derived fungus Penicillium antarcticum KMM 4670 and their biological activities. Mar. Drugs 2023, 21, 21110584

3. Glass, N.L.; Donaldson, G.C. Development of primer sets designed for use with the PCR to amplify conserved genes from filamentous ascomycetes. Applied Environmental Microbiology 1995, 61, 1323-1330. - https://www.scopus.com/inward/record.uri?eid=2-s2.0-0028969372&doi=10.1128%2faem.61.4.1323-1330.1995&partnerID=40&md5=ee11611f6b61ec23e654da22ba152a0f

4. J, H.; H, S.; JC, F. Rasamsonia, a new genus comprising thermotolerant and thermophilic Talaromyces and Geosmithia species. Antonie Van Leeuwenhoek 2012, 101, 403-421.

5. CM, V.; J, H.; JC, F.; SB, H.; CH, K.; G, P.; KA, S.; J, V.; T, Y.; RA, S. Identification and nomenclature of the genus Penicillium. Stud. Mycol. 2014, 78, 343-371.


**Table S1. The strains of the species used in multi-locus phylogenetic analysis and GenBank accession numbers.**

| Species | Strain Number | GenBank Accession Number | | | |
|---|---|---|---|---|---|
| | | *ITS* | *BenA* | *CaM* | *RPB2* |
| *Penicillium aurantioviolaceum* | CBS 137777$^{T}$ | KM189756 | KM089005 | KM089392 | KM089779 |

| | | | | | |
|---|---|---|---|---|---|
| *Penicillium austroafricanum* | CBS 137773[T] | KM189610 | KM088854 | KM089241 | KM089628 |
| *Penicillium cartierense* | CBS 137956[T] | KM189564 | KM088804 | KM089189 | KM089576 |
| *Penicillium contaminatum* | CBS 345.52[T] | KM189554 | KM088793 | KM089178 | KM089565 |
| *Penicillium crocicola* | CBS 745.70[T] | KM189581 | KJ834445 | KM089210 | JN406535 |
| *Penicillium fusisporum* | CBS 137463[T] | KF769424 | KF769400 | KF769413 | MN969117 |
| *Penicillium grevilleicola* | CBS 137775[T] | KM189630 | KM088874 | KM089261 | KM089648 |
| *Penicillium jejuense* | CBS 138646[T] | KF818464 | KF818461 | KF818470 | KF818467 |
| *Penicillium roseoviride* | CBS 267.35[T] | KM189549 | KM088787 | KM089172 | KM089559 |
| *Penicillium thomii* | CBS 225.81[T] | KM189560 | KM088799 | KM089184 | KM089571 |
| *Penicillium valentinum* | CBS 172.81[T] | KM189550 | KM088788 | KM089173 | KM089560 |
| *Penicillium yezoense* | CBS 350.59[T] | KM189553 | KM088792 | KM089177 | KM089564 |
| ***Penicillium yezoense*** | **KMM 4679** | PP854452 | PP861099 | PP861100 | PP861101 |
| *Talaromyces marneffei* | CBS 388.87[T] | JN899344 | JX091389 | KF741958 | KM023283 |


1. Houbraken J; Kocsubé S; Visagie CM; Yilmaz N; Wang XC; Meijer M; Kraak B; Hubka V; Bensch K; Samson RA, *et al.* Classification of *Aspergillus*, *Penicillium*, *Talaromyces* and related genera (Eurotiales): An overview of families, genera, subgenera, sections, series and species. *Stud. Mycol.* **2020**, *95*, 5-169.

**Figure S1 HRESIMS for 1**

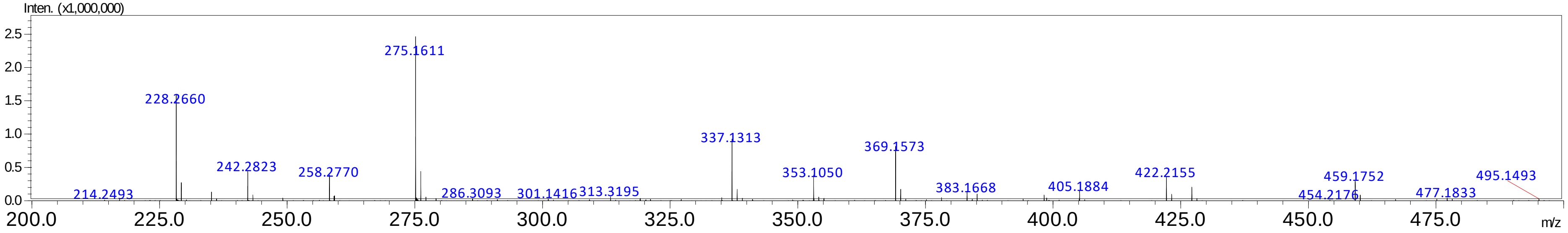


| | meas | calc | Δ (ppm) |
|---|---|---|---|
| [M+Na]+ | 275,1611 | 275,1617 | -2,4 |

**Figure S2.**[1]**H NMR spectrum of 1 measured at 700 MHz in $CDCl_3$**

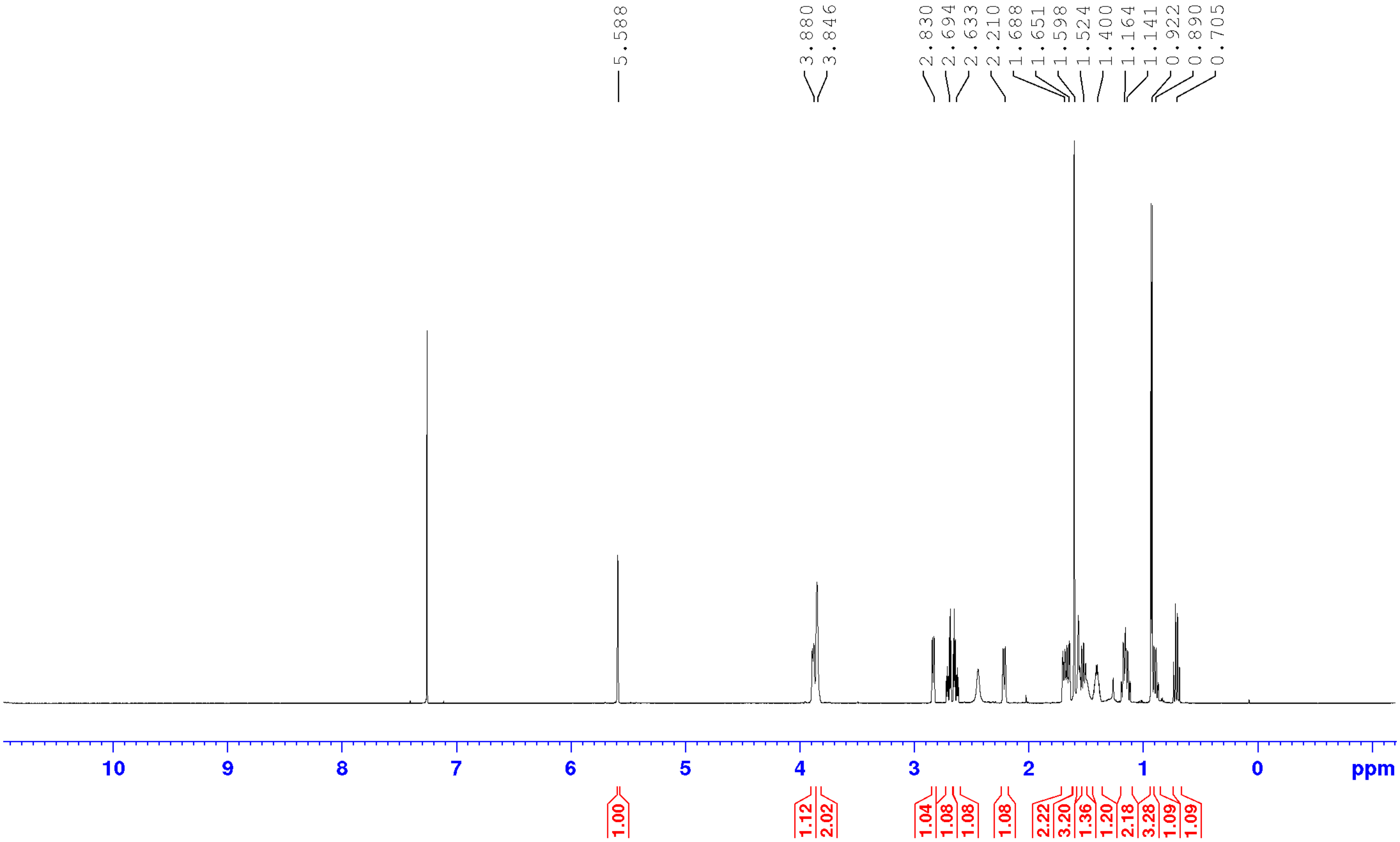

**Figure S3.**[13]**C NMR spectrum of 1 measured at 176 MHz in CDCl**$_3$

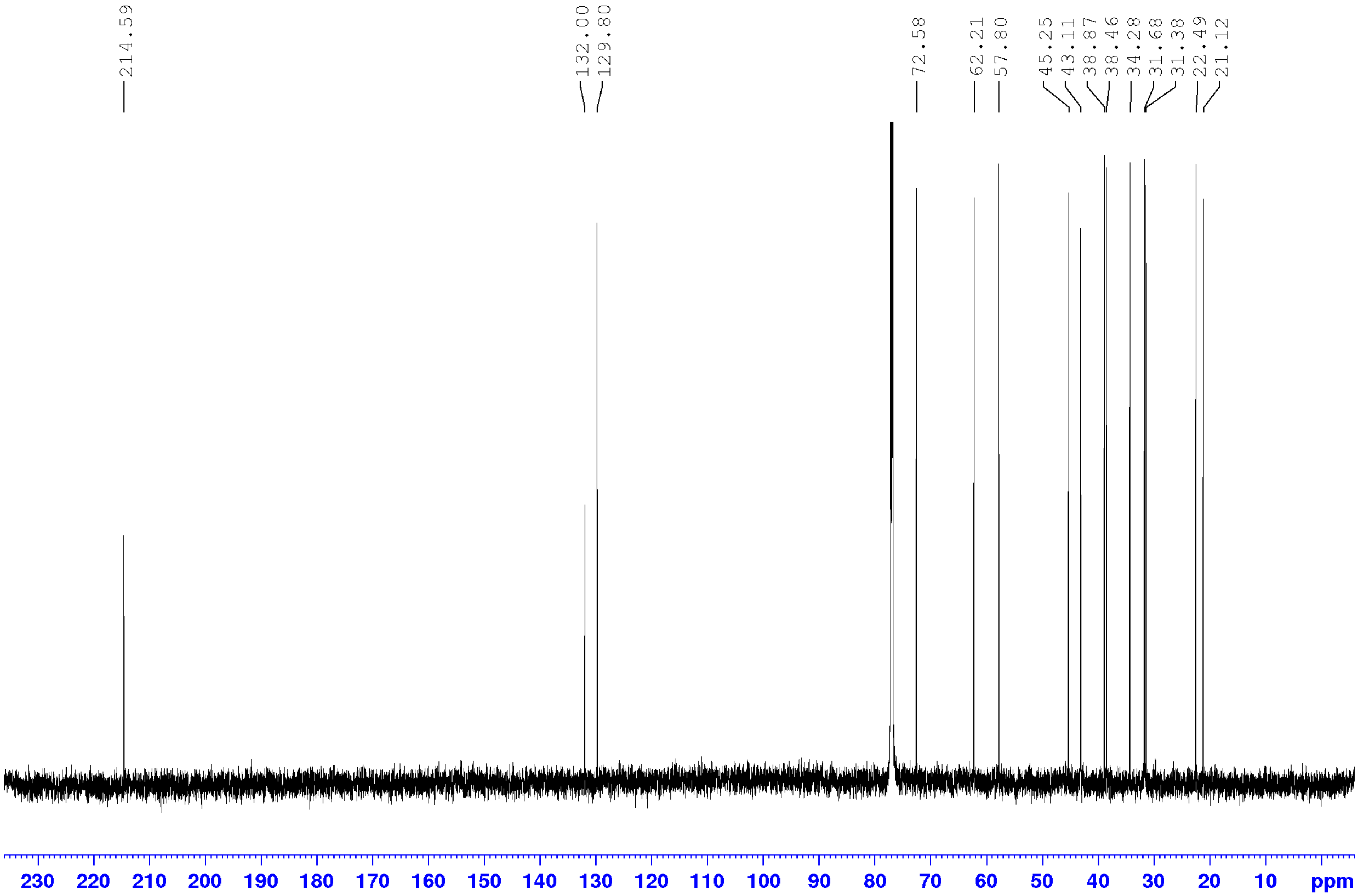

**Figure S4. DEPT-135 spectrum of 1 measured at 176 MHz in $CDCl_3$**

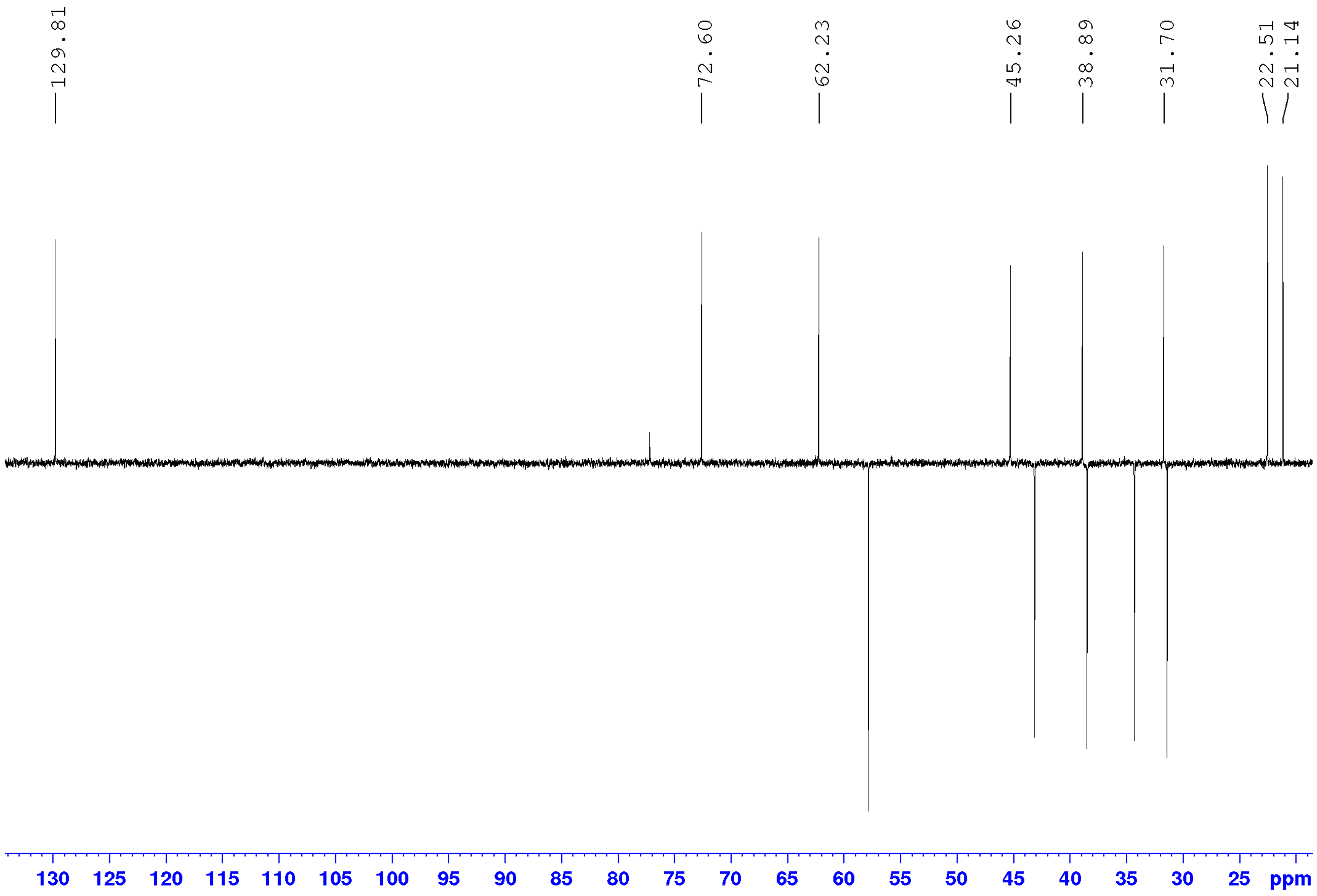

**Figure S5. HSQC spectrum of 1 measured in $CDCl_3$**

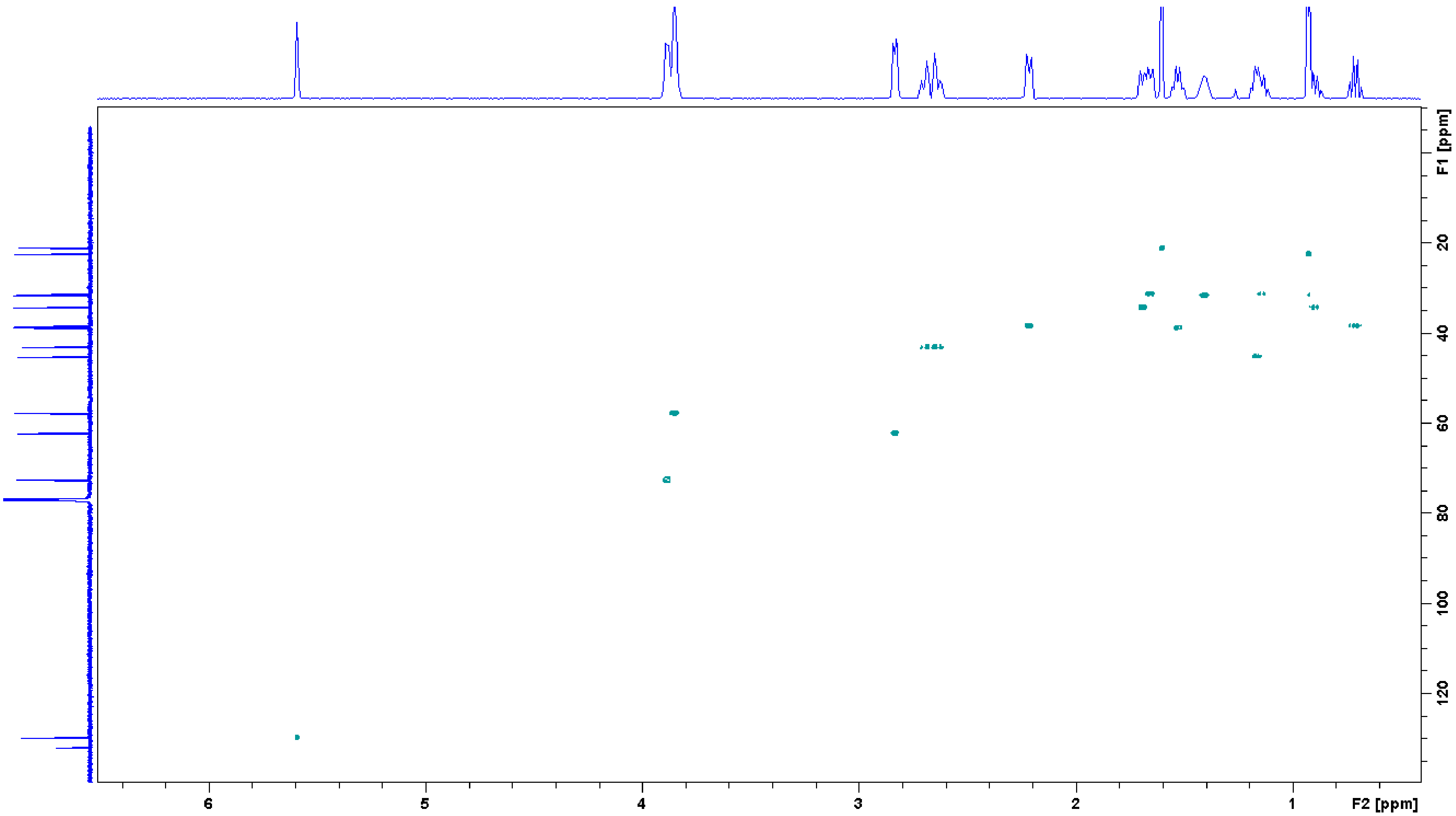

**Figure S6. COSY spectrum of 1 measured in $CDCl_3$**

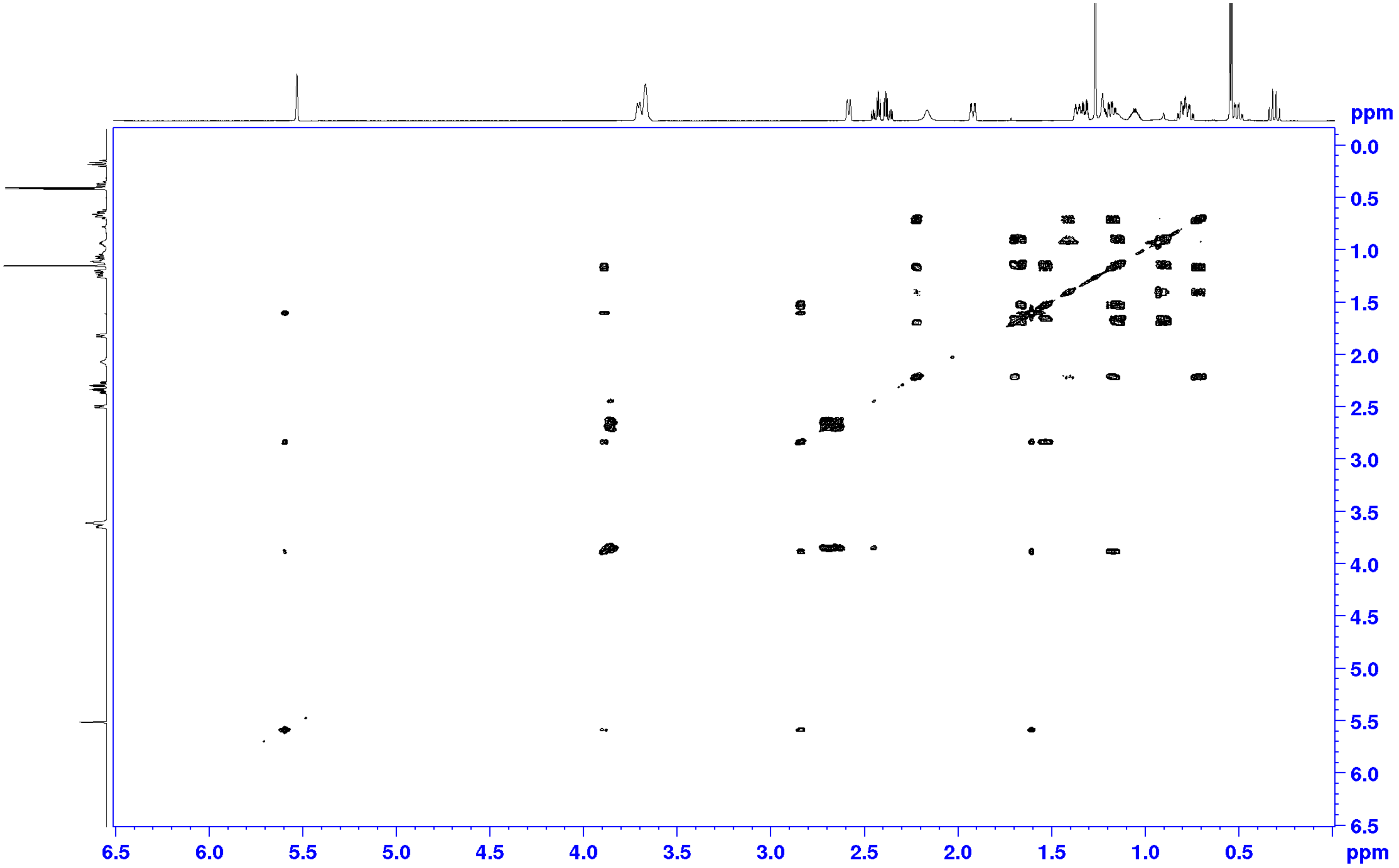

**Figure S7. HMBC spectrum of 1 measured in $CDCl_3$**

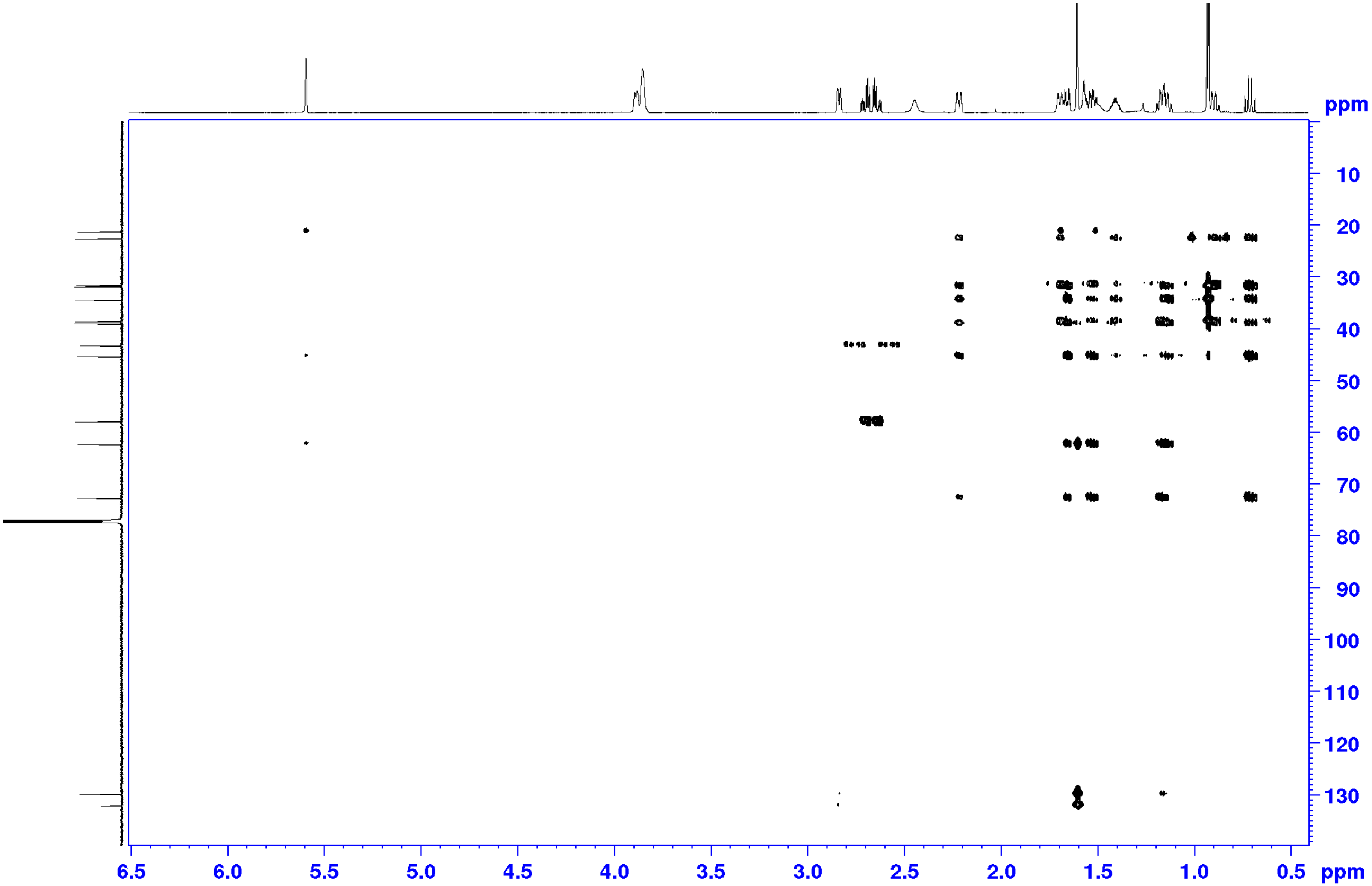

**Figure S8. ROESY spectrum of 1 measured in $CDCl_3$**

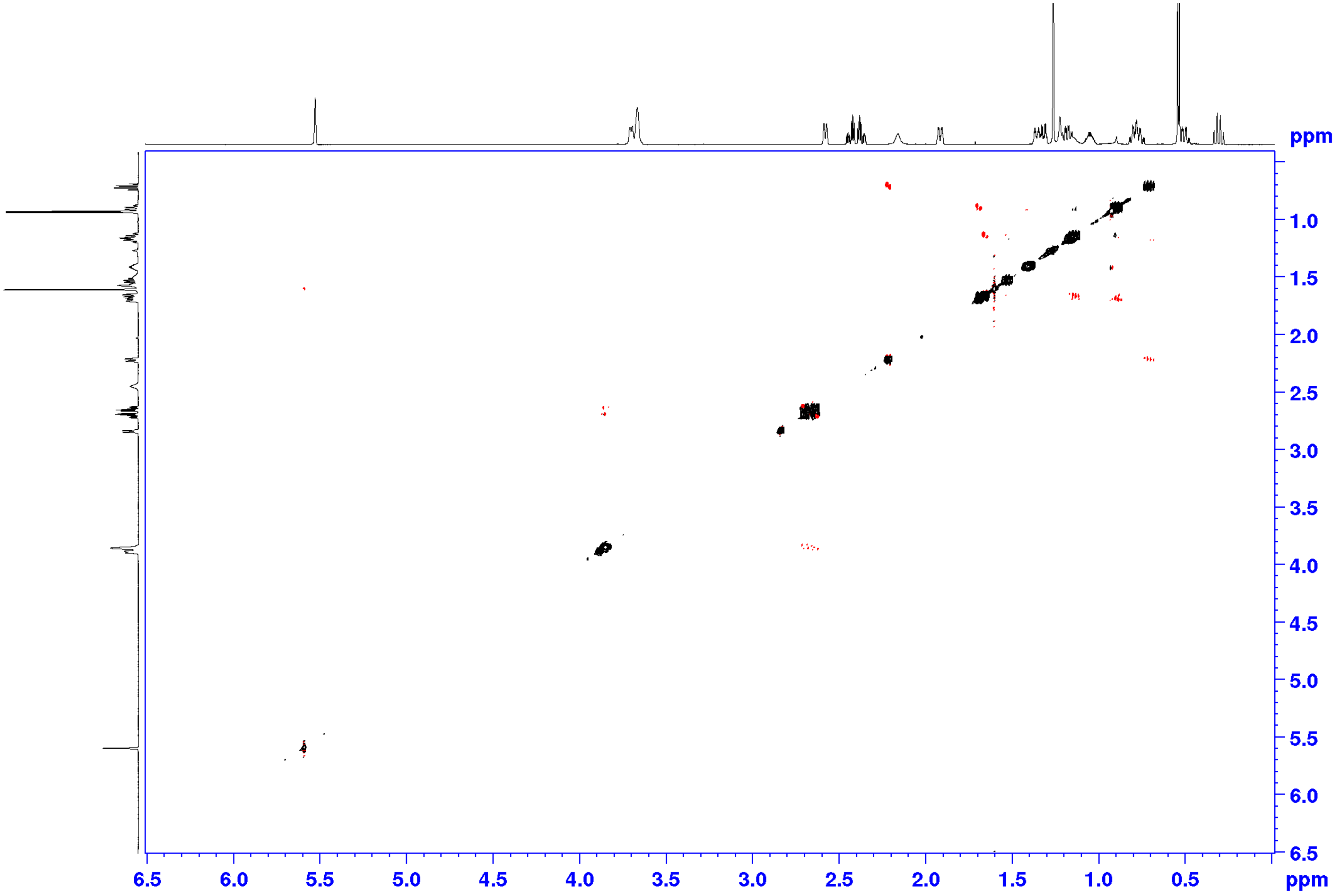

**Figure S9. HRESIMS for 2**

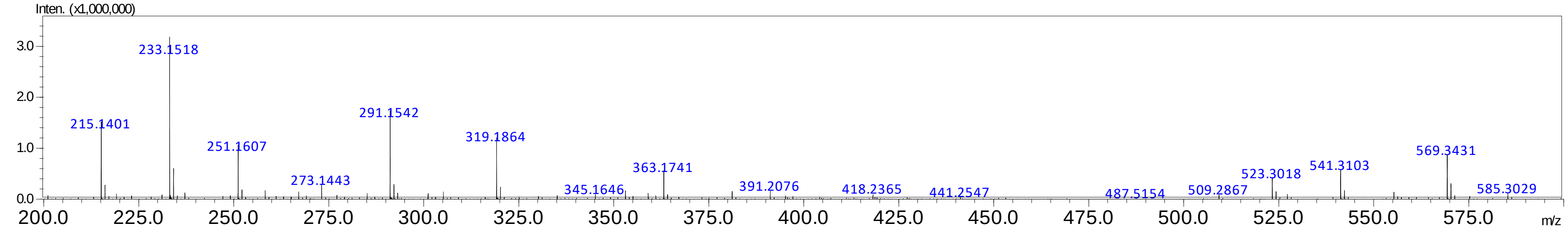


| | meas | calc | Δ (ppm) |
|---|---|---|---|
| $[M+Na]^+$ | 291,1542 | 291,1566 | -8,5 |

**Figure S10.** $^1H$ **NMR spectrum of 2 measured at 700 MHz in** $CDCl_3$

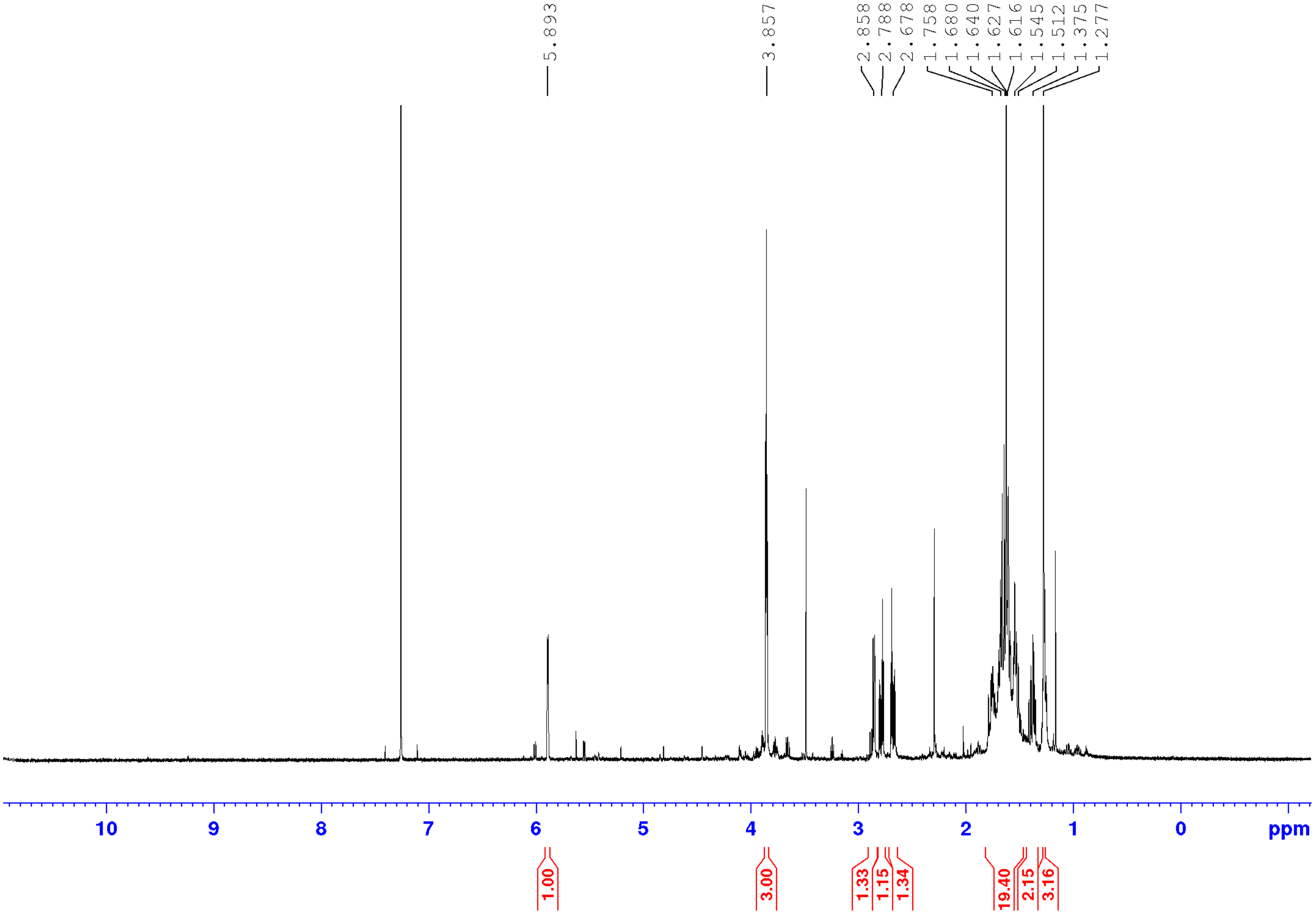

**Figure S11.** $^{13}C$ **NMR spectrum of 2 measured at 176 MHz in** $CDCl_3$

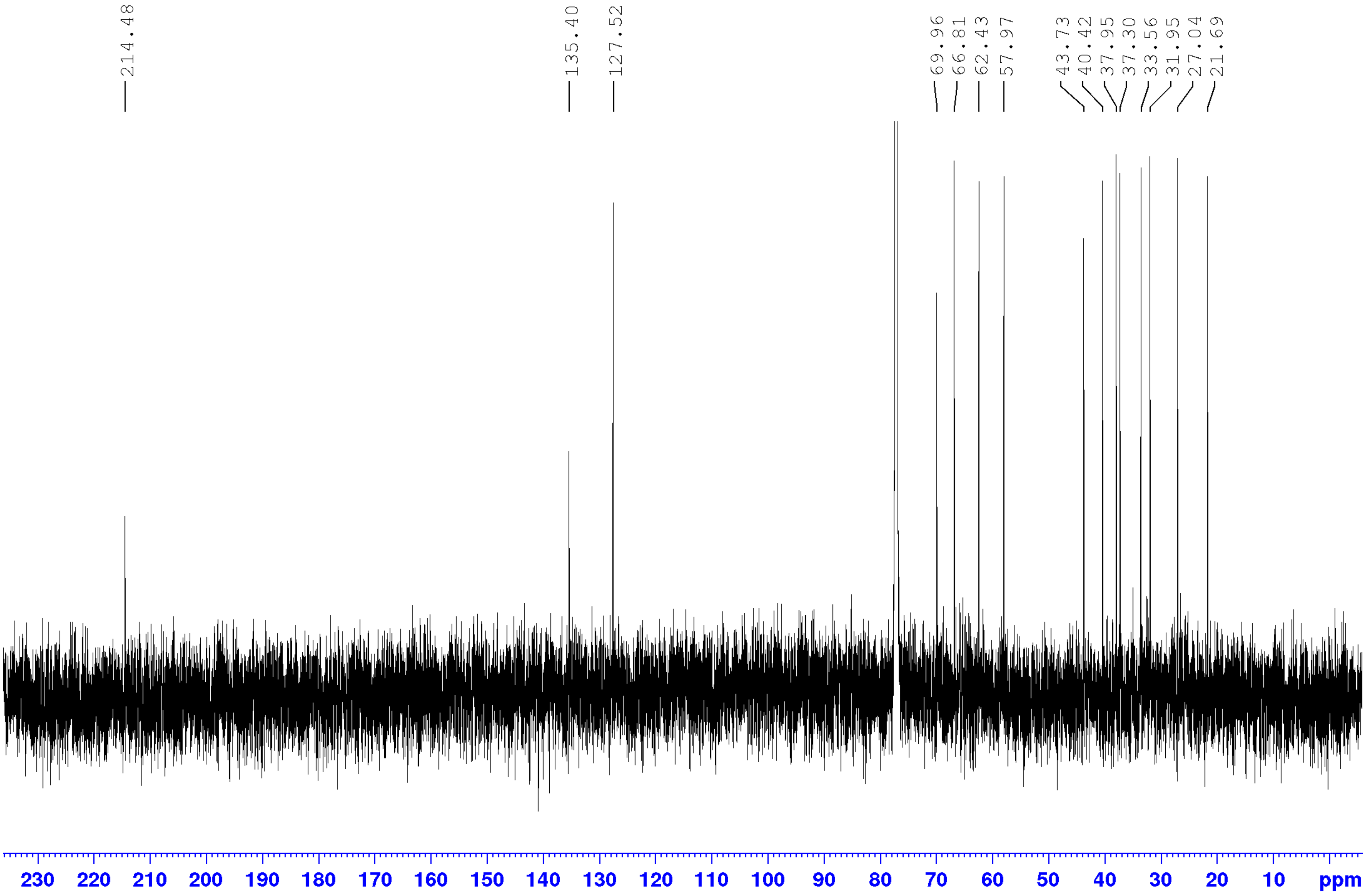

**Figure S12. DEPT-135 spectrum of 2 measured at 176 MHz in $CDCl_3$**

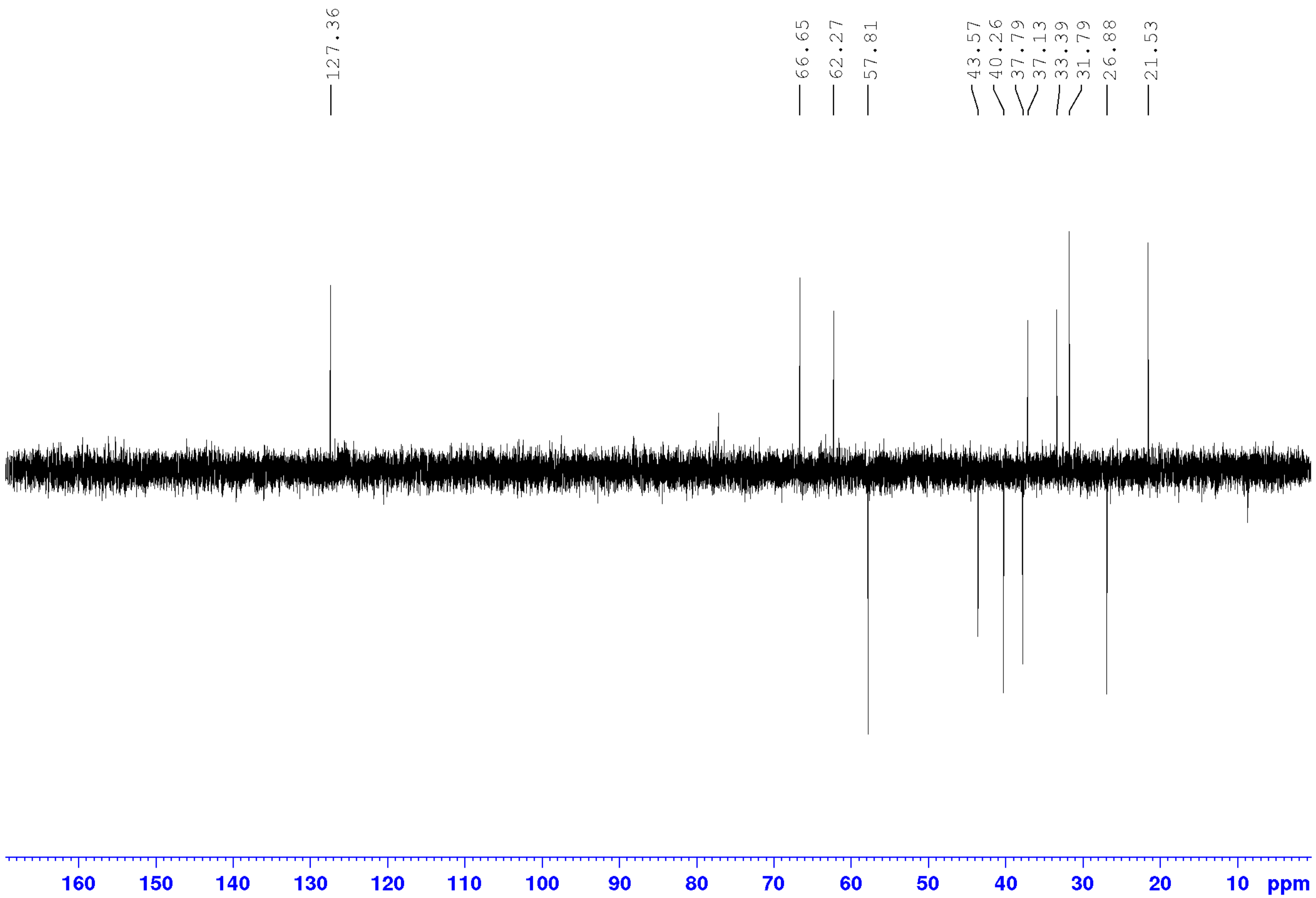

**Figure S13. HSQC spectrum of 2 measured in $CDCl_3$**

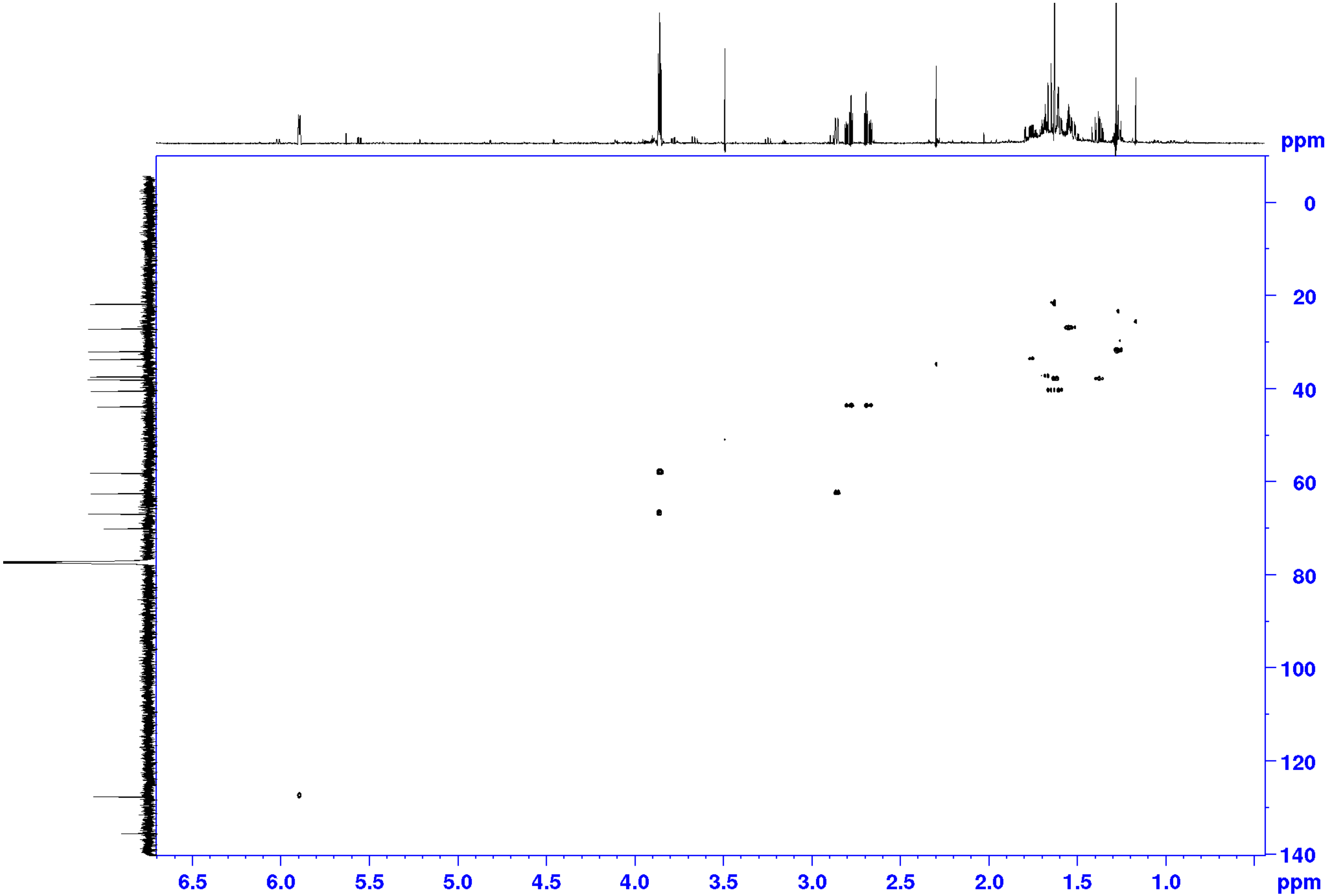

**Figure S14. COSY spectrum of 2 measured in $CDCl_3$**

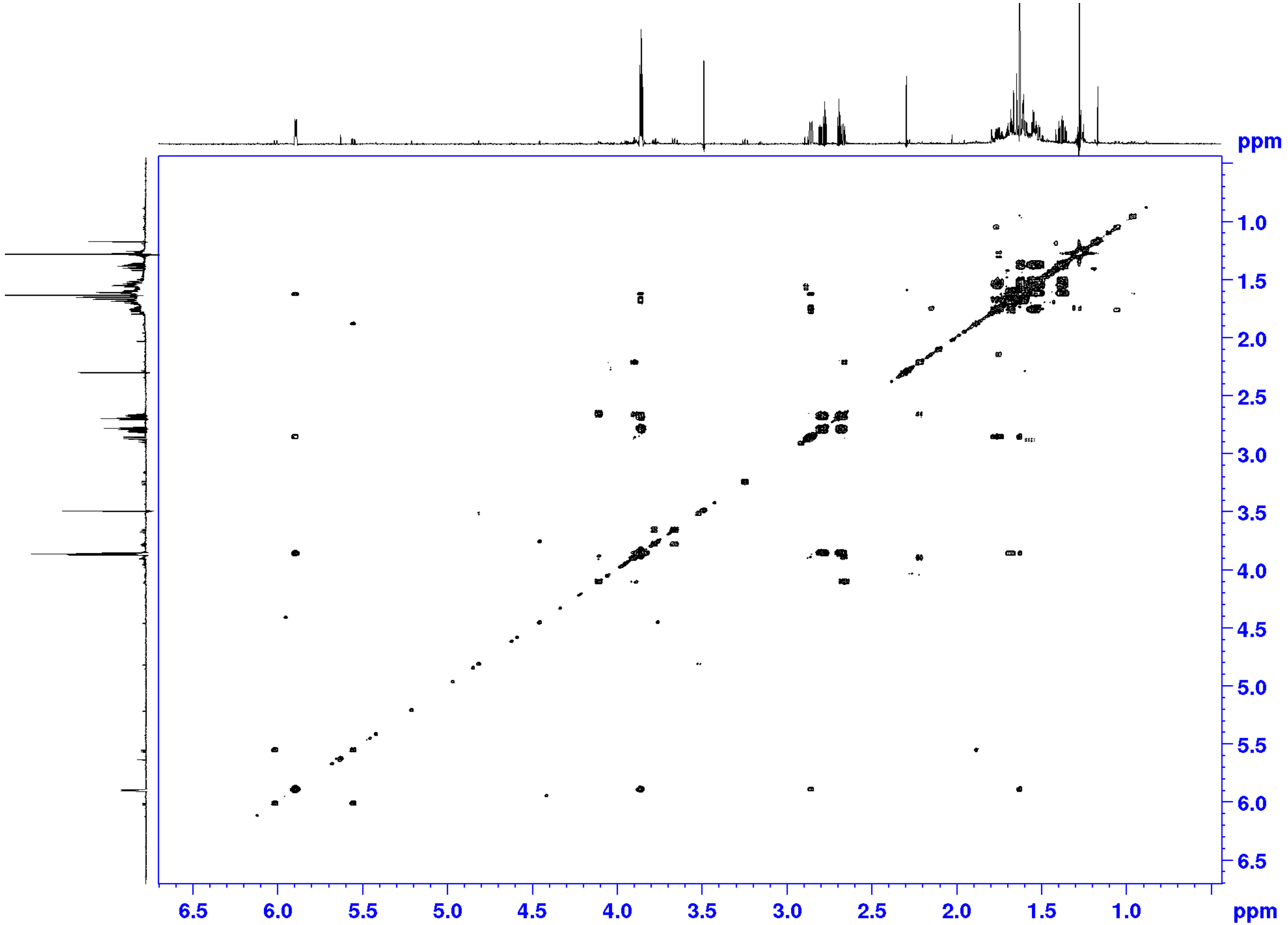

**Figure S15. HMBC spectrum of 2 measured in CDCl$_3$**

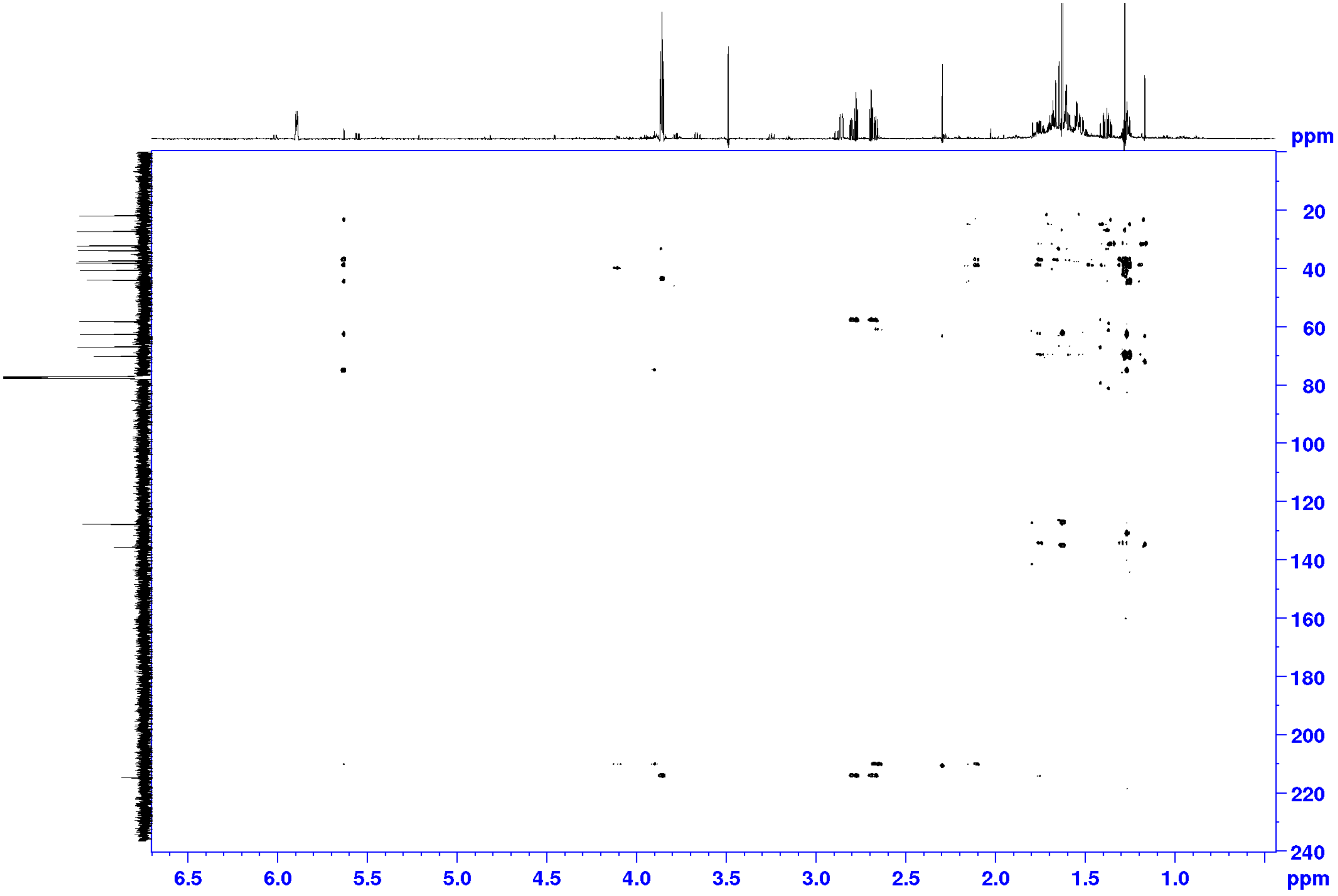

**Figure S16. ROESY spectrum of 2 measured in $CDCl_3$**

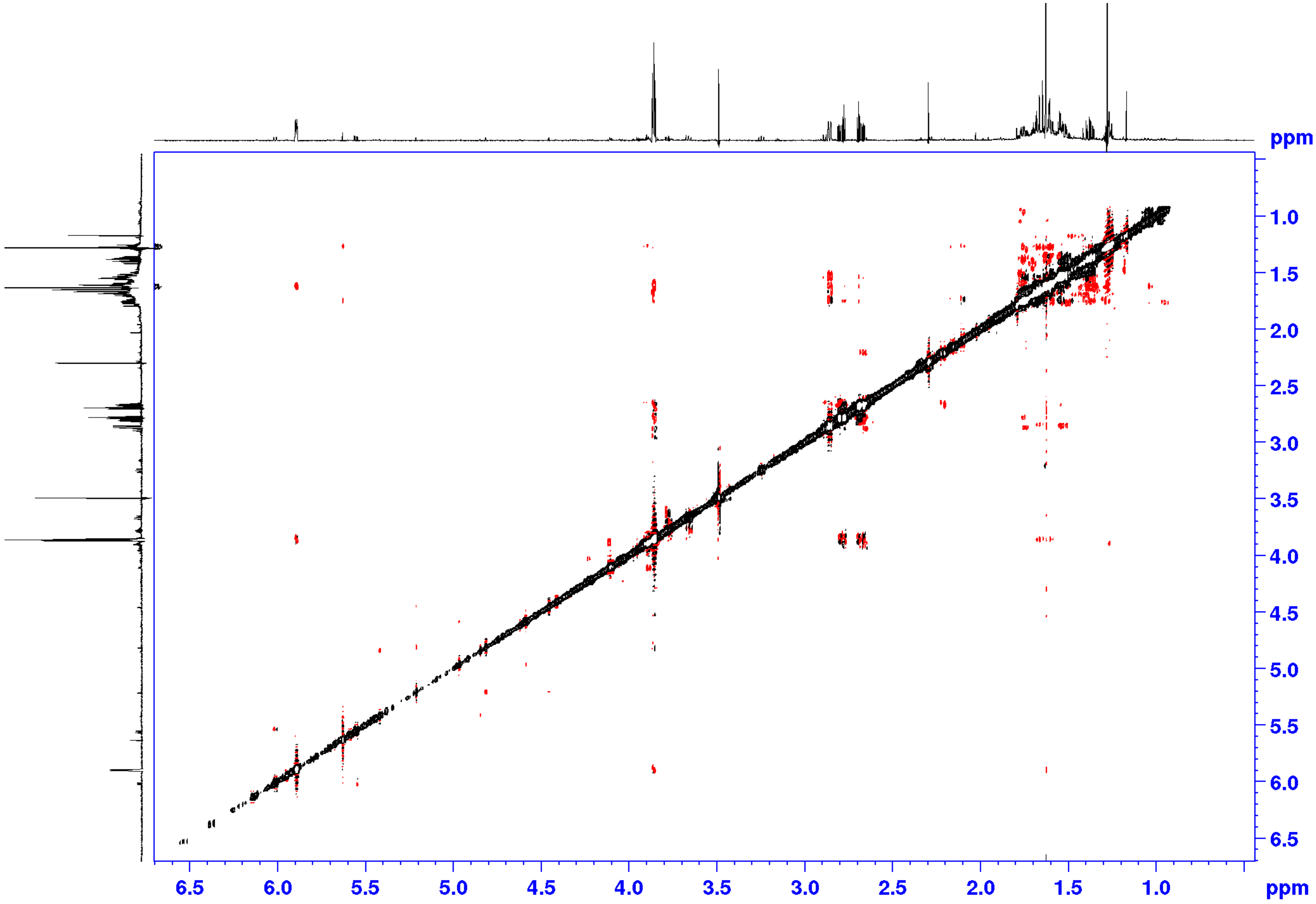

**Figure S17. HRESIMS for 3**

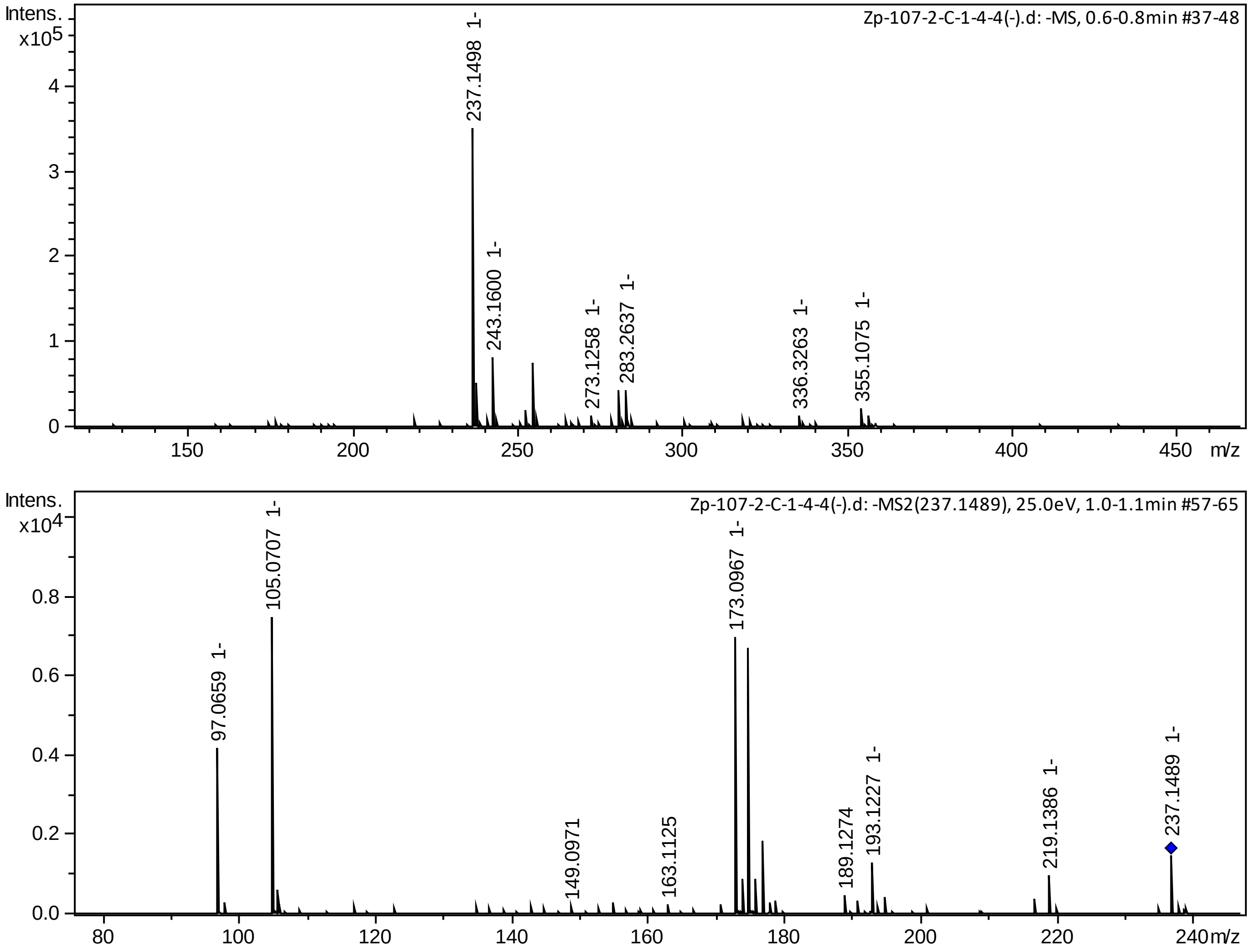

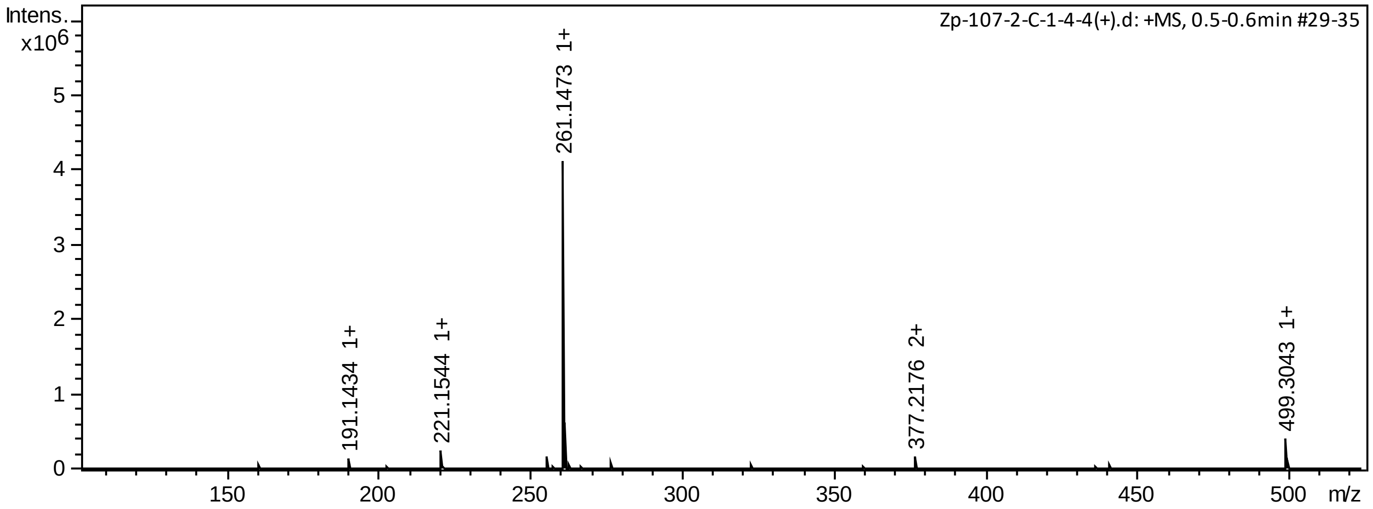


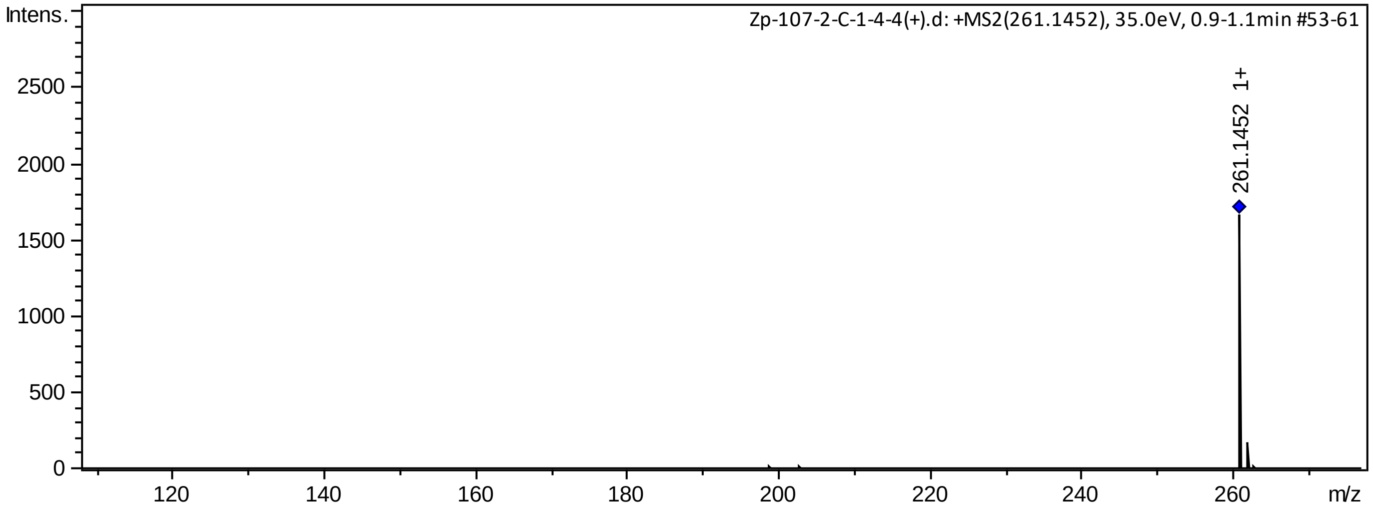


| | meas | calc | Δ (ppm) |
|---|---|---|---|
| $[M-H]^-$ | 237,1498 | 237,1496 | -0,8 |
| $[M+Na]^+$ | 261,1473 | 261,1461 | -4,6 |

**Figure S18. ¹H NMR spectrum of 3 measured at 700 MHz in $CDCl_3$**

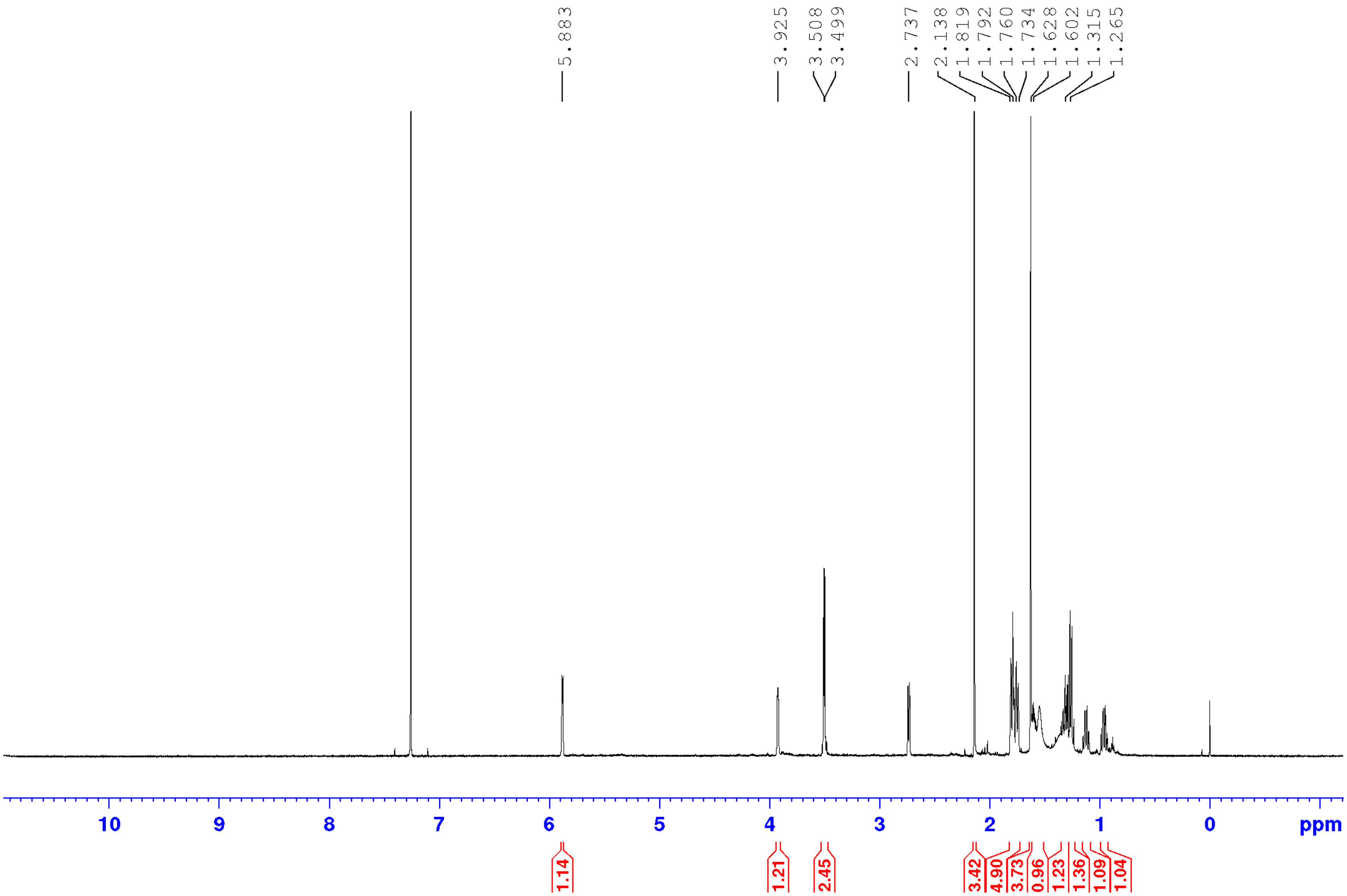

**Figure S19.** $^{13}C$ **NMR spectrum of 3 measured at 176 MHz in** $CDCl_3$

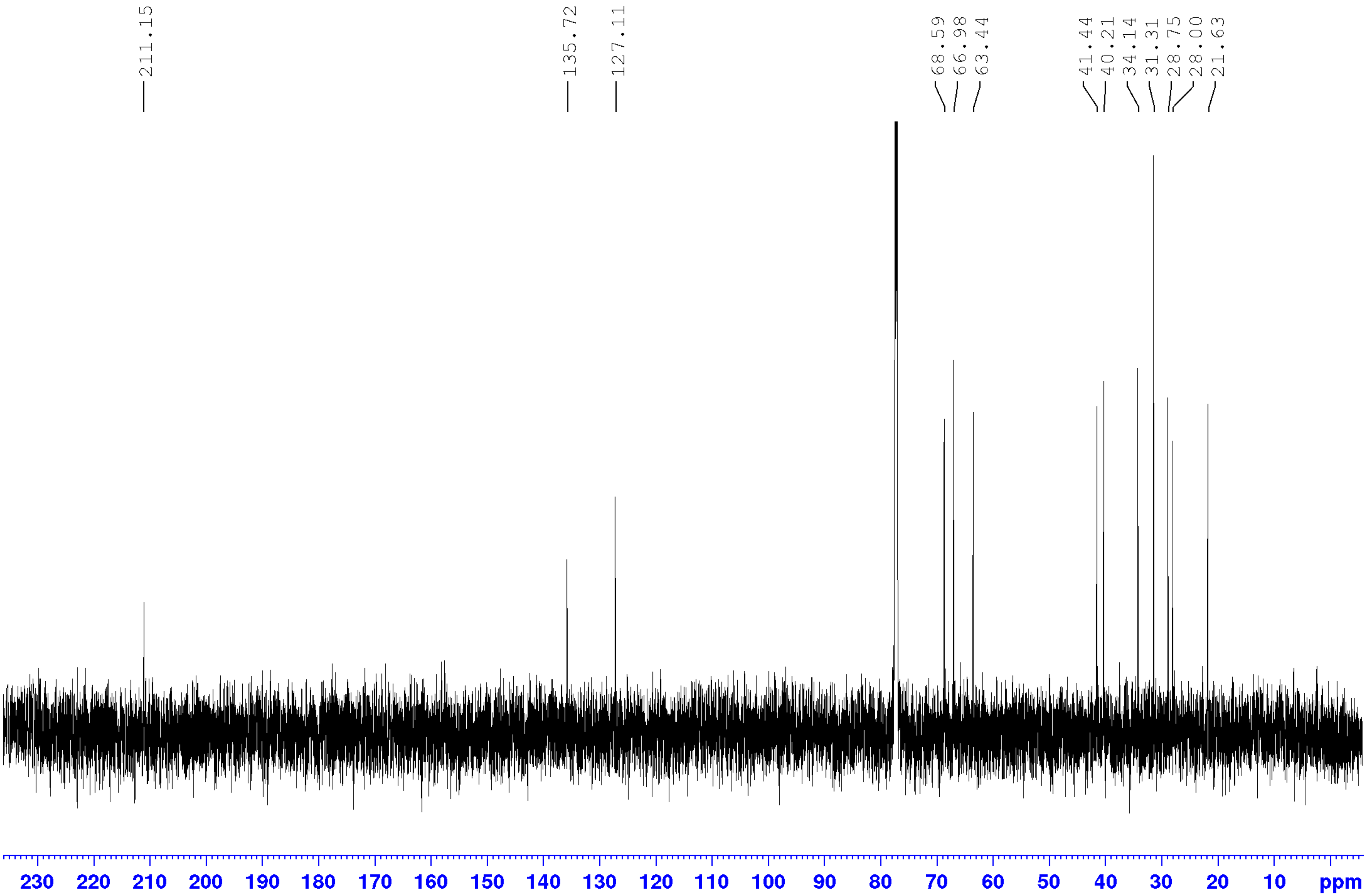

**Figure S20. DEPT-135 spectrum of 3 measured at 176 MHz in CDCl₃**

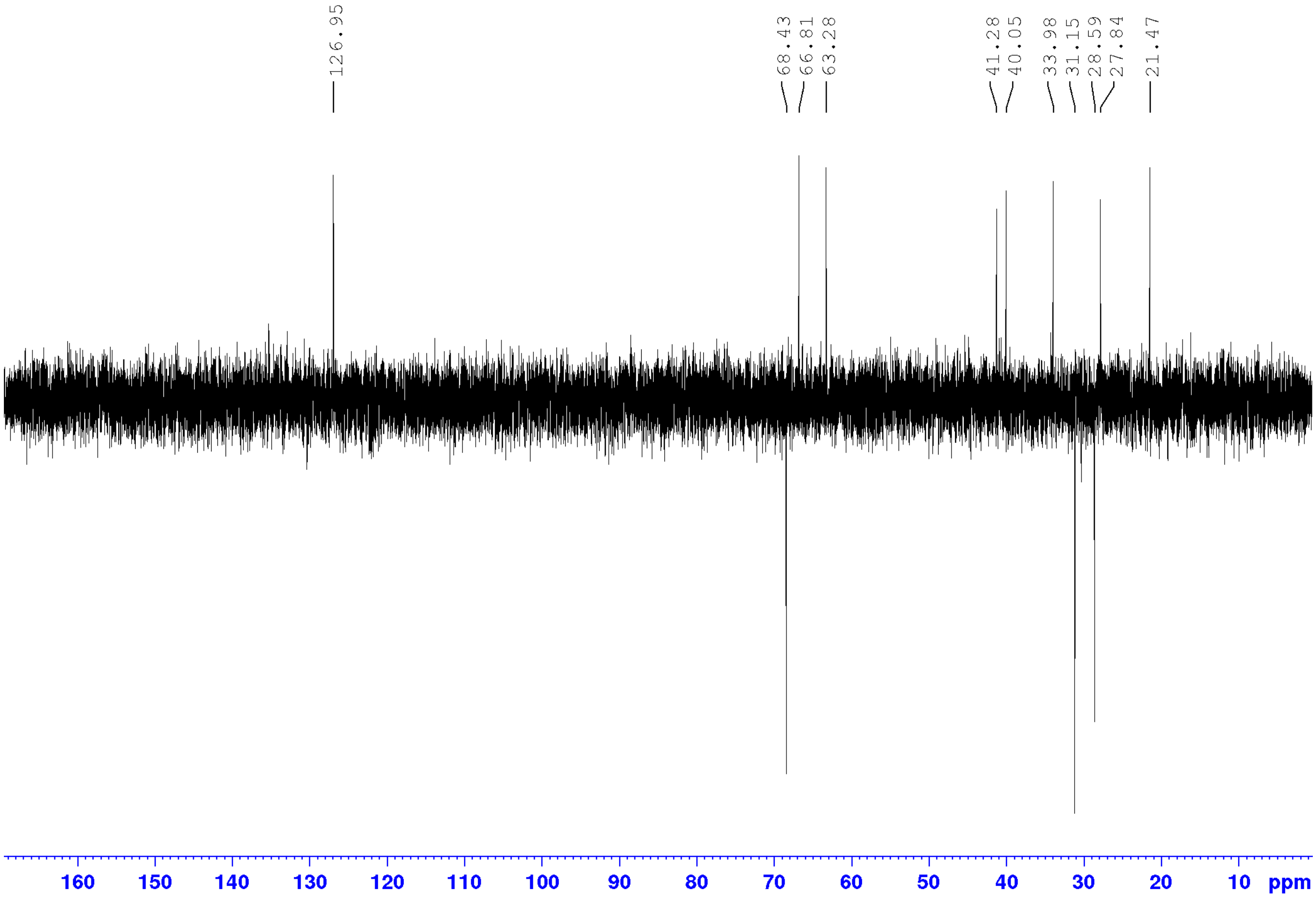

**Figure S21. HSQC spectrum of 3 measured in $CDCl_3$**

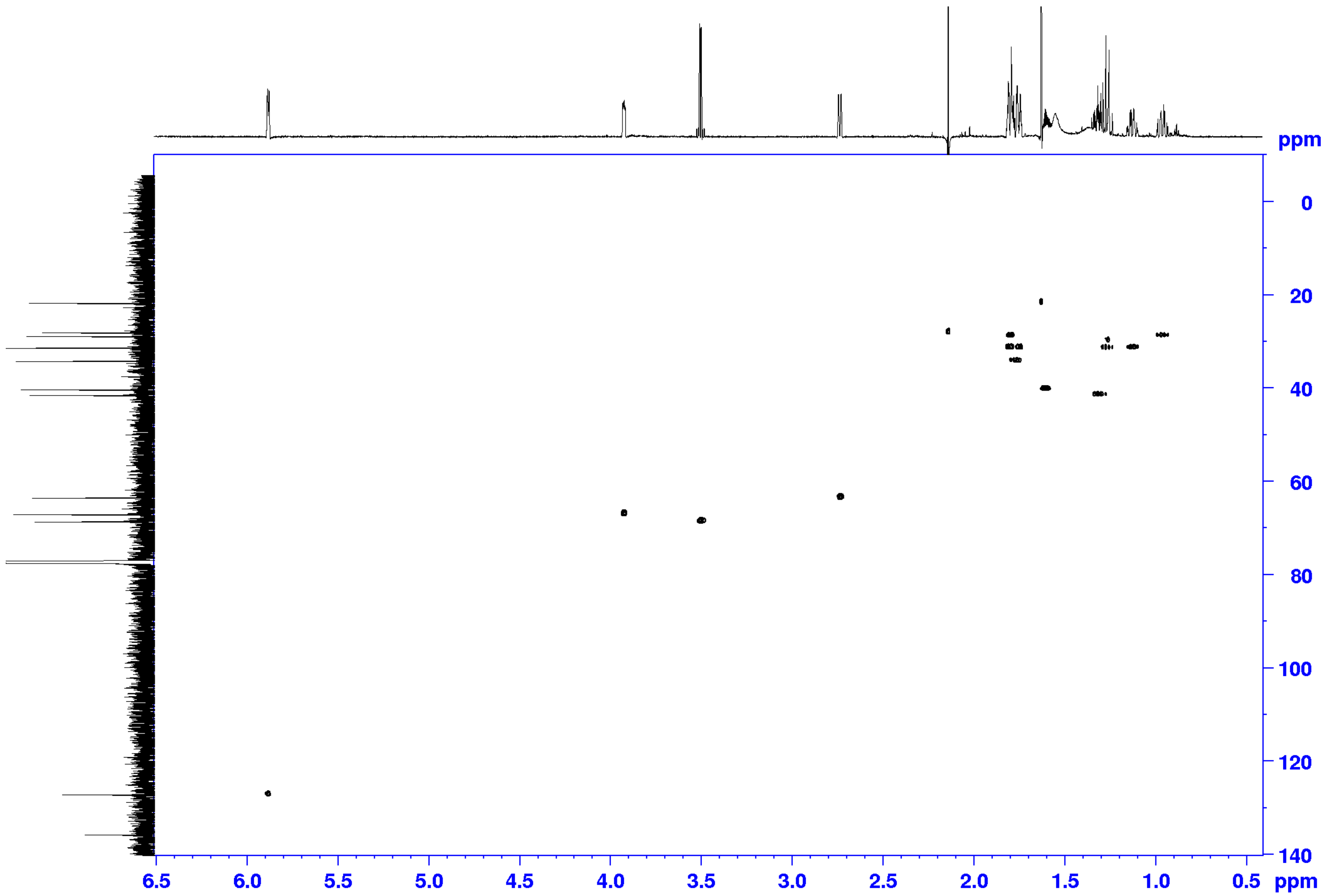

**Figure S22. COSY spectrum of 3 measured in $CDCl_3$**

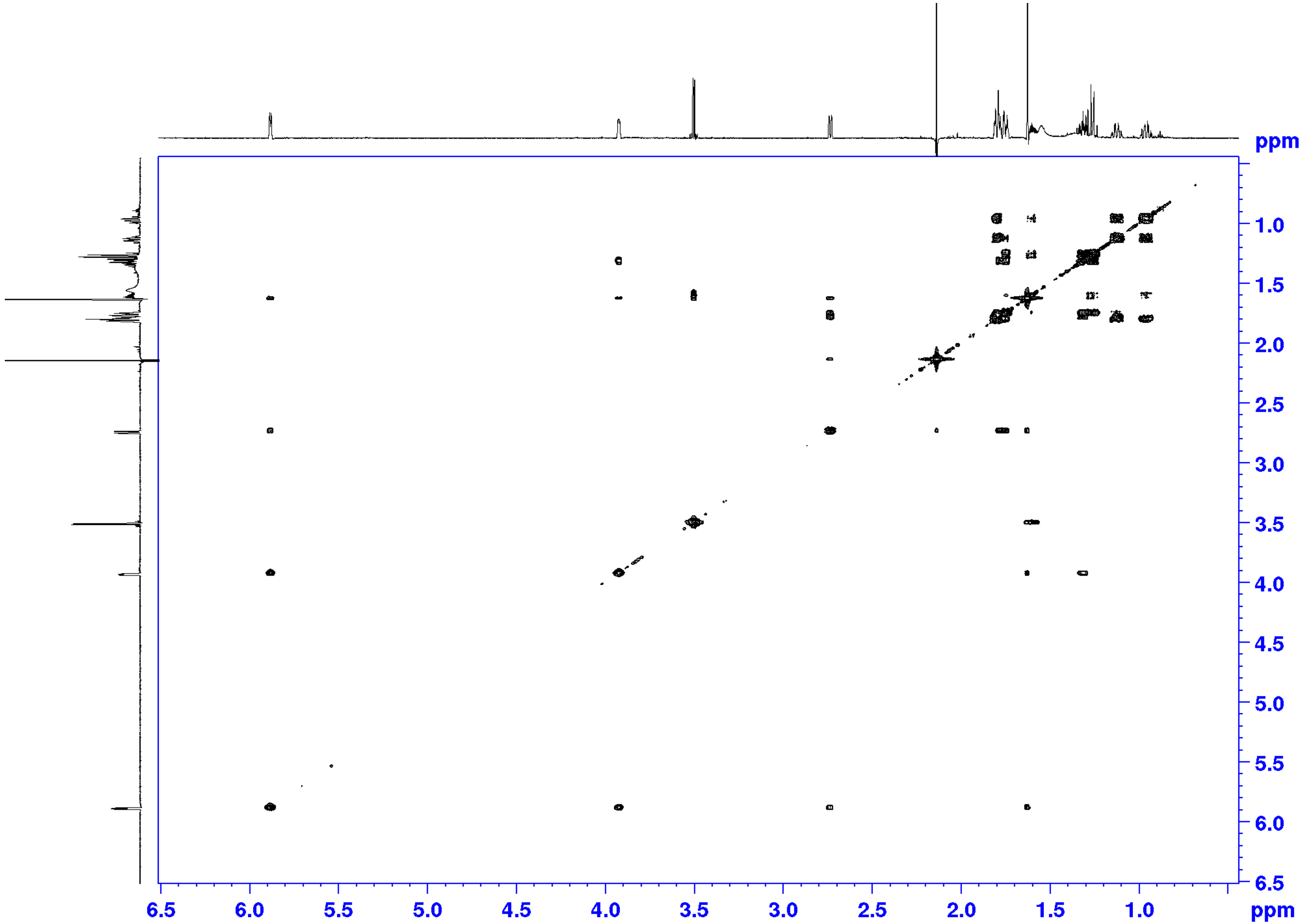

**Figure S23. HMBC spectrum of 3 measured in CDCl$_3$**

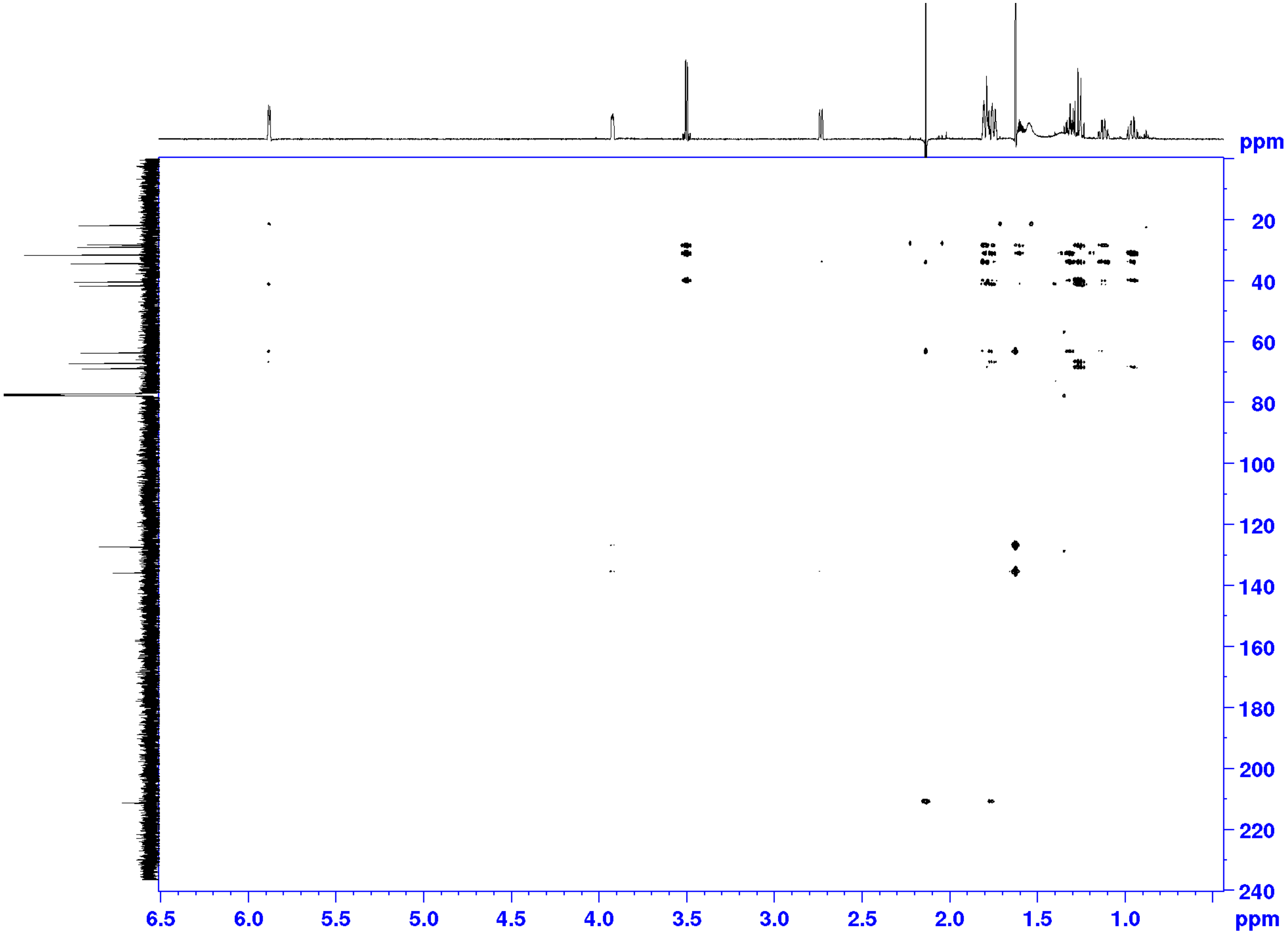

**Figure S24. ROESY spectrum of 3 measured in $CDCl_3$**

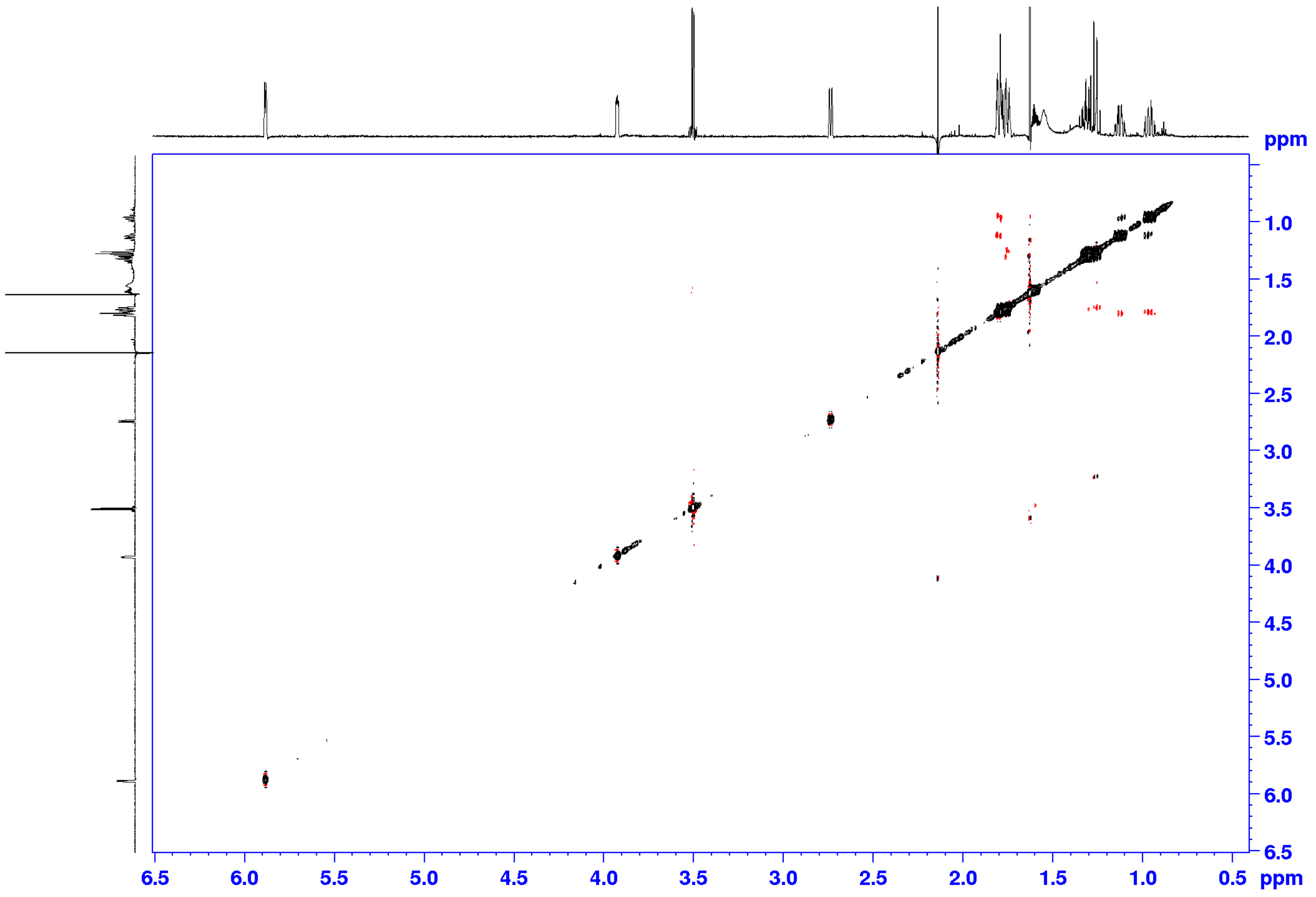

**Figure S25. HRESIMS for 4**

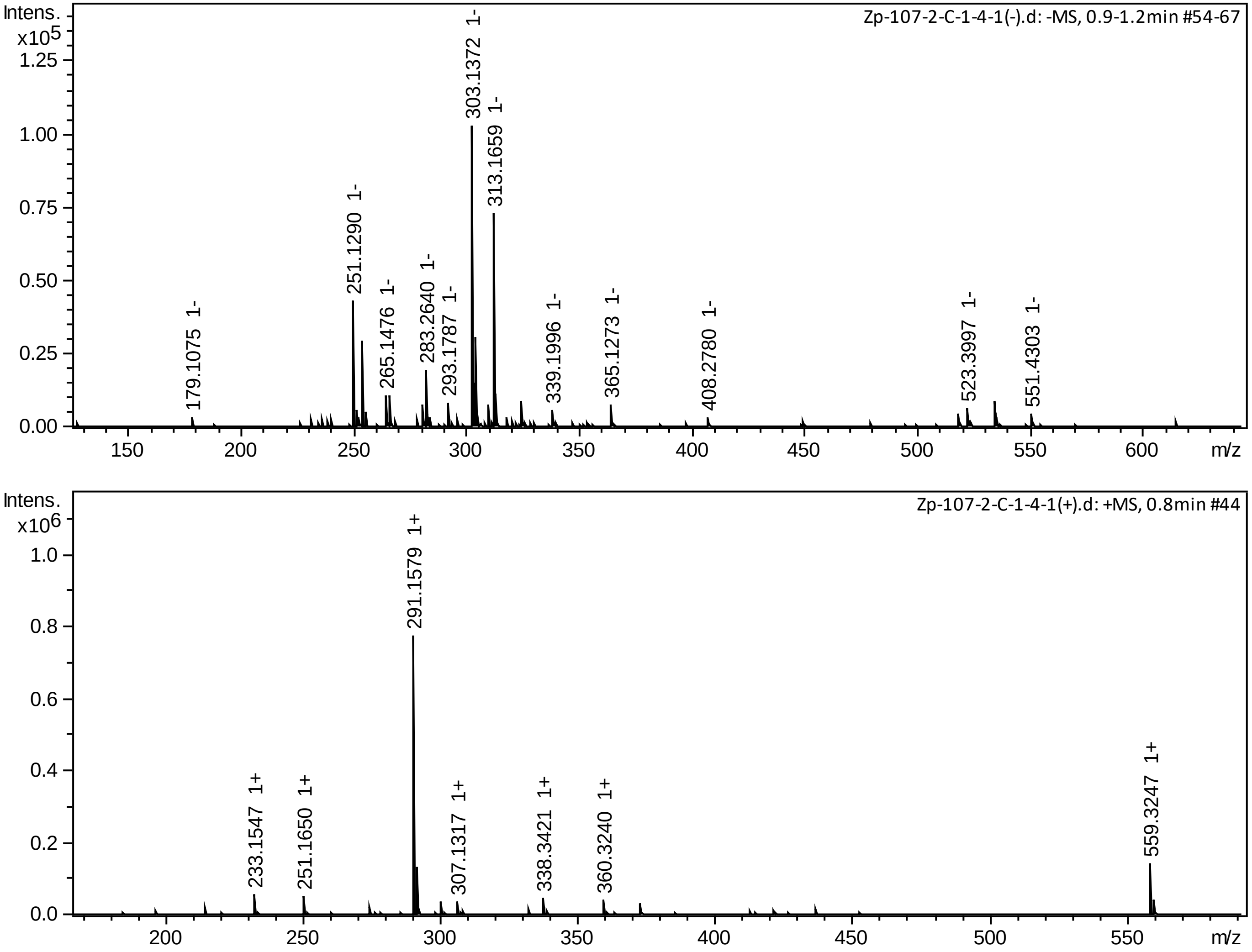

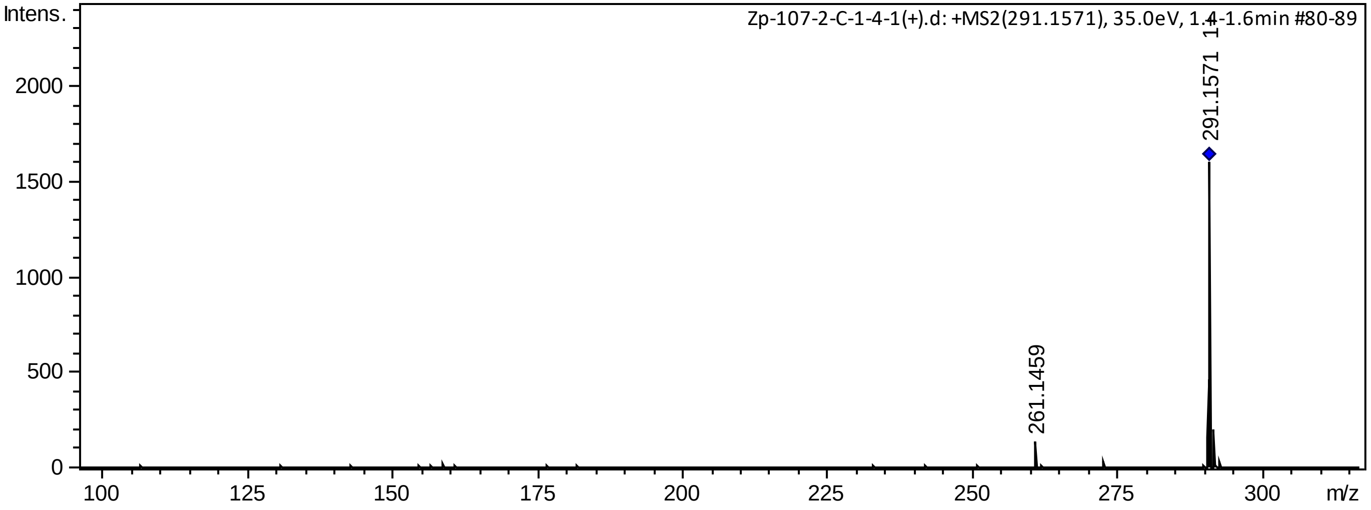


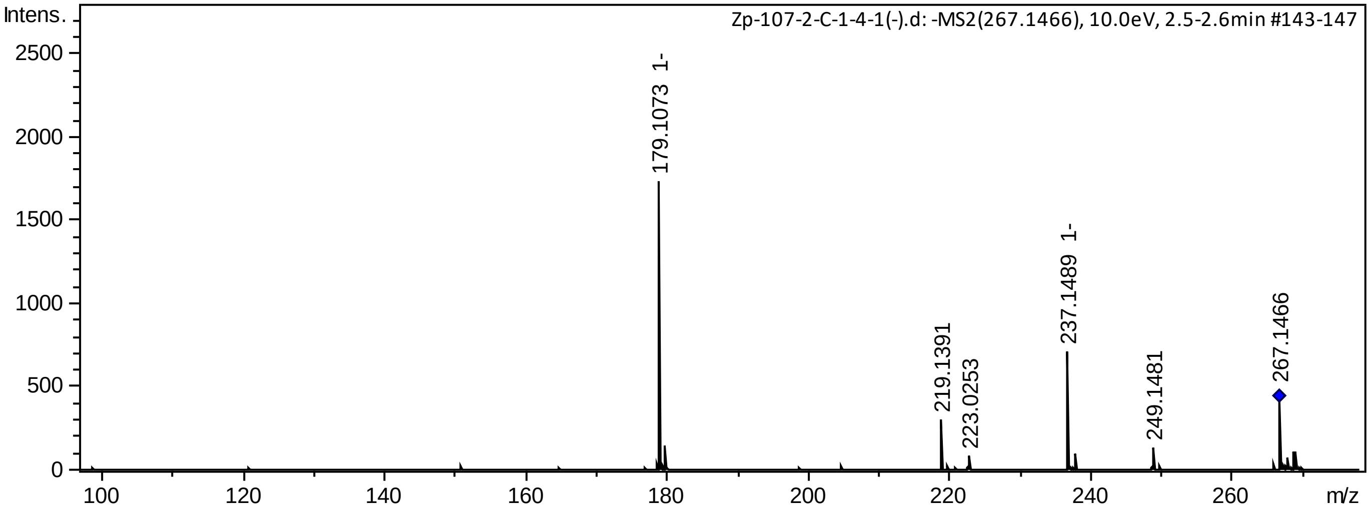


| | meas | calc | Δ (ppm) |
|---|---|---|---|
| $[M-H]^-$ | 267,1598 | 267,1602 | 1,5 |
| $[M+Cl]^-$ | 303,1372 | 303,1369 | -1,0 |
| $[M+Na]^+$ | 291,1579 | 291,1567 | -4,1 |

**Figure S26. $^1$H NMR spectrum of 4 measured at 500 MHz in $CDCl_3$**

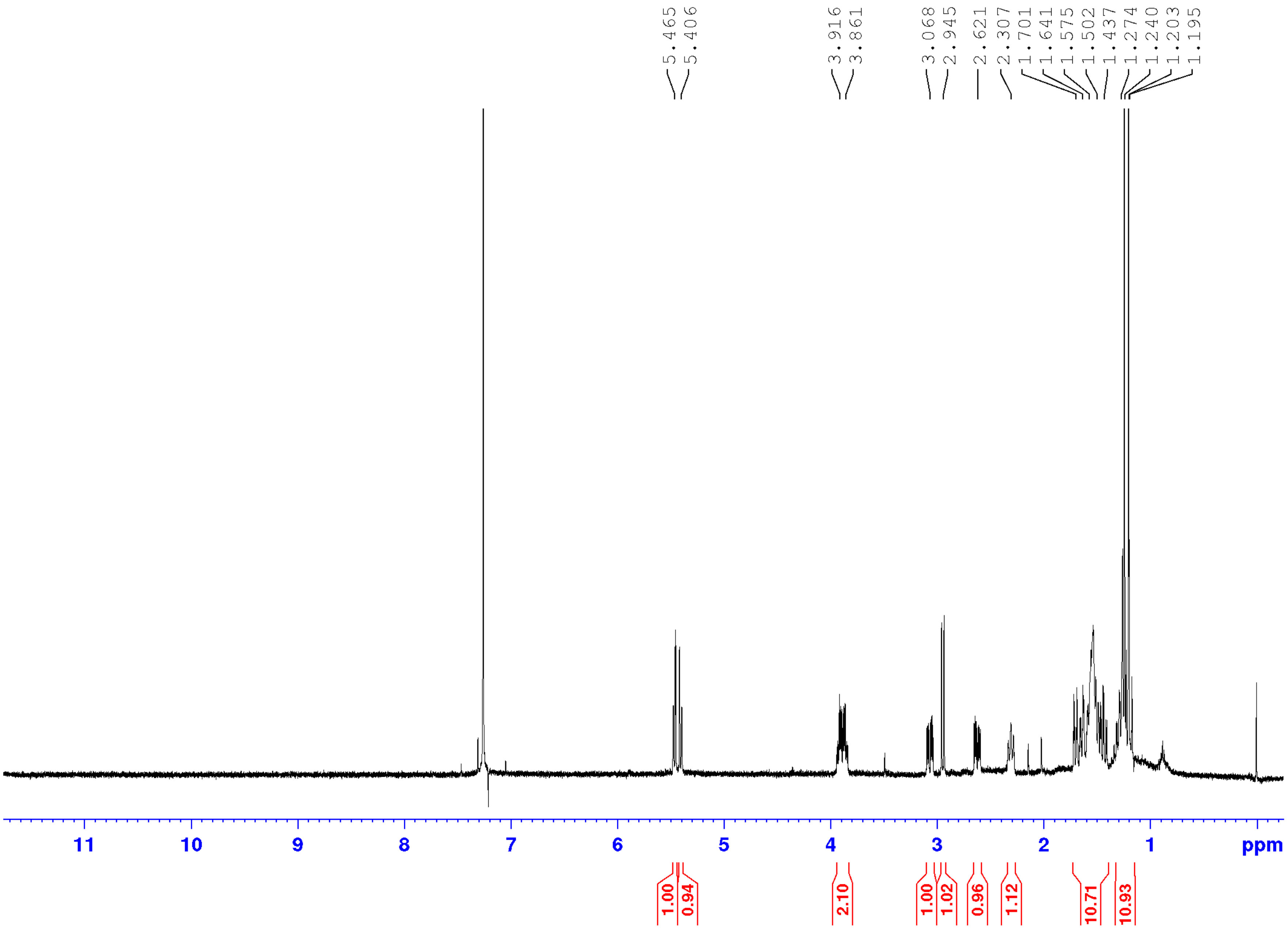

**Figure S27.**[13]**C NMR spectrum of 4 measured at 125 MHz in** $CDCl_3$

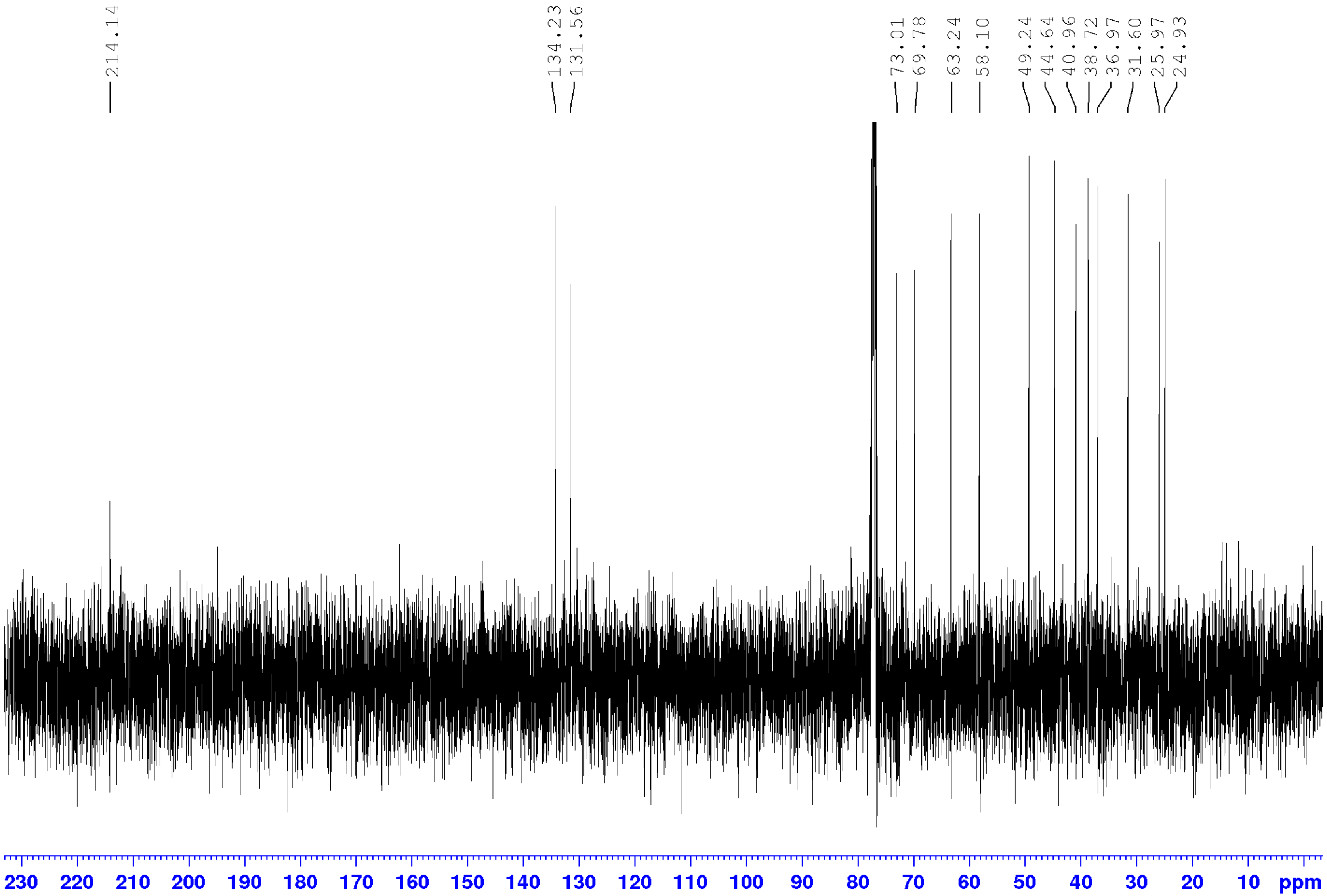

**Figure S28. HSQC spectrum of 4 measured in CDCl₃**

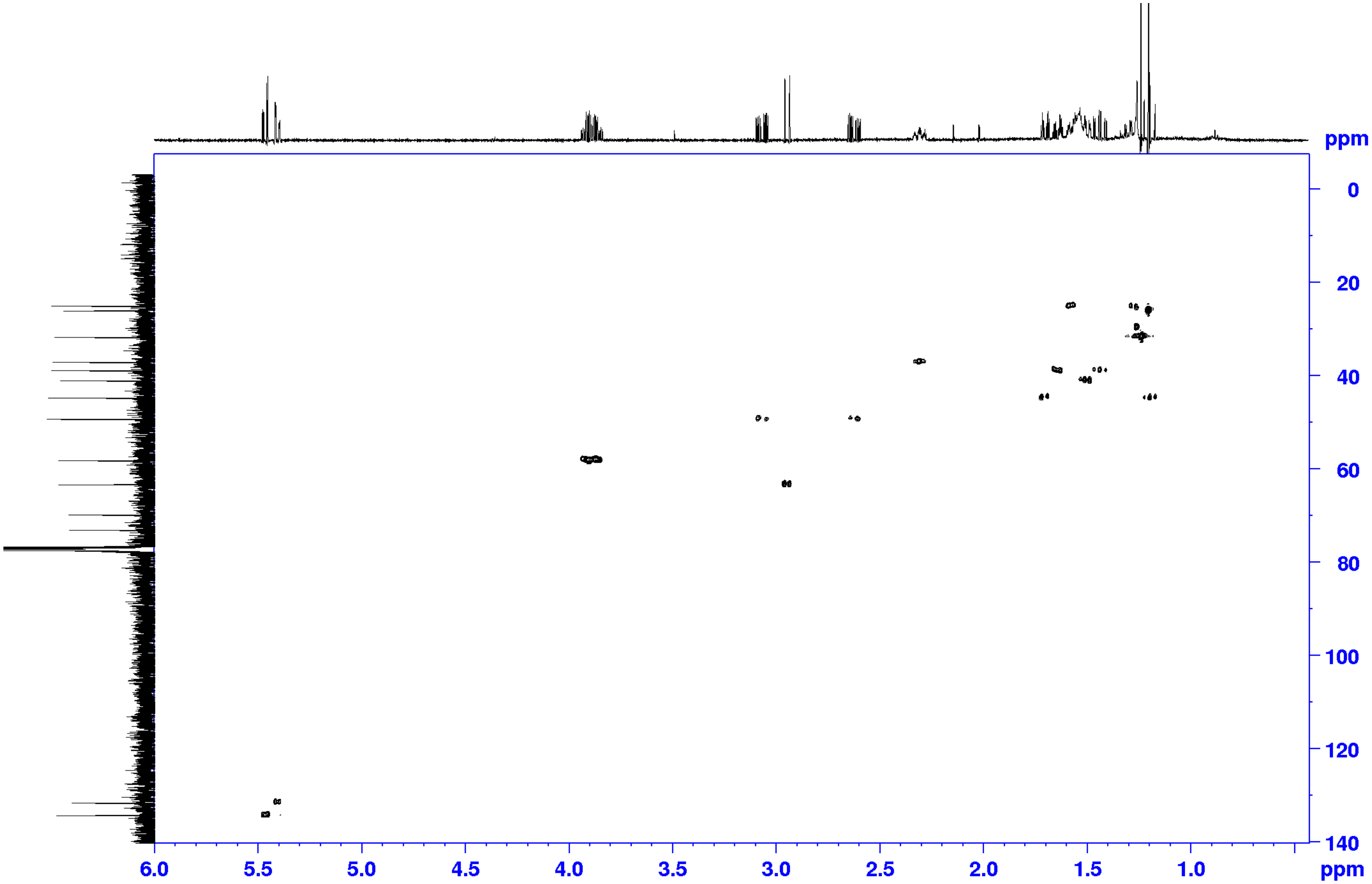

**Figure S29. COSY spectrum of 4 measured in $CDCl_3$**

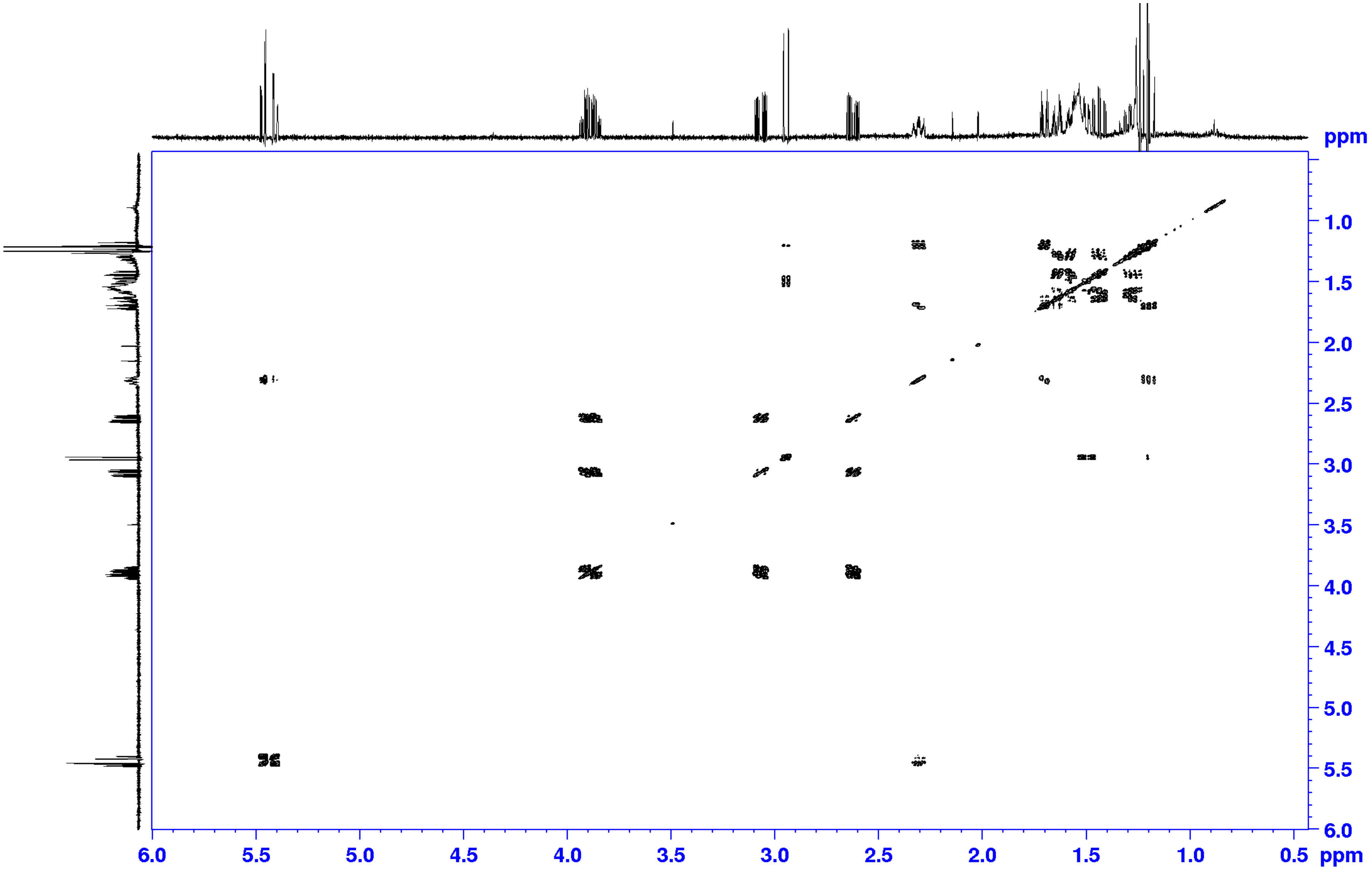

**Figure S30. HMBC spectrum of 4 measured in $CDCl_3$**

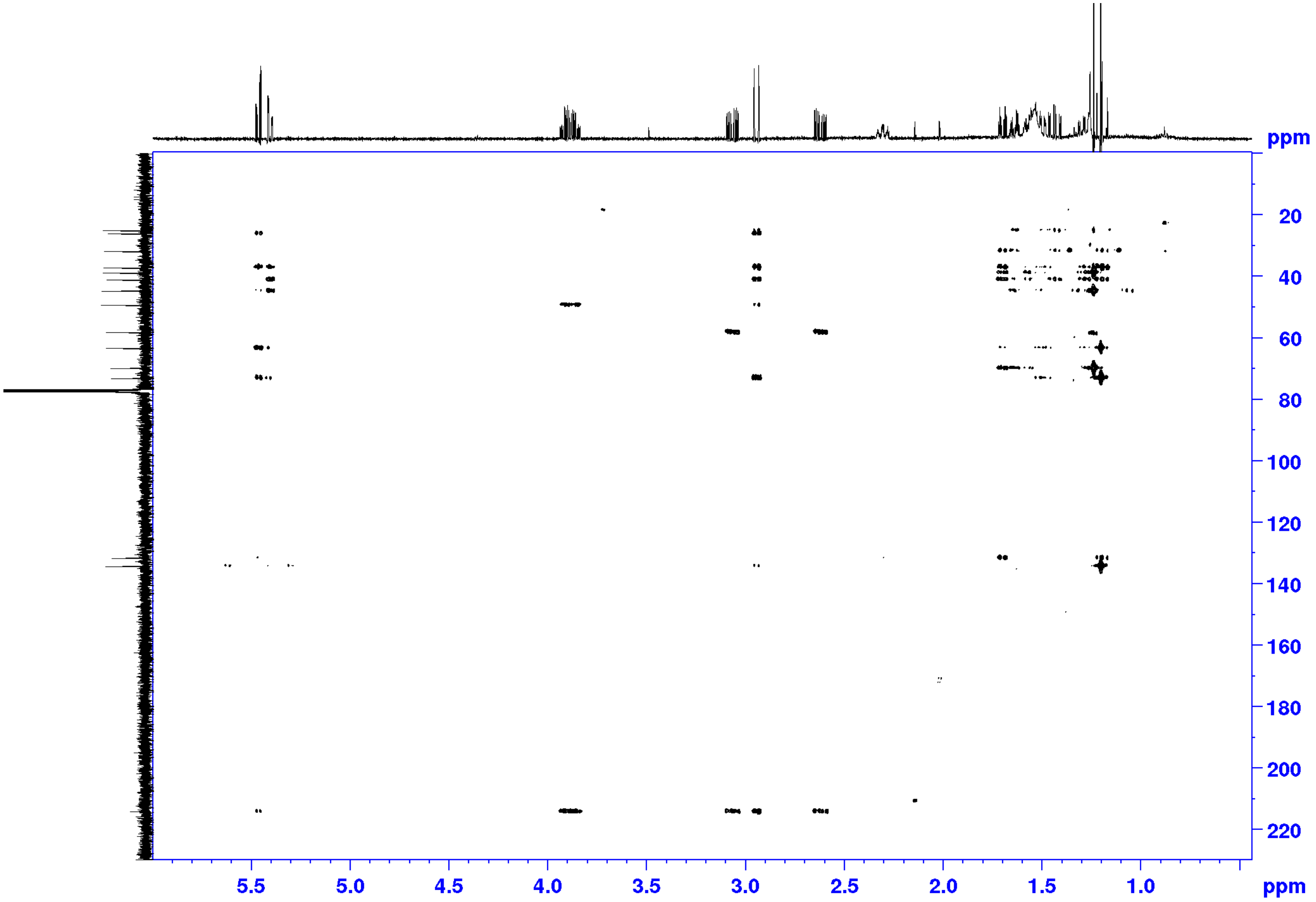

**Figure S31. ROESY spectrum of 4 measured in $CDCl_3$**

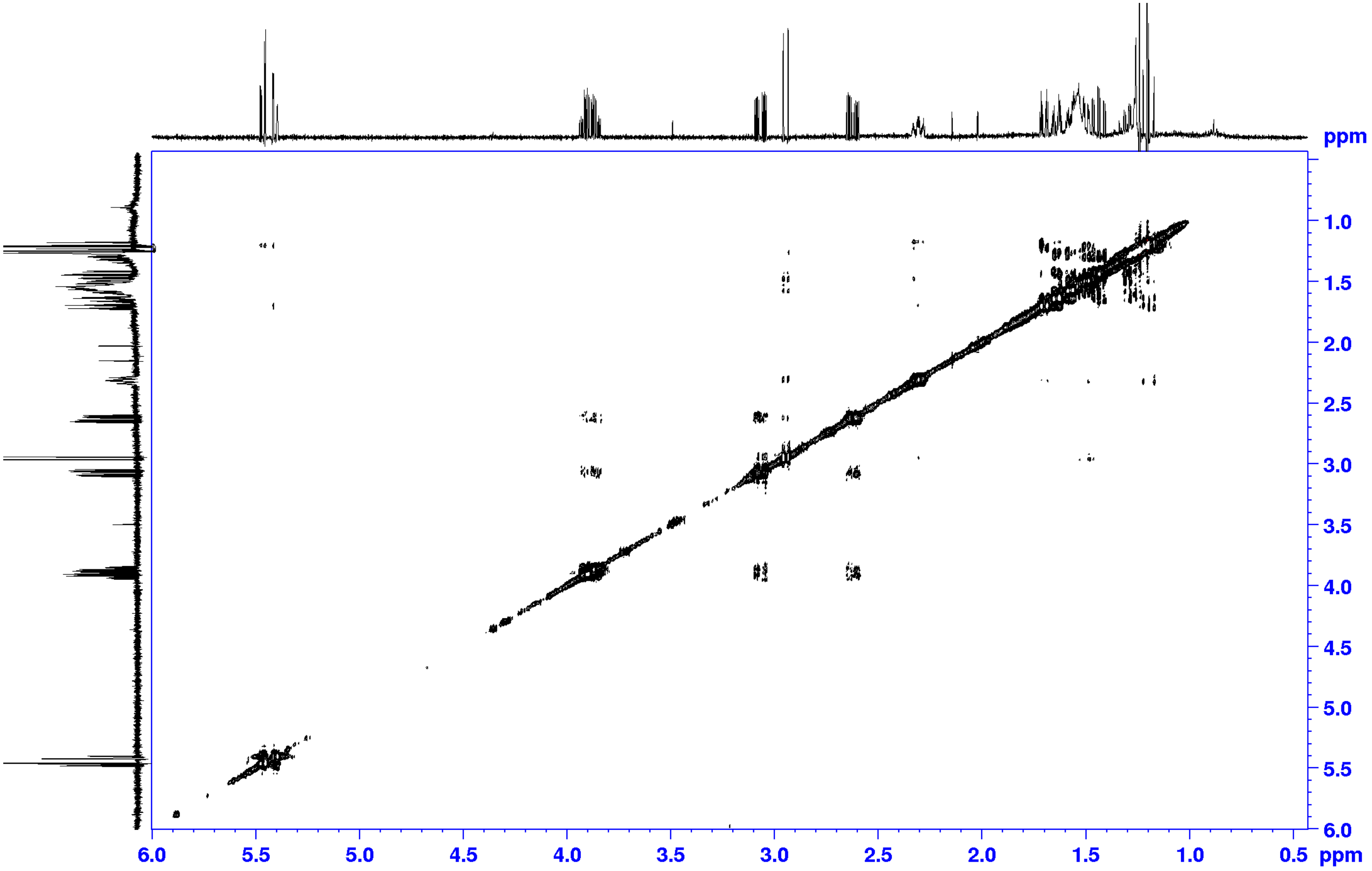

**Figure S32. HRESIMS for 5**

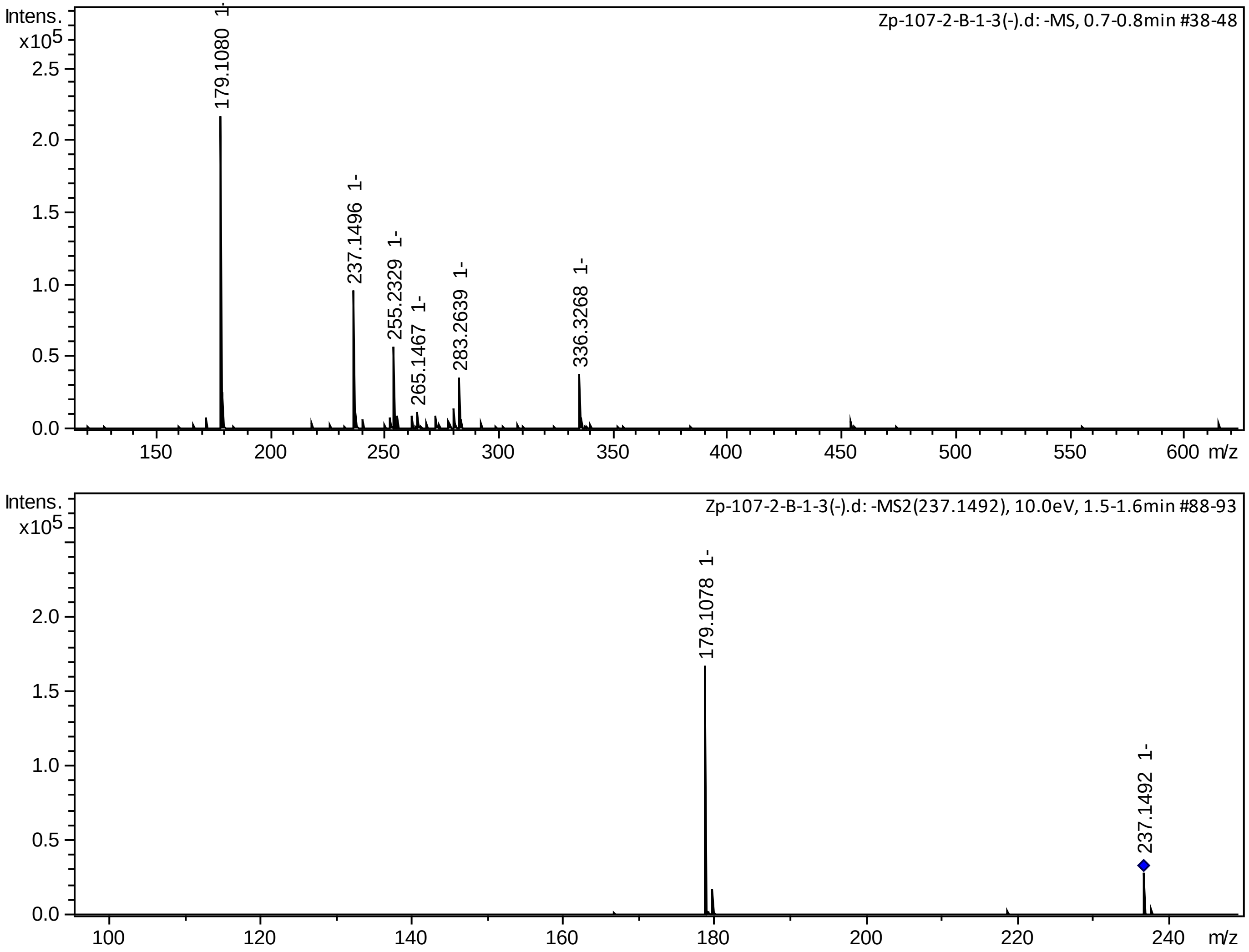

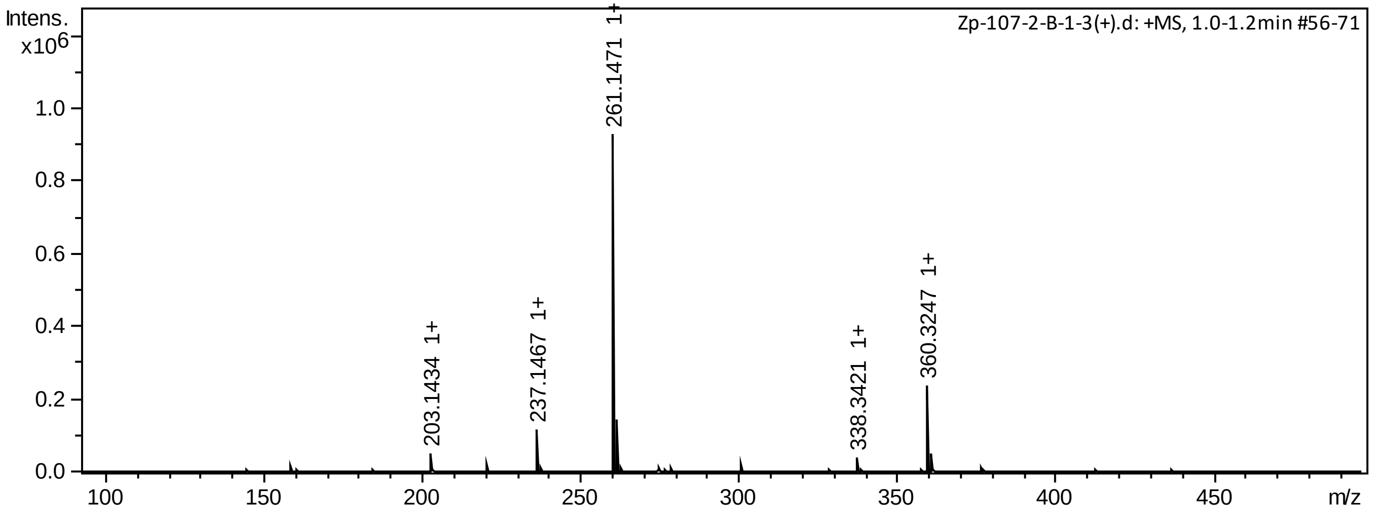


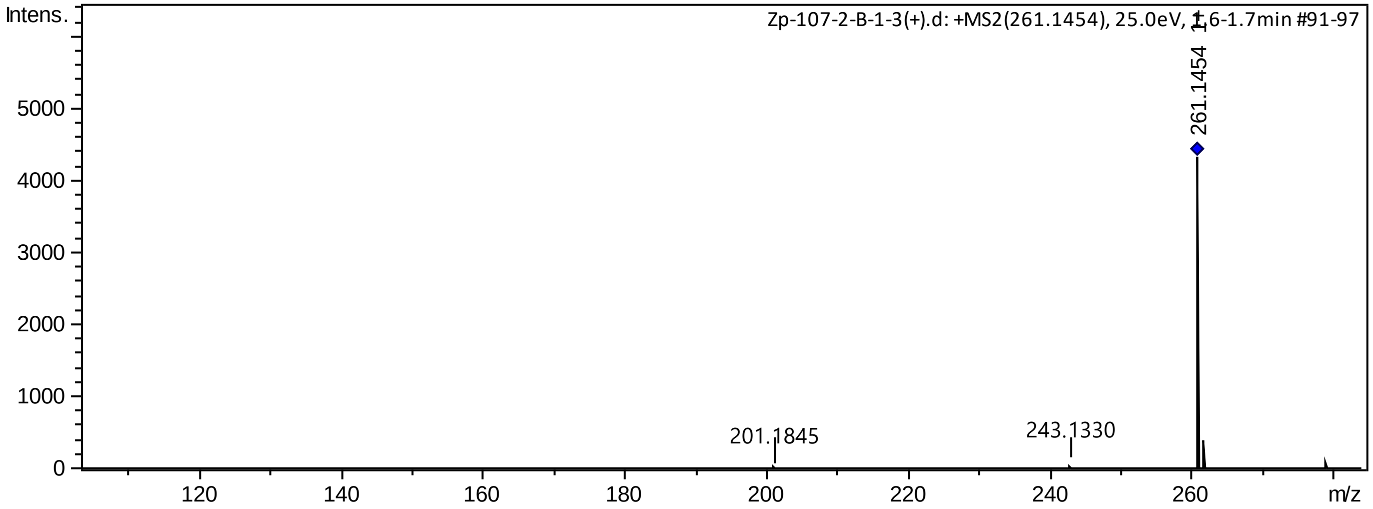


| | meas | calc | Δ (ppm) |
|---|---|---|---|
| $[M-H]^-$ | 237,1496 | 237,1496 | 0,0 |
| $[M+Na]^+$ | 261,1471 | 261,1461 | -3,8 |

**Figure S33. ¹H NMR spectrum of 5 measured at 700 MHz in $CDCl_3$**

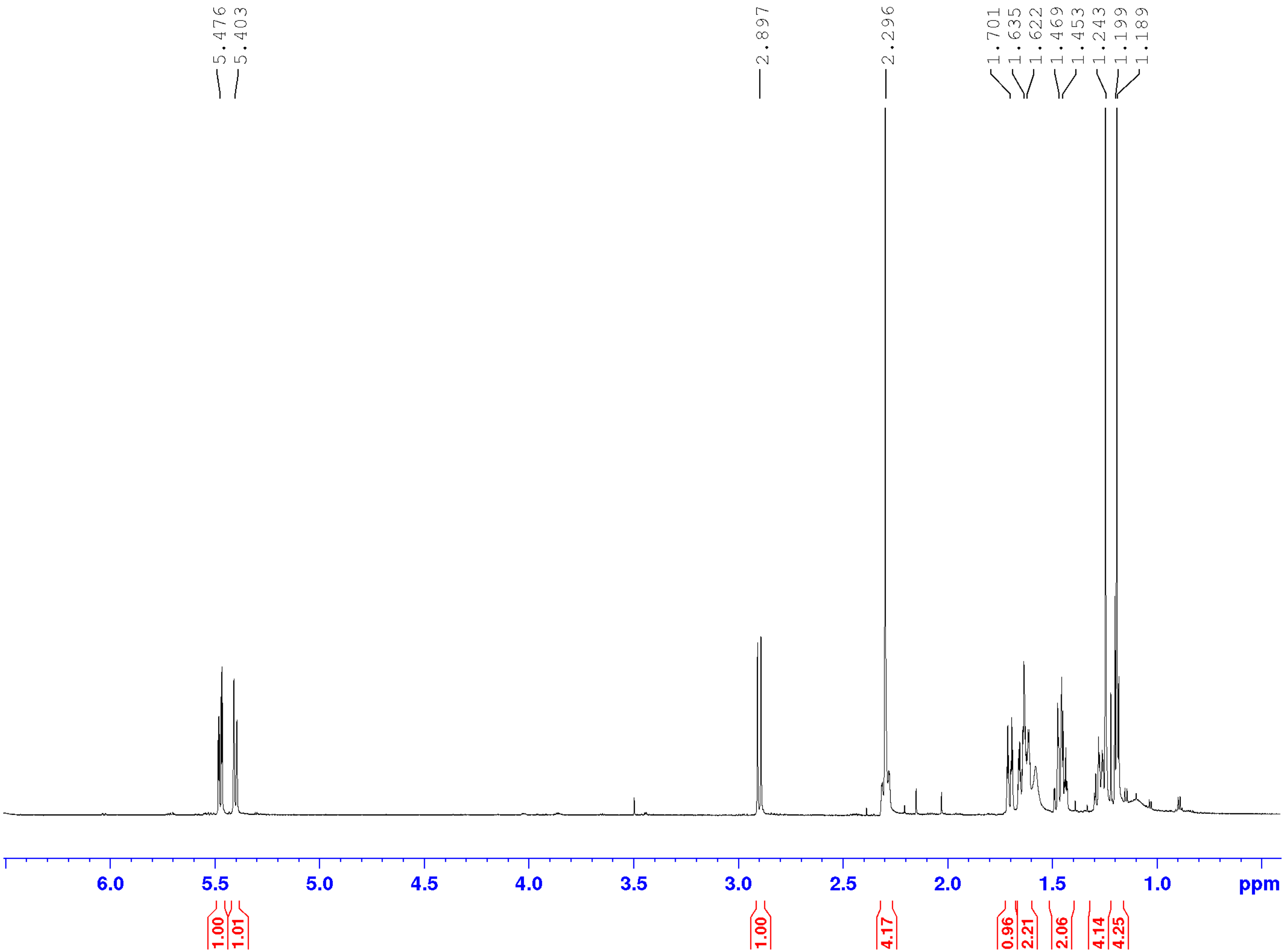

**Figure S34.$^{13}$C NMR spectrum of 5 measured at 176 MHz in $CDCl_3$**

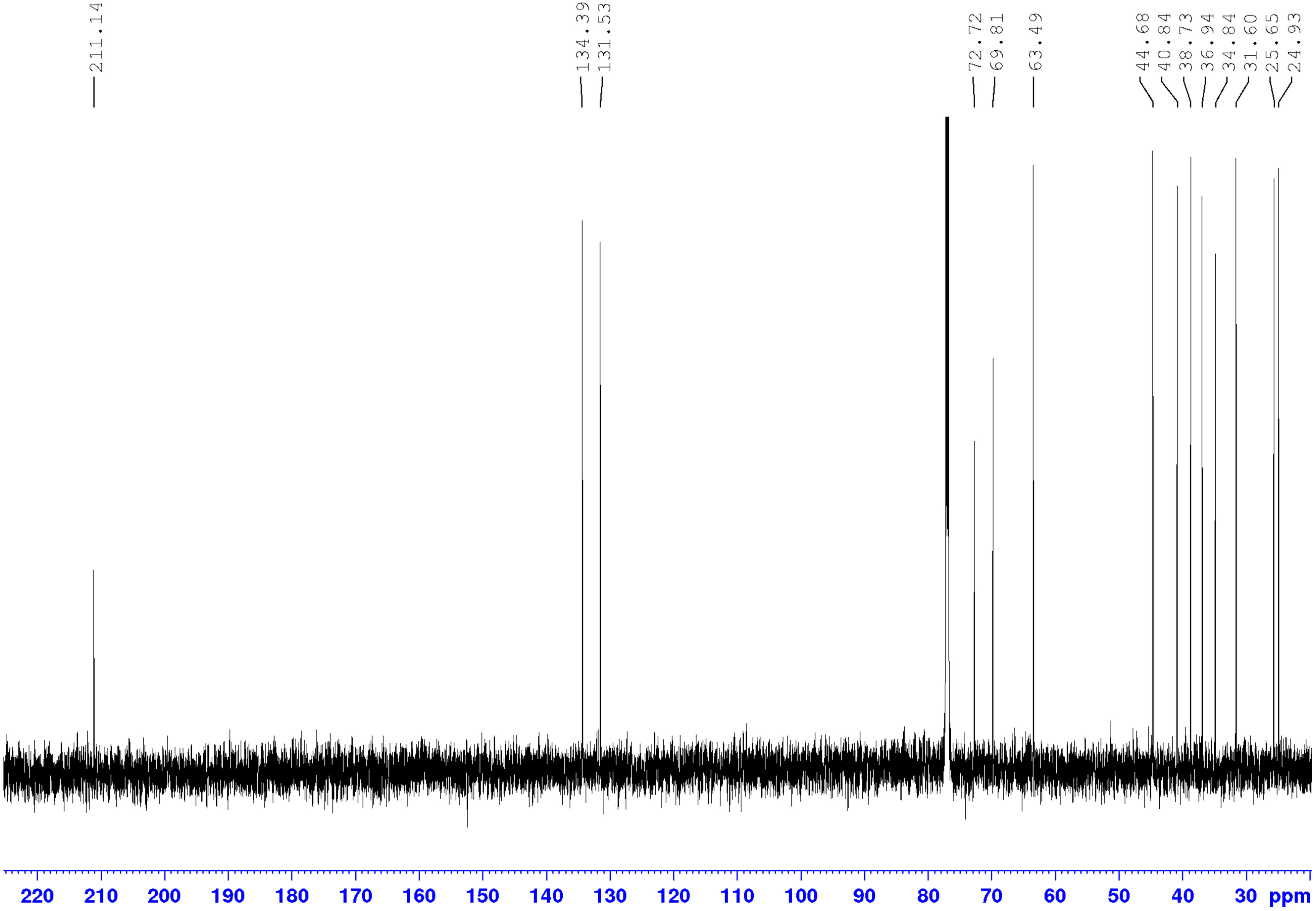

**Figure S35. DEPT-135 spectrum of 5 measured at 176 MHz in $CDCl_3$**

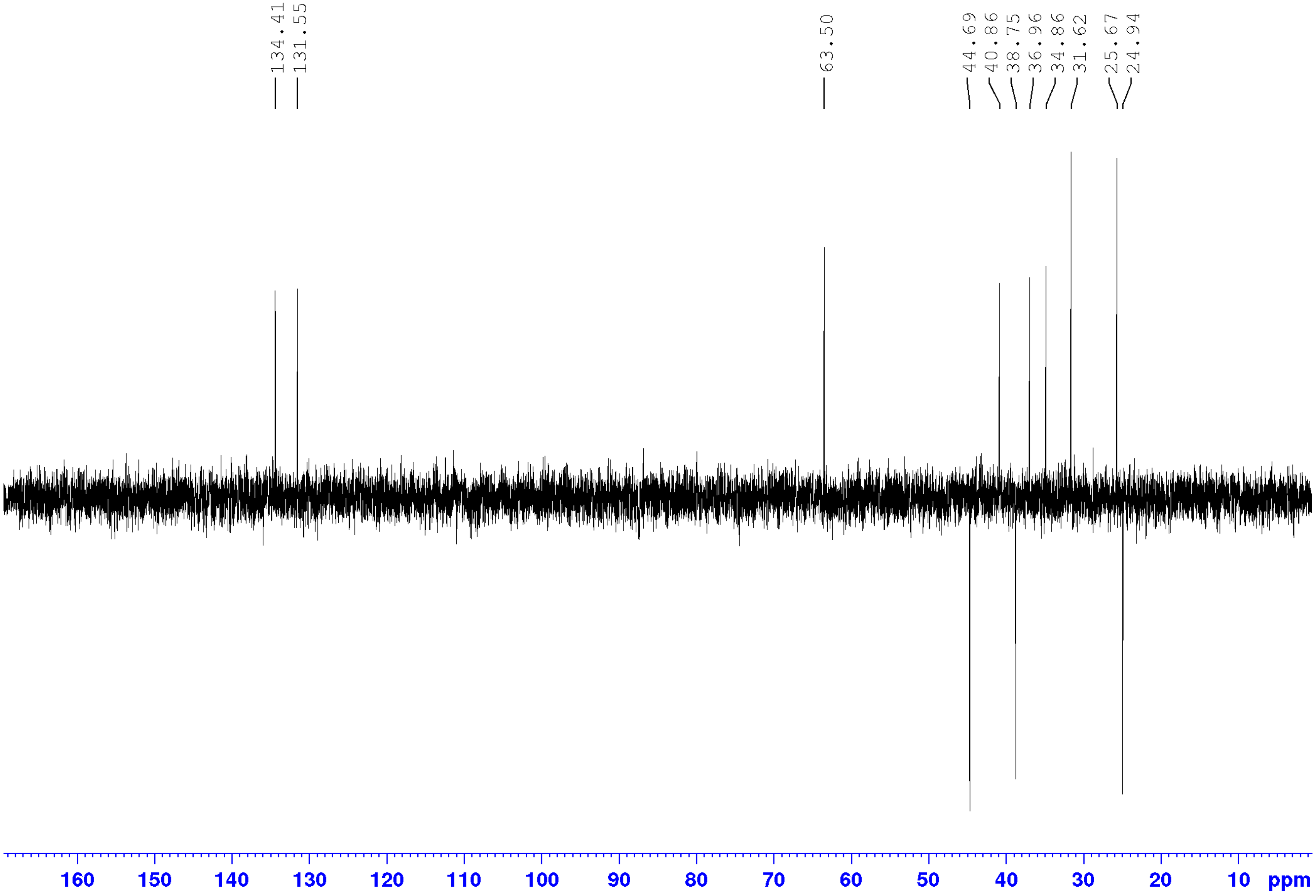

**Figure S36. HSQC spectrum of 5 measured in $CDCl_3$**

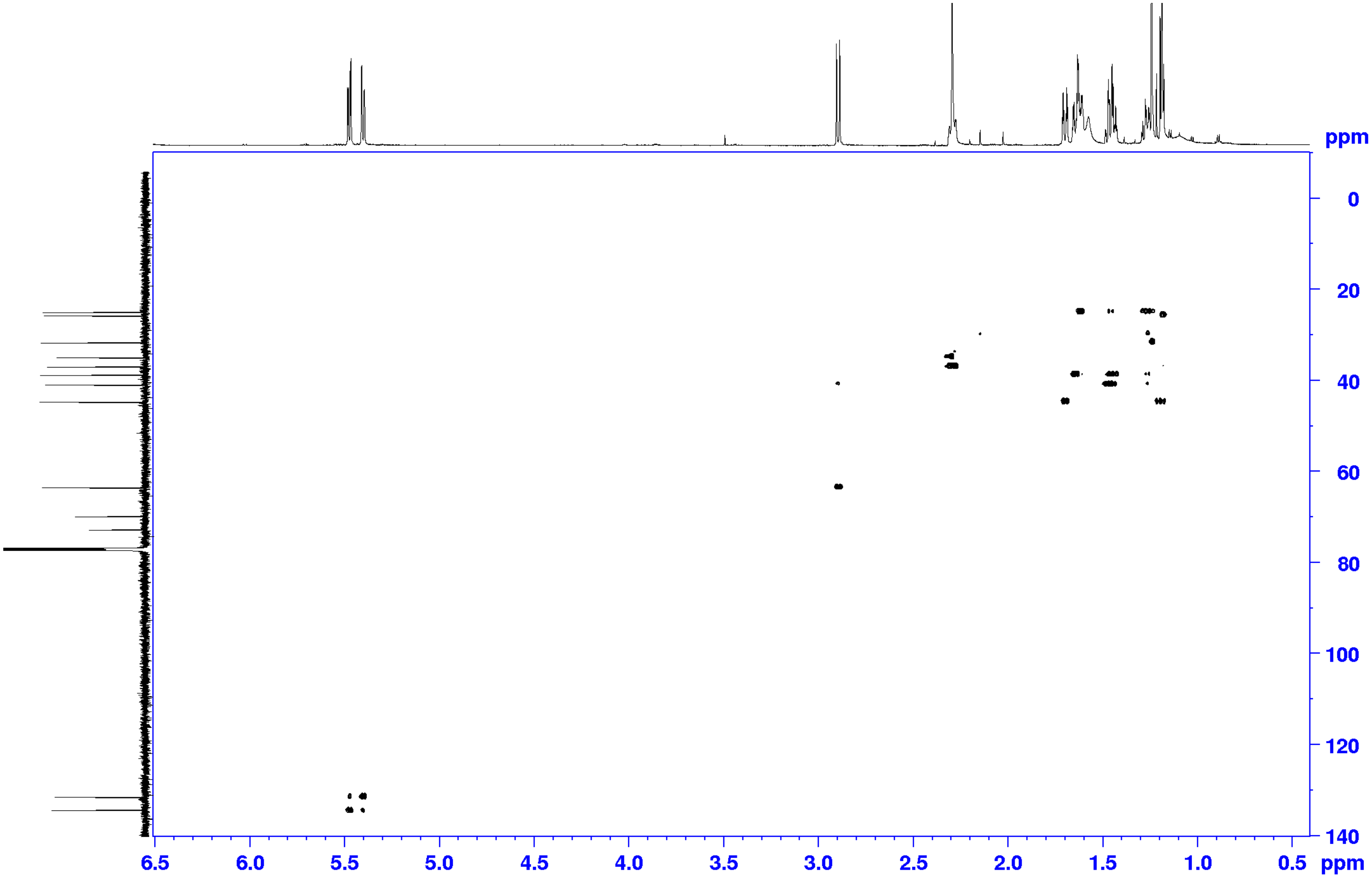

**Figure S37. COSY spectrum of 5measured in $CDCl_3$**

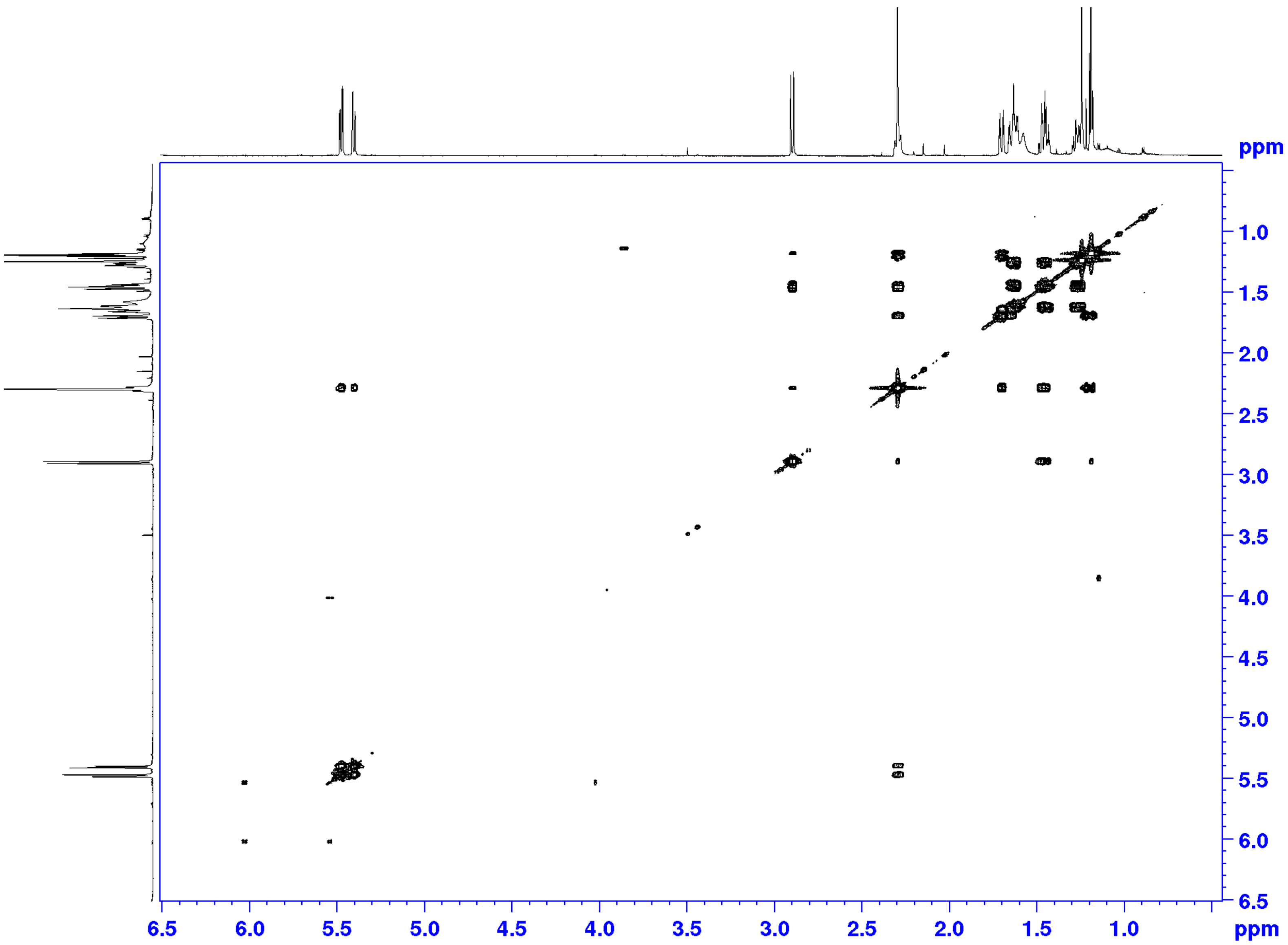

**Figure S38. HMBC spectrum of 5 measured in CDCl3**

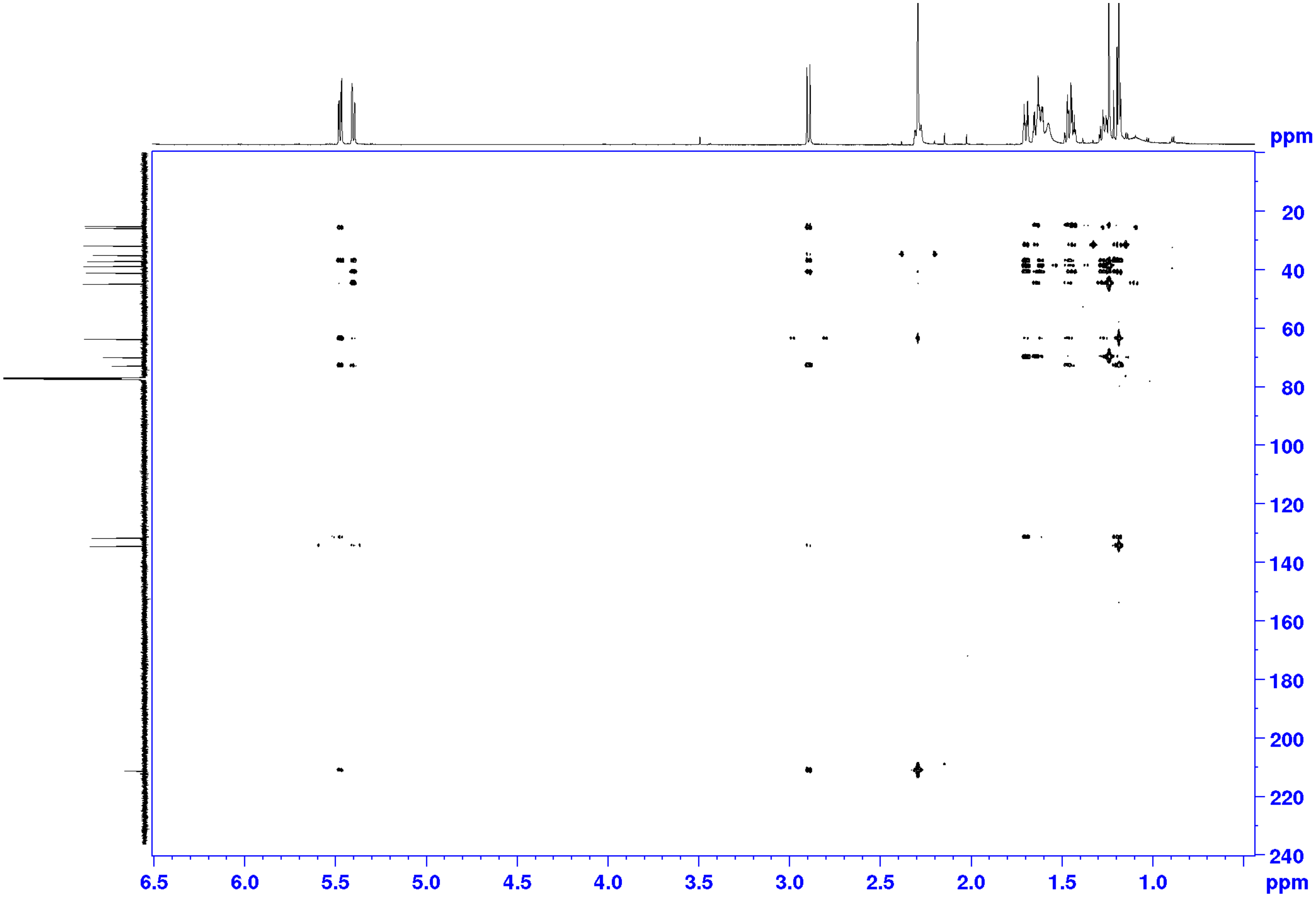

**Figure S39. ROESY spectrum of 5 measured in CDCl3**

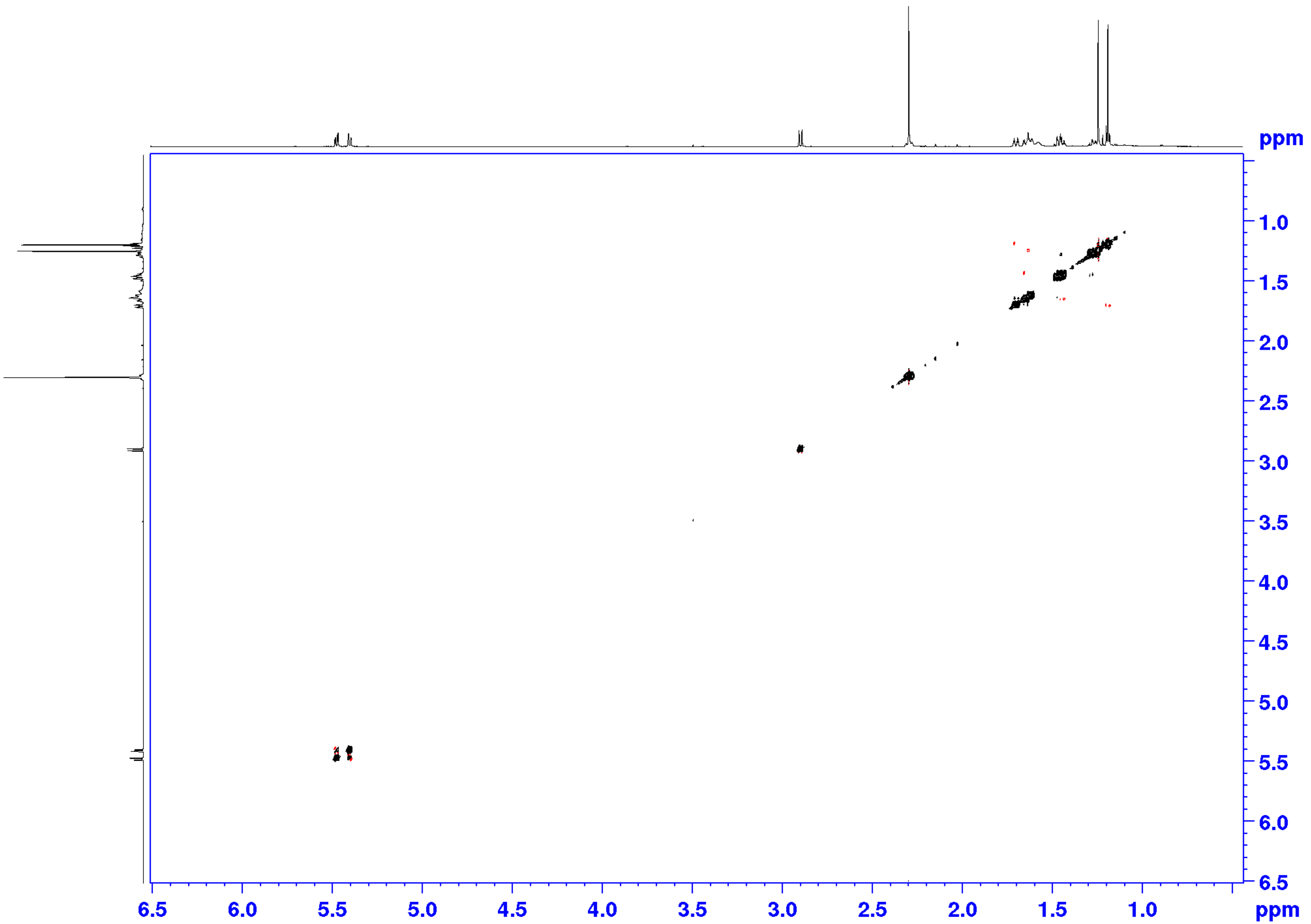

**Figure S40. HRESIMS for 6**

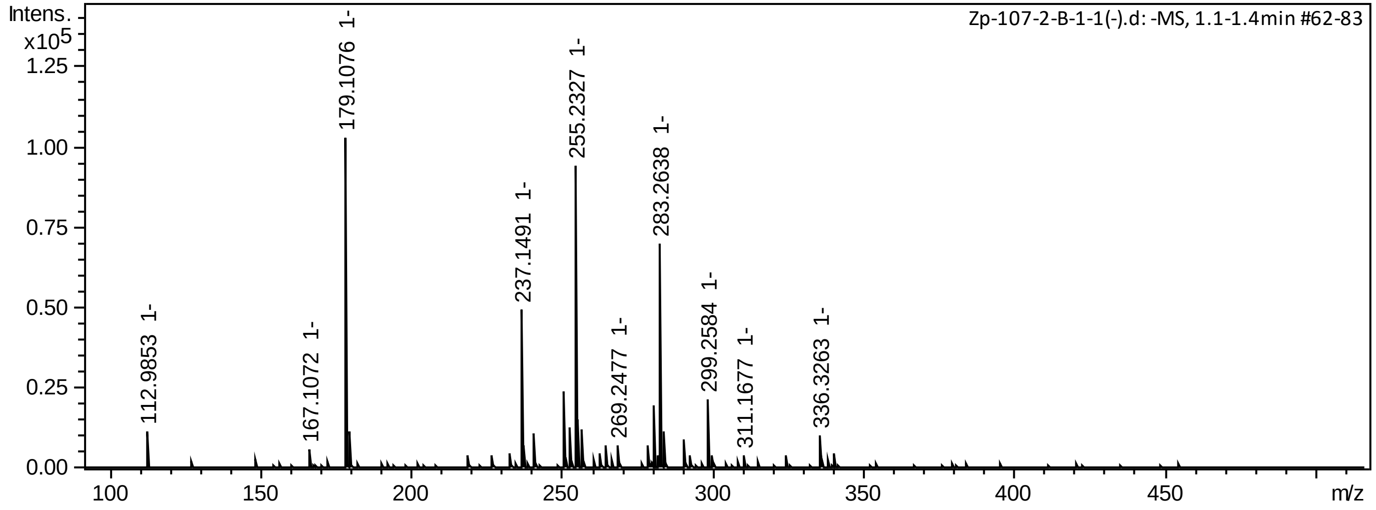


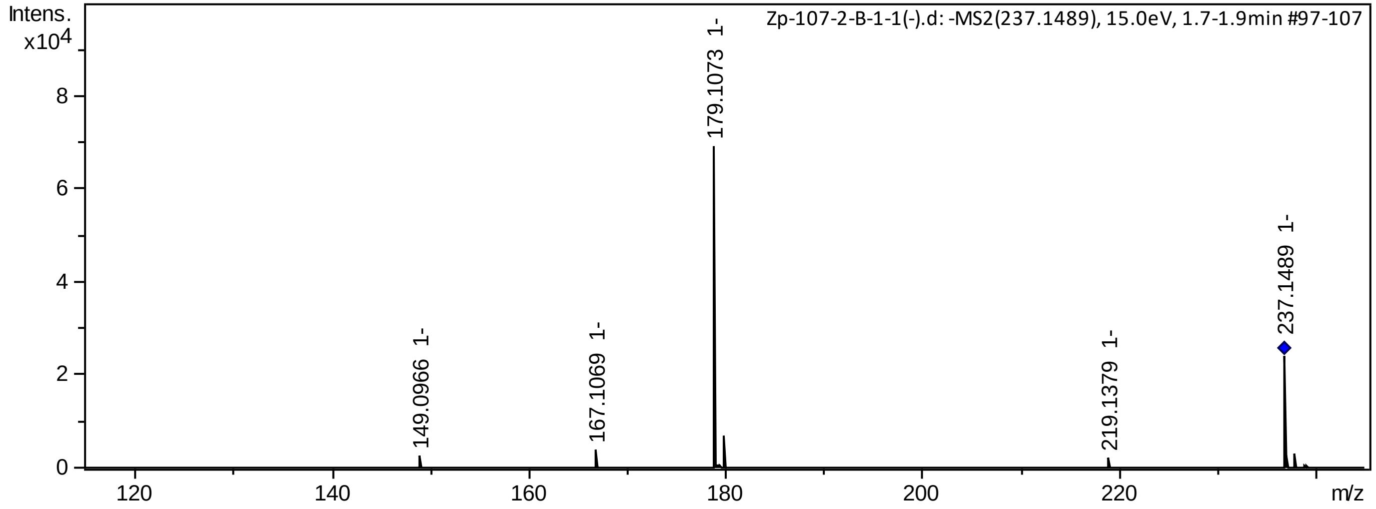

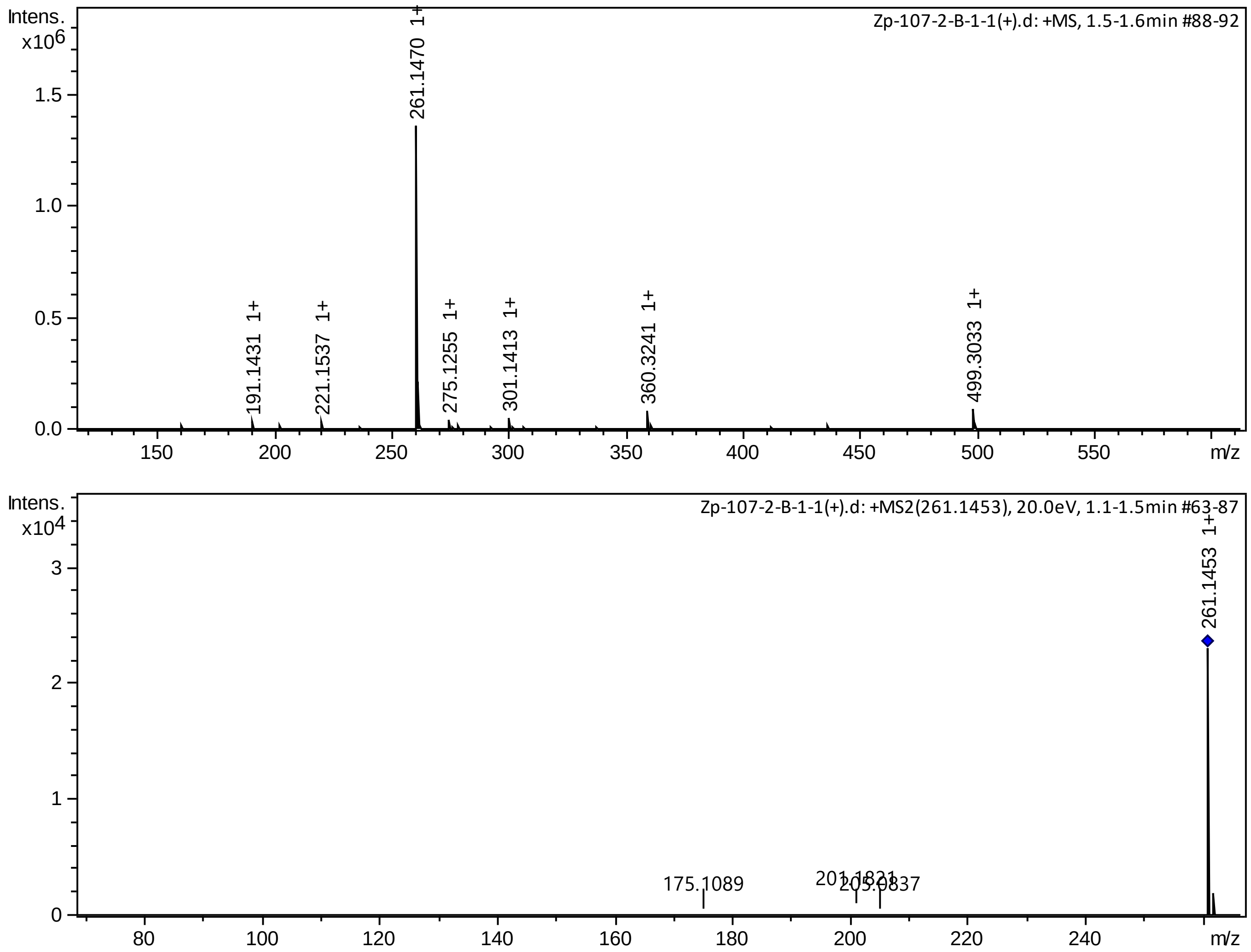


| | meas | calc | Δ (ppm) |
|---|---|---|---|
| $[M-H]^-$ | 237,1491 | 237,1496 | 2,1 |
| $[M+Na]^+$ | 261,147 | 261,1461 | -3,4 |

**Figure S41. $^1$H NMR spectrum of 6 measured at 700 MHz in $CDCl_3$**

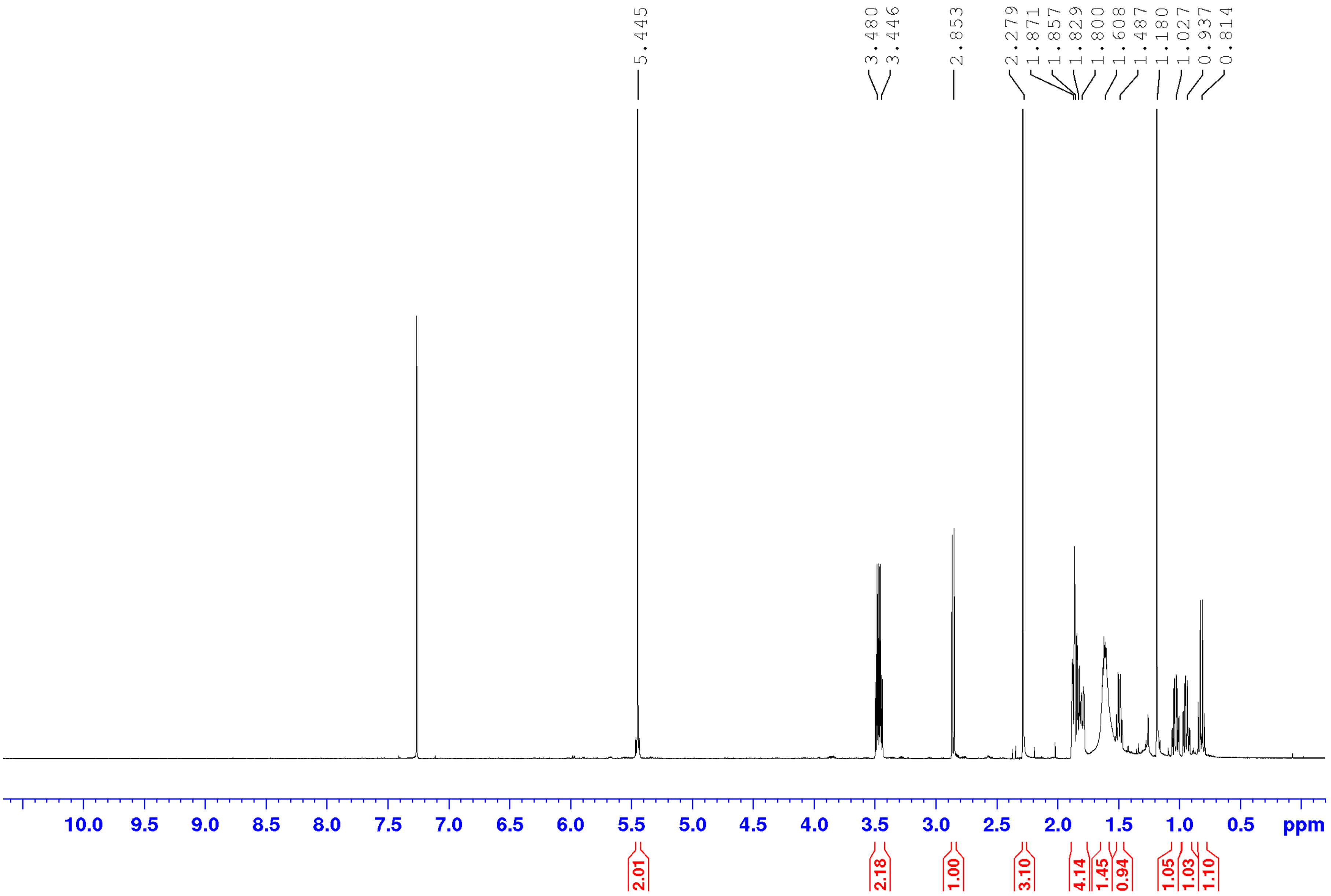

**Figure S42.** $^{13}C$ NMR spectrum of 6 measured at 176 MHz in $CDCl_3$

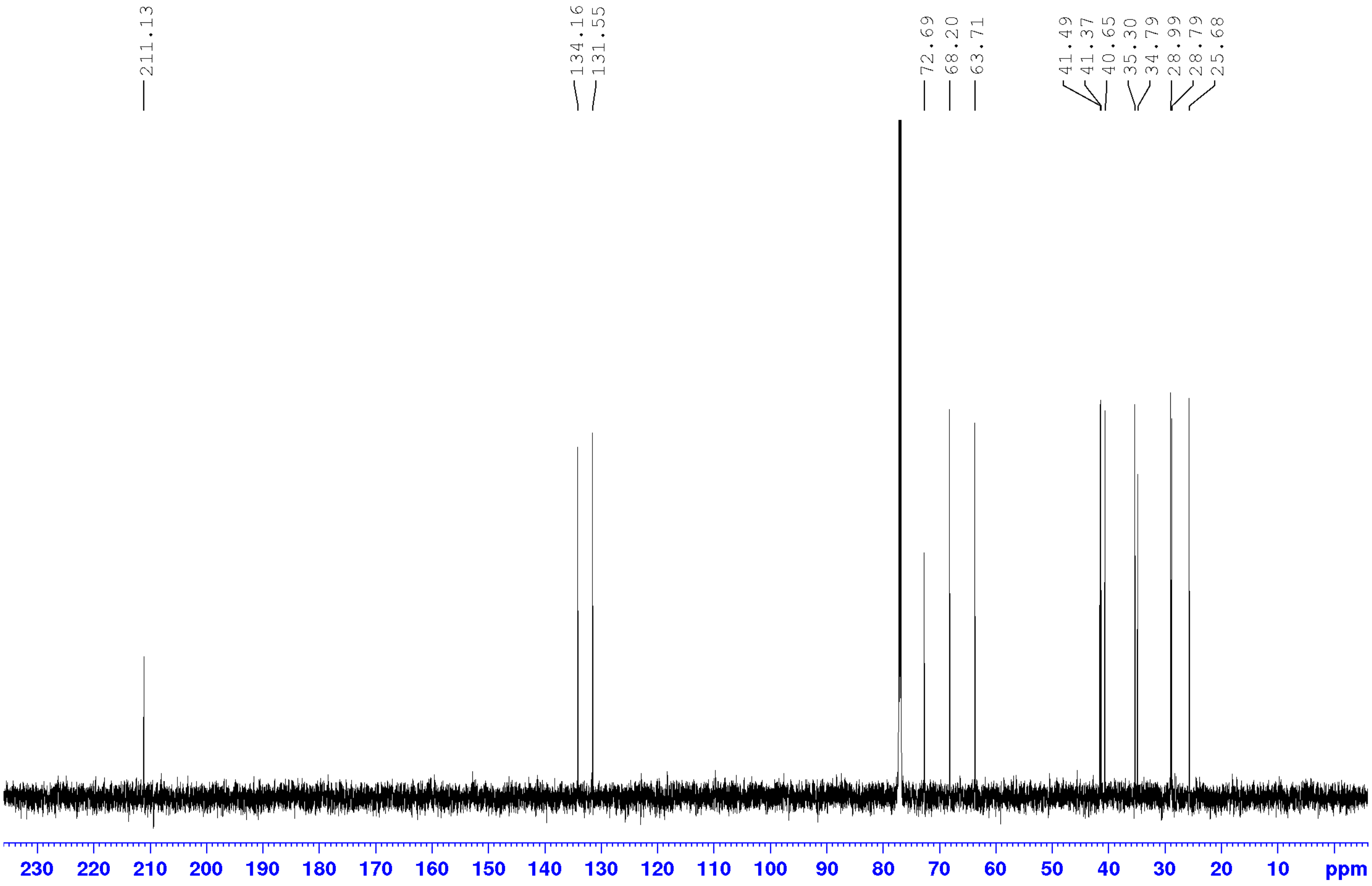

**Figure S43. DEPT-135 spectrum of 6 measured at 176 MHz in $CDCl_3$**

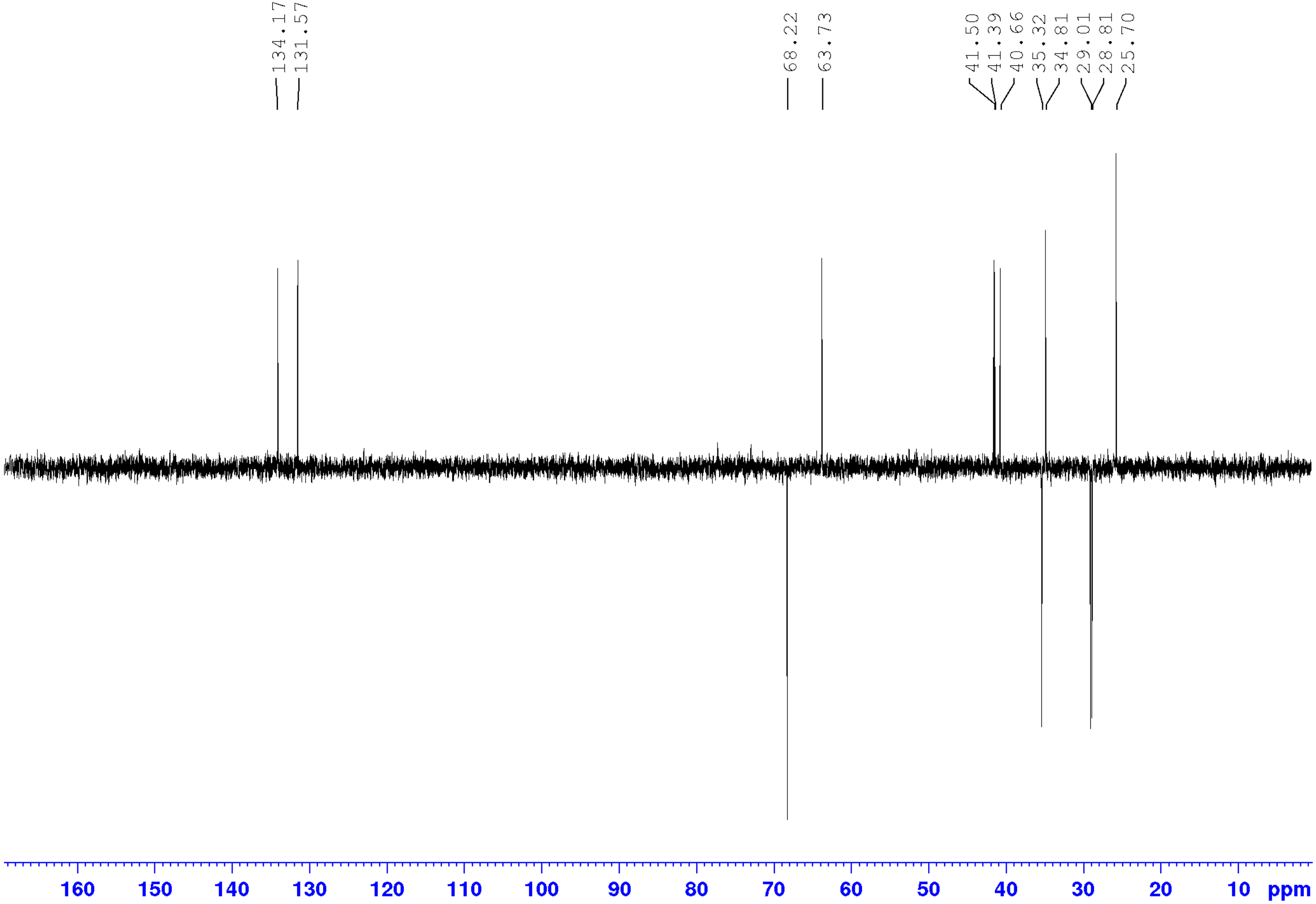

**Figure S44. HSQC spectrum of 6 measured in $CDCl_3$**

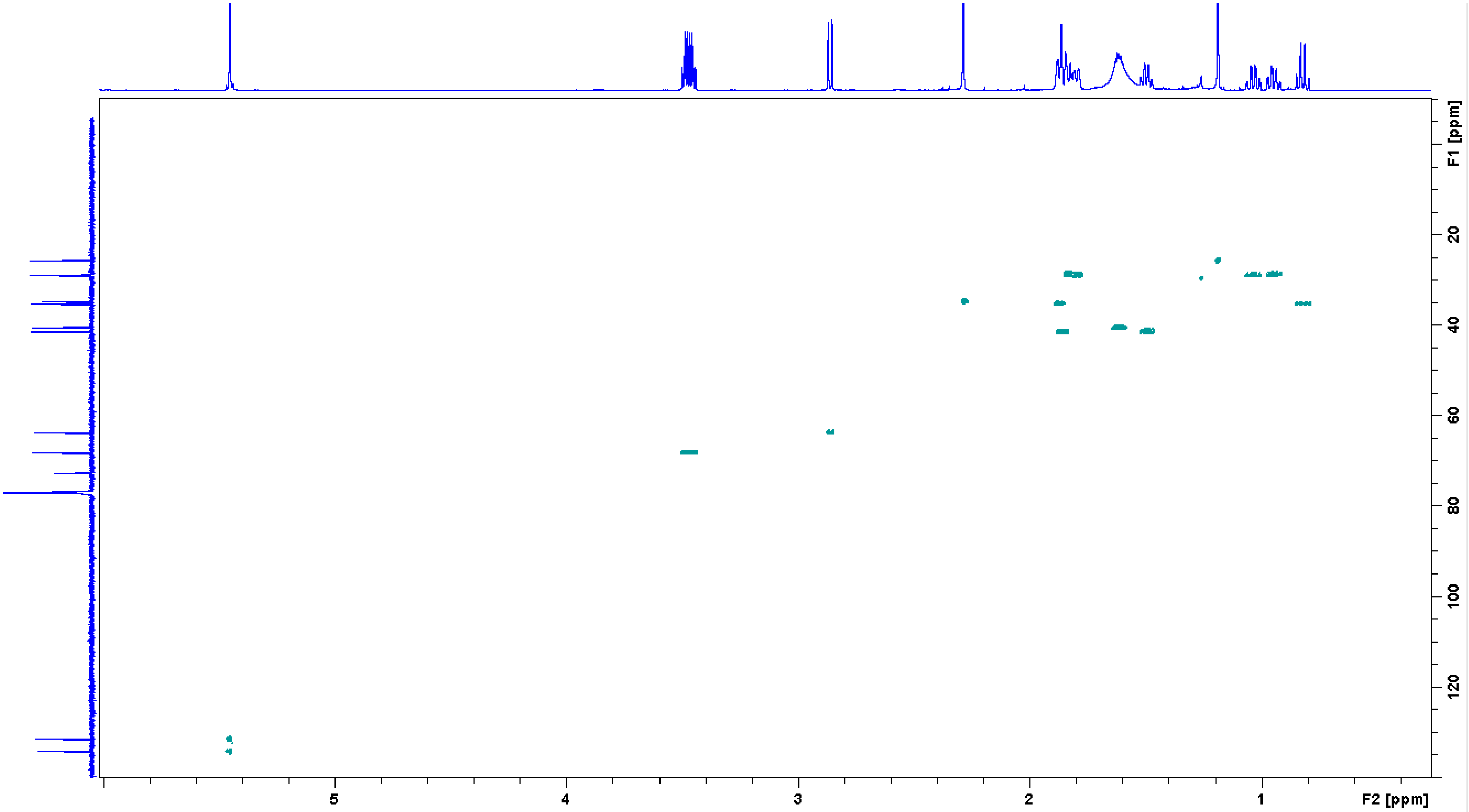

**Figure S45. COSY spectrum of 6 measured in $CDCl_3$**

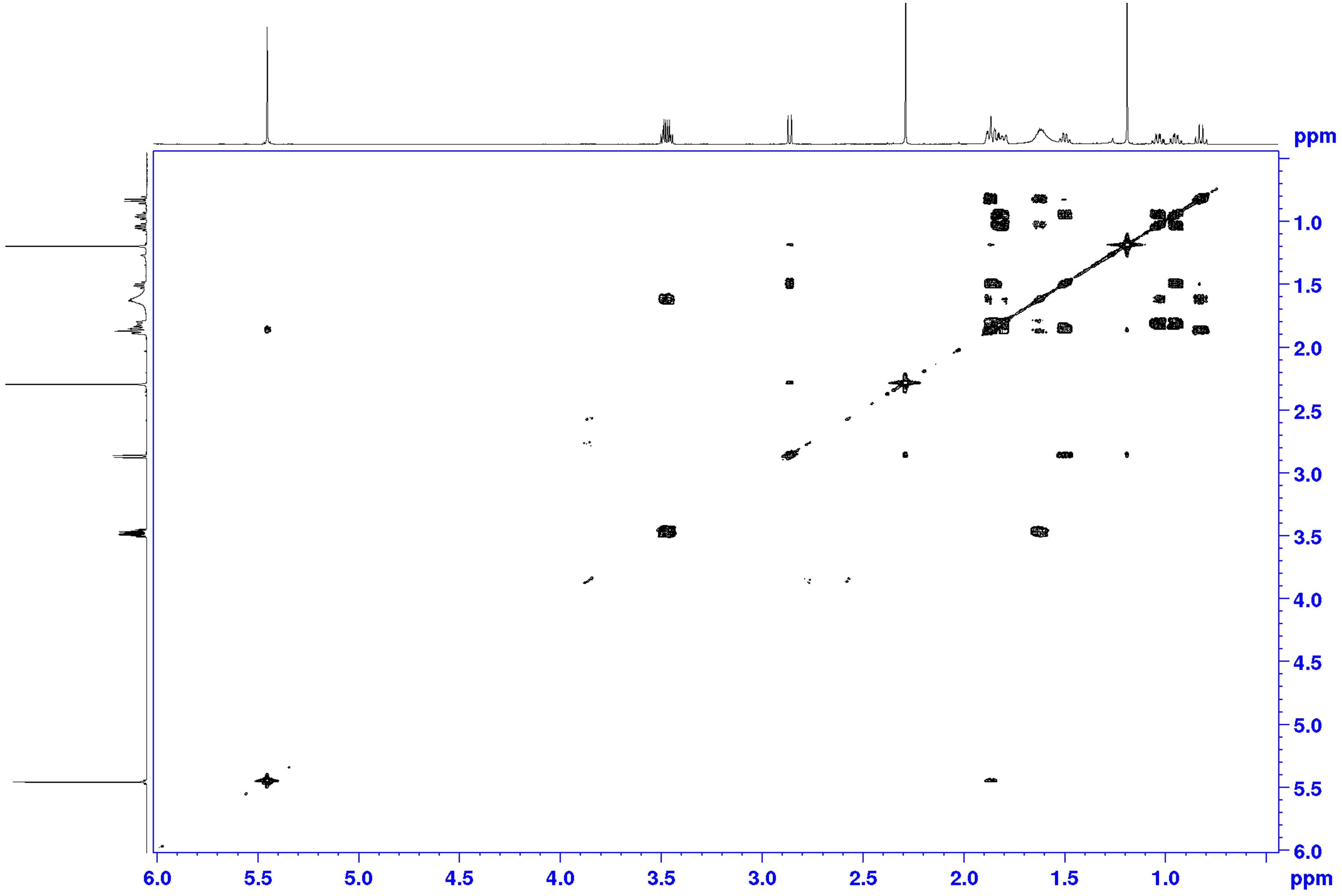

**Figure S46. HMBC spectrum of 6 measured in CDCl$_3$**

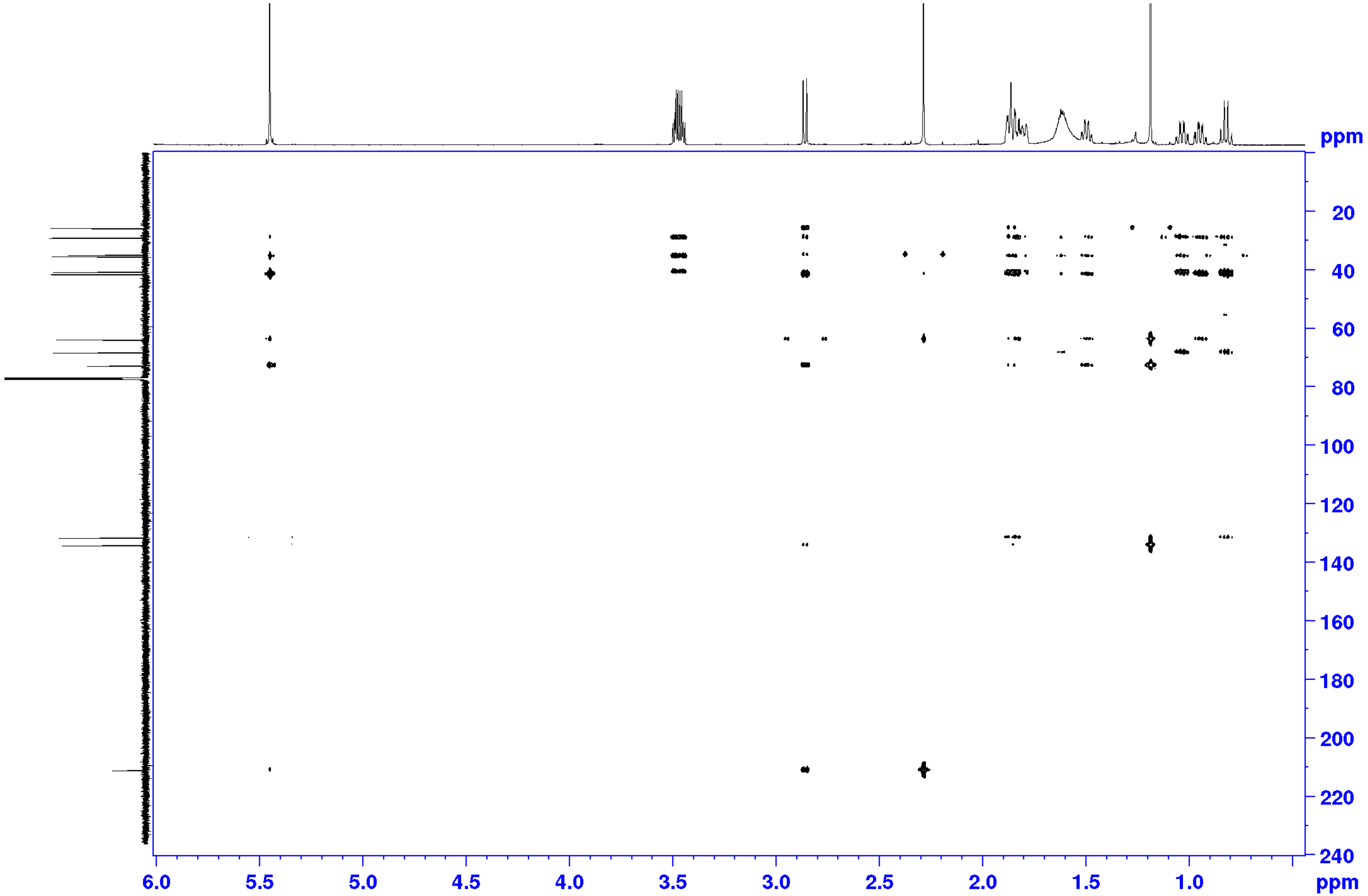

**Figure S47. ROESY spectrum of 6 measured in CDCl₃**

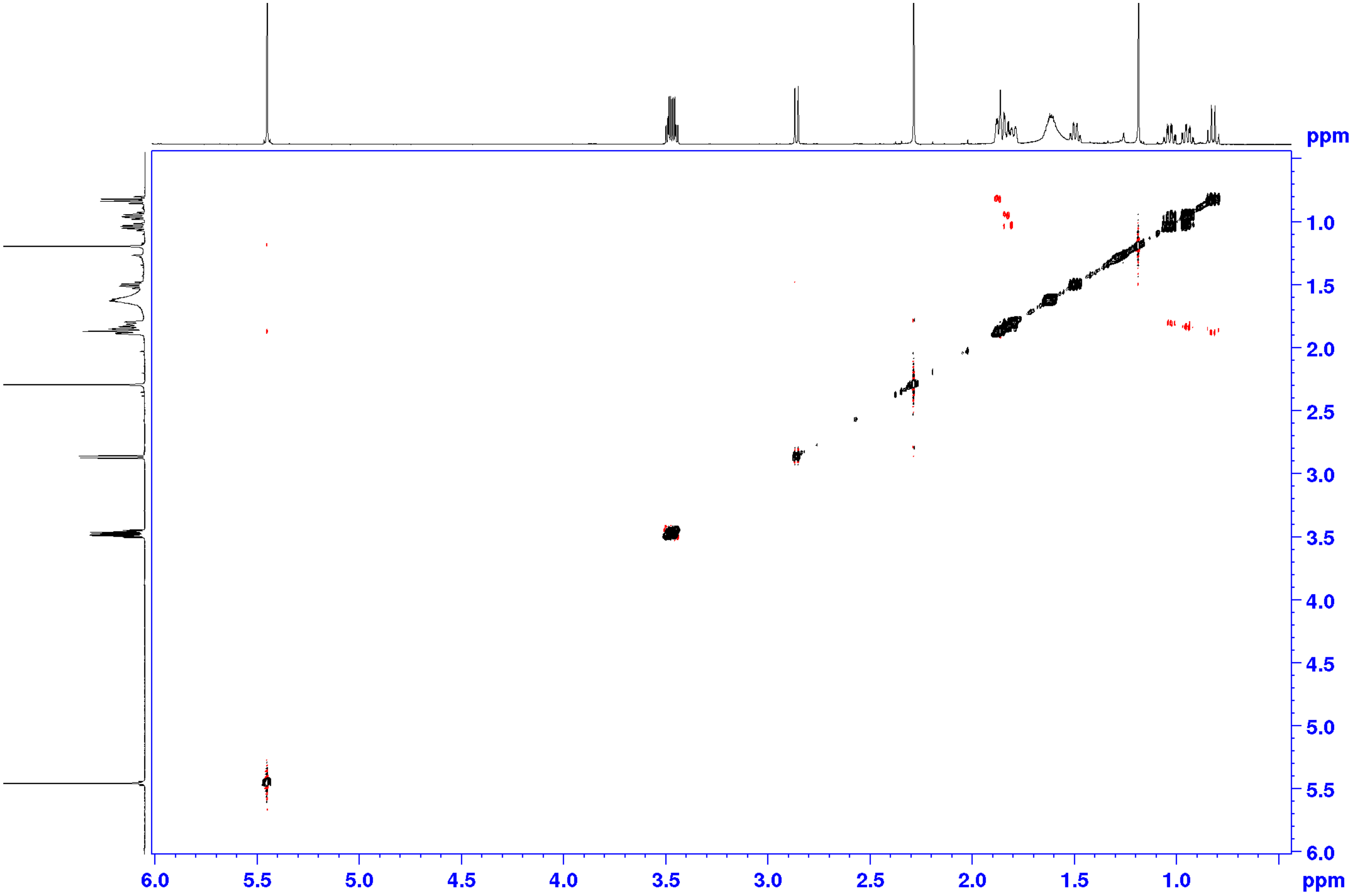

**Figure S48. HRESIMS for 7**

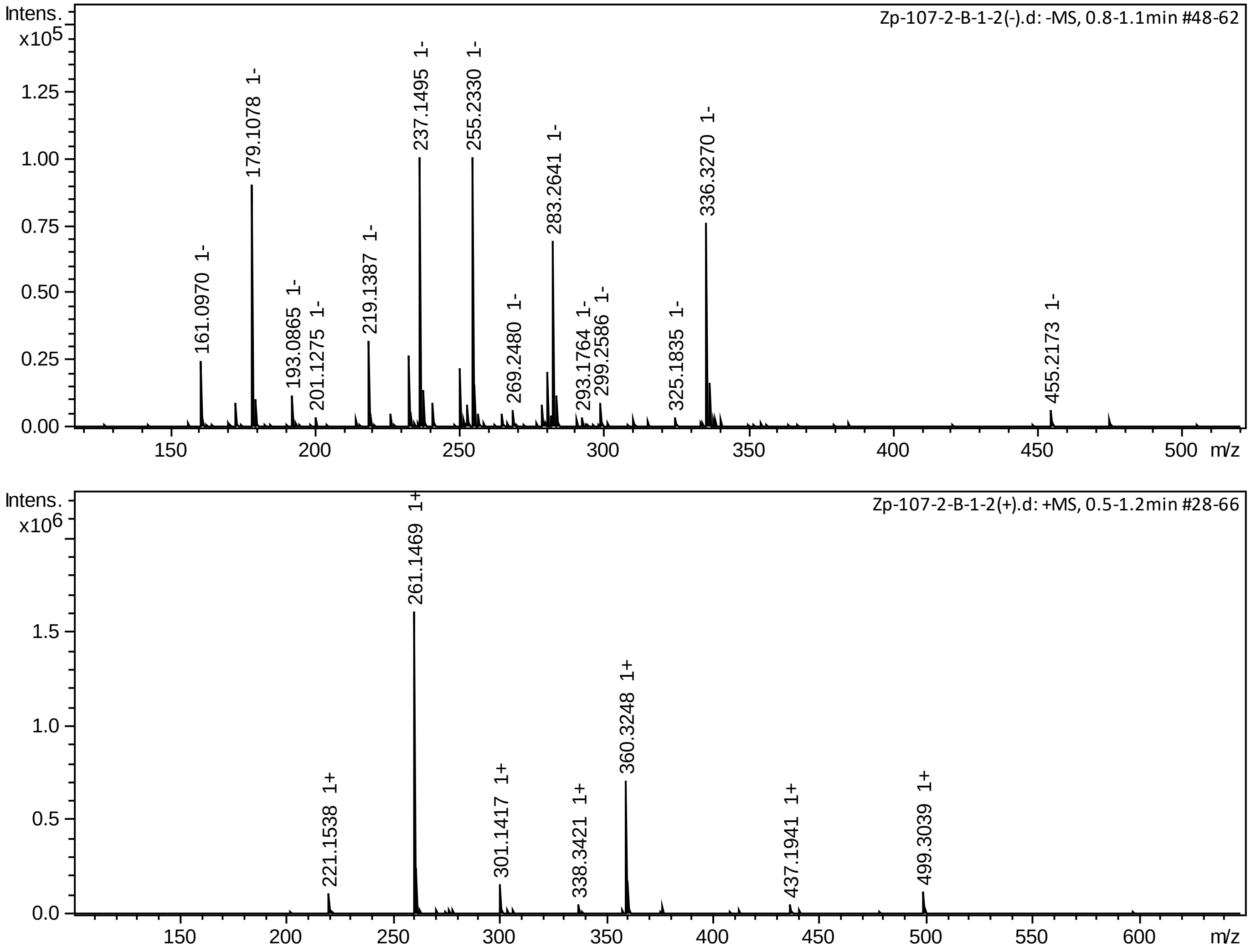

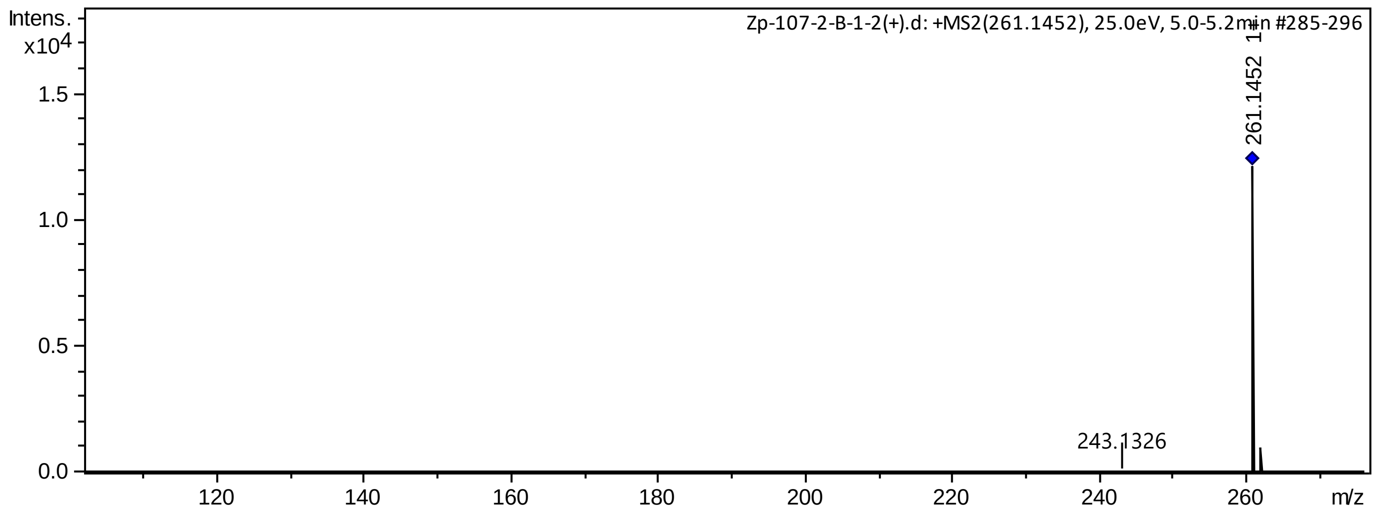


| | meas | calc | Δ (ppm) |
|---|---|---|---|
| $[M-H]^-$ | 237,1495 | 237,1496 | 0,4 |
| $[M+Na]^+$ | 261,1469 | 261,1461 | -3,1 |

**Figure S49.** $^1$H NMR spectrum of 7 measured at 700 MHz in $CDCl_3$

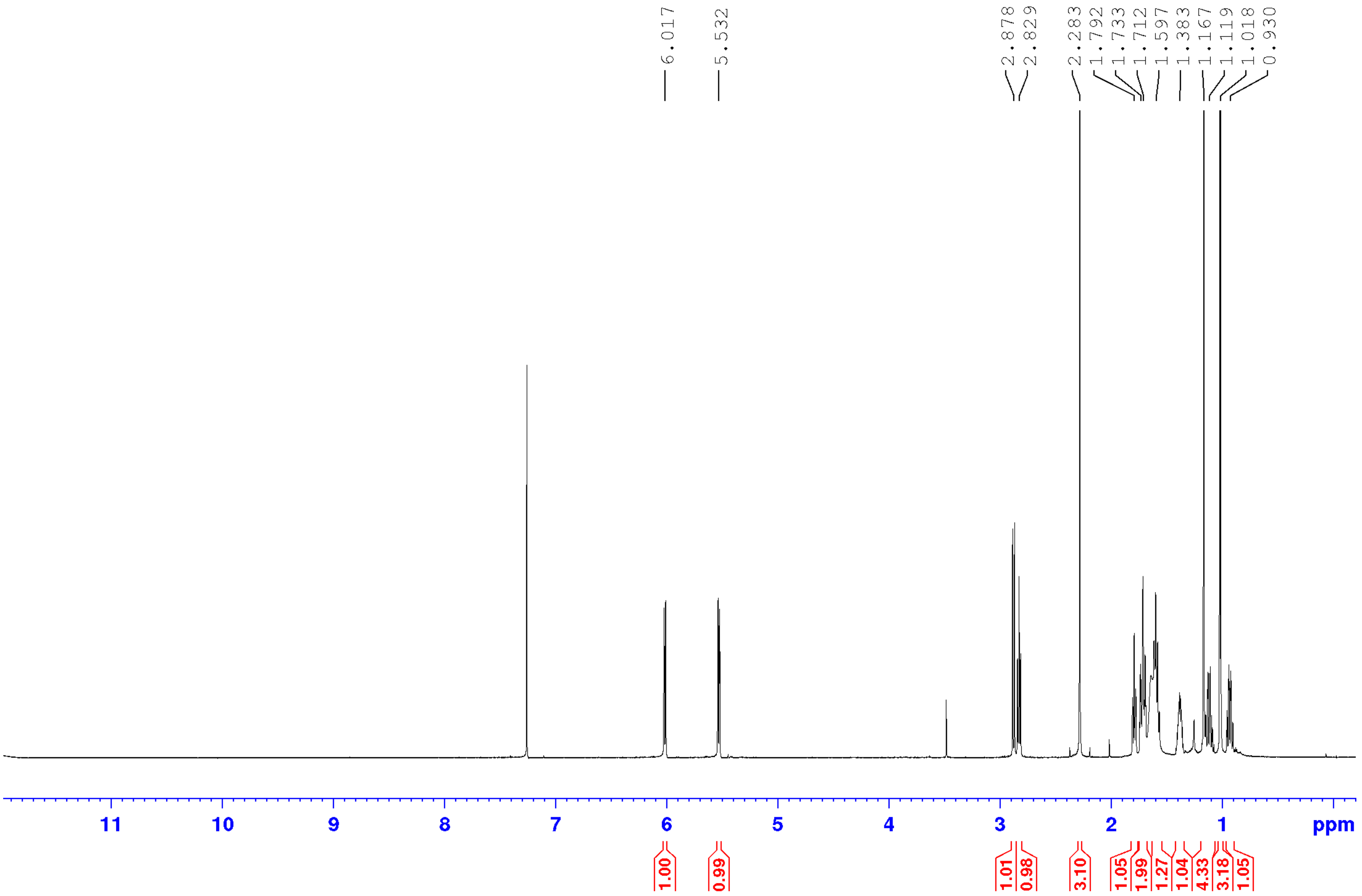

**Figure S50.**[13]**C NMR spectrum of 7 measured at 176 MHz in CDCl₃**

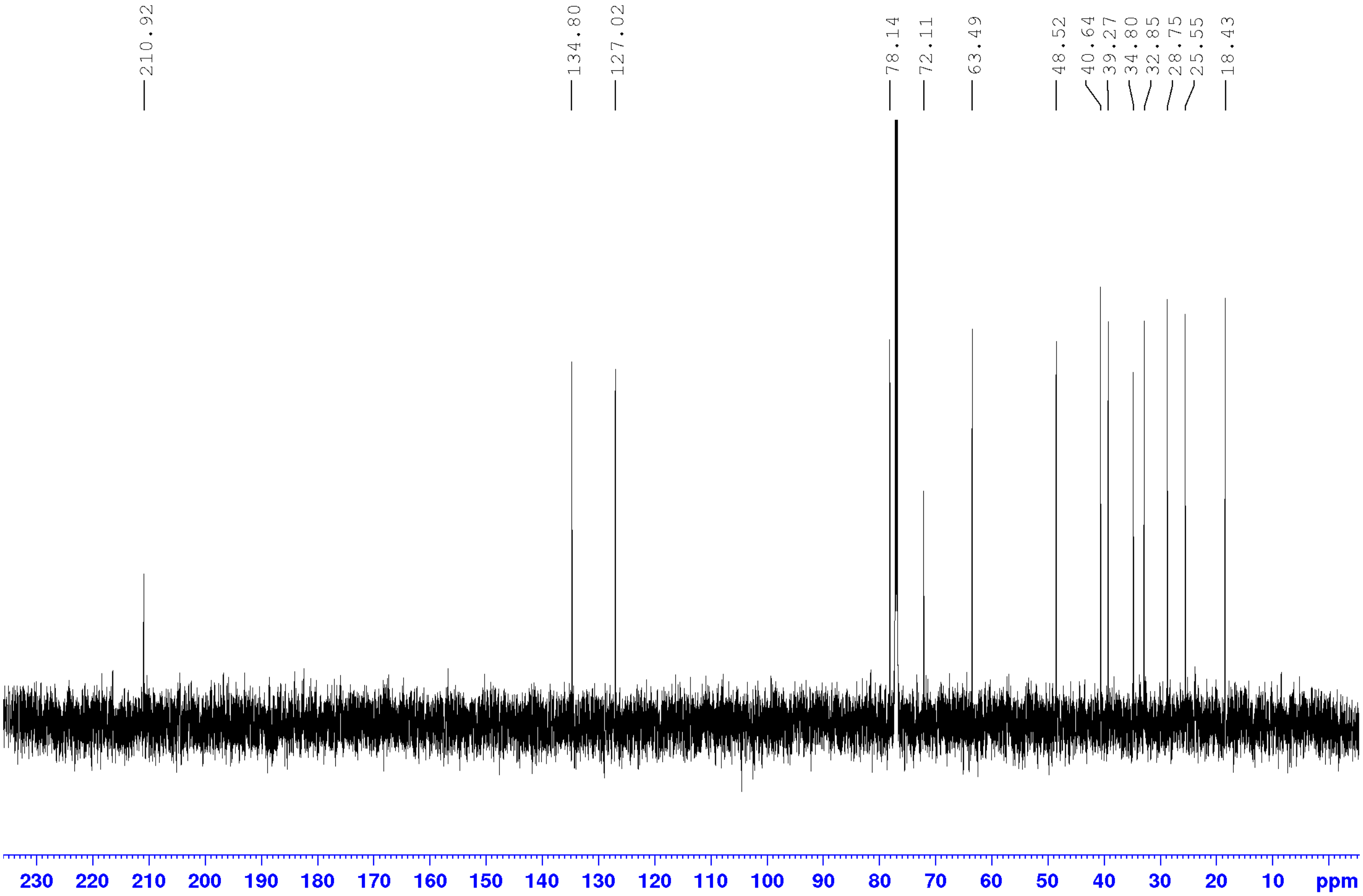

**Figure S51. DEPT-135 spectrum of 7 measured at 176 MHz in $CDCl_3$**

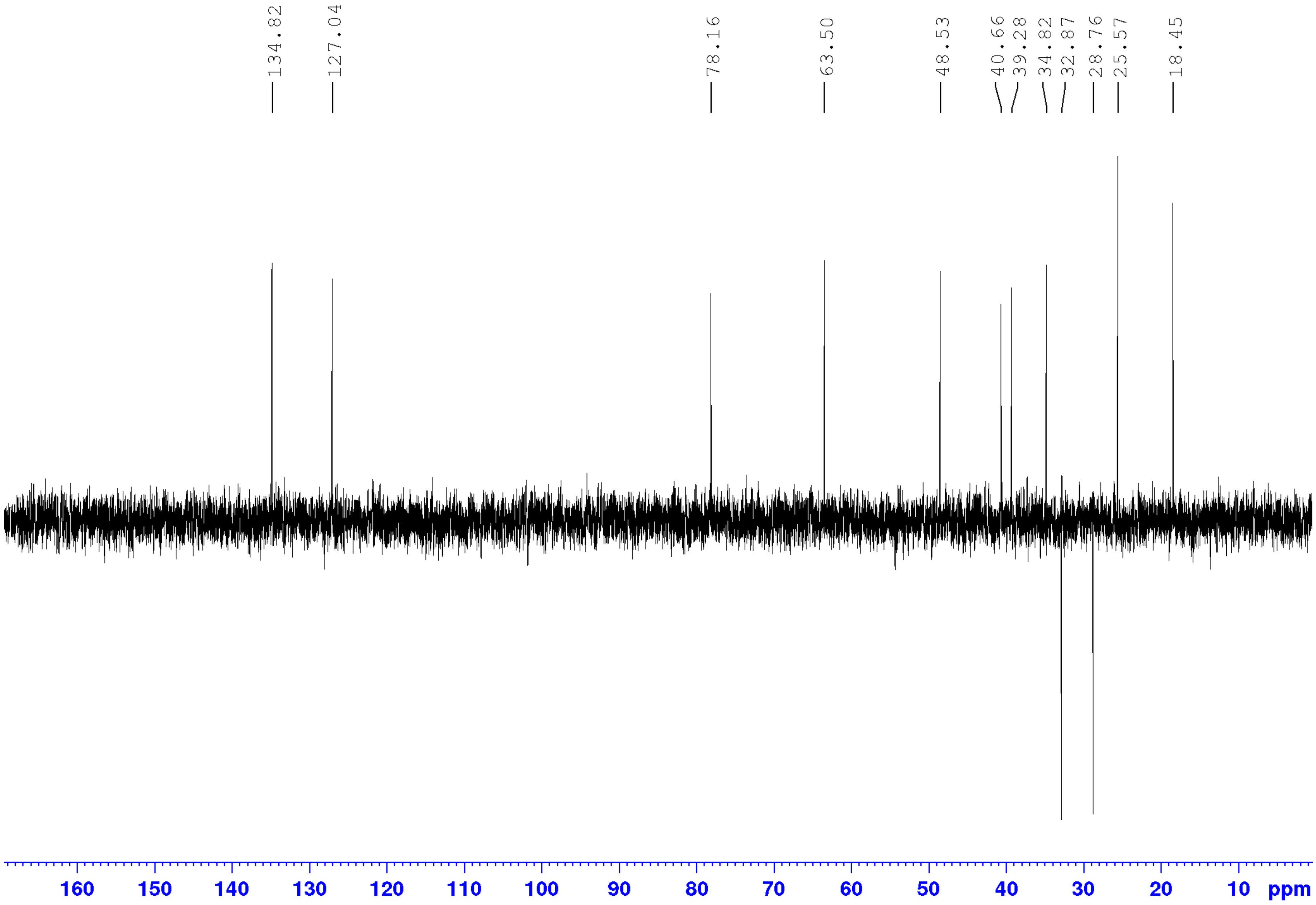

**Figure S52. HSQC spectrum of 7 measured in $CDCl_3$**

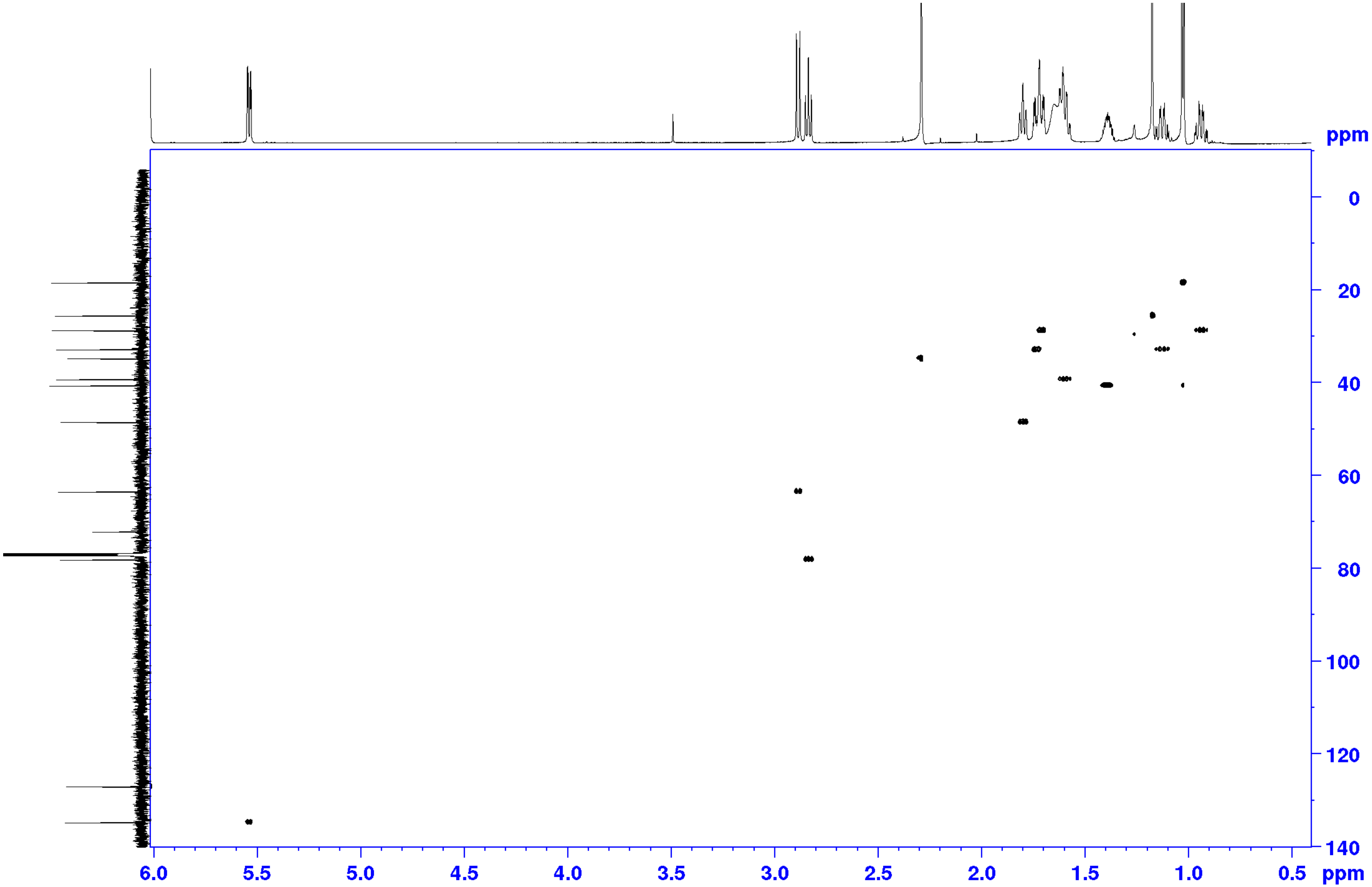

**Figure S53. COSY spectrum of 7 measured in CDCl$_3$**

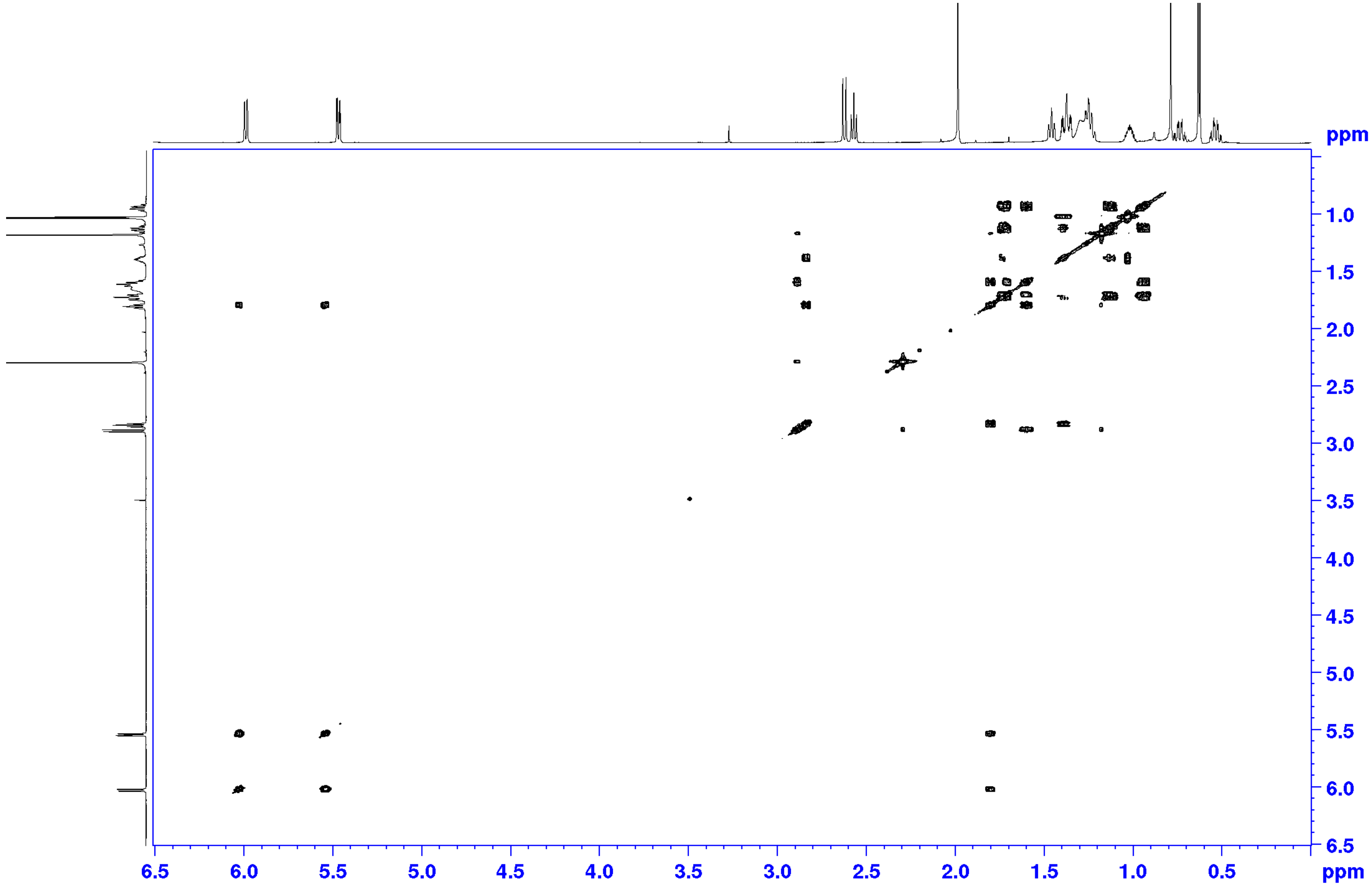

**Figure S54. HMBC spectrum of 7 measured in $CDCl_3$**

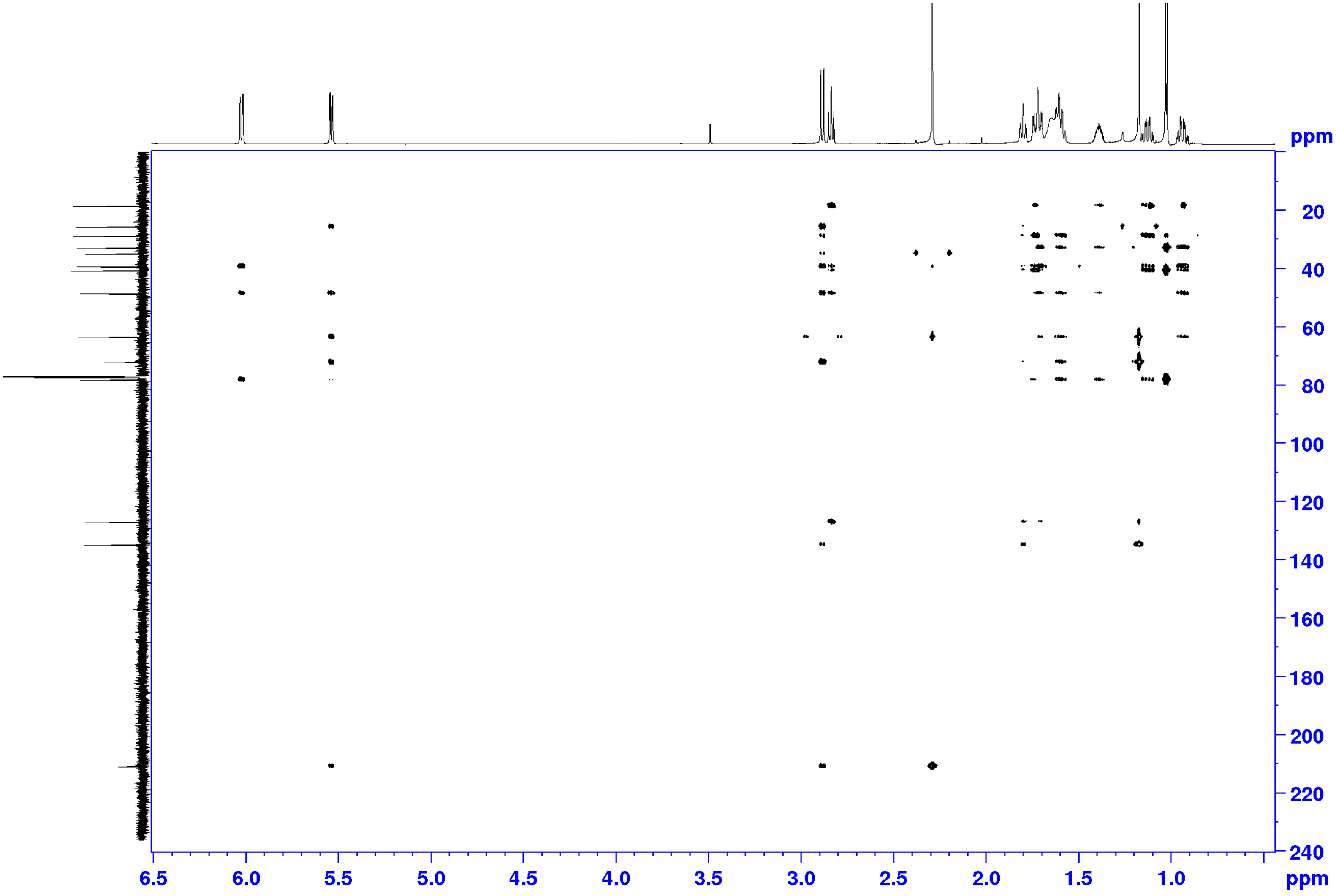

**Figure S55. ROESY spectrum of 7 measured in $CDCl_3$**

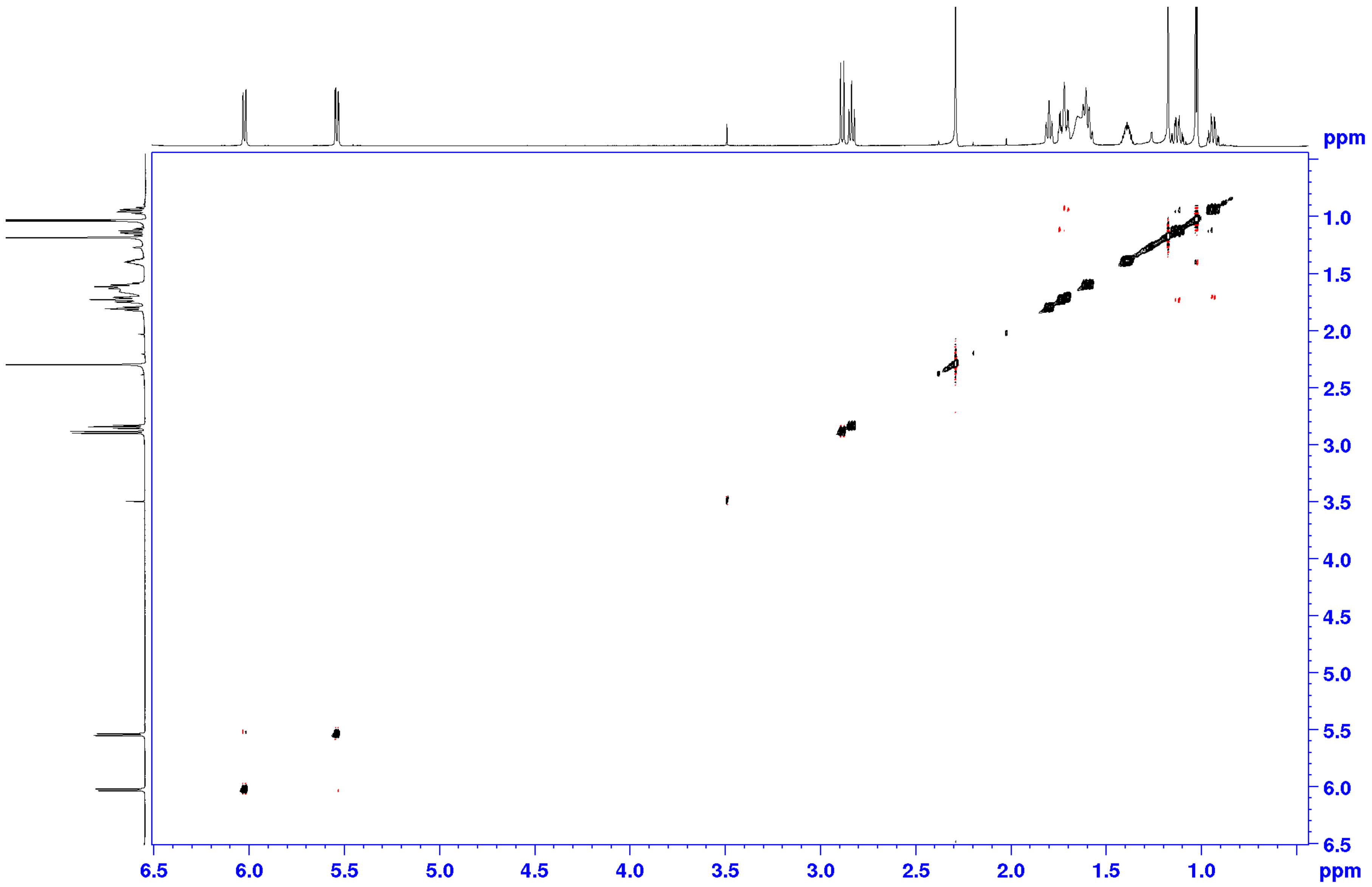

## Figure S56. HRESIMS for 8

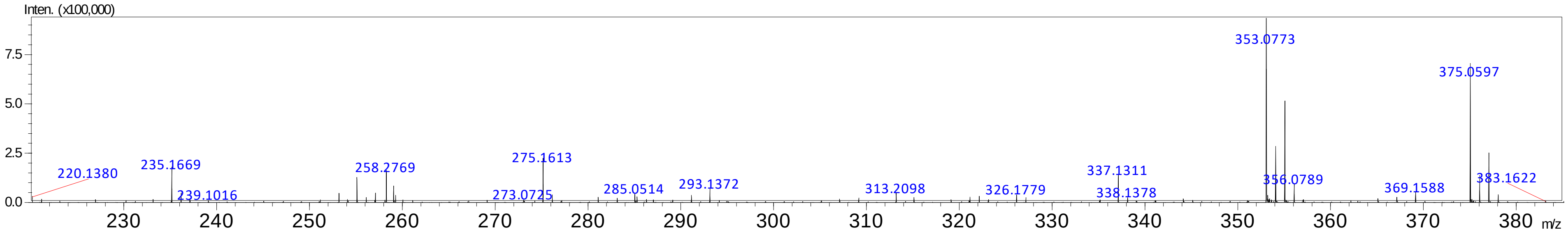


| | meas | calc | Δ (ppm) |
|---|---|---|---|
| | | | |
| $[M+Na]^+$ | 275,1613 | 275,1617 | -1,4 |

**Figure S57. ¹H NMR spectrum of 8 measured at 700 MHz in CDCl₃**

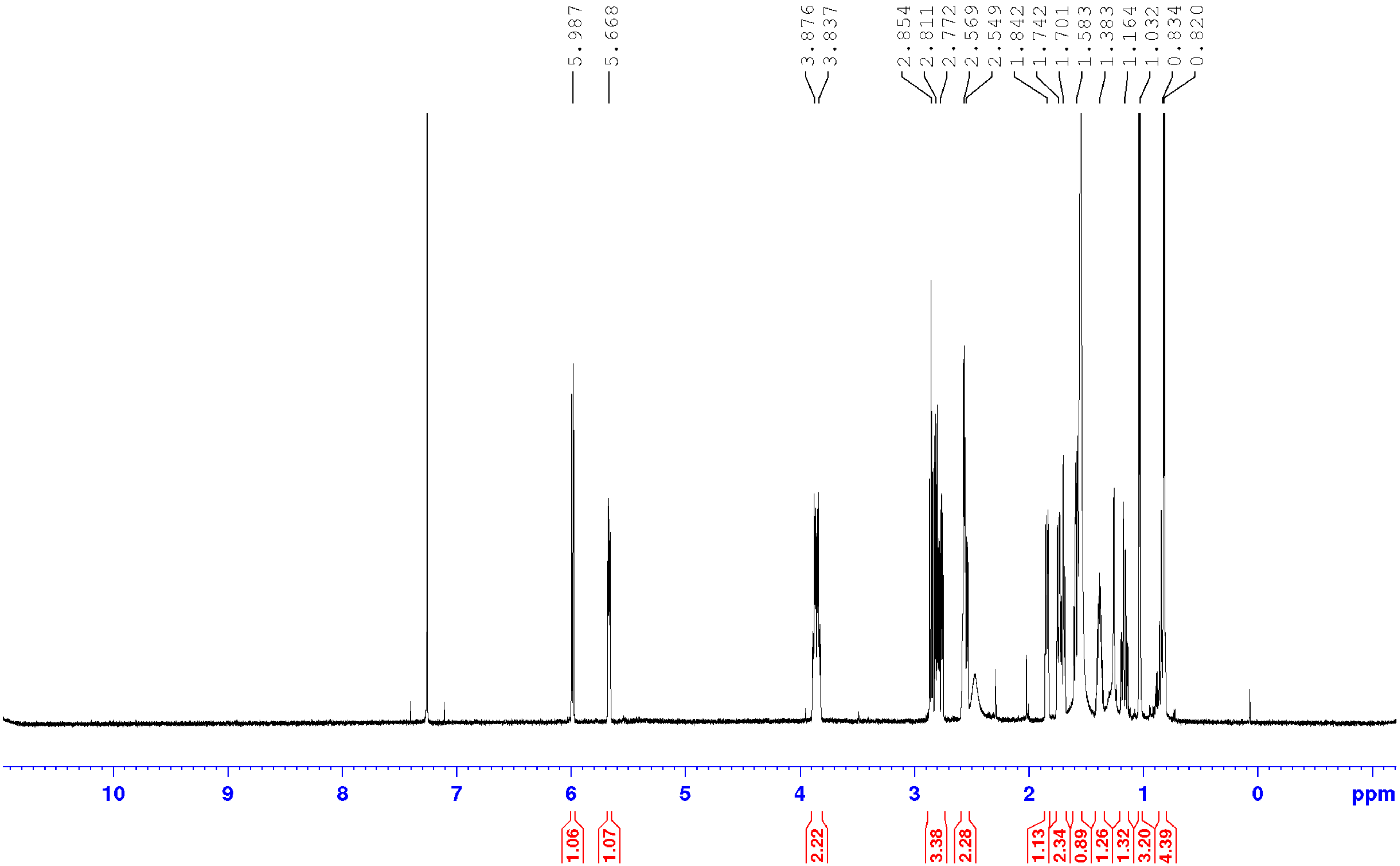

**Figure S58.**[13]**C NMR spectrum of 8 measured at 176 MHz in CDCl**$_3$

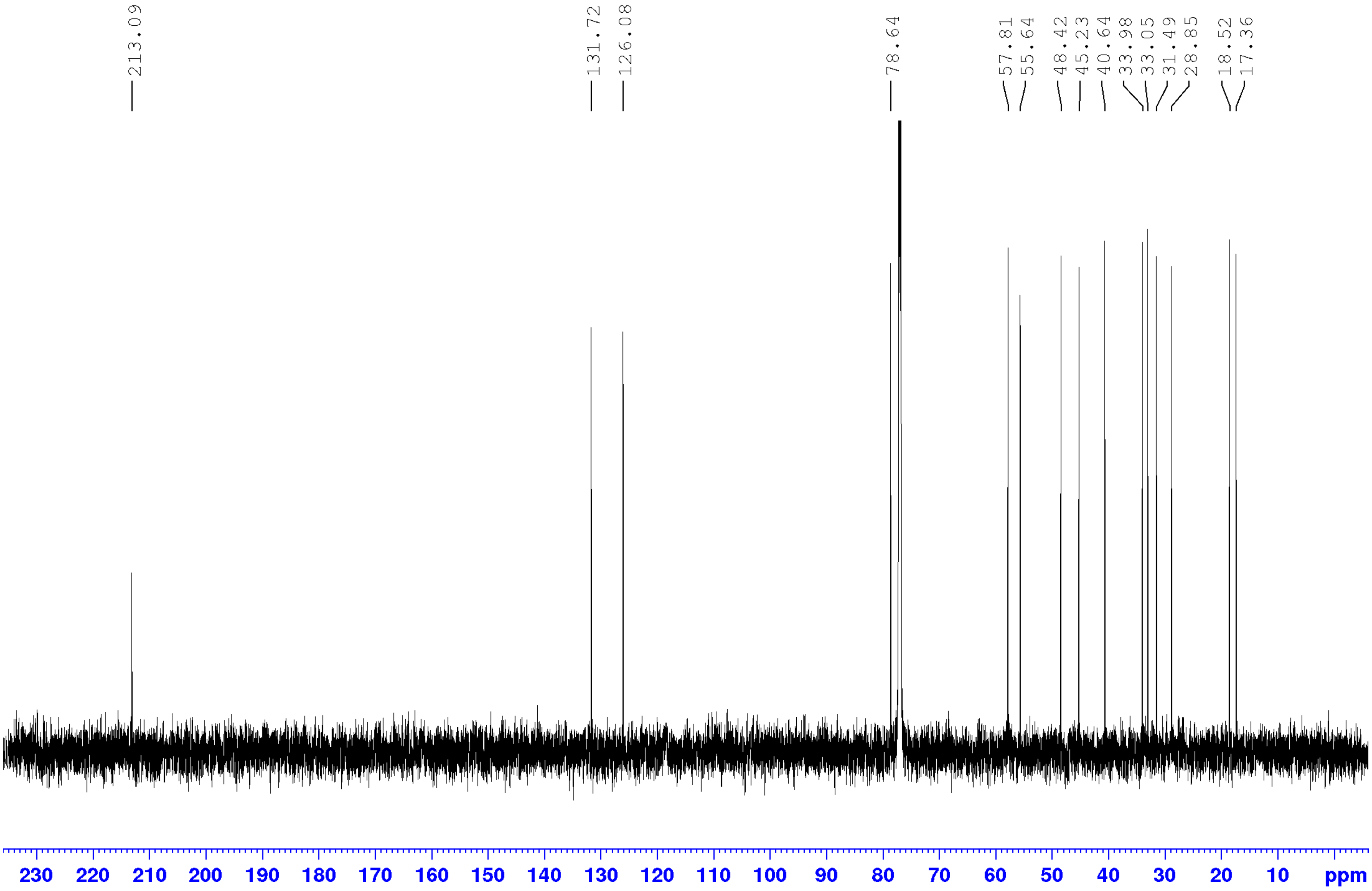

**Figure S59. DEPT-135 spectrum of 8 measured at 176 MHz in $CDCl_3$**

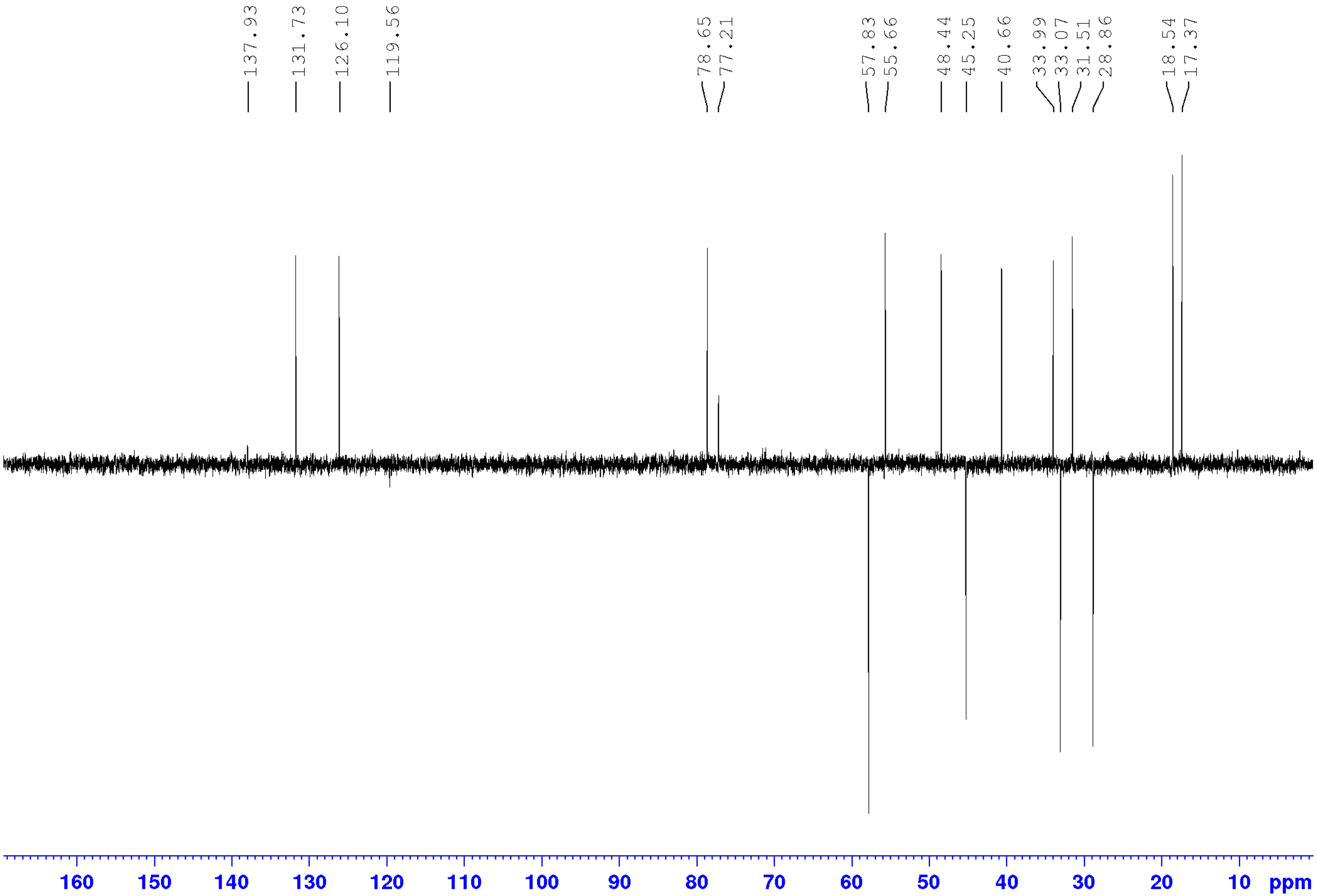

**Figure S60. HSQC spectrum of 8 measured in CDCl₃**

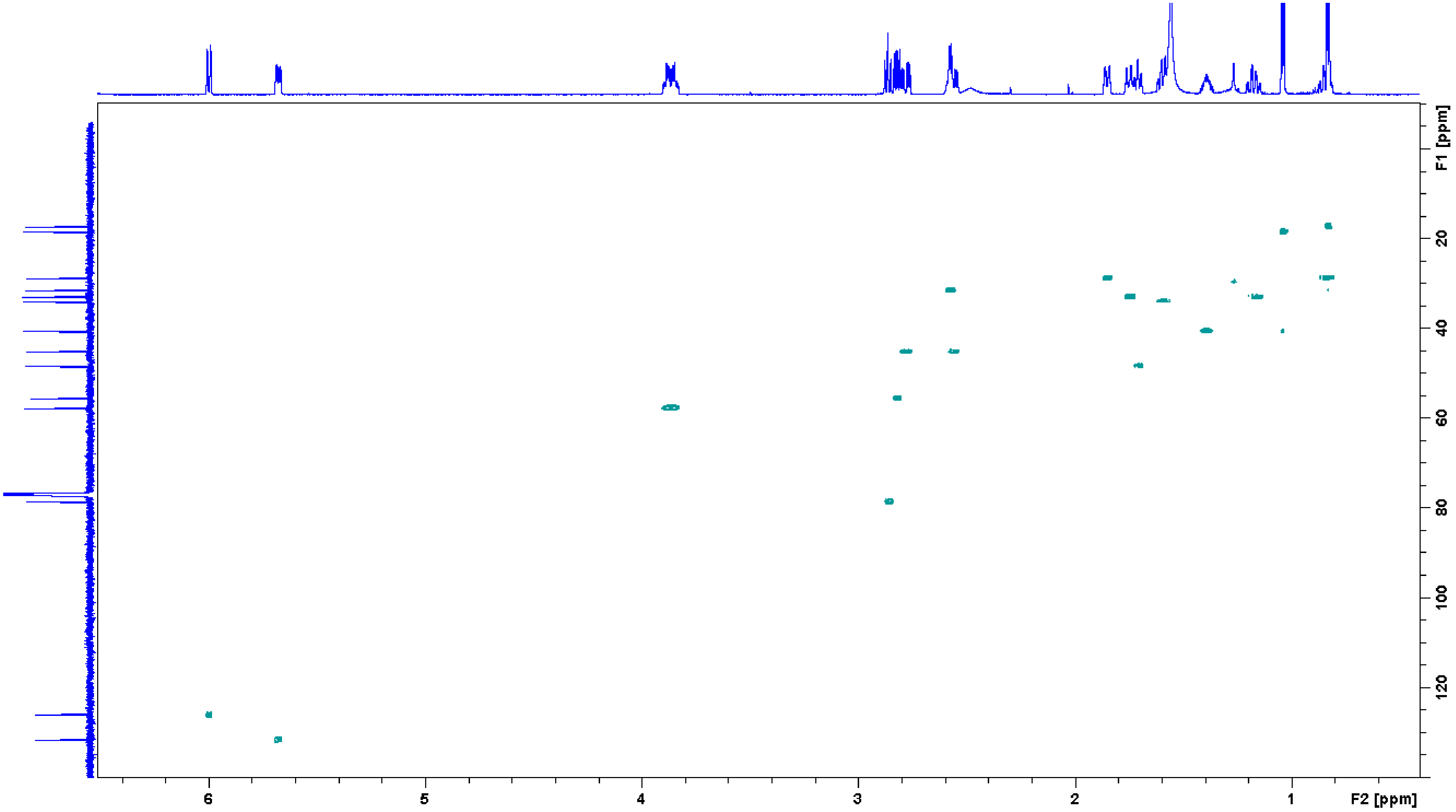

**Figure S61. COSY spectrum of 8 measured in CDCl₃**

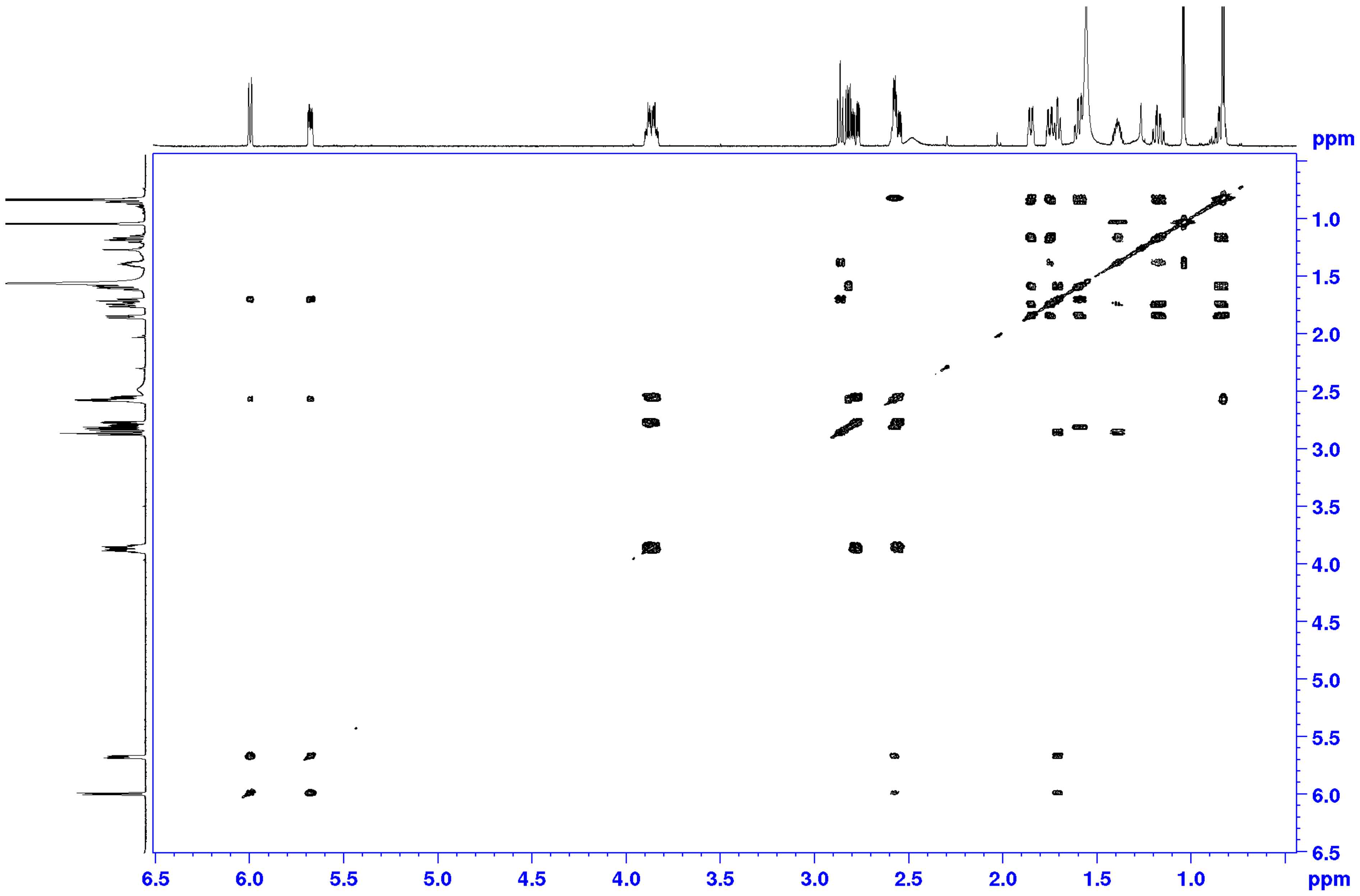

**Figure S62. HMBC spectrum of 8 measured in CDCl$_3$**

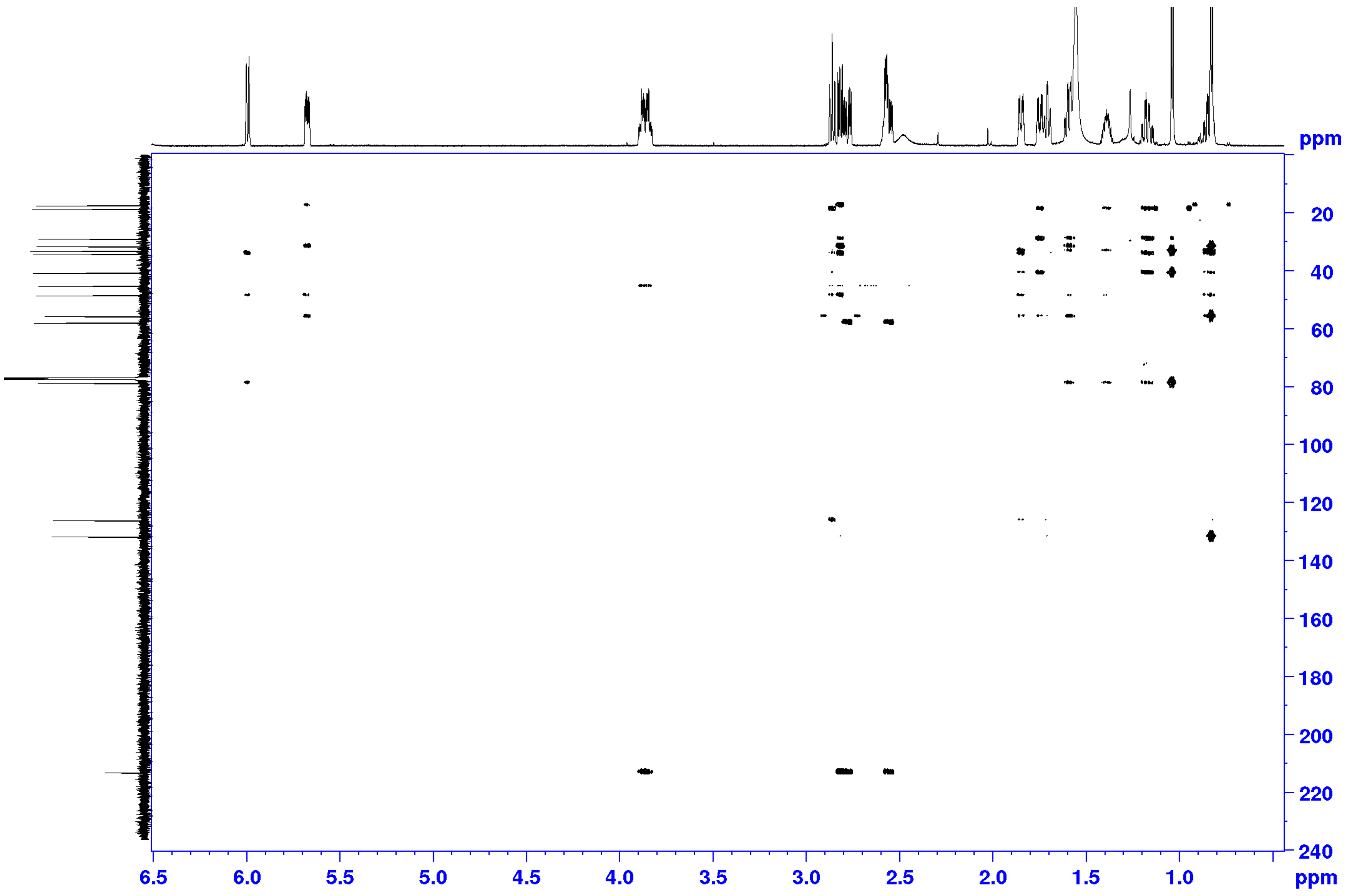

**Figure S63. ROESY spectrum of 8 measured in $CDCl_3$**

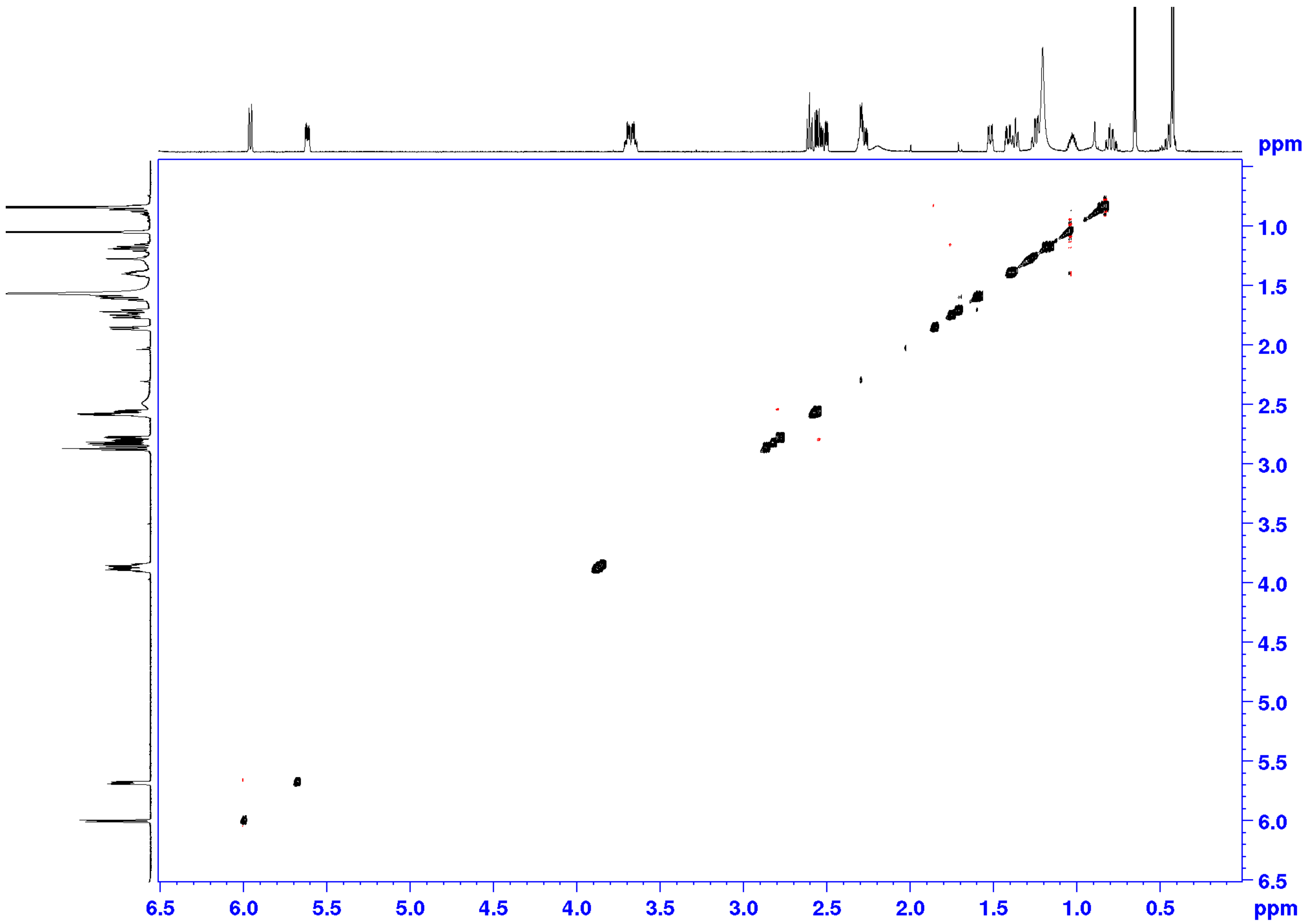

**Figure S64. HRESIMS for 9**

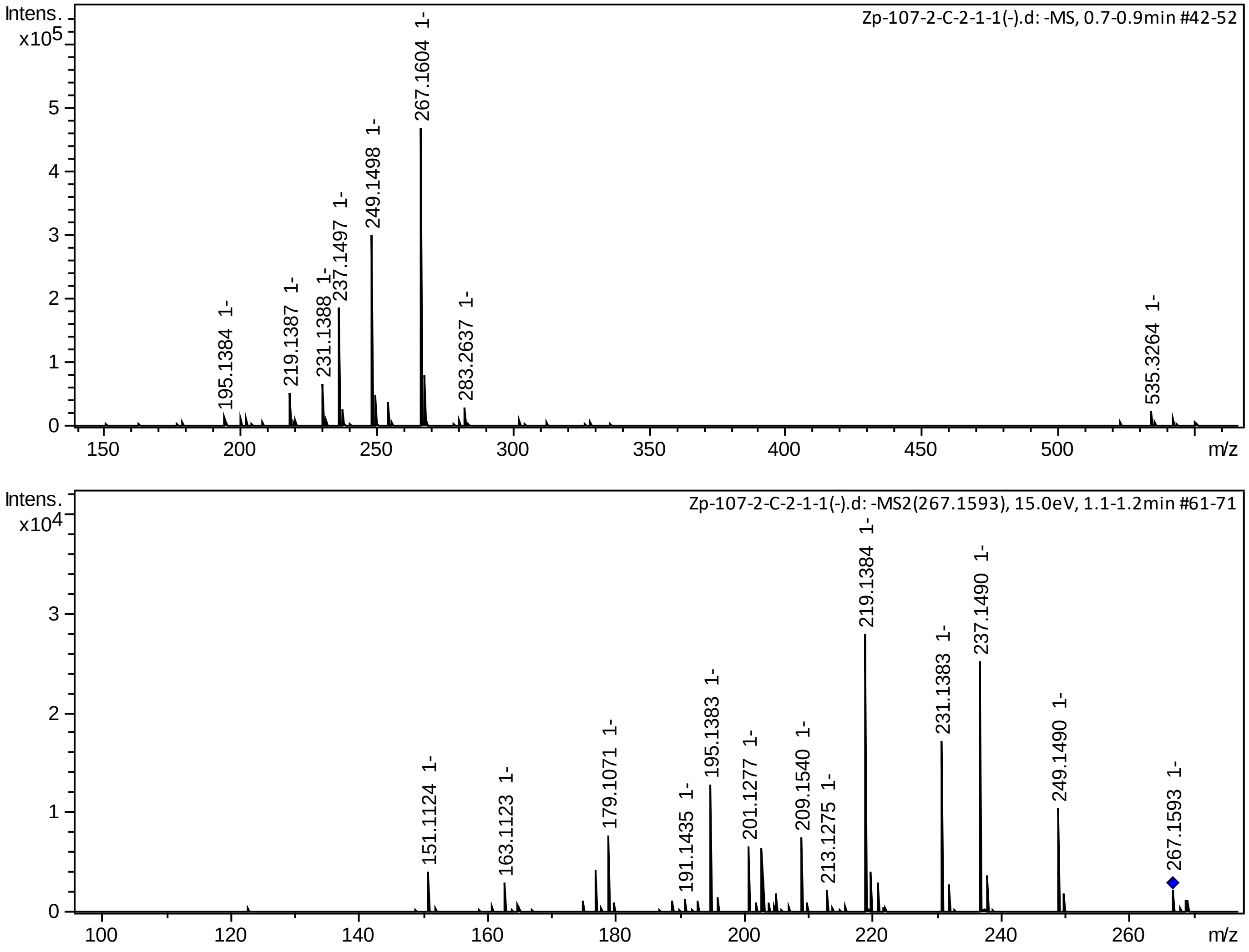

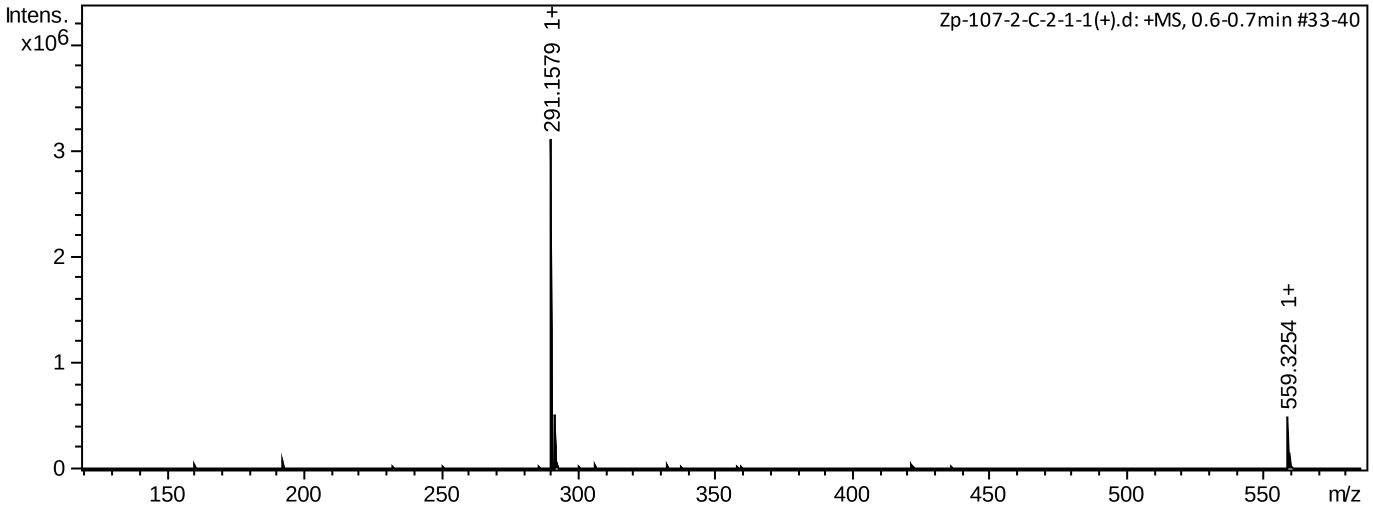


| | meas | calc | Δ (ppm) |
|---|---|---|---|
| $[M-H]^-$ | 267,1604 | 267,1602 | -0,7 |
| $[M+Na]^+$ | 291,1579 | 291,1567 | -4,1 |

**Figure S65.** ¹H NMR spectrum of 9 measured at 700 MHz in $CDCl_3$

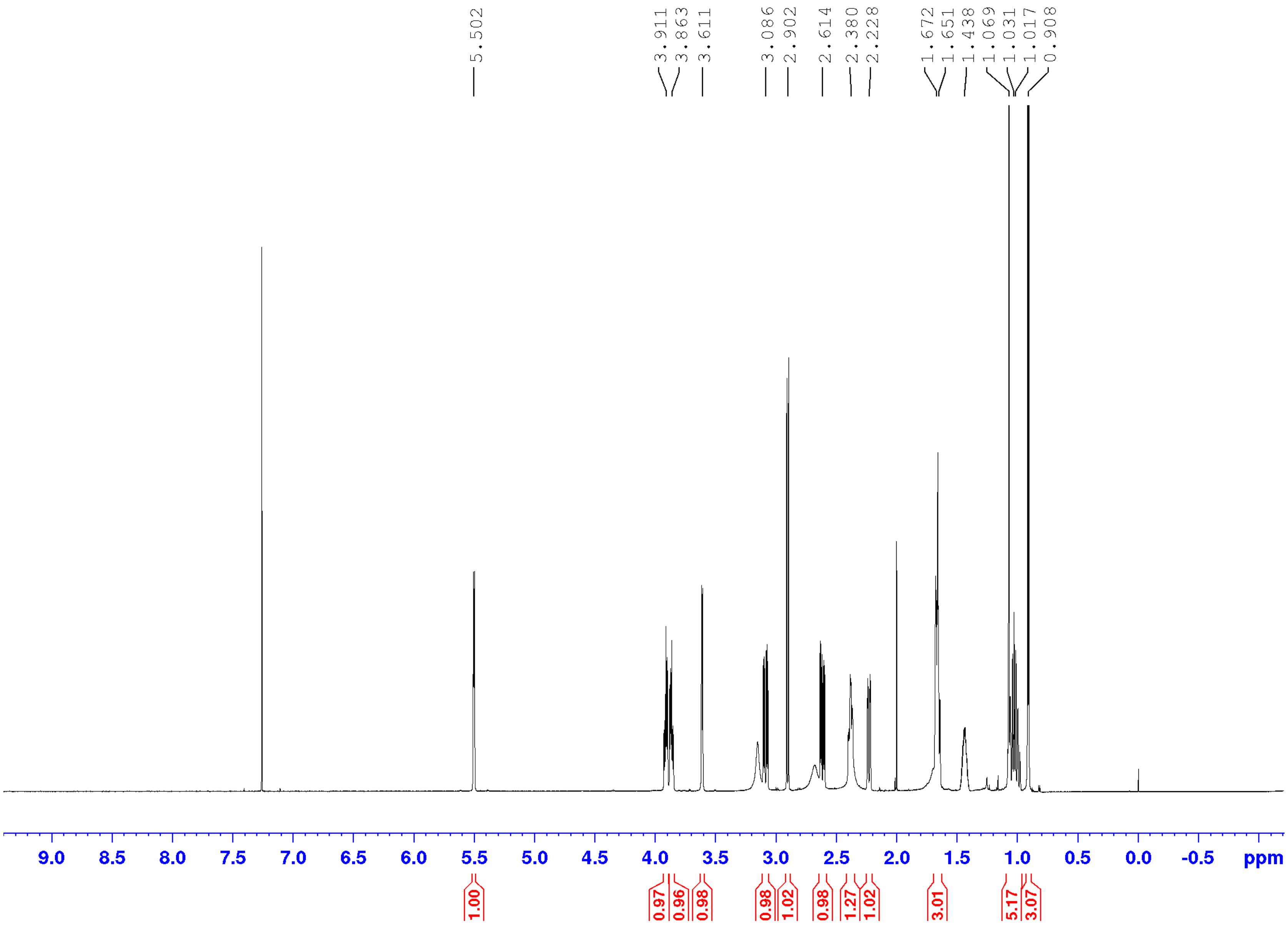

**Figure S66.$^{13}$C NMR spectrum of 9 measured at 176 MHz in $CDCl_3$**

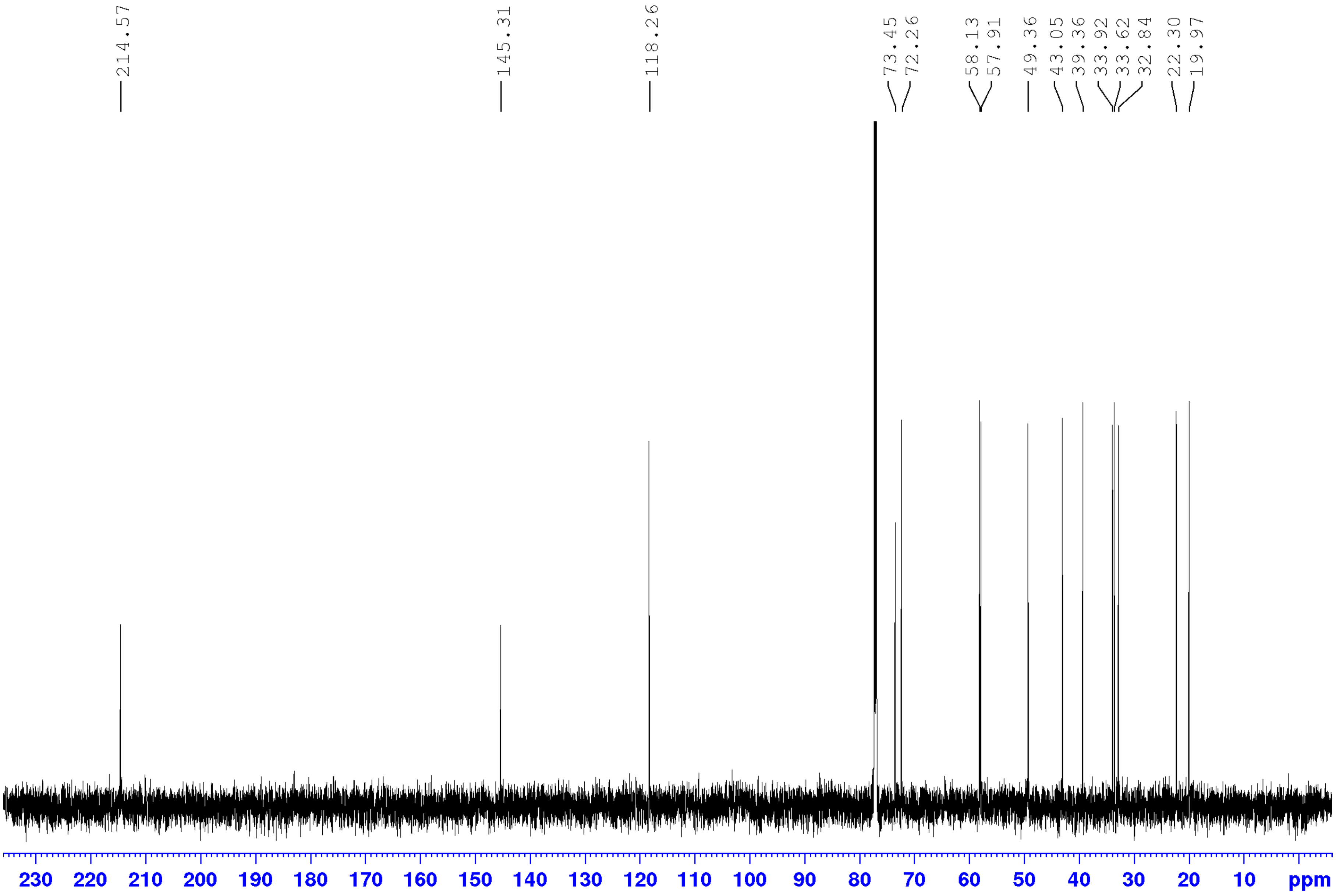

**Figure S67. DEPT-135 spectrum of 9 measured at 176 MHz in $CDCl_3$**

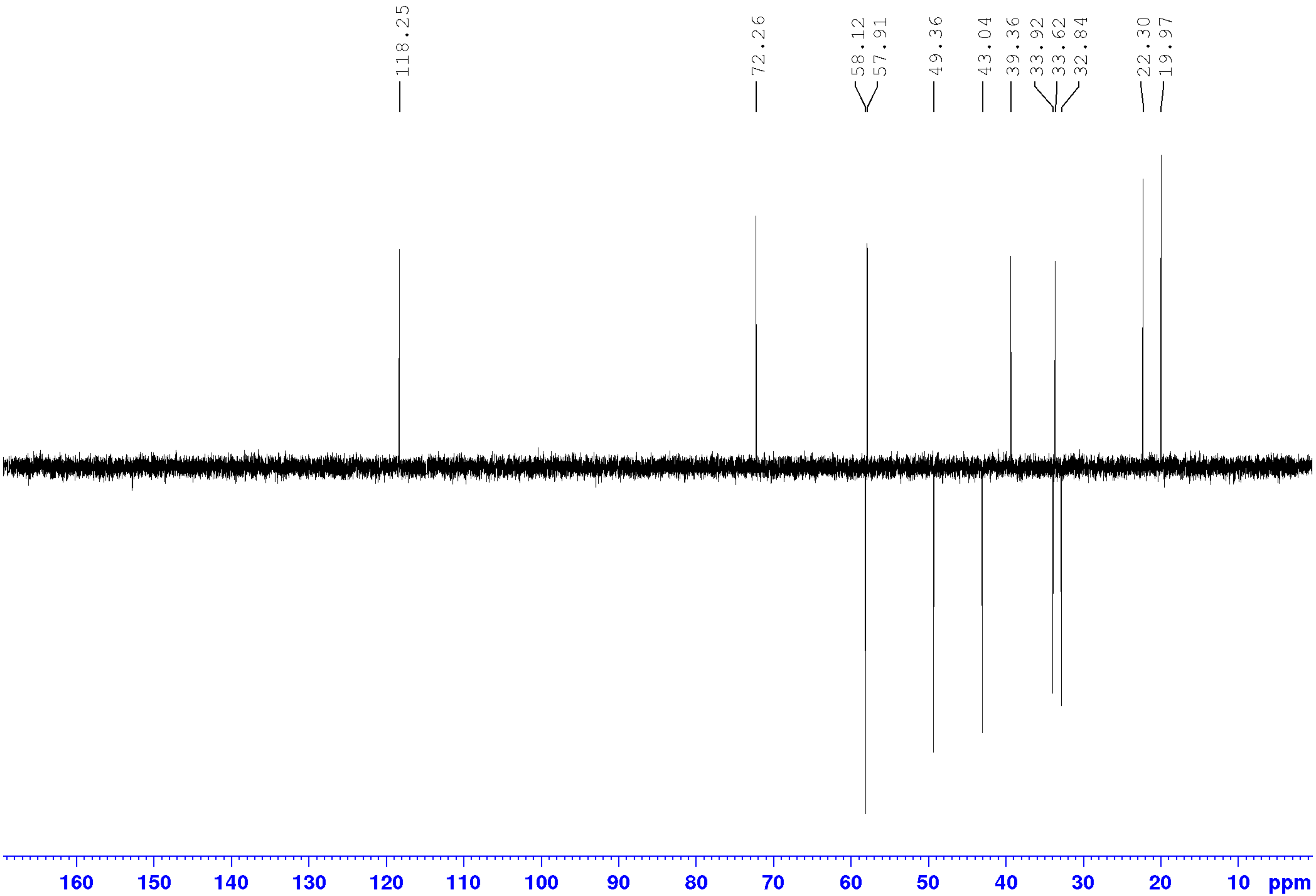

**Figure S68. HSQC spectrum of 9 measured in CDCl$_3$**

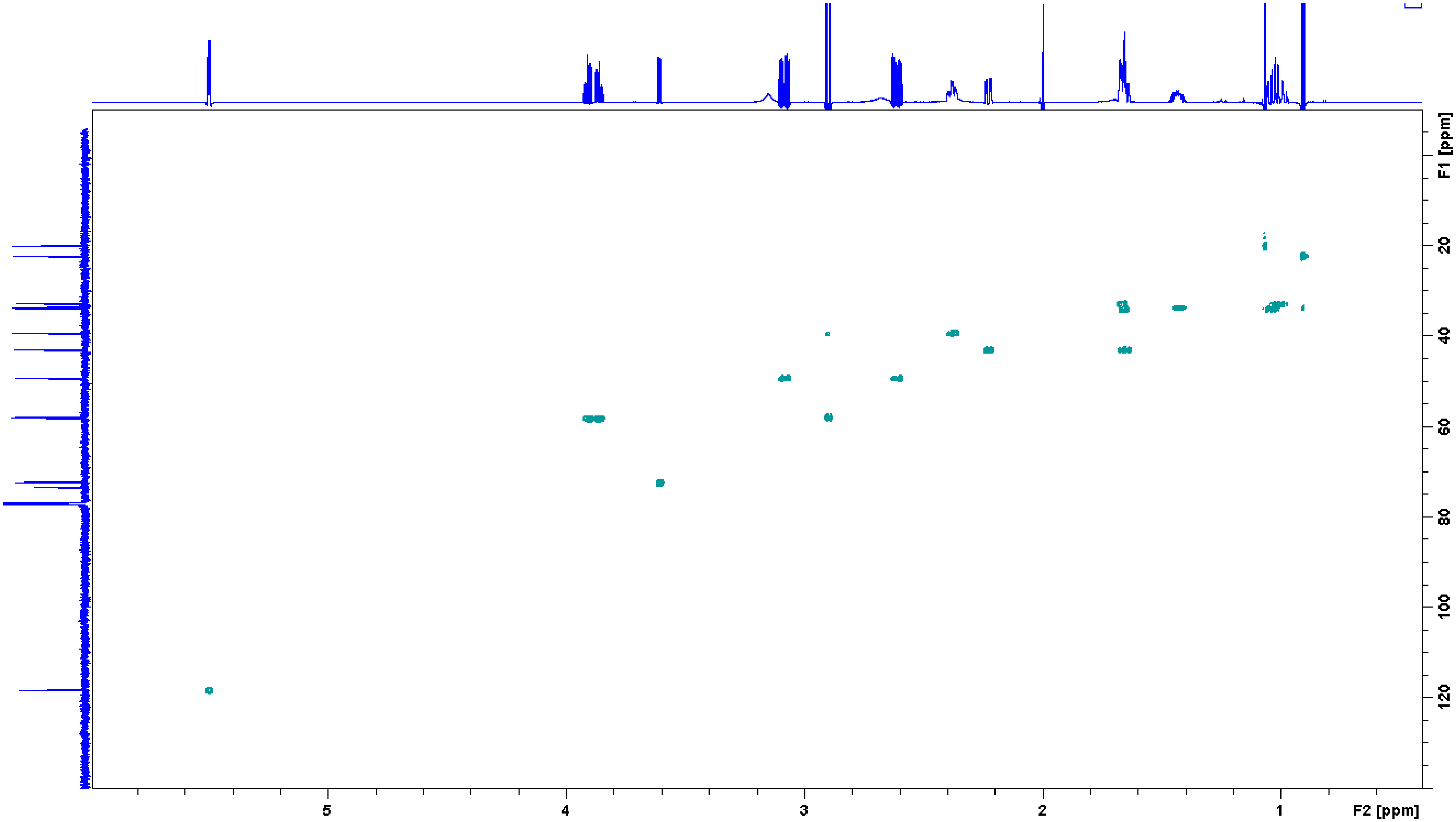

**Figure S69. COSY spectrum of 9 measured in $CDCl_3$**

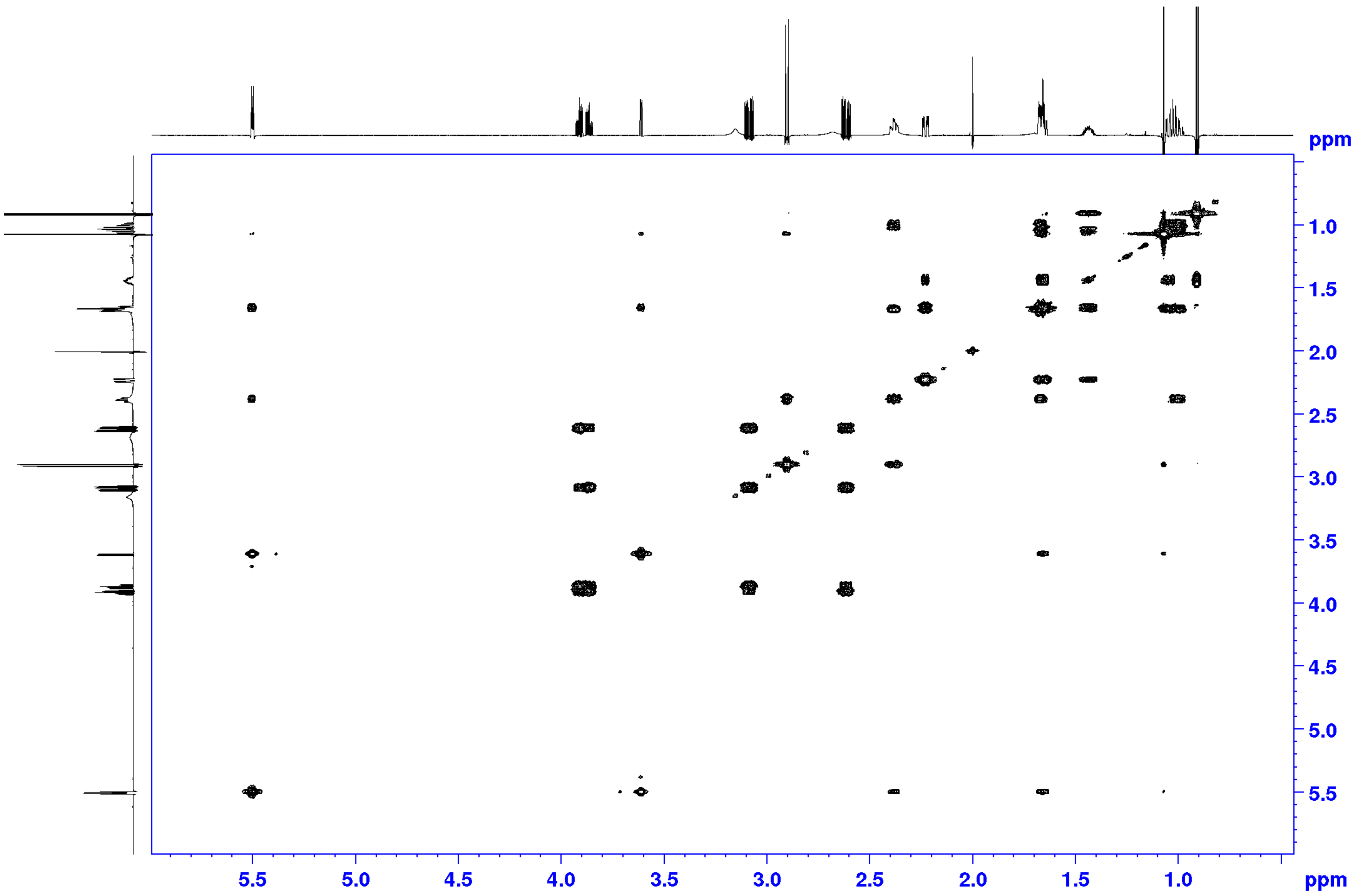

**Figure S70. HMBC spectrum of 9 measured in $CDCl_3$**

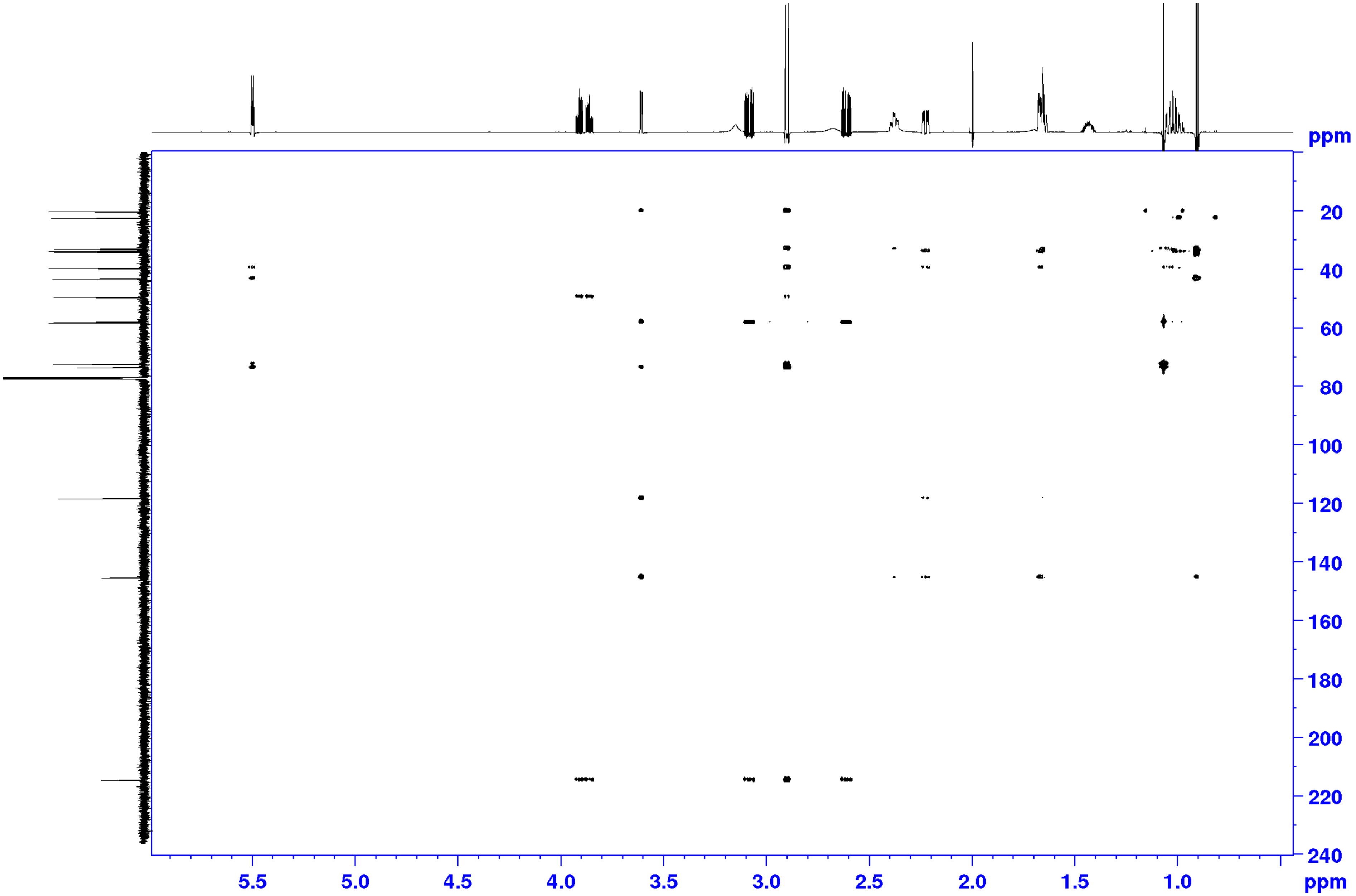

**Figure S71. ROESY spectrum of 9 measured in CDCl$_3$**

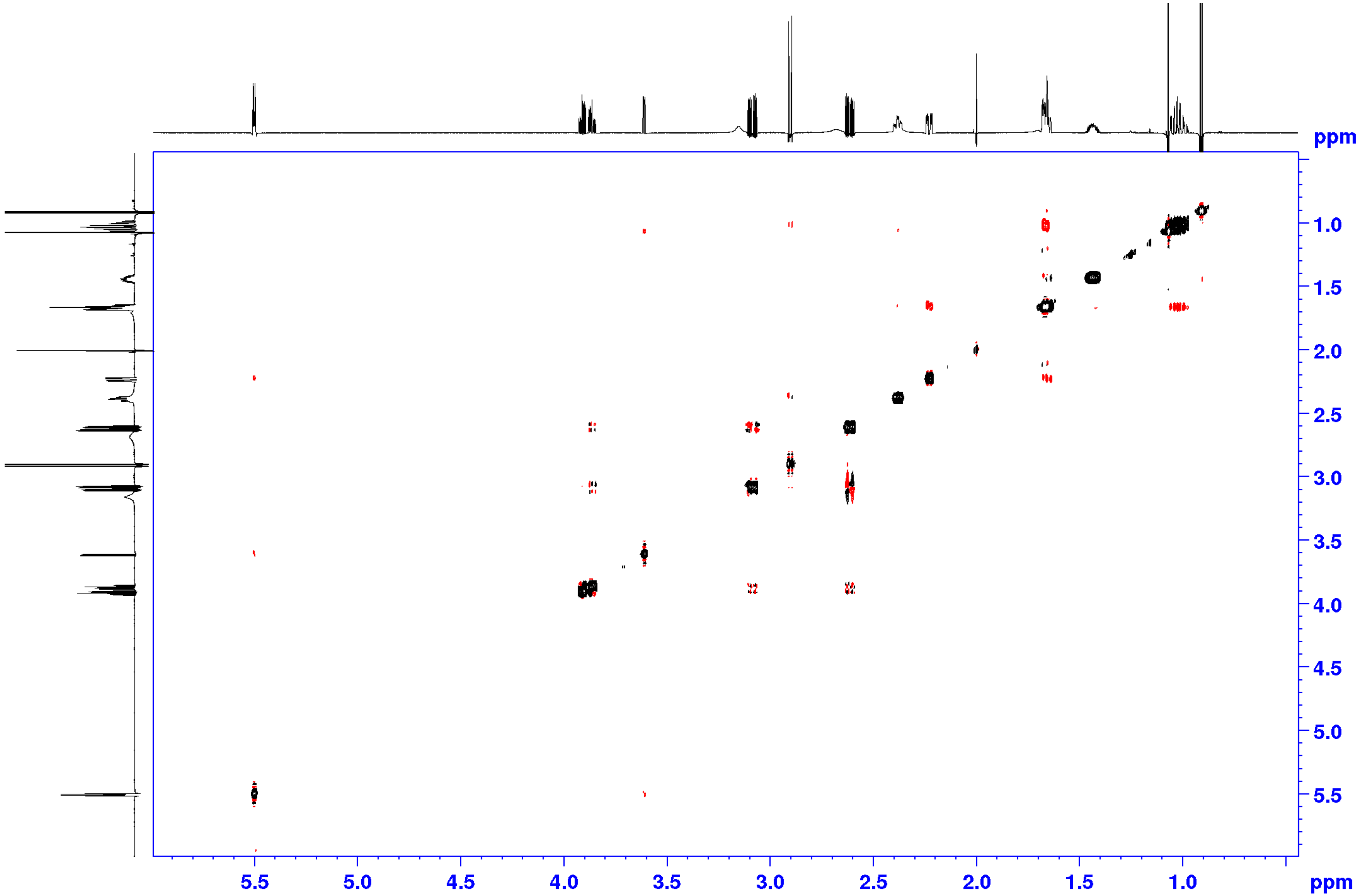


**Figure S72. HRESIMS for 10**

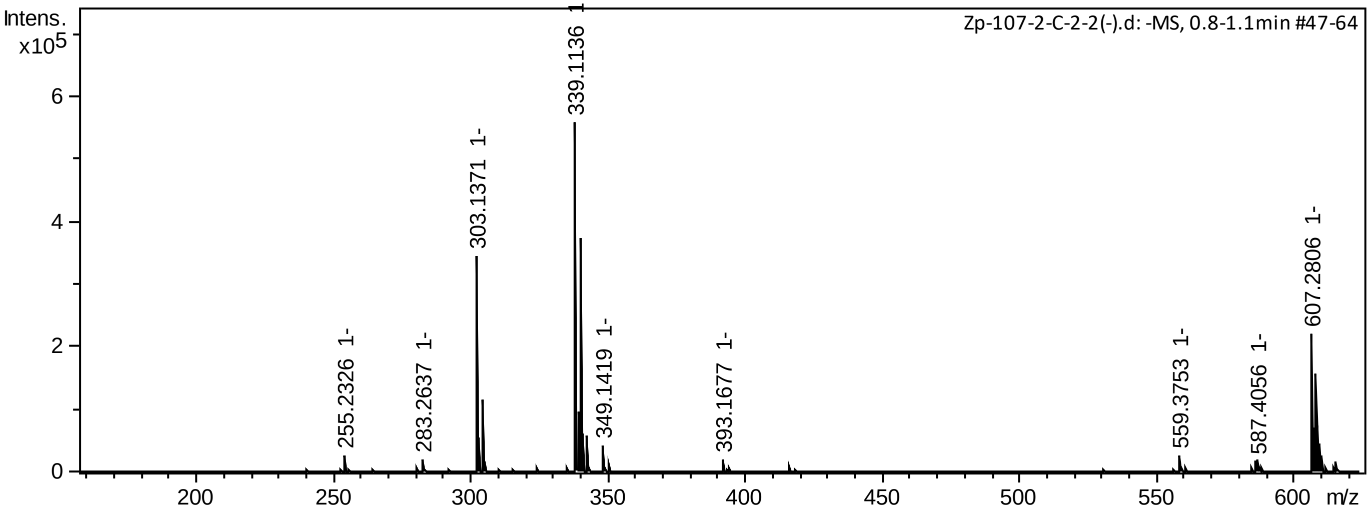
Intens.
x10^5
Zp-107-2-C-2-2(-).d: -MS, 0.8-1.1min #47-64
339.1136 1-
303.1371 1-
255.2326 1-
283.2637 1-
349.1419 1-
393.1677 1-
559.3753 1-
587.4056 1-
607.2806 1-
m/z

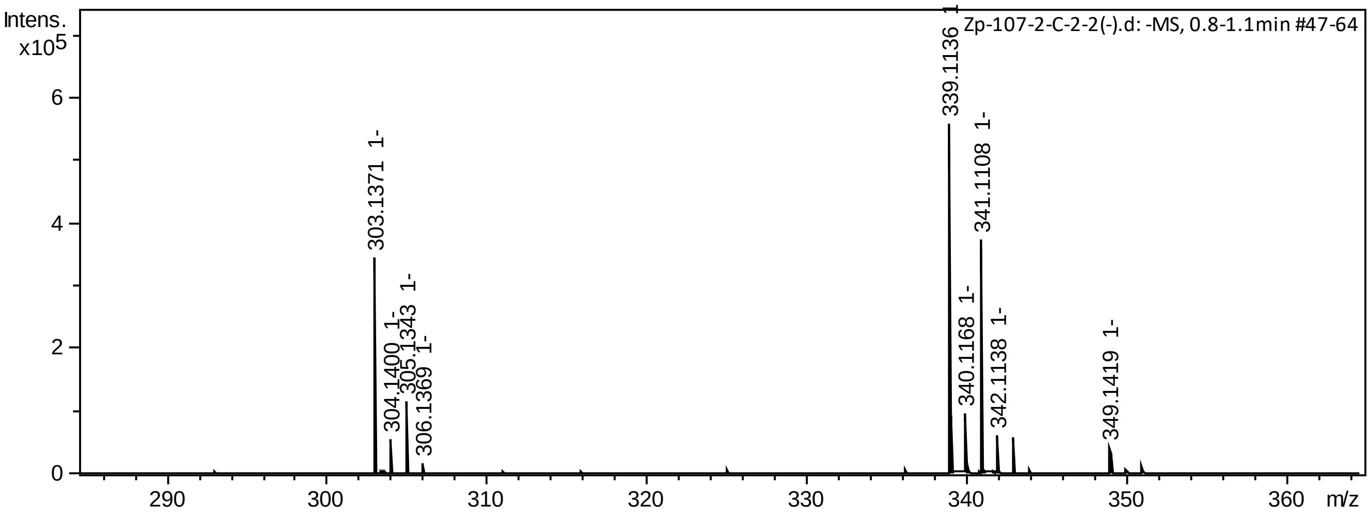
Intens.
x10^5
Zp-107-2-C-2-2(-).d: -MS, 0.8-1.1min #47-64
339.1136 1
303.1371 1-
341.1108 1-
340.1168 1-
342.1138 1-
304.1400 1-
305.1343 1-
306.1369 1-
349.1419 1-
m/z

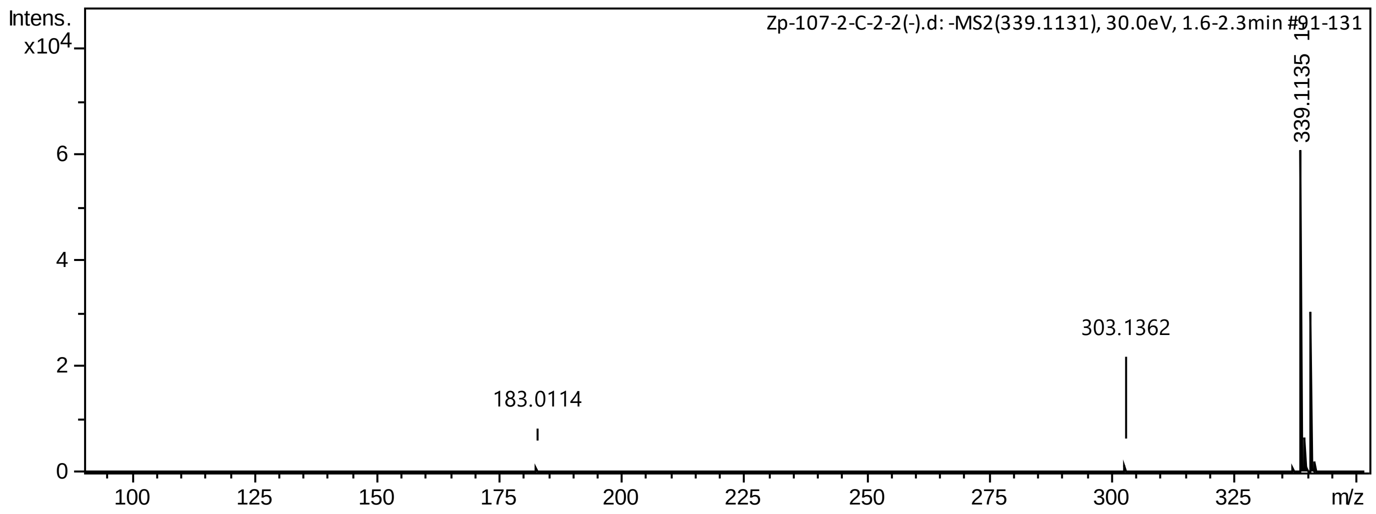
Intens.
x10^4
Zp-107-2-C-2-2(-).d: -MS2(339.1131), 30.0eV, 1.6-2.3min #91-131
339.1135 1-
303.1362
183.0114
m/z

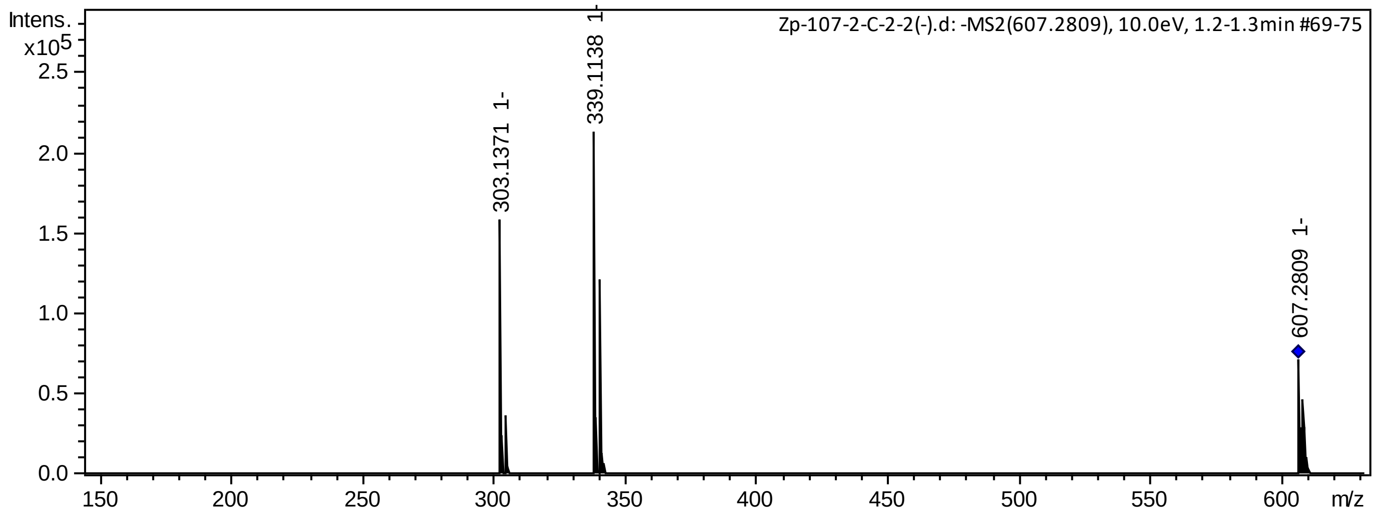
Intens.
x10^5
Zp-107-2-C-2-2(-).d: -MS2(607.2809), 10.0eV, 1.2-1.3min #69-75
303.1371 1-
339.1138 1-
607.2809 1-
m/z

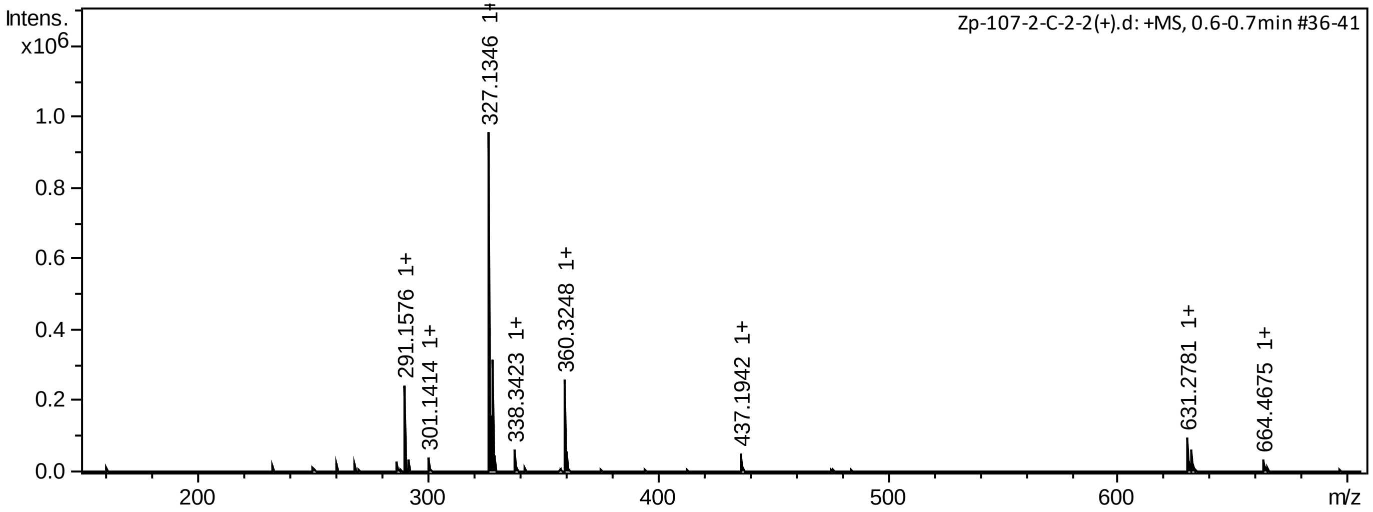
Intens.
x10^6
Zp-107-2-C-2-2(+).d: +MS, 0.6-0.7min #36-41
327.1346 1+
291.1576 1+
301.1414 1+
338.3423 1+
360.3248 1+
437.1942 1+
631.2781 1+
664.4675 1+
m/z

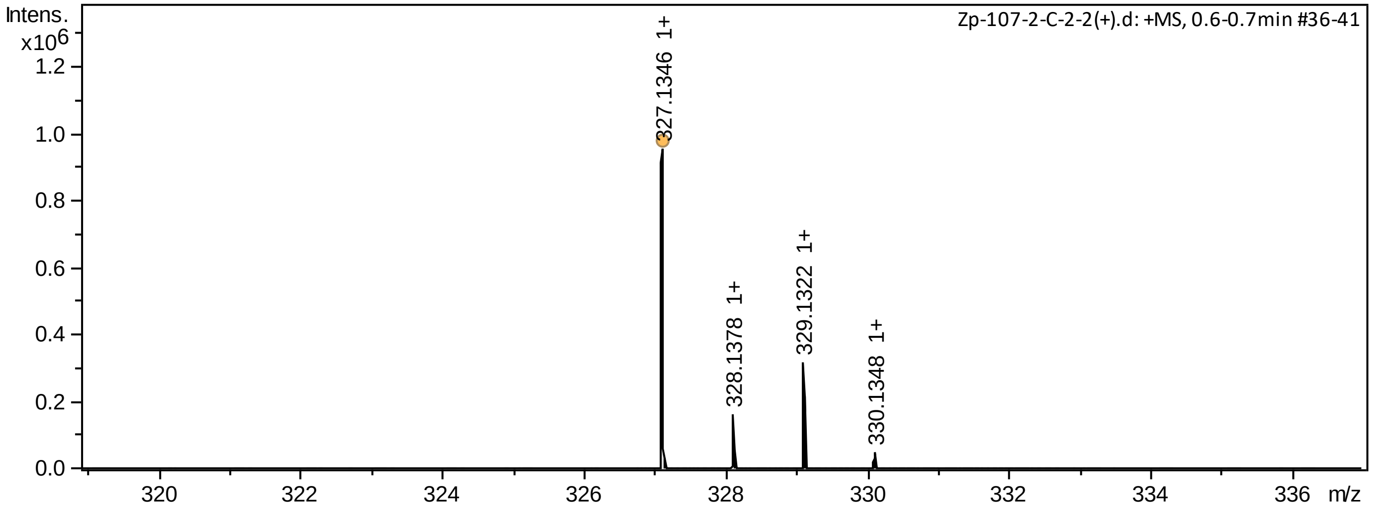
Intens.
x10^6
Zp-107-2-C-2-2(+).d: +MS, 0.6-0.7min #36-41
327.1346 1+
328.1378 1+
329.1322 1+
330.1348 1+
m/z

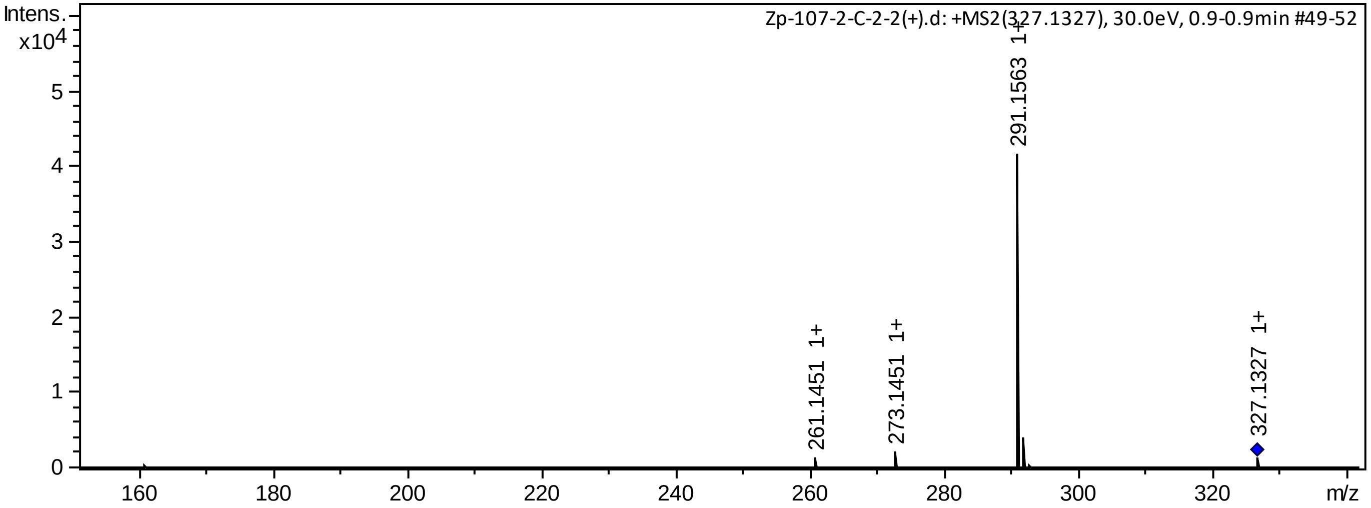


| | meas | calc | Δ (ppm) |
|---|---|---|---|
| $[M-H]^-$ | 303,1371 | 303,1369 | -0,7 |
| $[M+Na]^+$ | 327,1346 | 327,1334 | -3,7 |

**Figure S73. $^1H$ NMR spectrum of 10 measured at 700 MHz in $CDCl_3$**

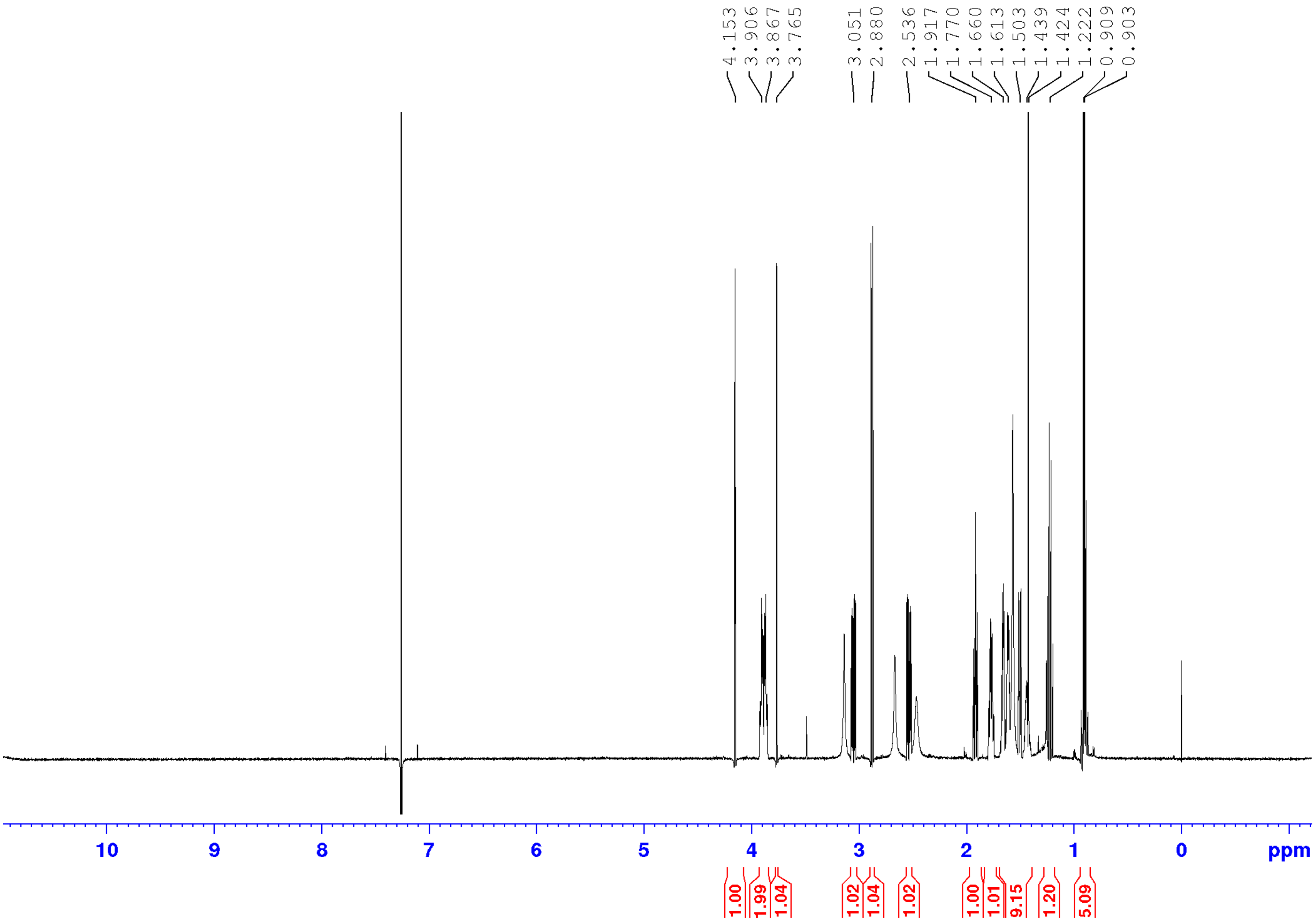

**Figure S74.** $^{13}C$ **NMR spectrum of 10 measured at 176 MHz in** $CDCl_3$

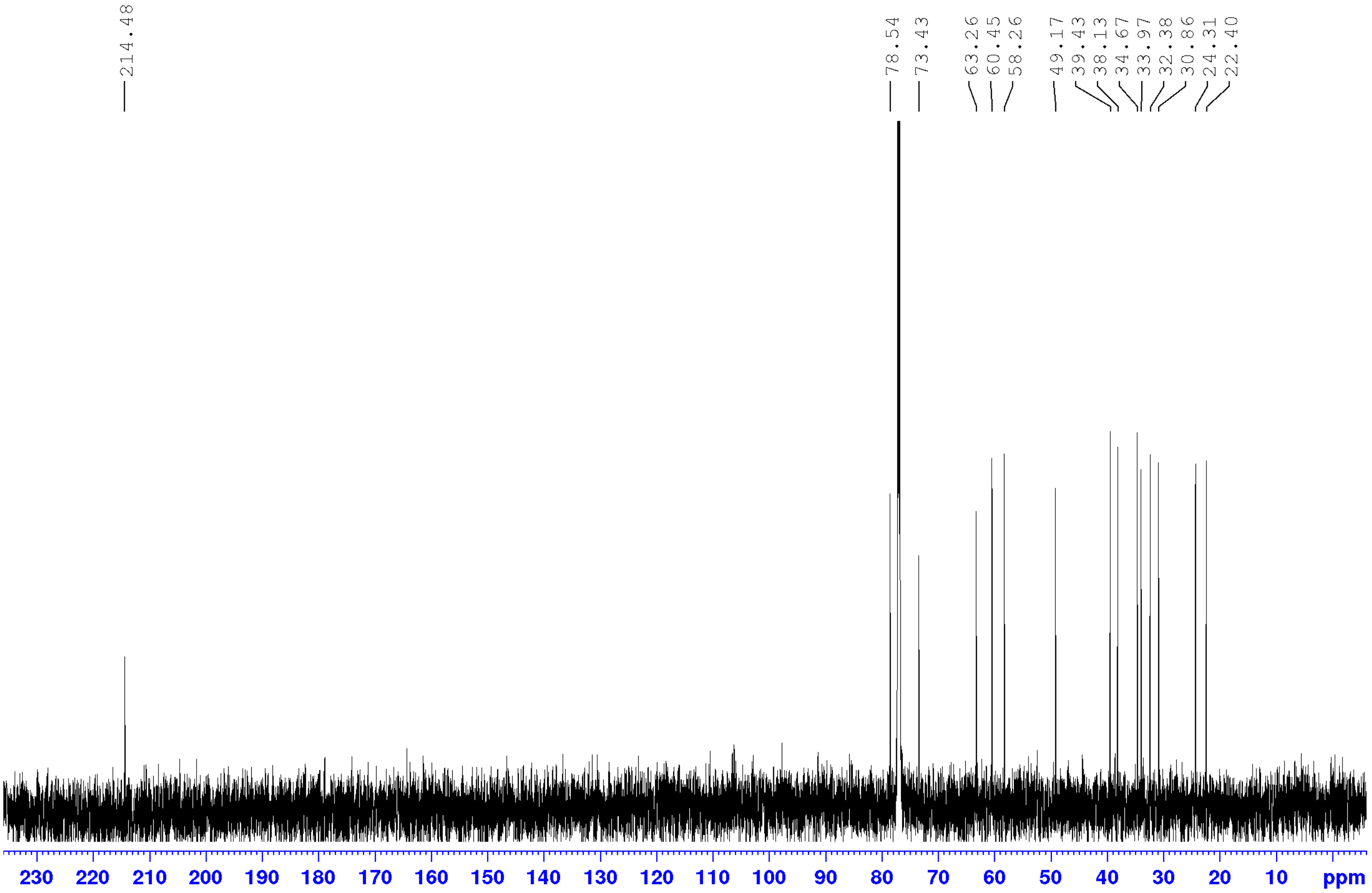

**Figure S75. DEPT-135 spectrum of 10 measured at 176 MHz in $CDCl_3$**

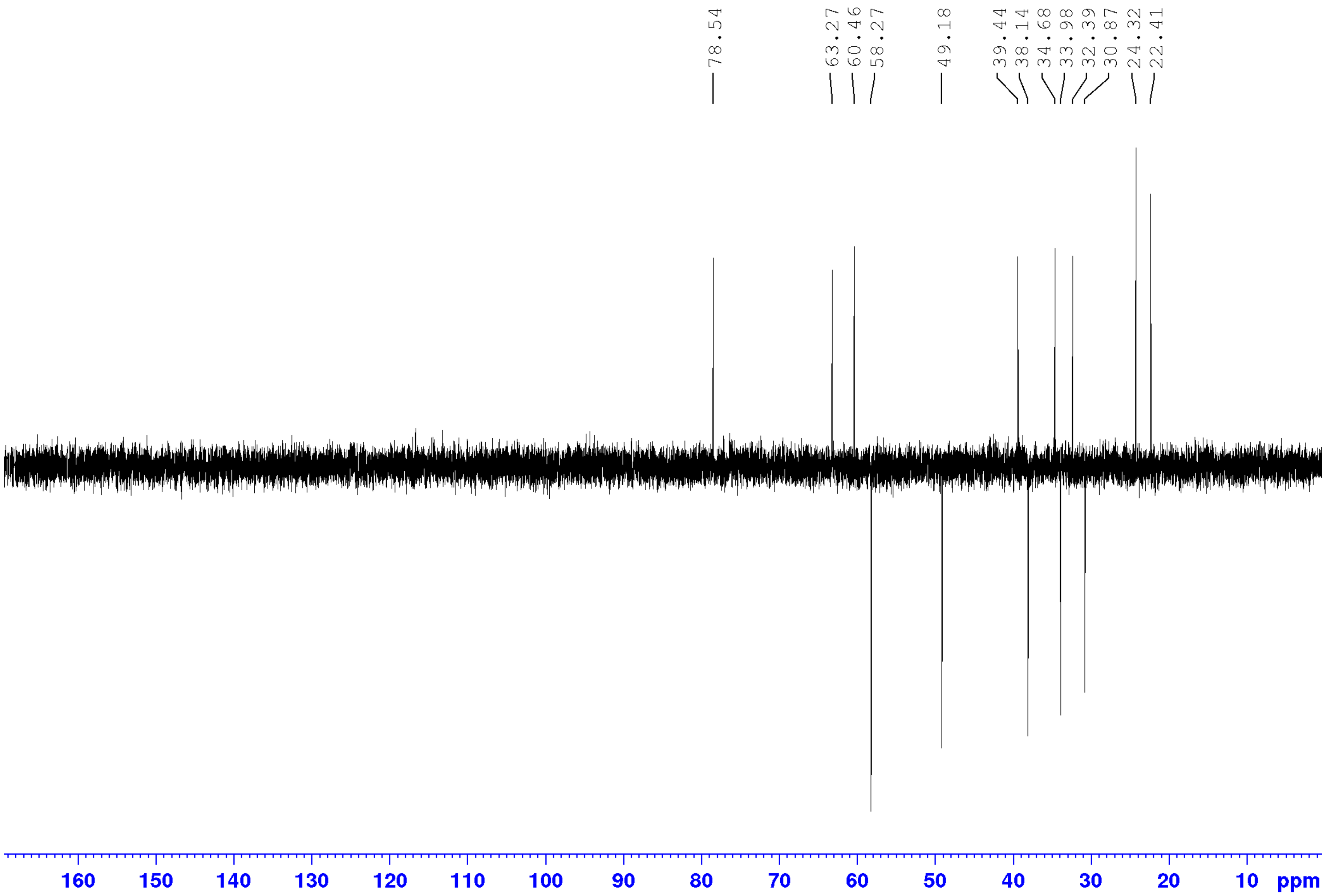

**Figure S76. HSQC spectrum of 10 measured in CDCl₃**

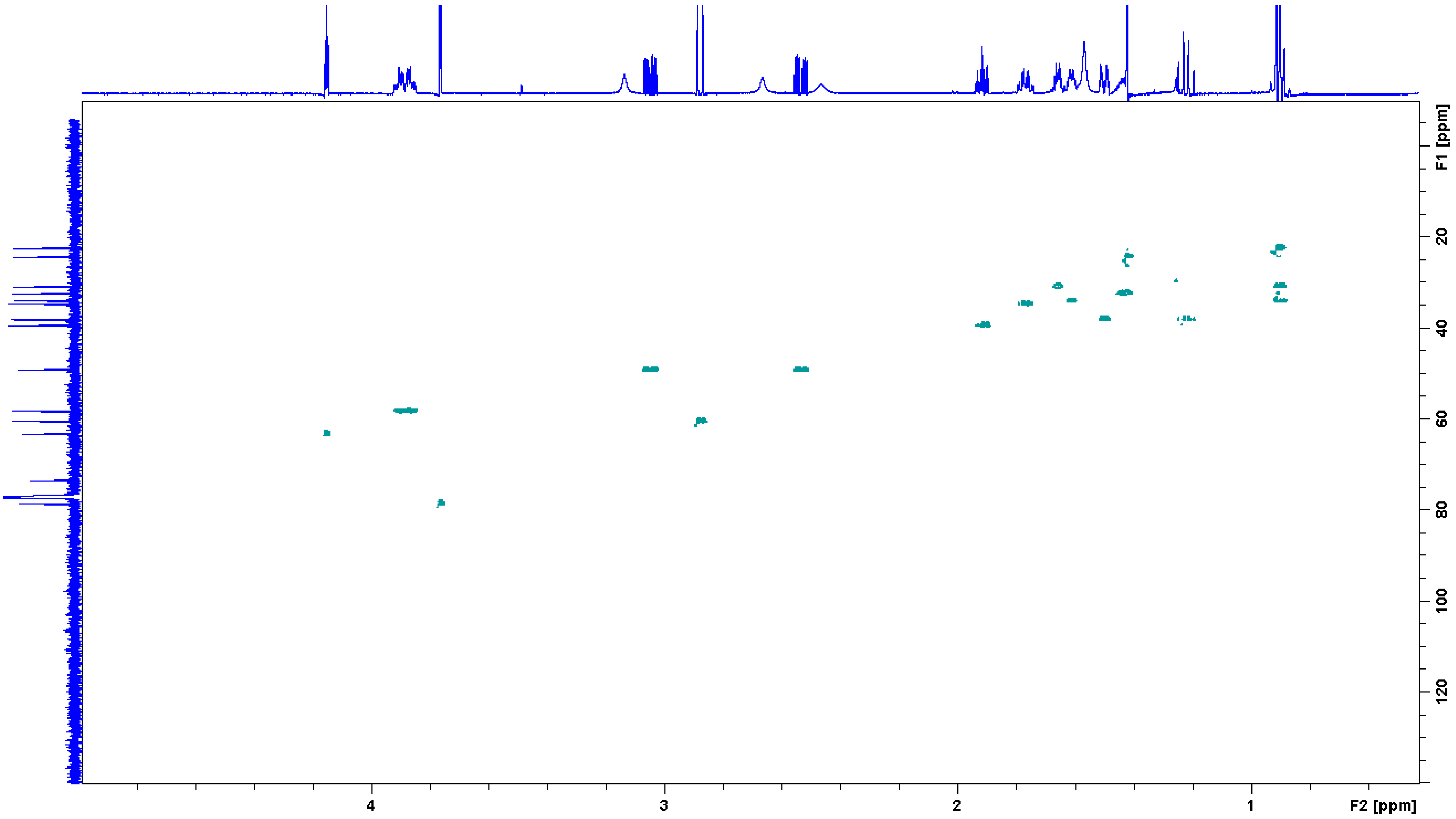

**Figure S77. COSY spectrum of 10 measured in $CDCl_3$**

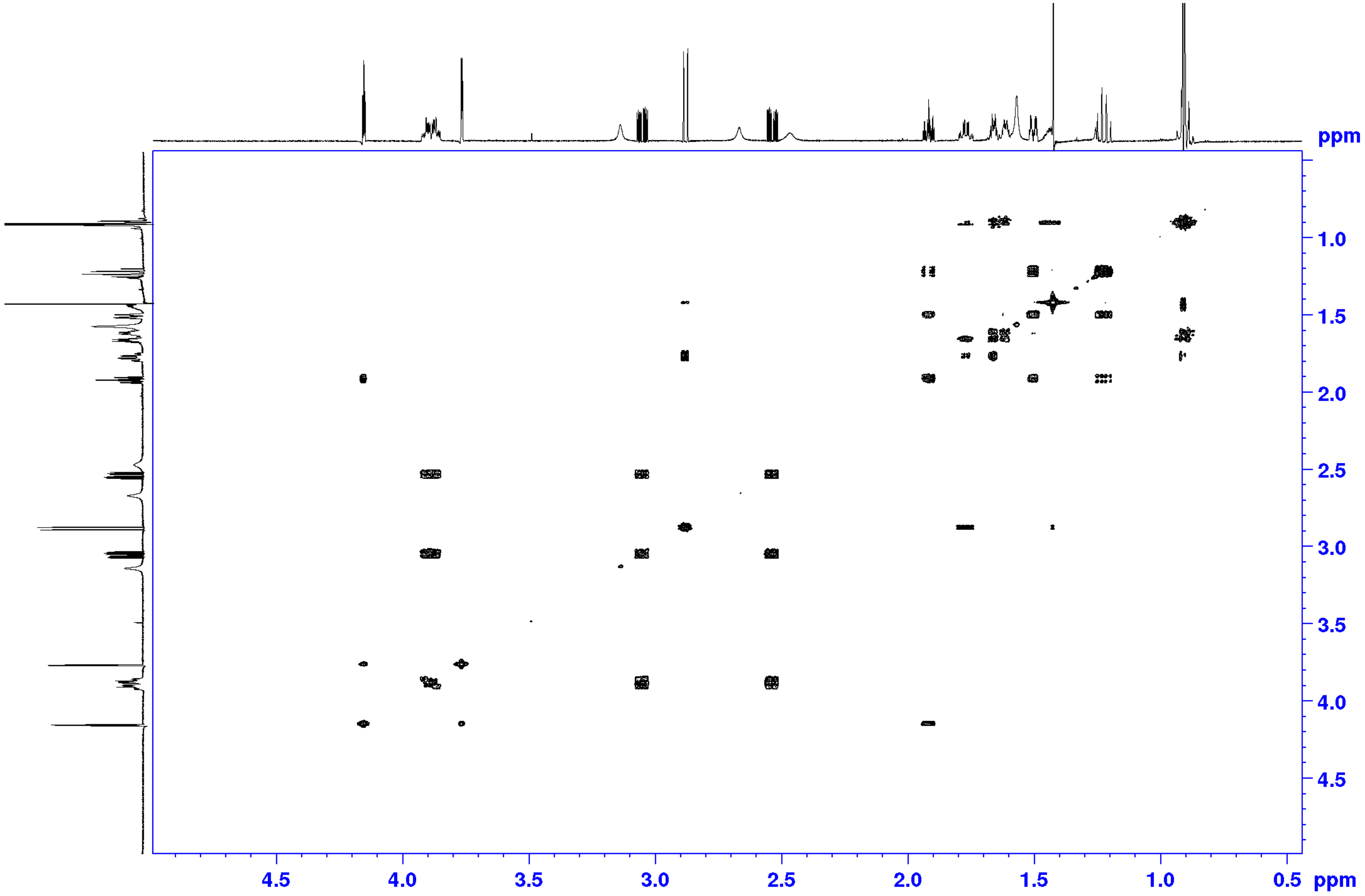

**Figure S78. HMBC spectrum of 10 measured in CDCl$_3$**

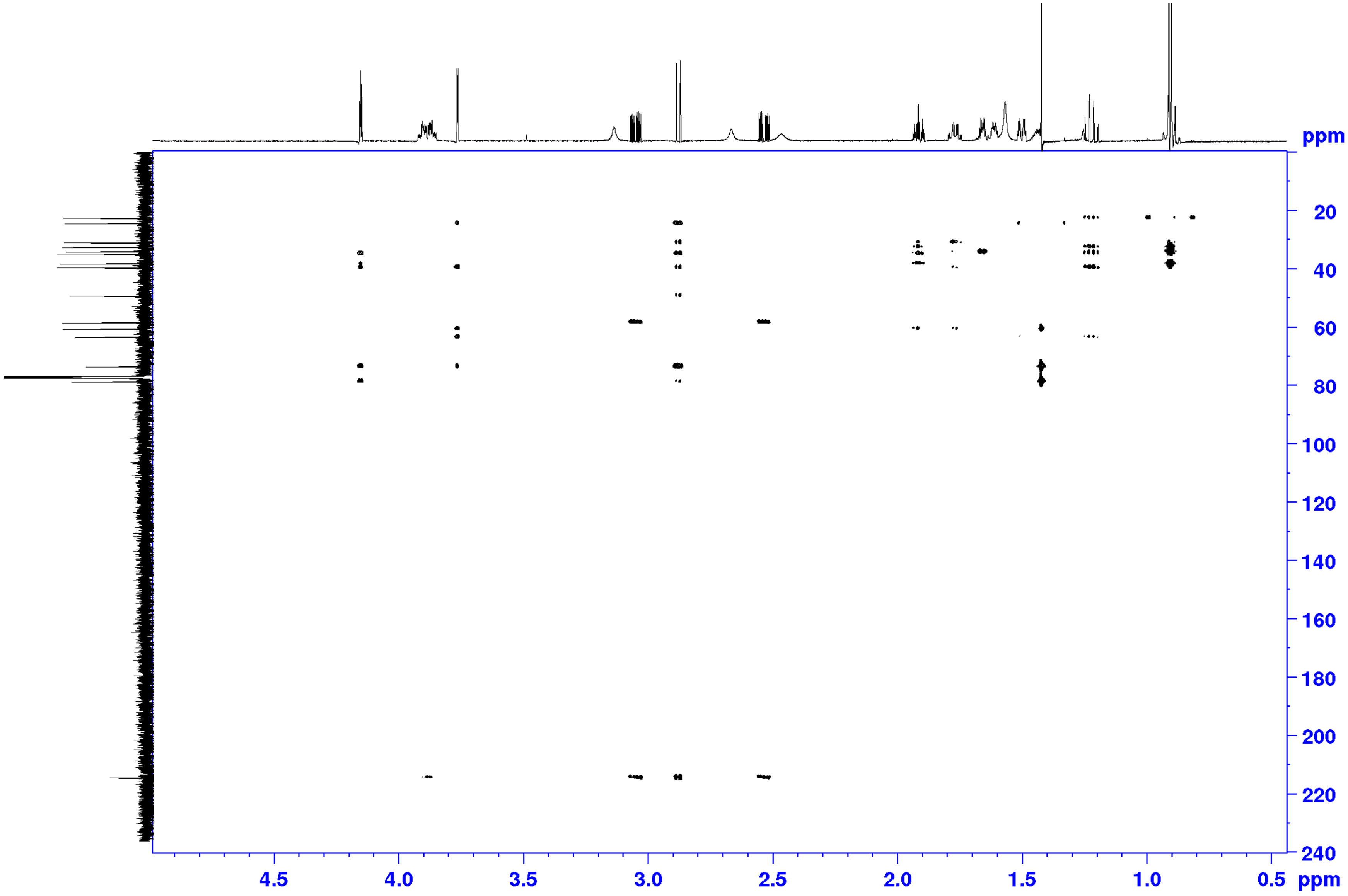

**Figure S79. ROESY spectrum of 10 measured in $CDCl_3$**

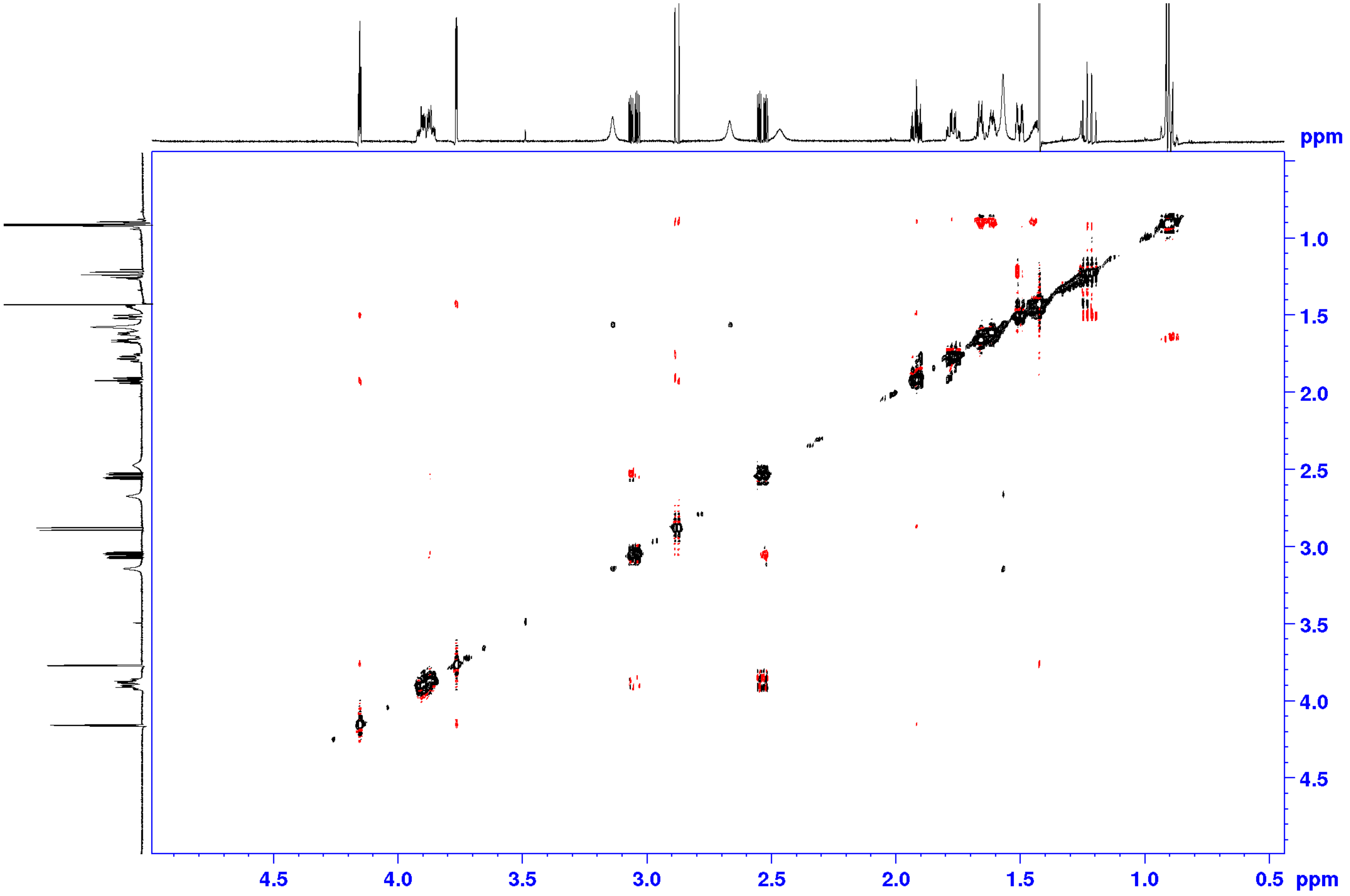

**Figure S80. HRESIMS for 11**

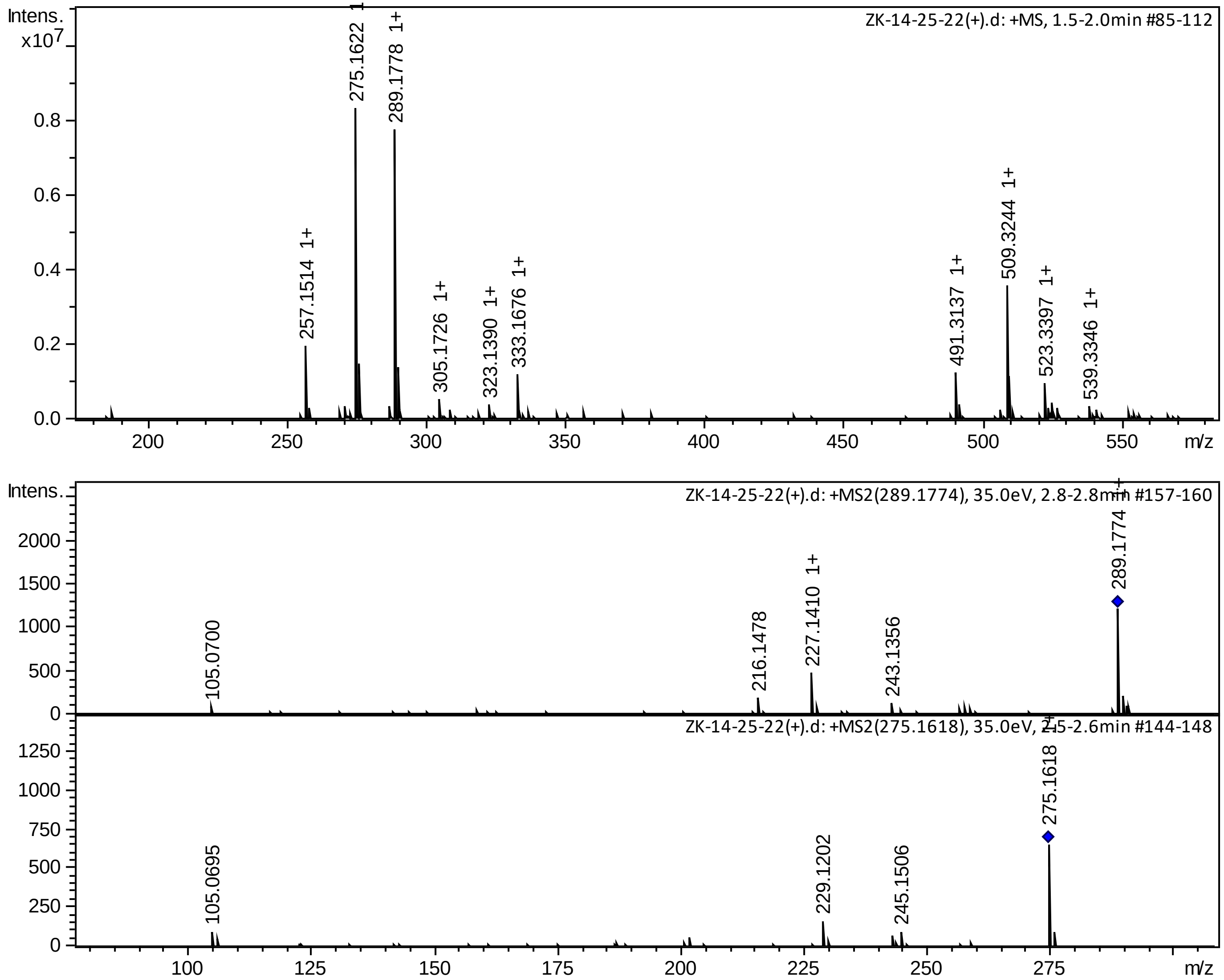


| | meas | calc | Δ (ppm) |
|---|---|---|---|
| $[M+Na]^+$ | 275,1622 | 257,1618 | 1,45 |

**Figure S81.** $^{1}H$ **NMR spectrum of 11 measured at 700 MHz in** $CDCl_3$

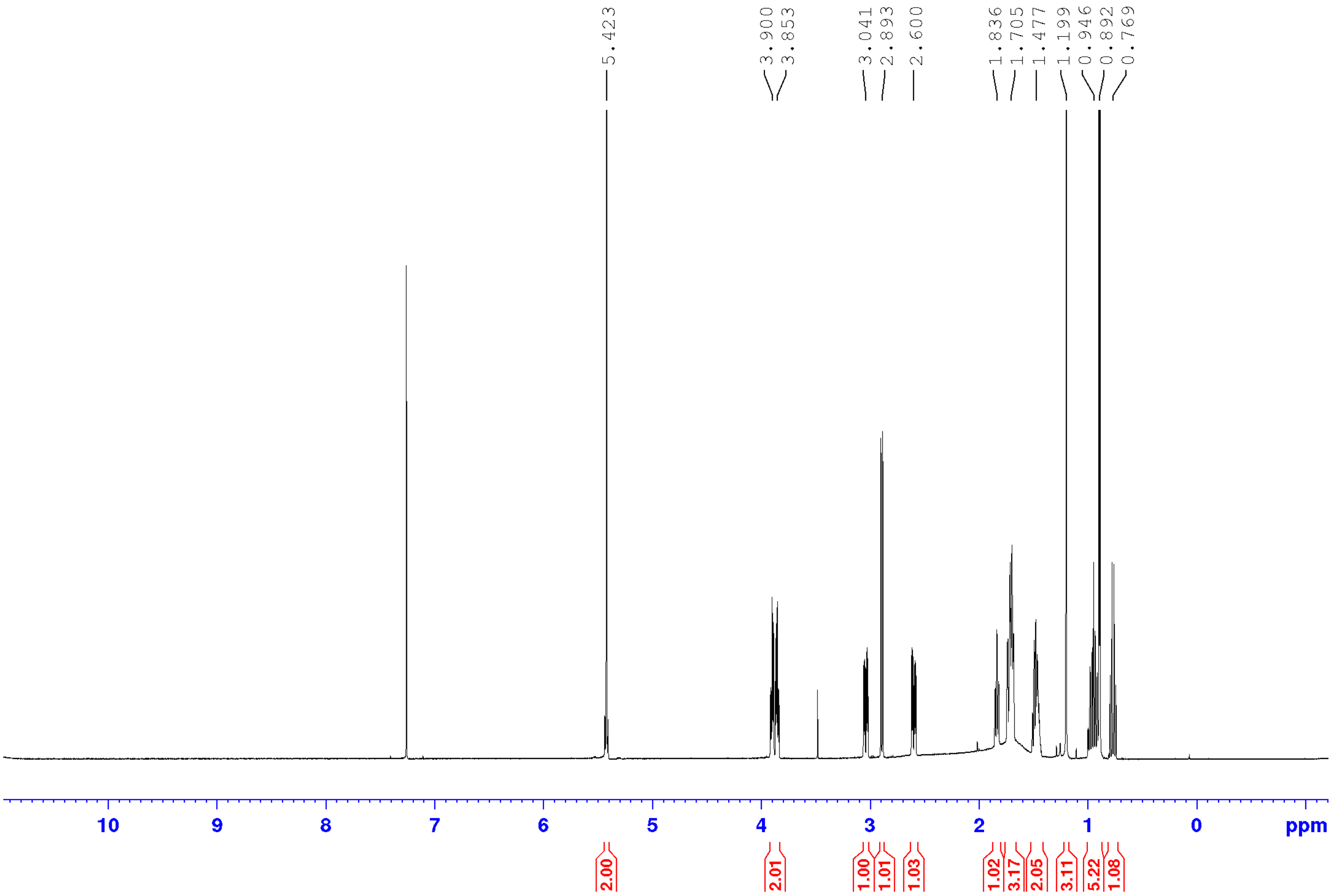

**Figure S82.$^{13}$C NMR spectrum of 11 measured at 176 MHz in $CDCl_3$**

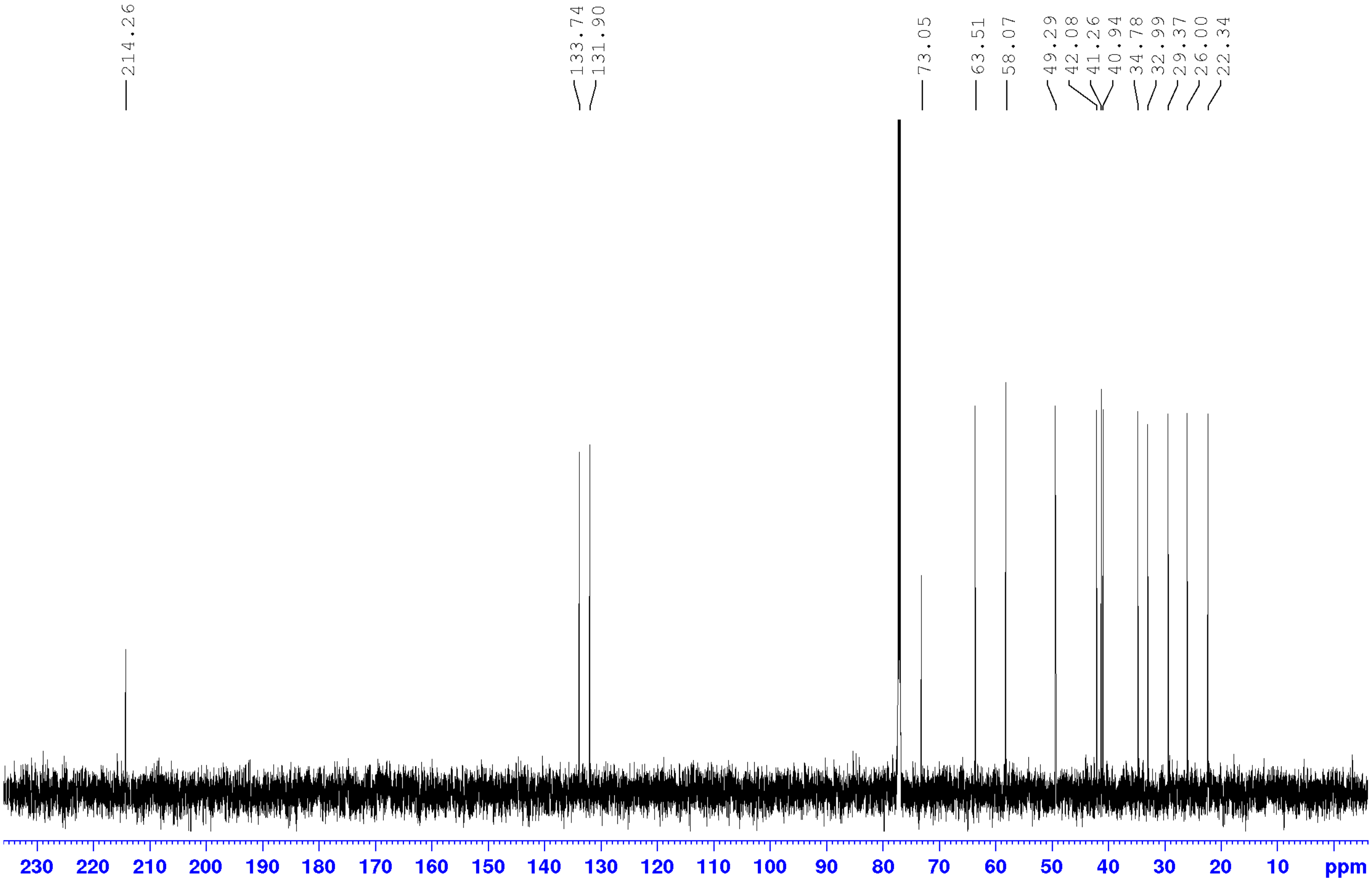


**Figure S83. HRESIMS for 12**

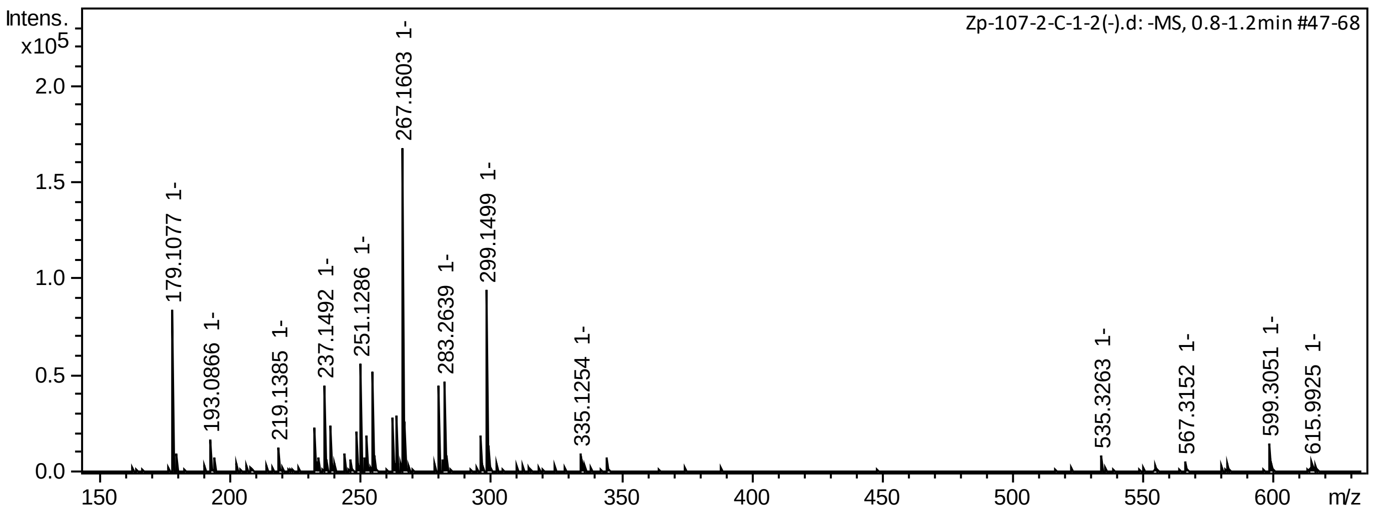

Intens.
x10^5
Zp-107-2-C-1-2(-).d: -MS, 0.8-1.2min #47-68
179.1077 1-
193.0866 1-
219.1385 1-
237.1492 1-
251.1286 1-
267.1603 1-
283.2639 1-
299.1499 1-
335.1254 1-
535.3263 1-
567.3152 1-
599.3051 1-
615.9925 1-
m/z


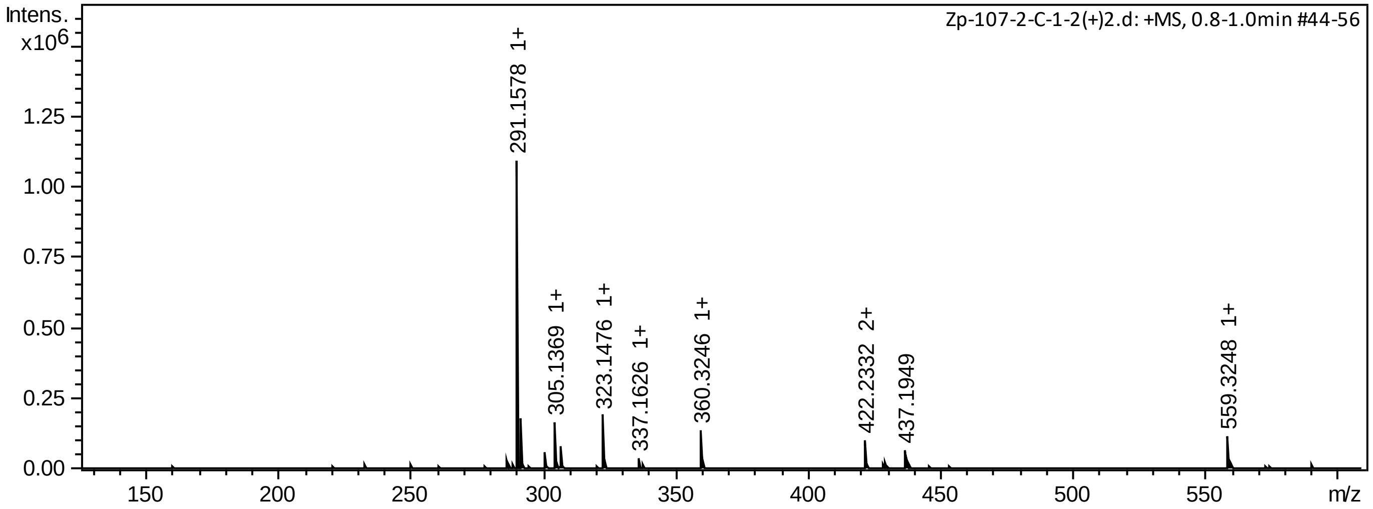

Intens.
x10^6
Zp-107-2-C-1-2(+)2.d: +MS, 0.8-1.0min #44-56
291.1578 1+
305.1369 1+
323.1476 1+
337.1626 1+
360.3246 1+
422.2332 2+
437.1949
559.3248 1+
m/z

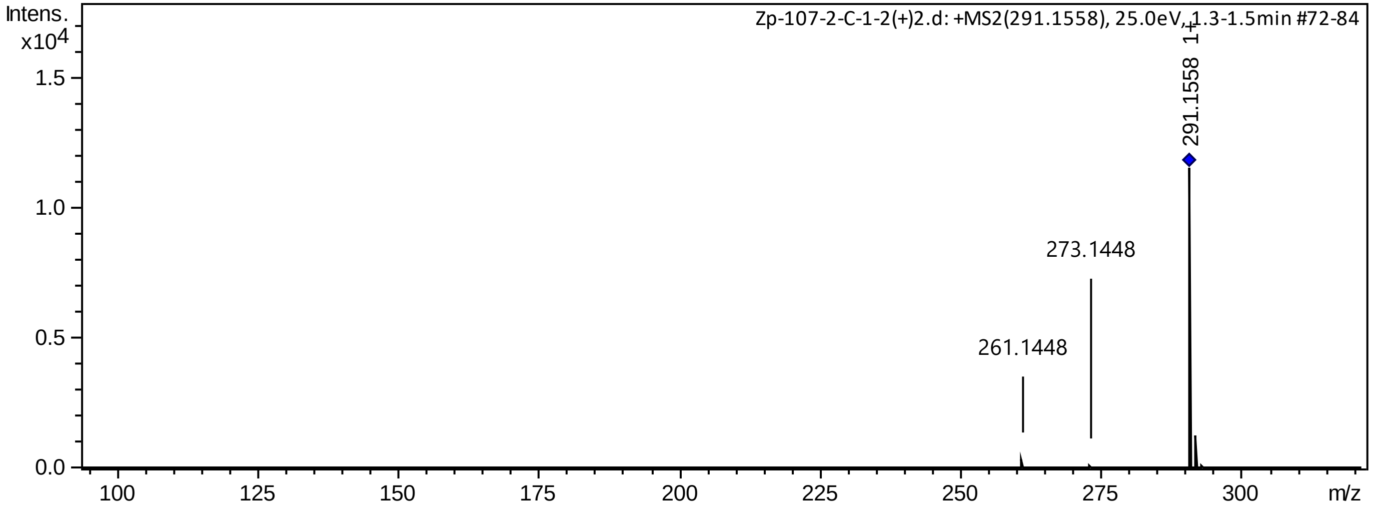


| | meas | calc | Δ (ppm) |
|---|---|---|---|
| $[M-H]^-$ | 267,1603 | 267,1602 | -0,4 |
| $[M+Na]^+$ | 291,1578 | 291,1567 | -3,8 |

**Figure S84. $^{1}$H NMR spectrum of 12 measured at 700 MHz in $CDCl_3$**

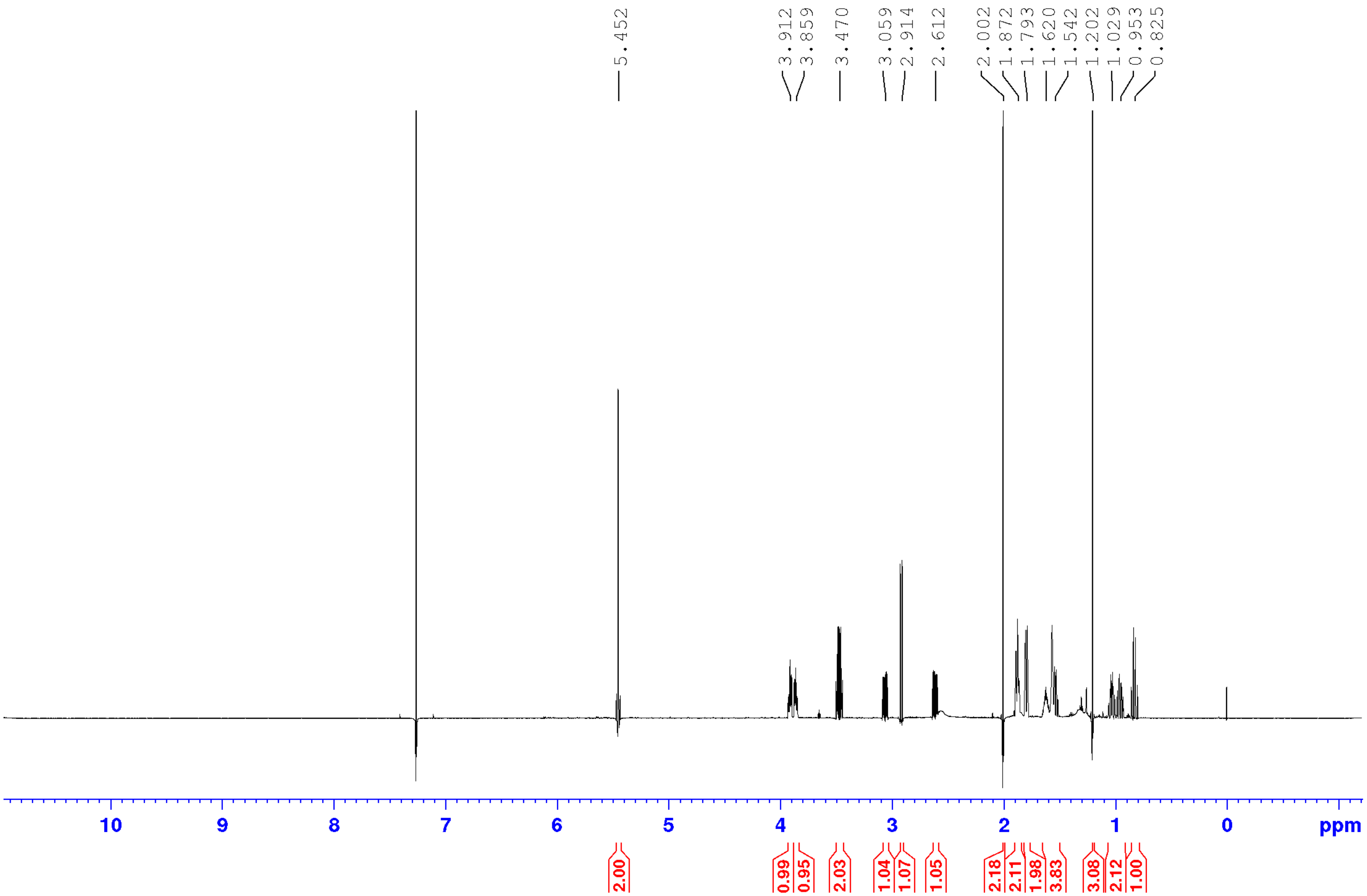

**Figure S85.$^{13}$C NMR spectrum of 12 measured at 176 MHz in $CDCl_3$**

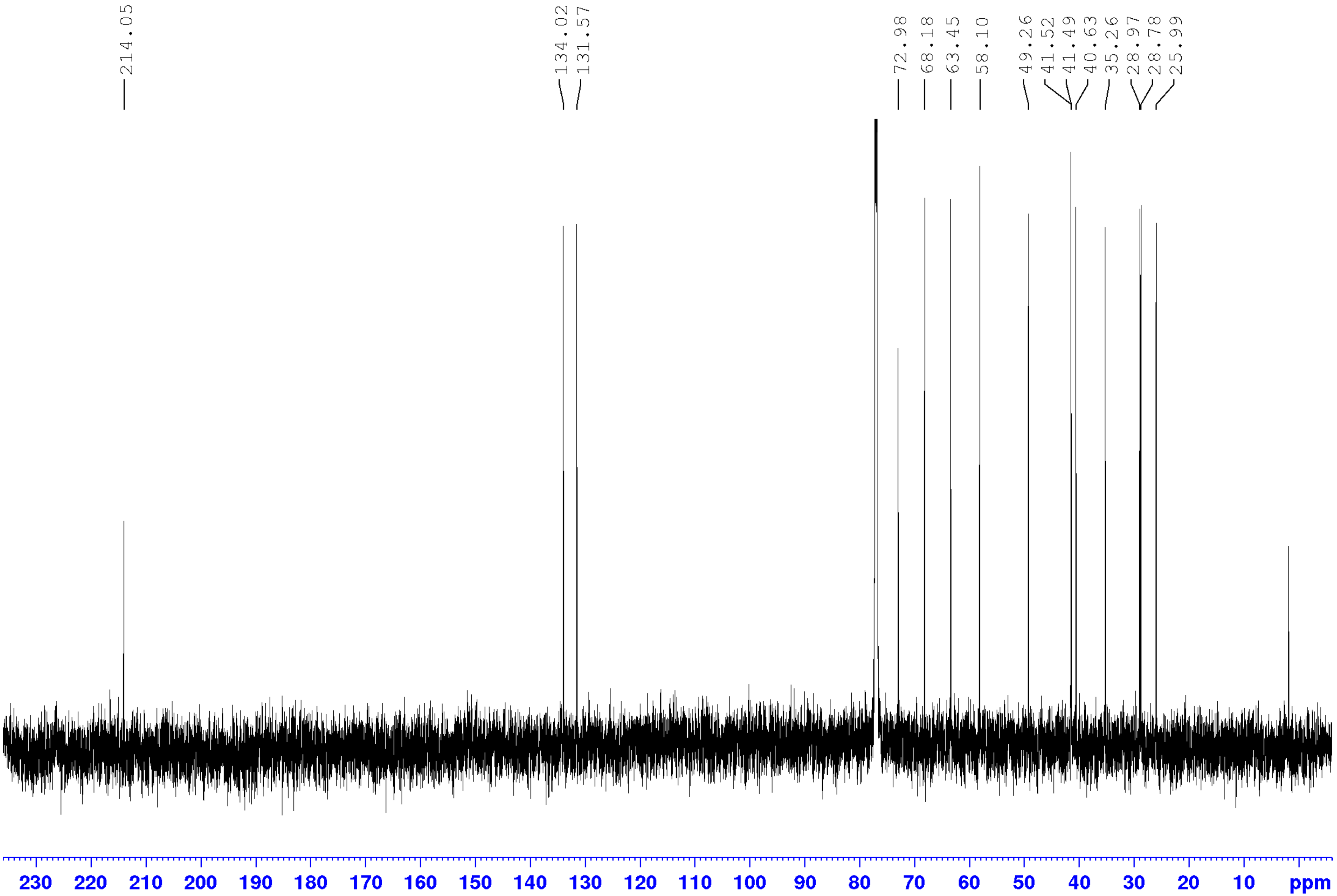

**Figure S86. HRESIMS for 13**

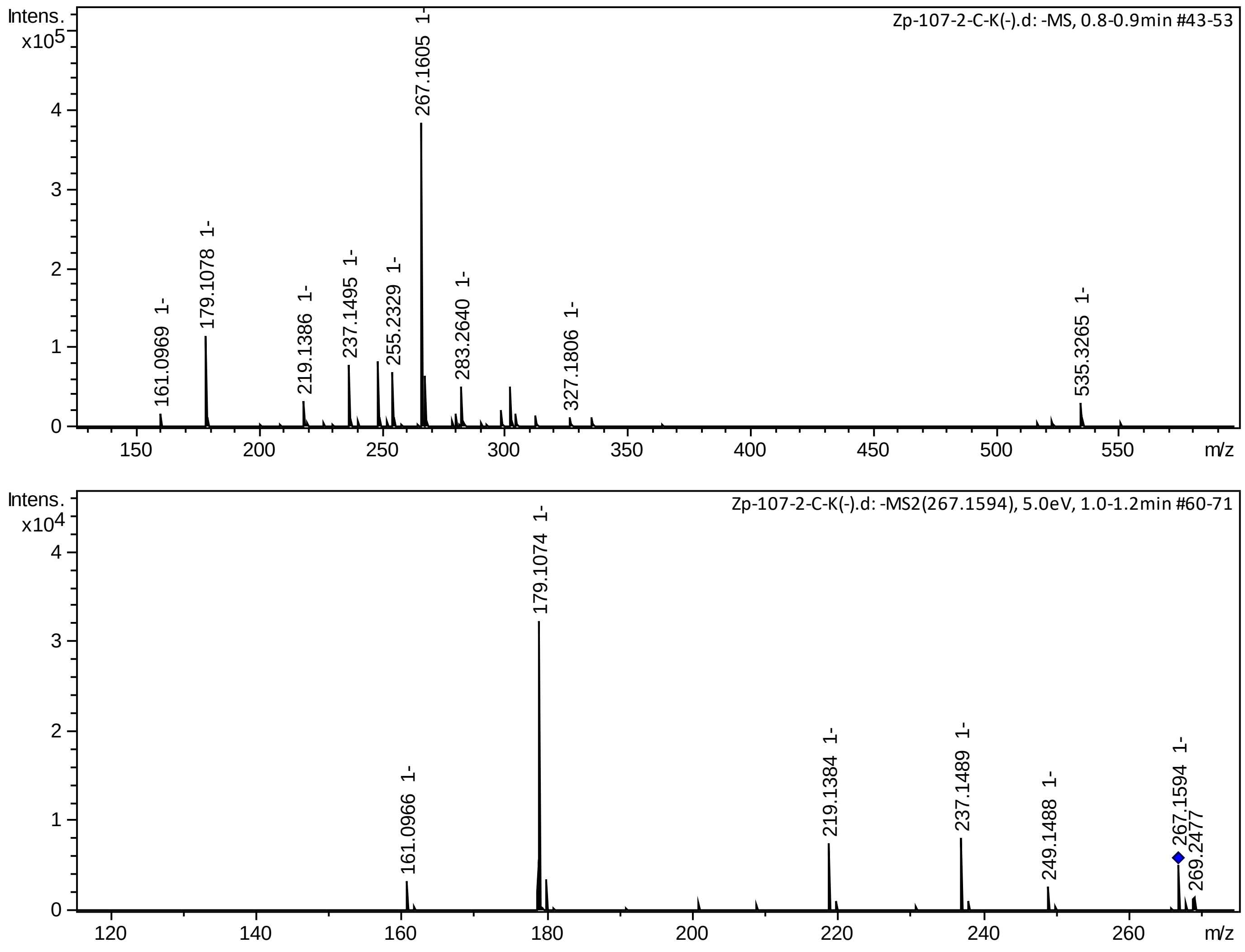

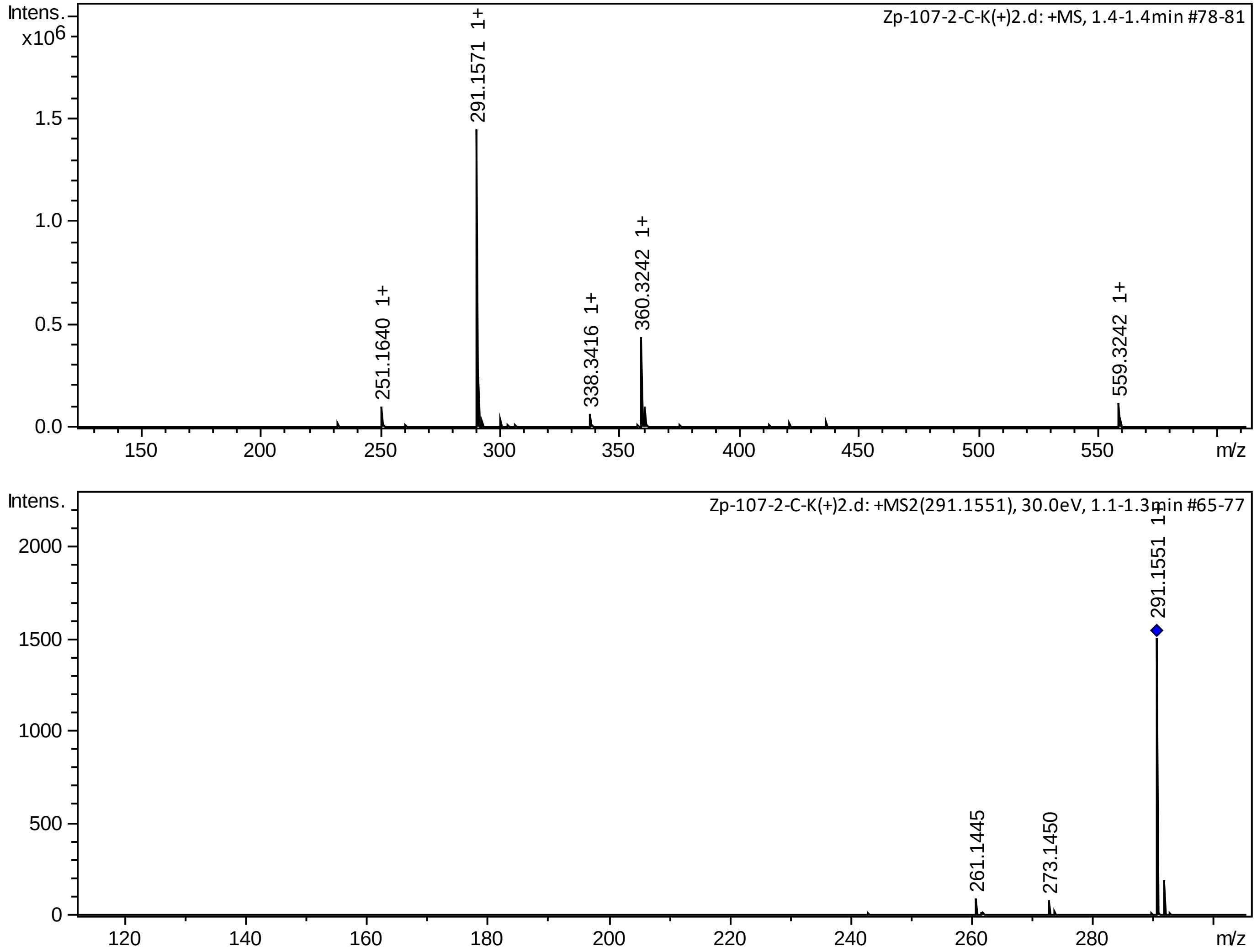


| | meas | calc | Δ (ppm) |
|---|---|---|---|
| $[M-H]^-$ | 267,1605 | 267,1602 | -1,1 |
| $[M+Na]^+$ | 291,1571 | 291,1567 | -1,4 |

**Figure S87. ¹H NMR spectrum of 13 measured at 700 MHz in $CDCl_3$**

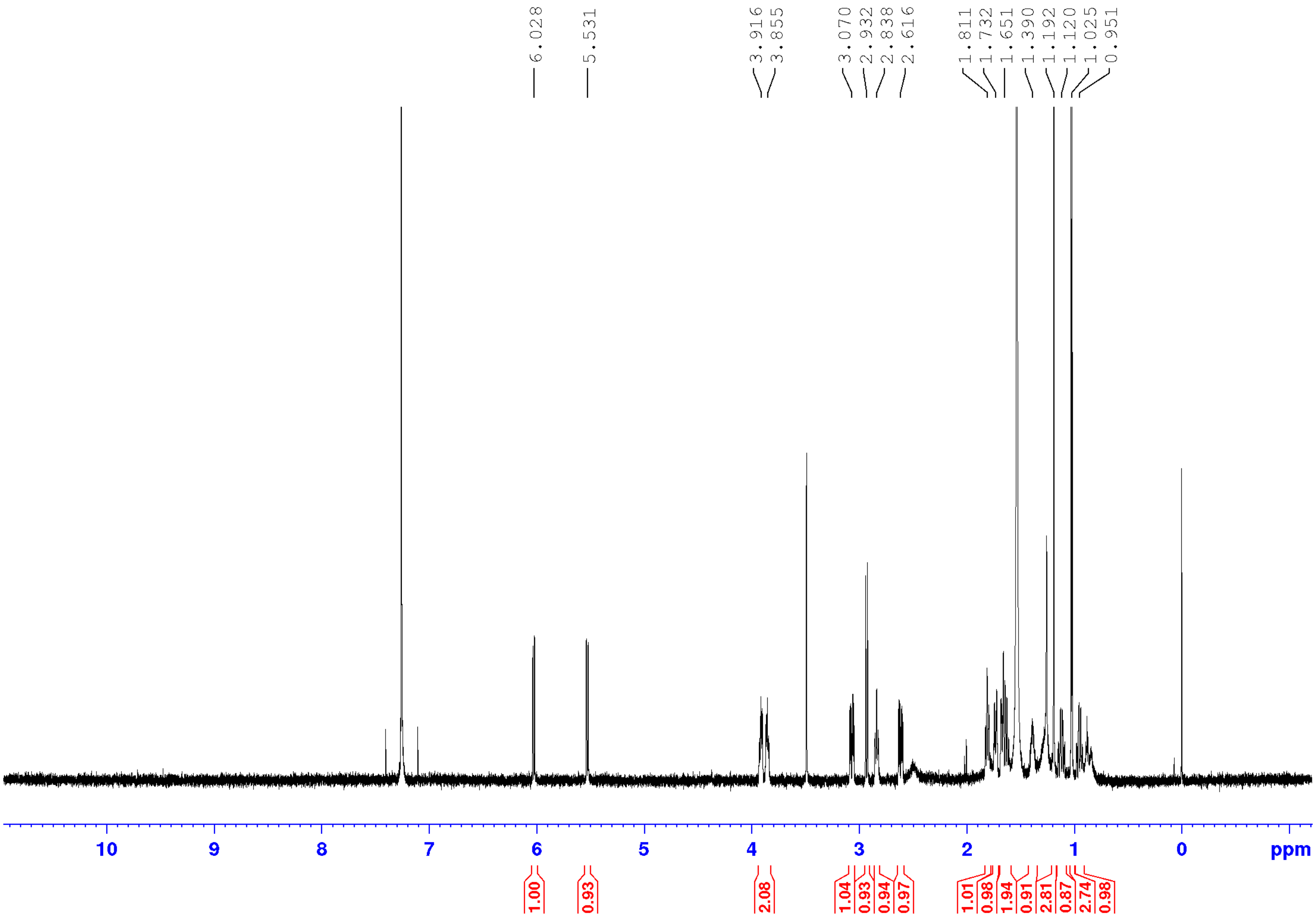

**Figure S88.**[13]**C NMR spectrum of 13 measured at 176 MHz in CDCl**$_3$

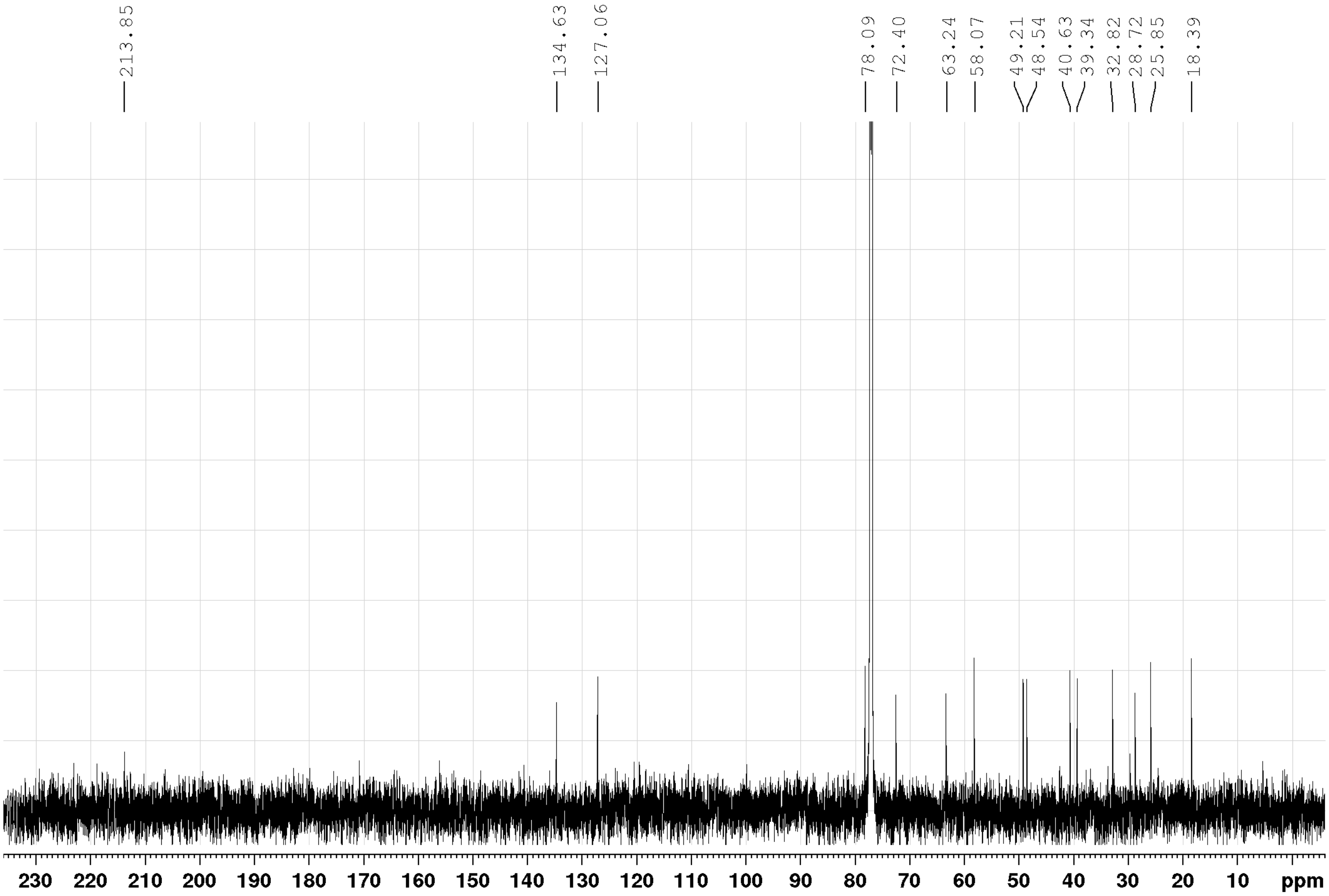

**Figure S89. HRESIMS for 14**

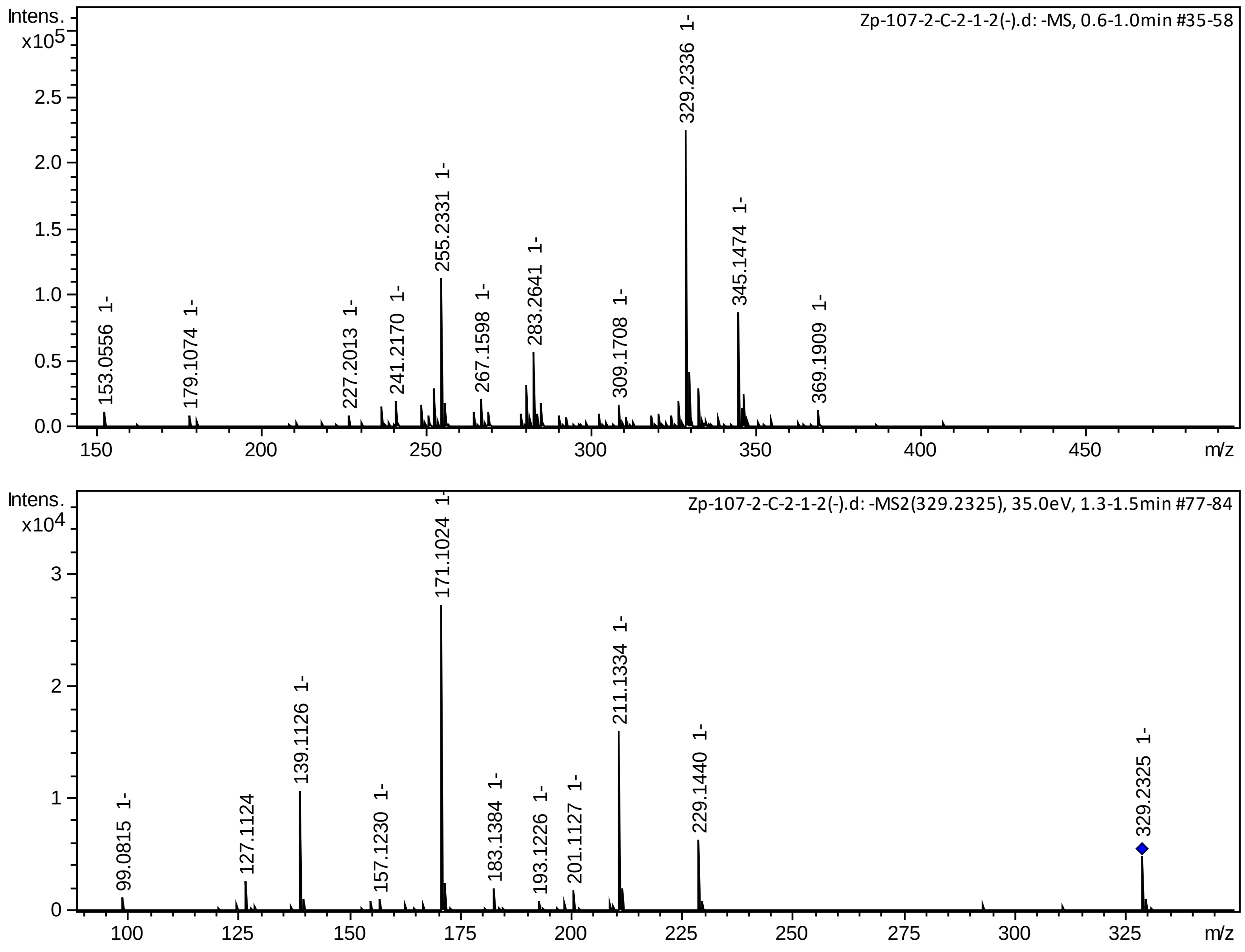

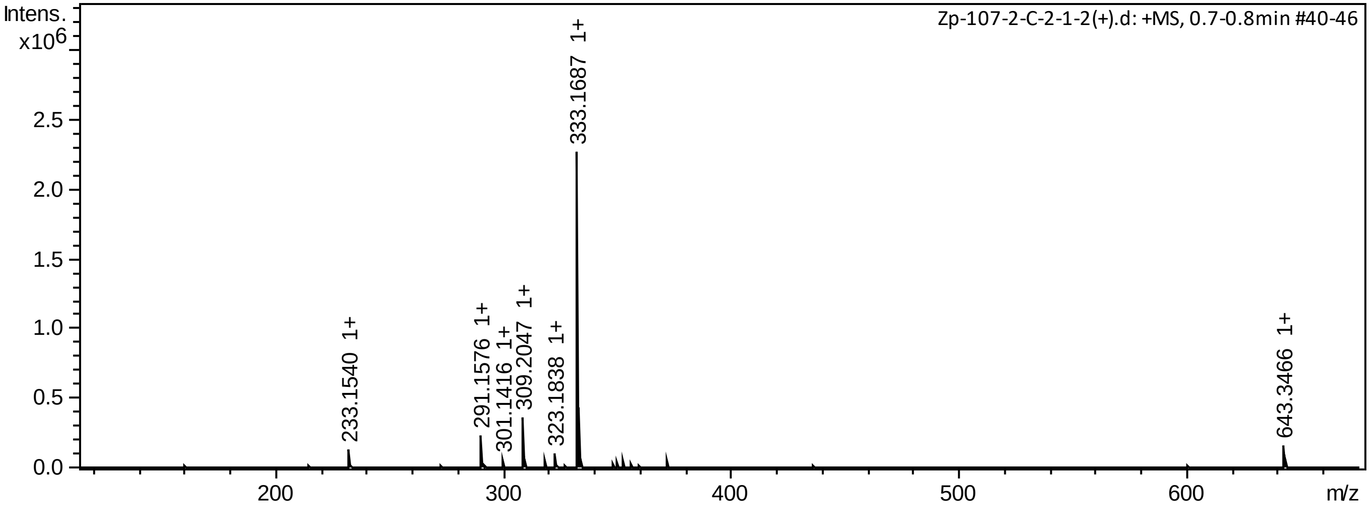


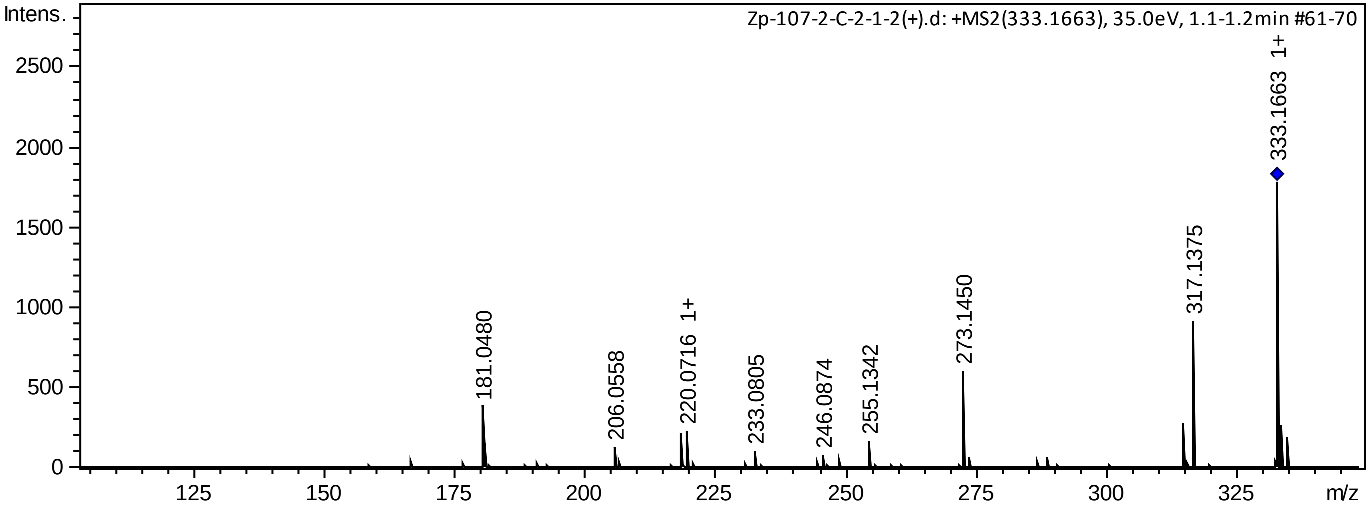


| | meas | calc | Δ (ppm) |
|---|---|---|---|
| $[M-H]^-$ | 309,1708 | 309,1707 | -0,3 |
| $[M+Na]^+$ | 333,1687 | 333,1672 | -4,5 |

Figure S90. $^1H$ NMR spectrum of 14 measured at 700 MHz in $CDCl_3$

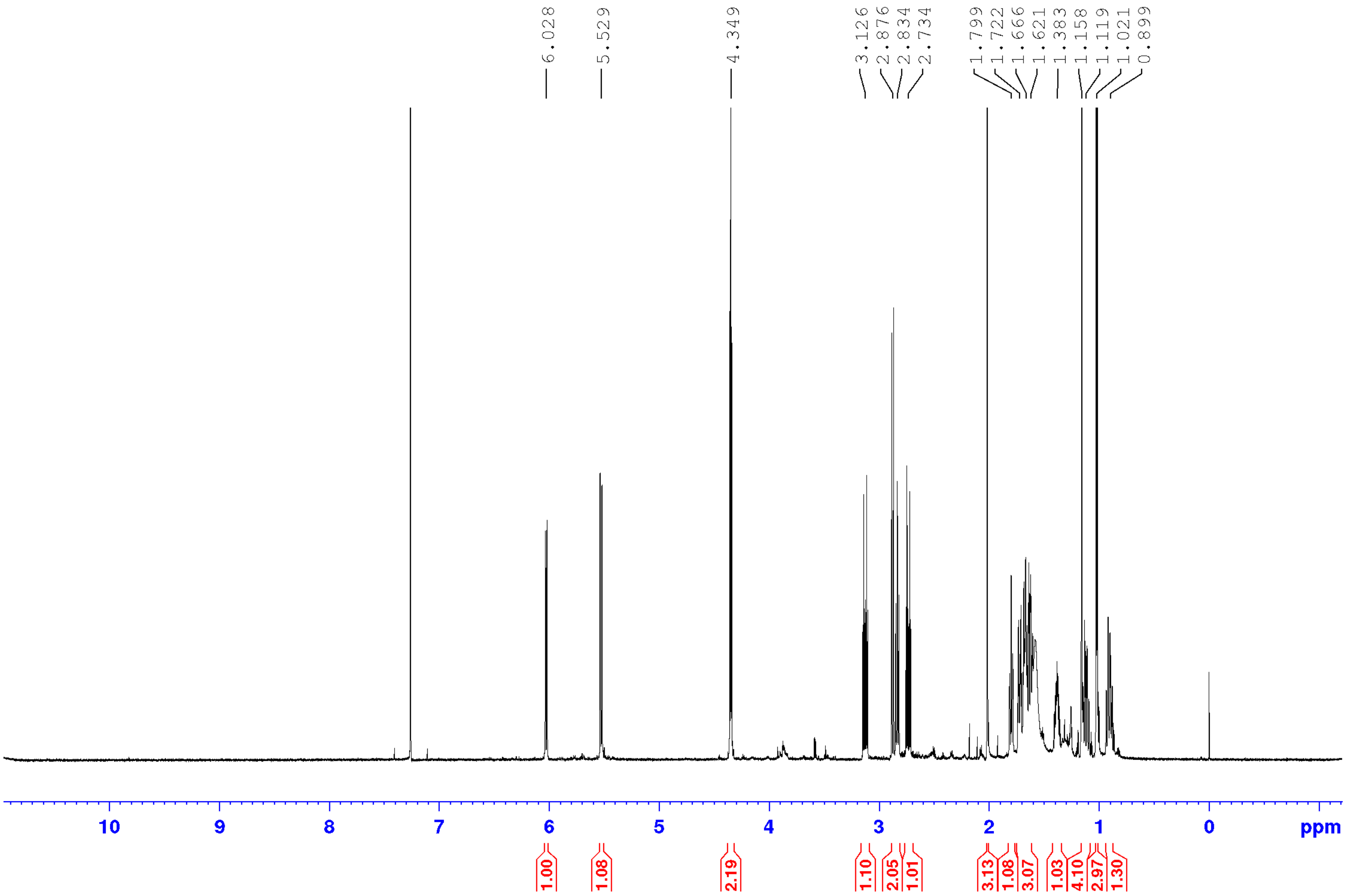

**Figure S91.$^{13}$C NMR spectrum of 14 measured at 176 MHz in $CDCl_3$**

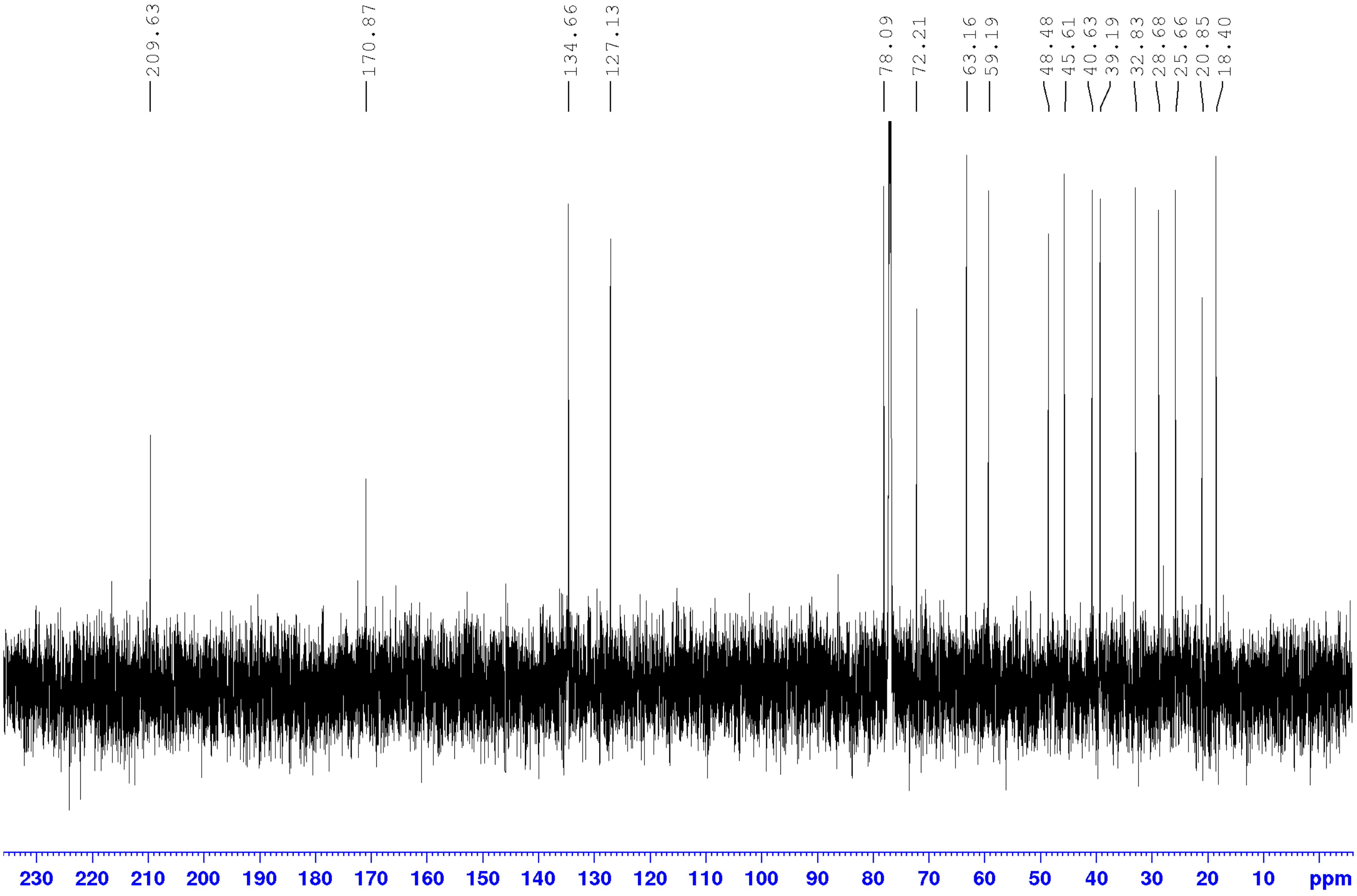

**Figure S92. UV and CD data for 1 in MeOH**

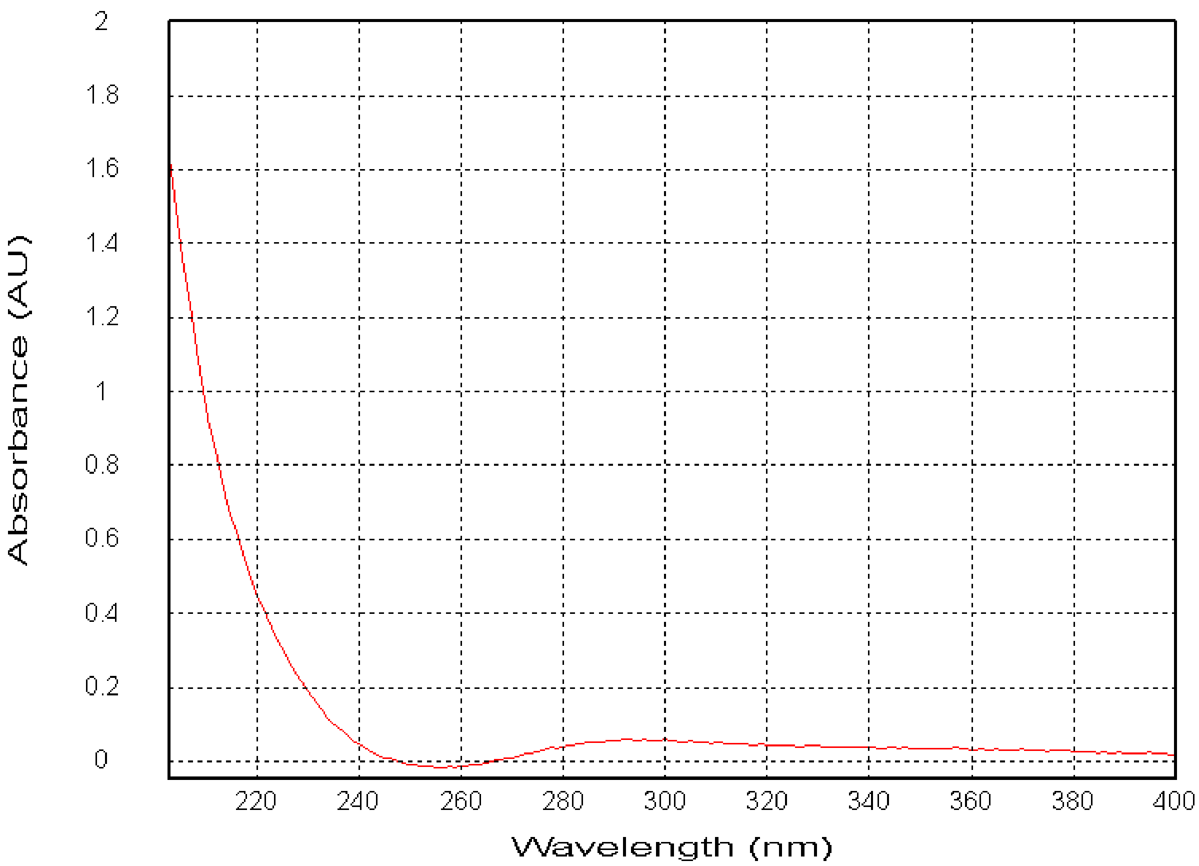

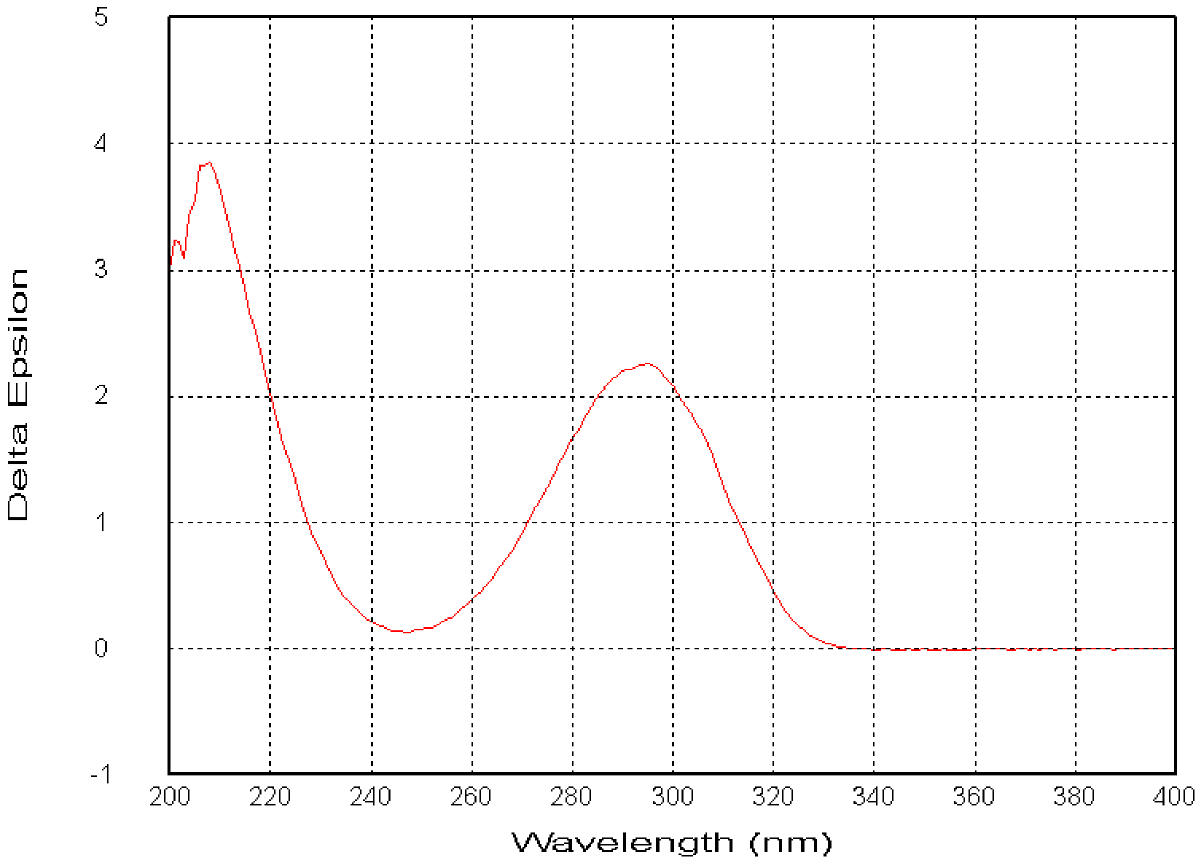

Delta Epsilon
5
4
3
2
1
0
-1
200
220
240
260
280
300
320
340
360
380
400
Wavelength (nm)

**Figure S93. UV and CD data for 2 in MeOH**

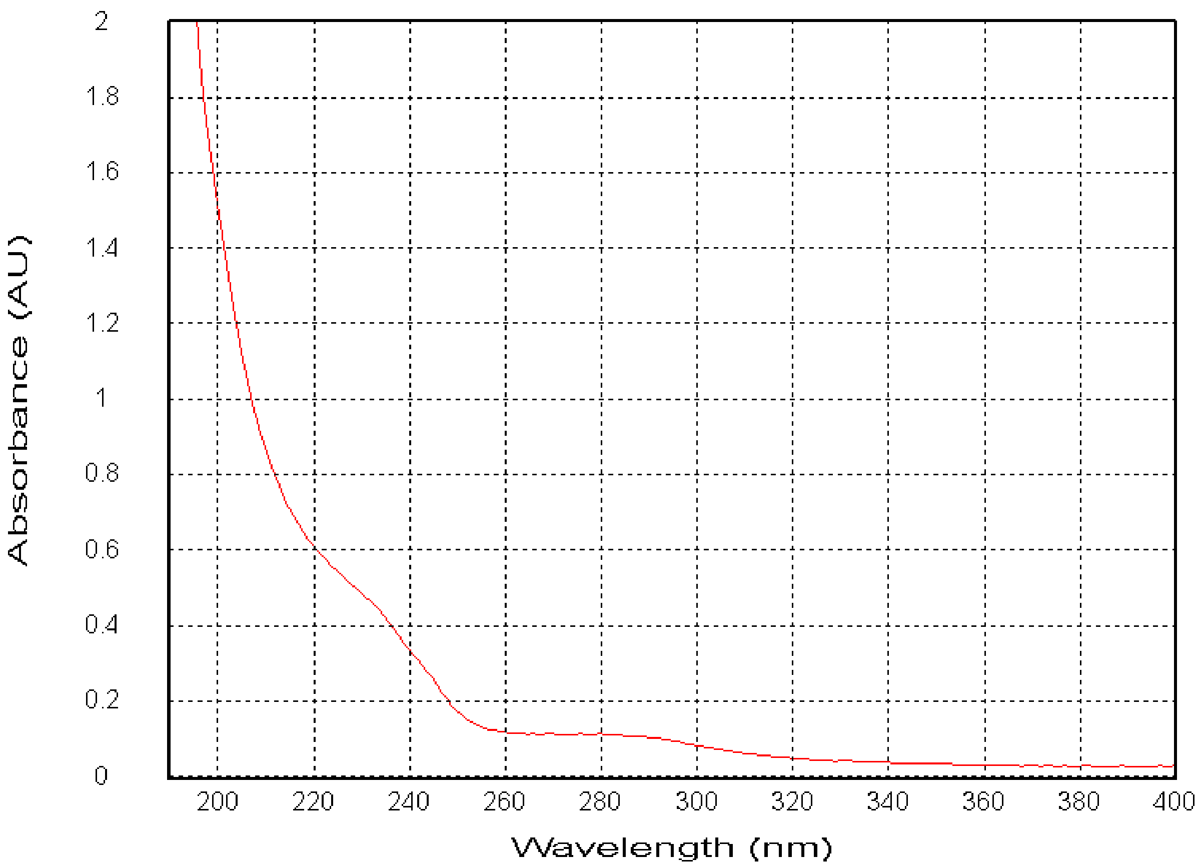

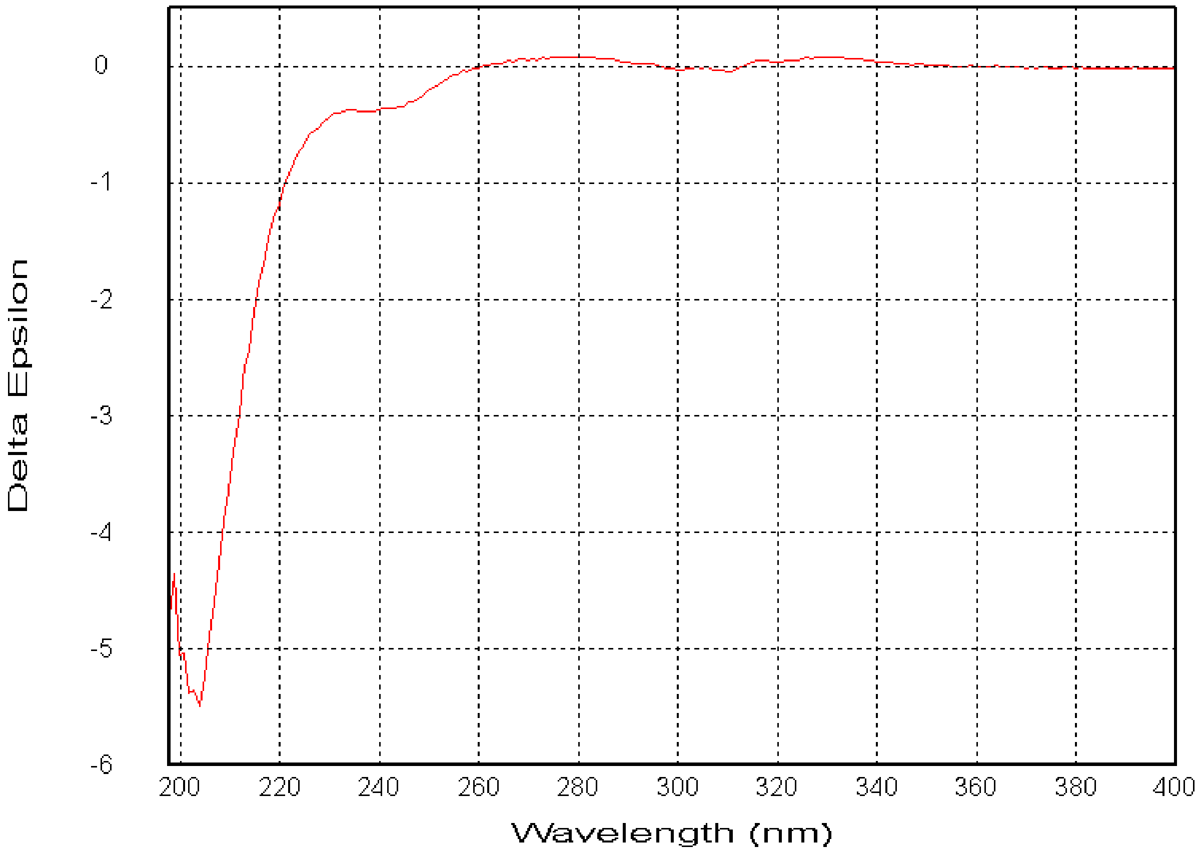

Delta Epsilon
0
-1
-2
-3
-4
-5
-6
200
220
240
260
280
300
320
340
360
380
400
Wavelength (nm)

**Figure S94. UV and CD data for 3 in MeOH**

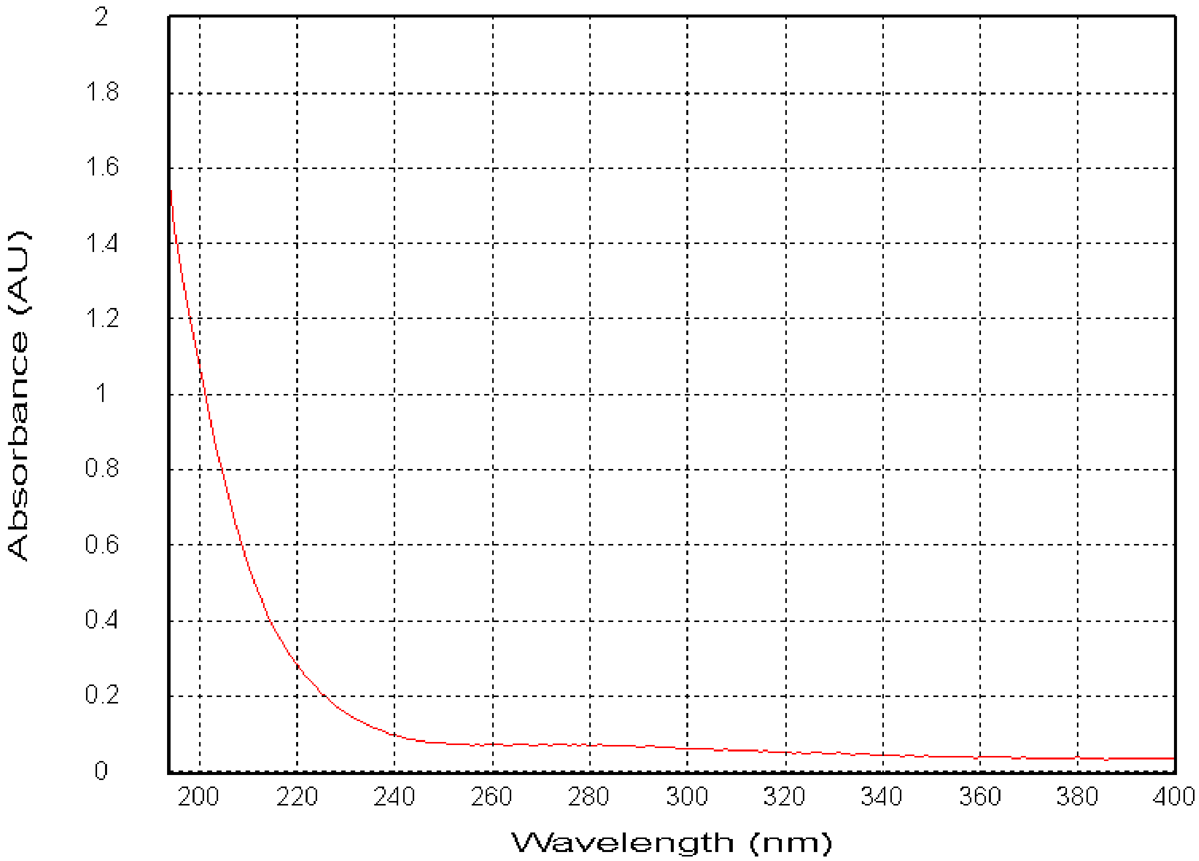

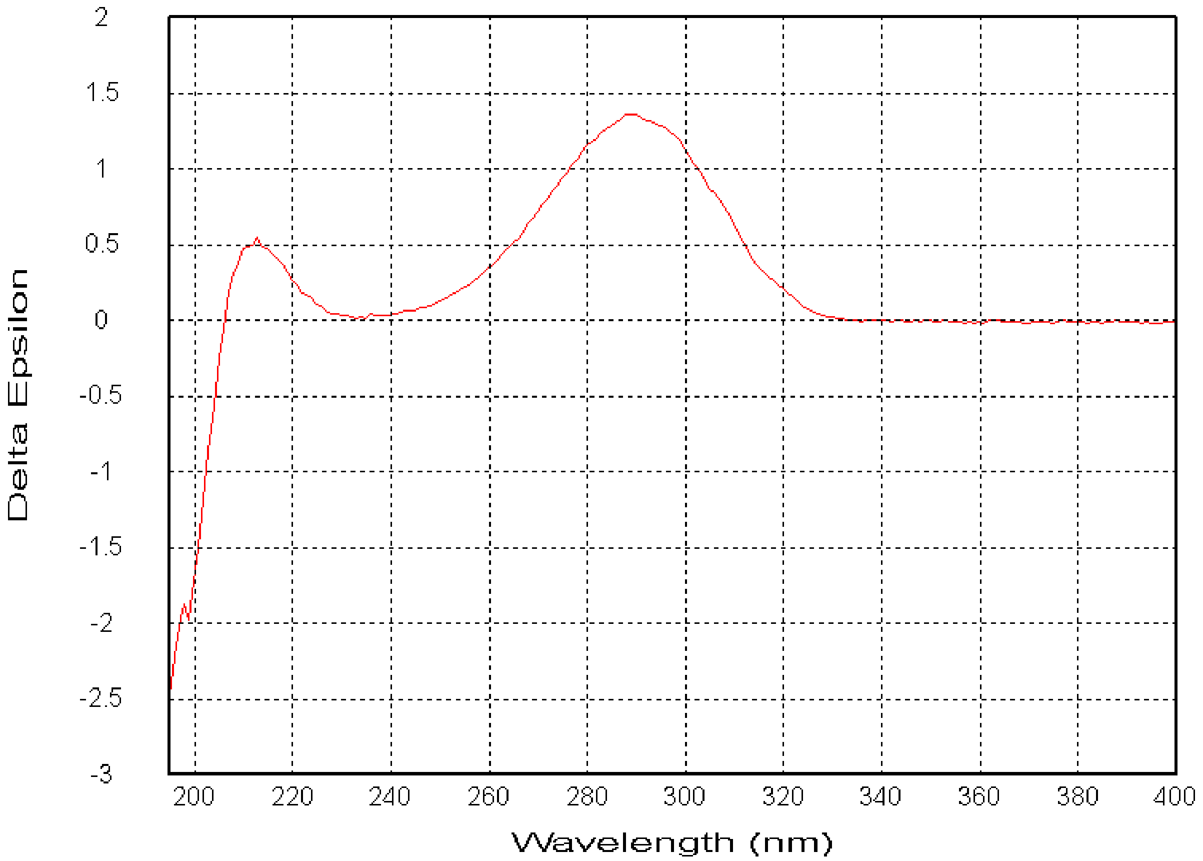

Delta Epsilon
2
1.5
1
0.5
0
-0.5
-1
-1.5
-2
-2.5
-3
200
220
240
260
280
300
320
340
360
380
400
Wavelength (nm)

**Figure S95. UV and CD data for 4 in MeOH**

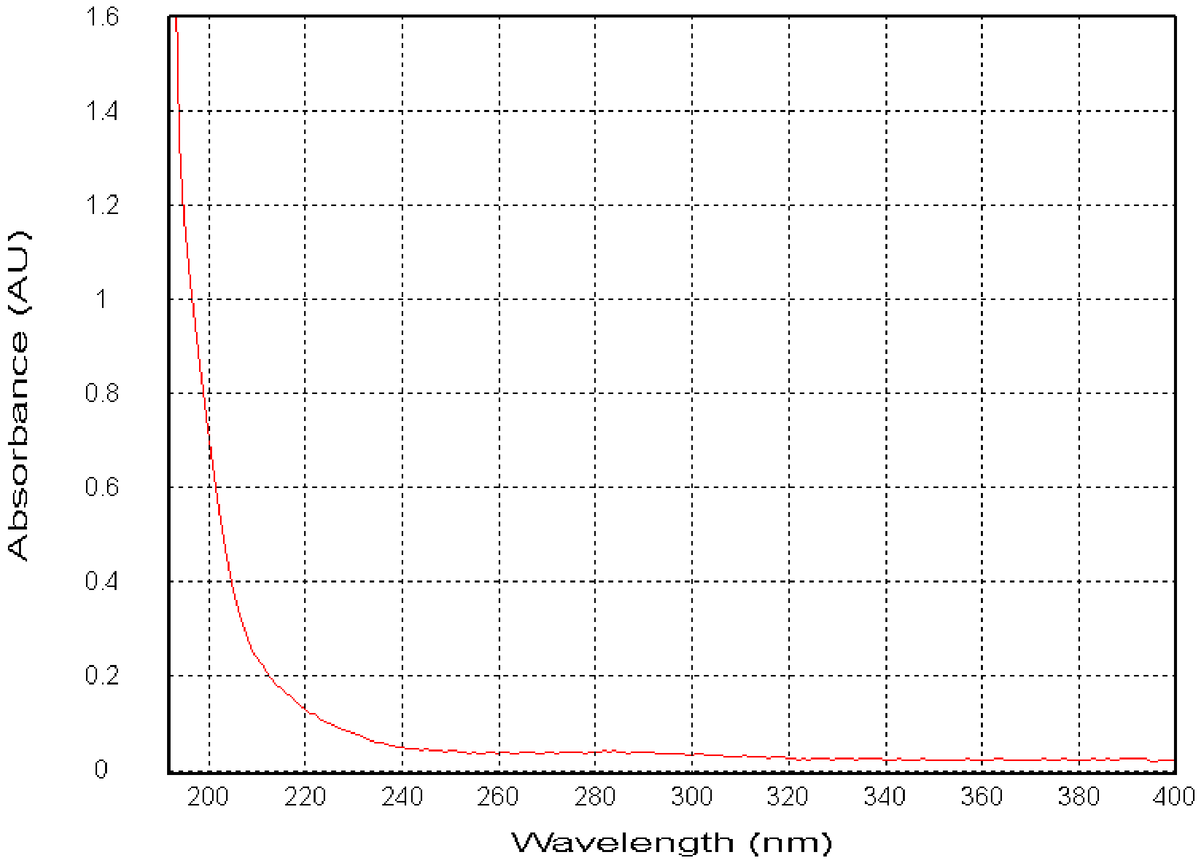

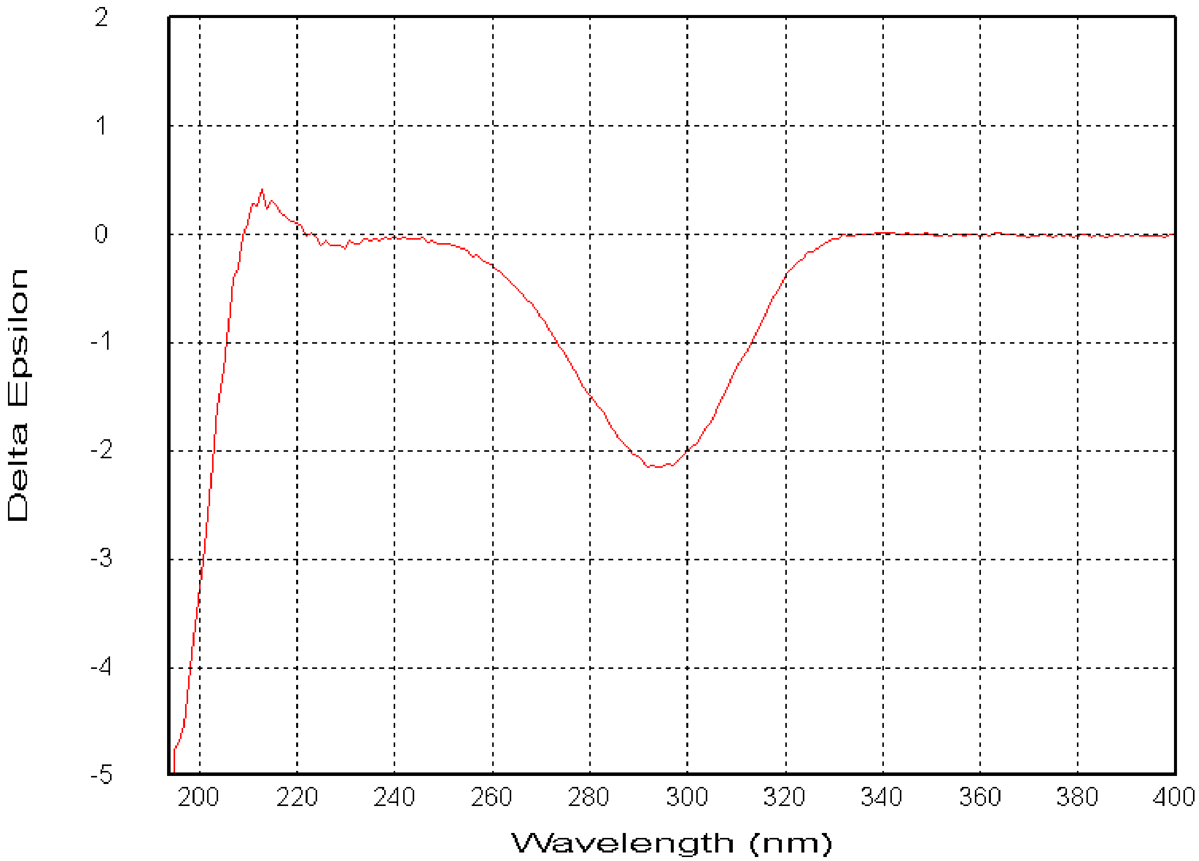

Delta Epsilon
2
1
0
-1
-2
-3
-4
-5
200
220
240
260
280
300
320
340
360
380
400
Wavelength (nm)

**Figure S96. UV and CD data for 5 in MeOH**

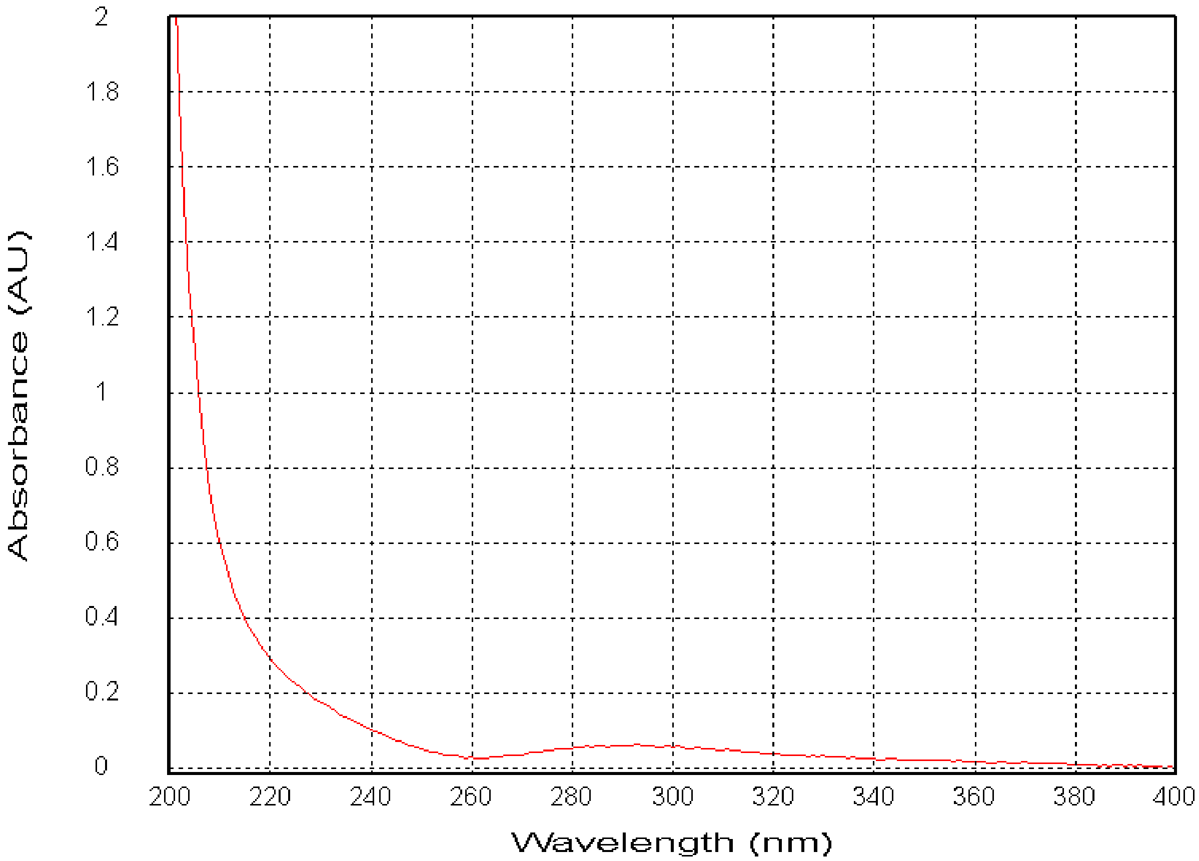

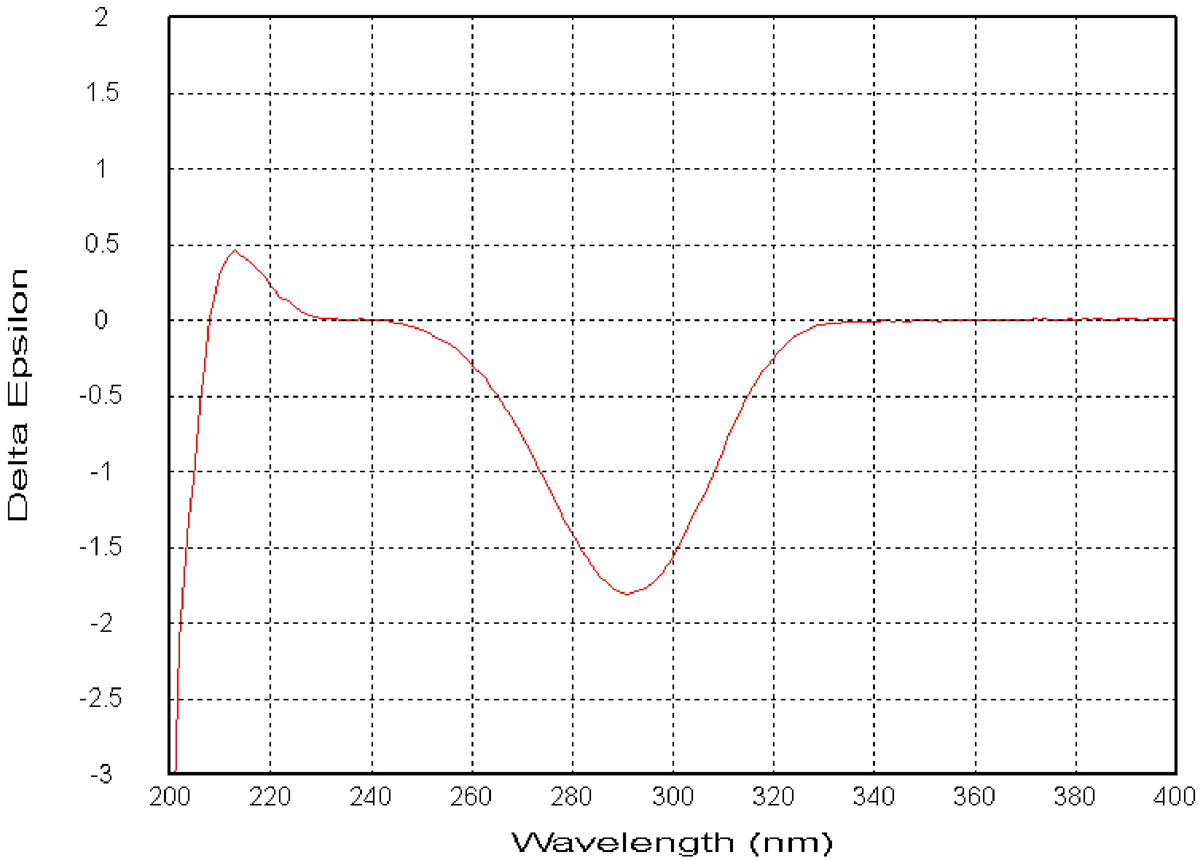
Delta Epsilon
Wavelength (nm)
2
1.5
1
0.5
0
-0.5
-1
-1.5
-2
-2.5
-3
200
220
240
260
280
300
320
340
360
380
400

**Figure S97. UV and CD data for 6 in MeOH**

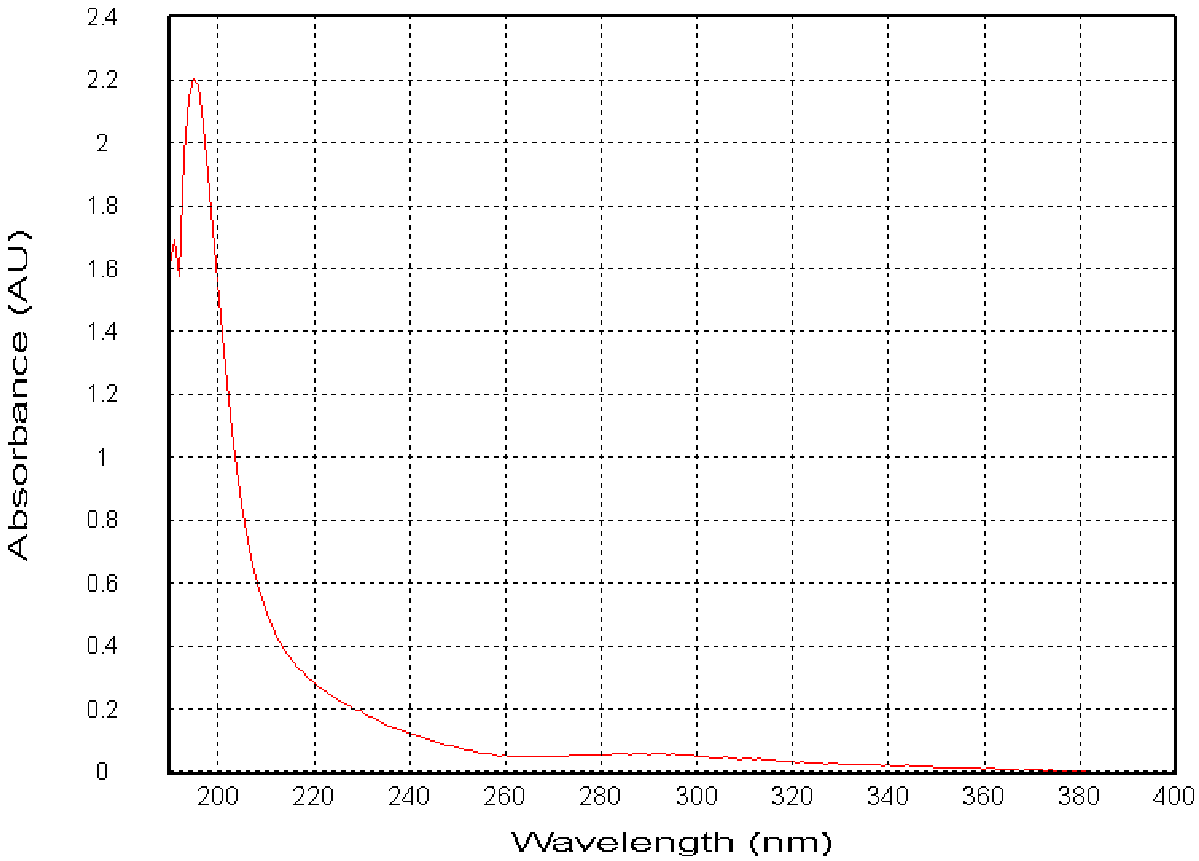

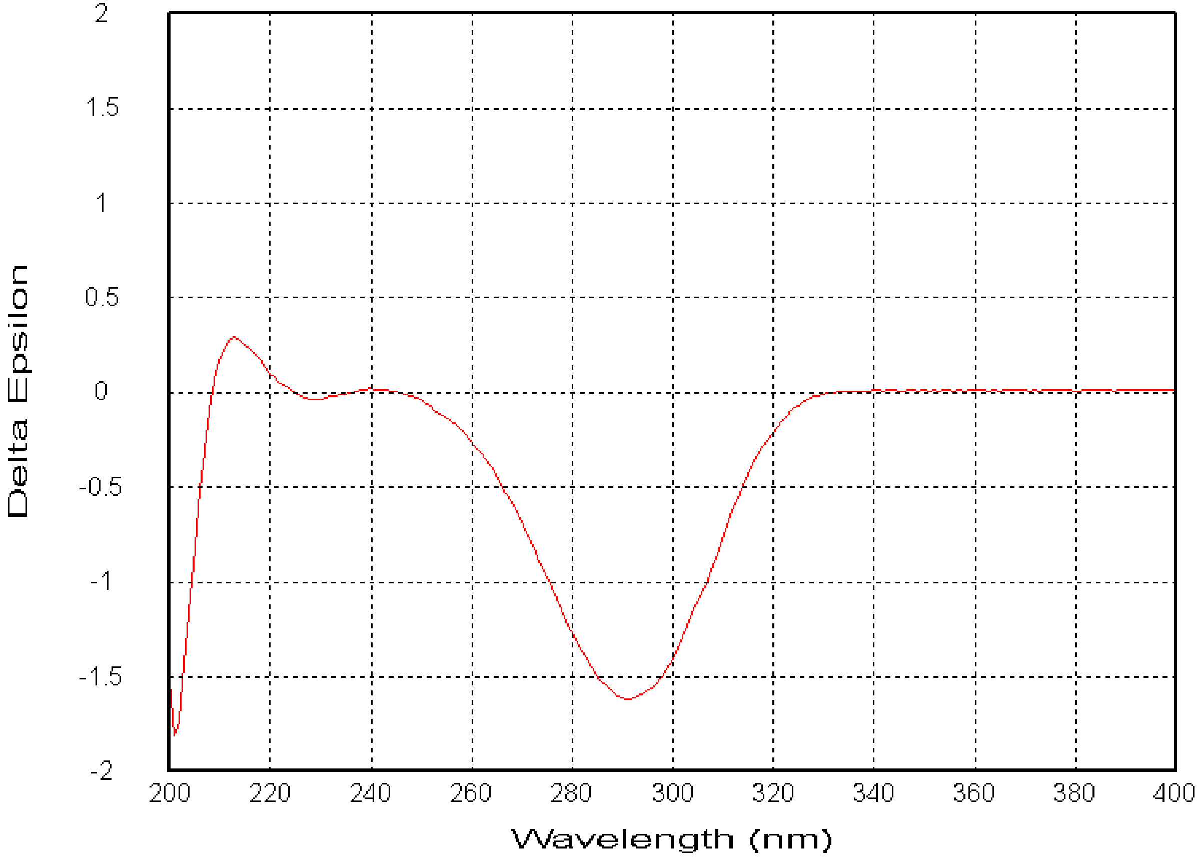
Delta Epsilon
2
1.5
1
0.5
0
-0.5
-1
-1.5
-2
200
220
240
260
280
300
320
340
360
380
400
Wavelength (nm)

**Figure S98. UV and CD data for 7 in MeOH**

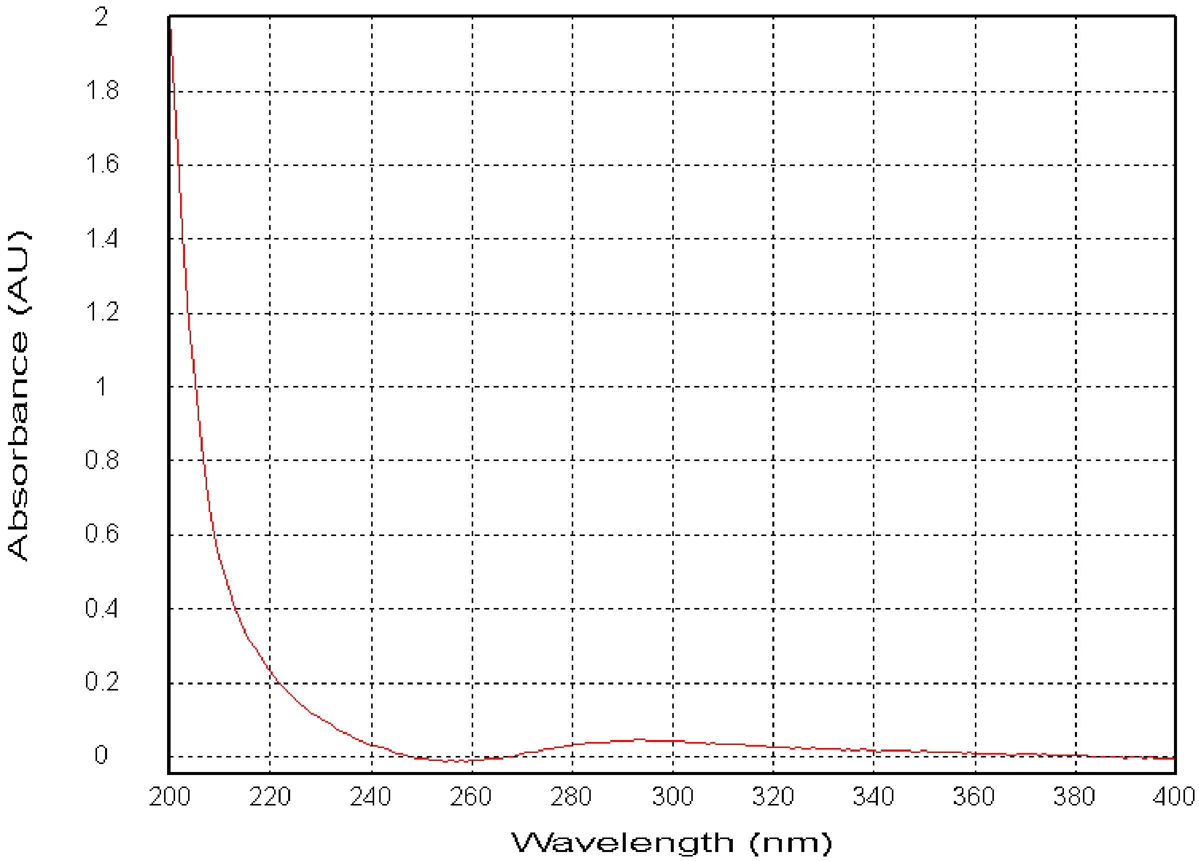

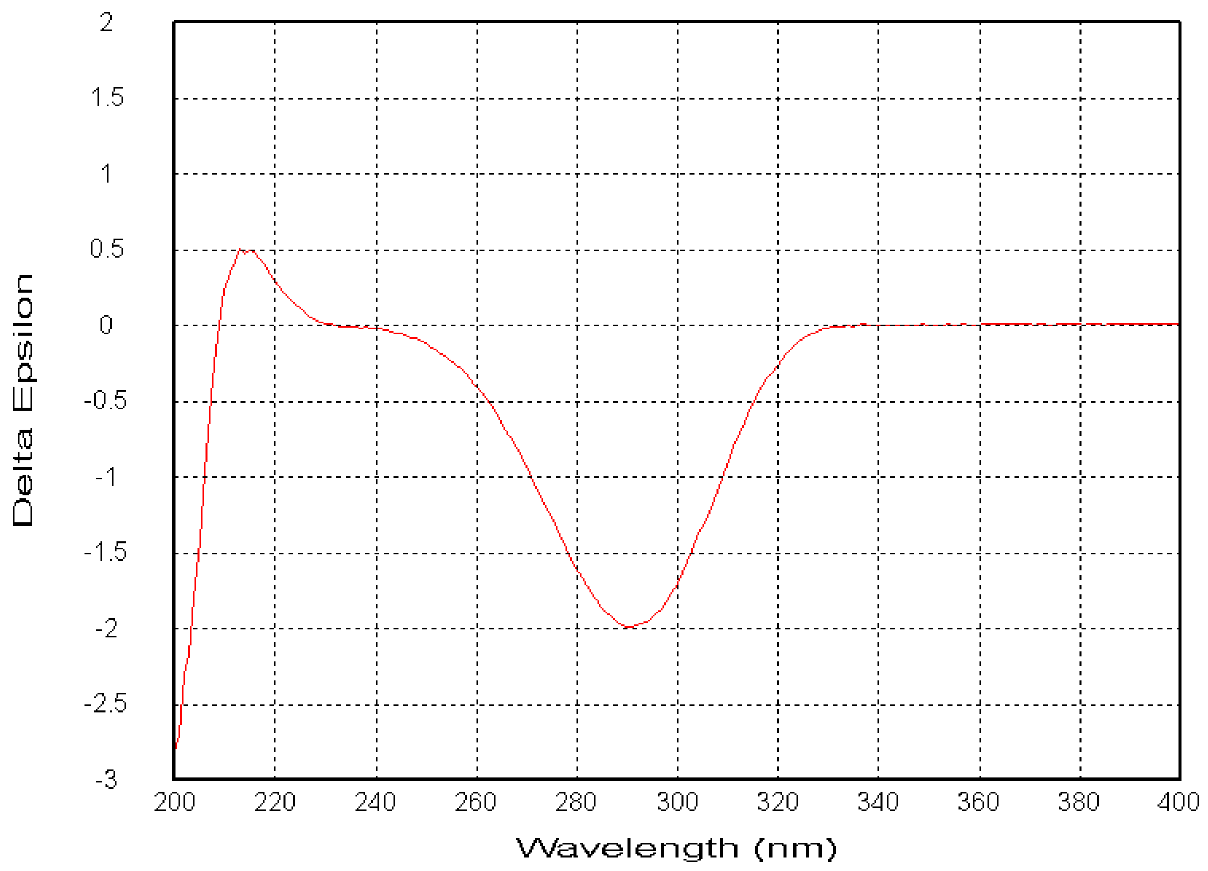

2
1.5
1
0.5
0
-0.5
-1
-1.5
-2
-2.5
-3
Delta Epsilon
200
220
240
260
280
300
320
340
360
380
400
Wavelength (nm)

**Figure S99. UV and CD data for 8 in MeOH**

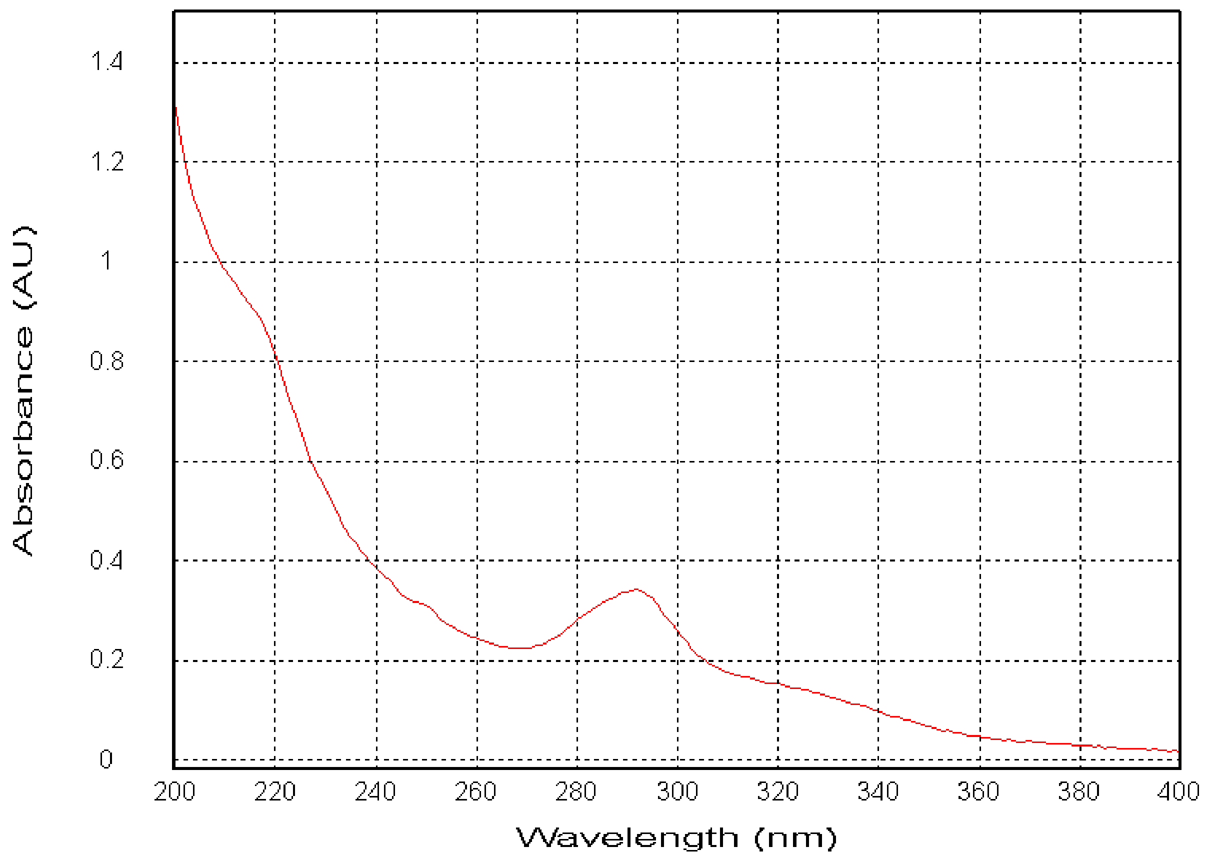

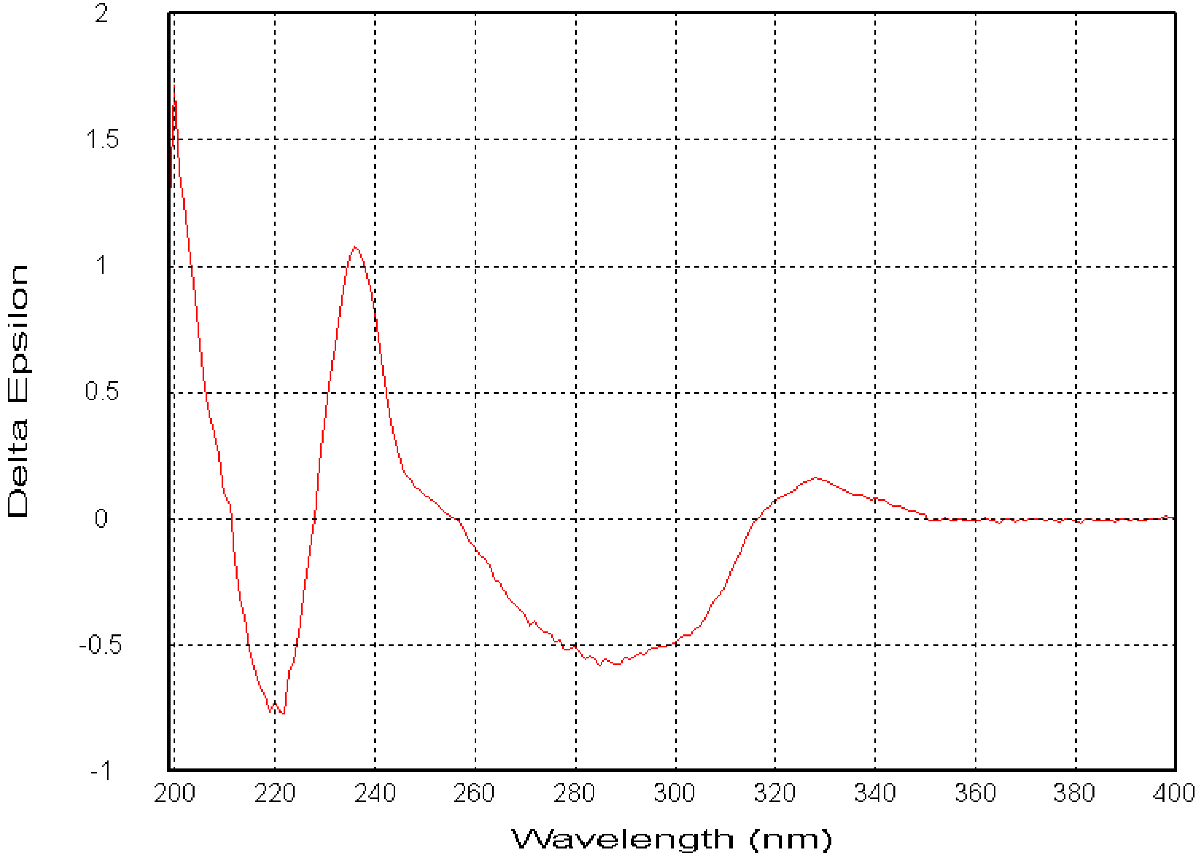

Delta Epsilon
2
1.5
1
0.5
0
-0.5
-1
200
220
240
260
280
300
320
340
360
380
400
Wavelength (nm)

**Figure S100. UV and CD data for 9 in MeOH**

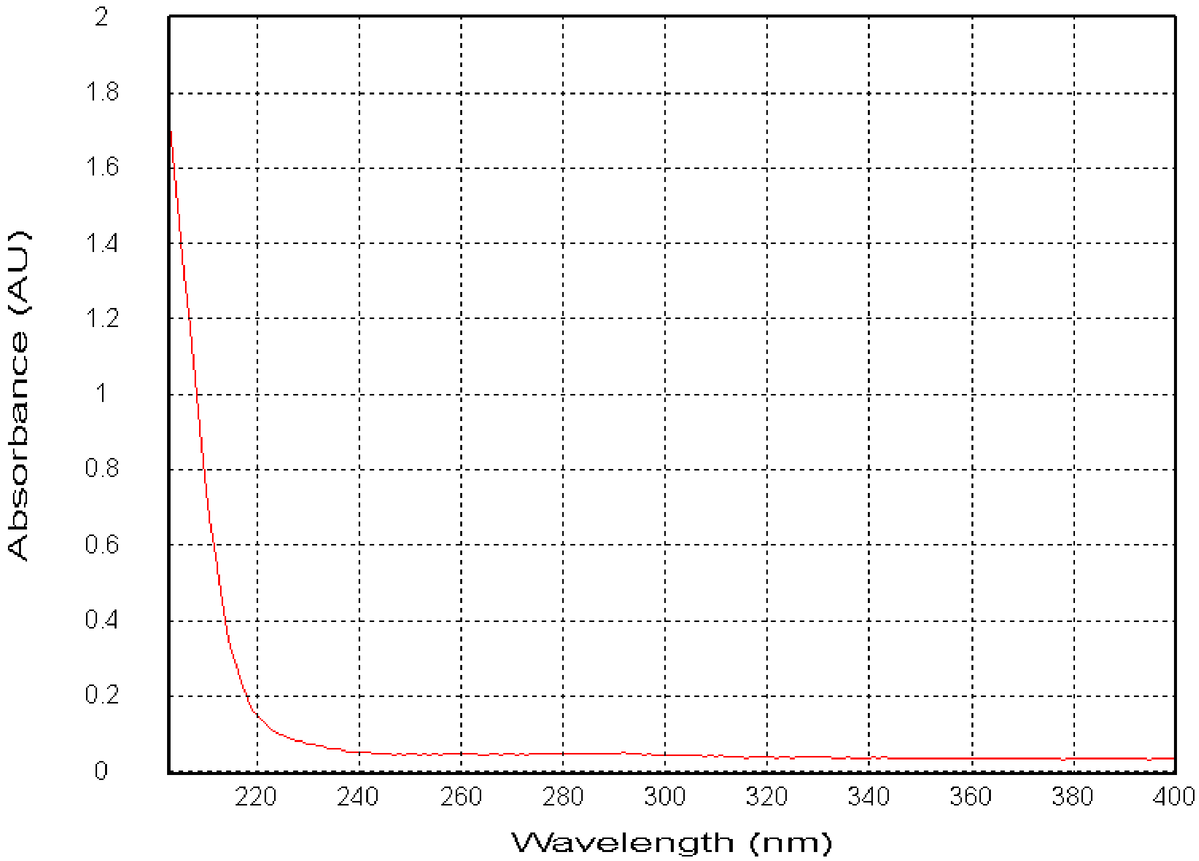

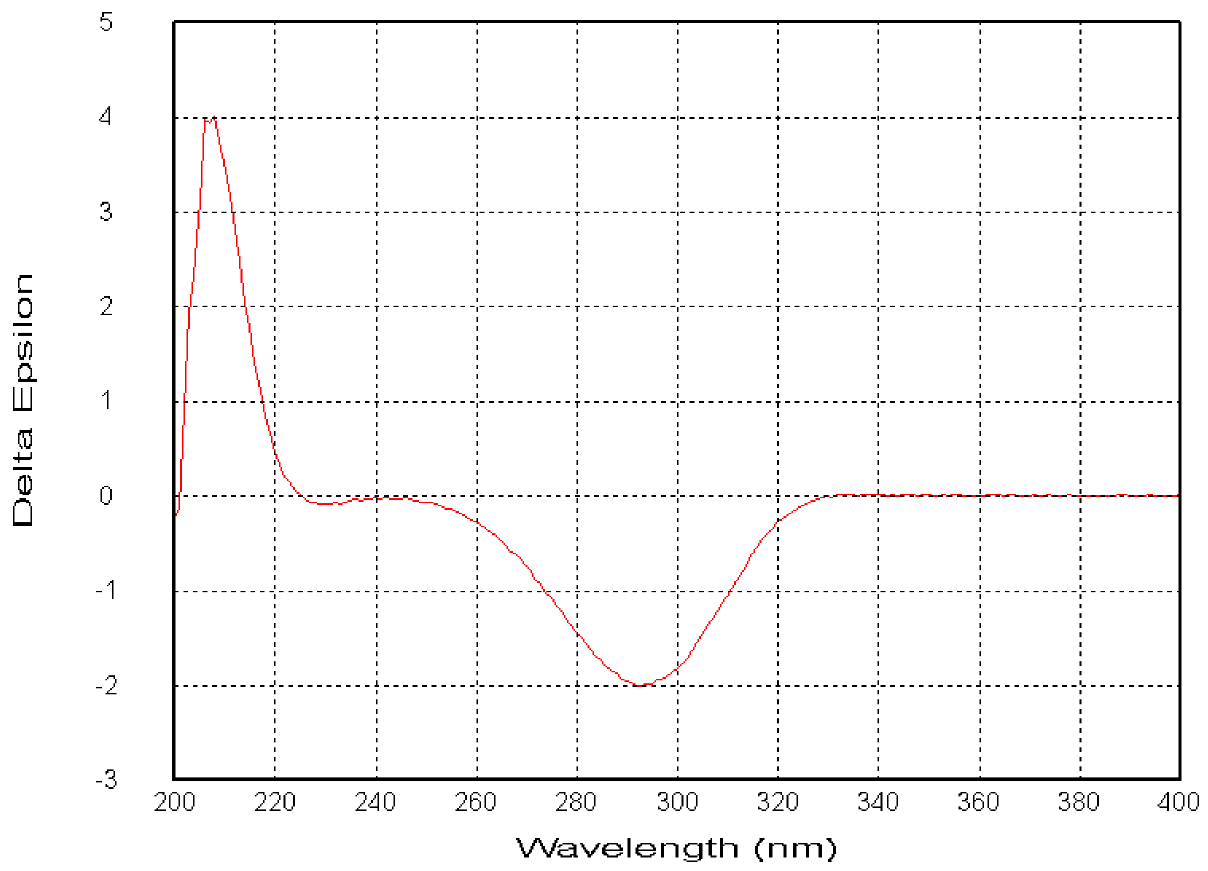

Delta Epsilon
5
4
3
2
1
0
-1
-2
-3
200
220
240
260
280
300
320
340
360
380
400
Wavelength (nm)

**Figure S101. UV and CD data for 10 in MeOH**

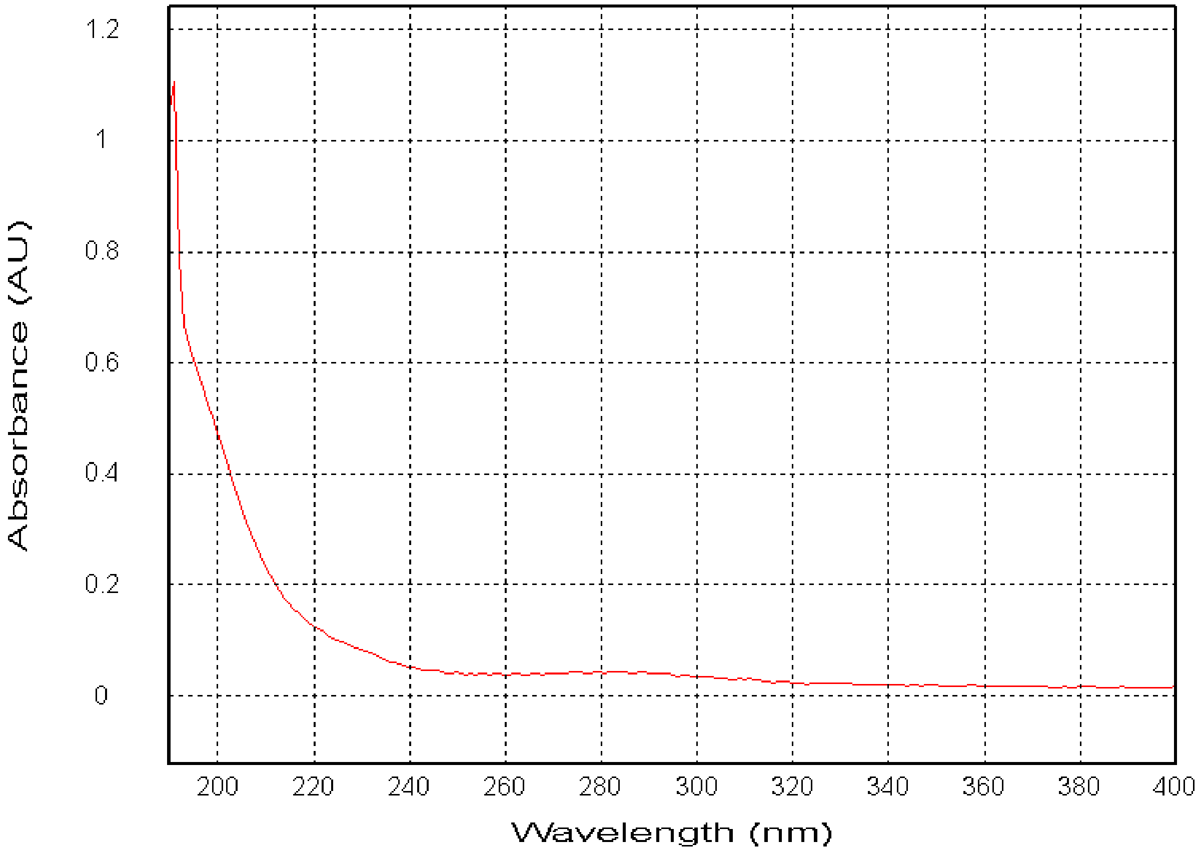

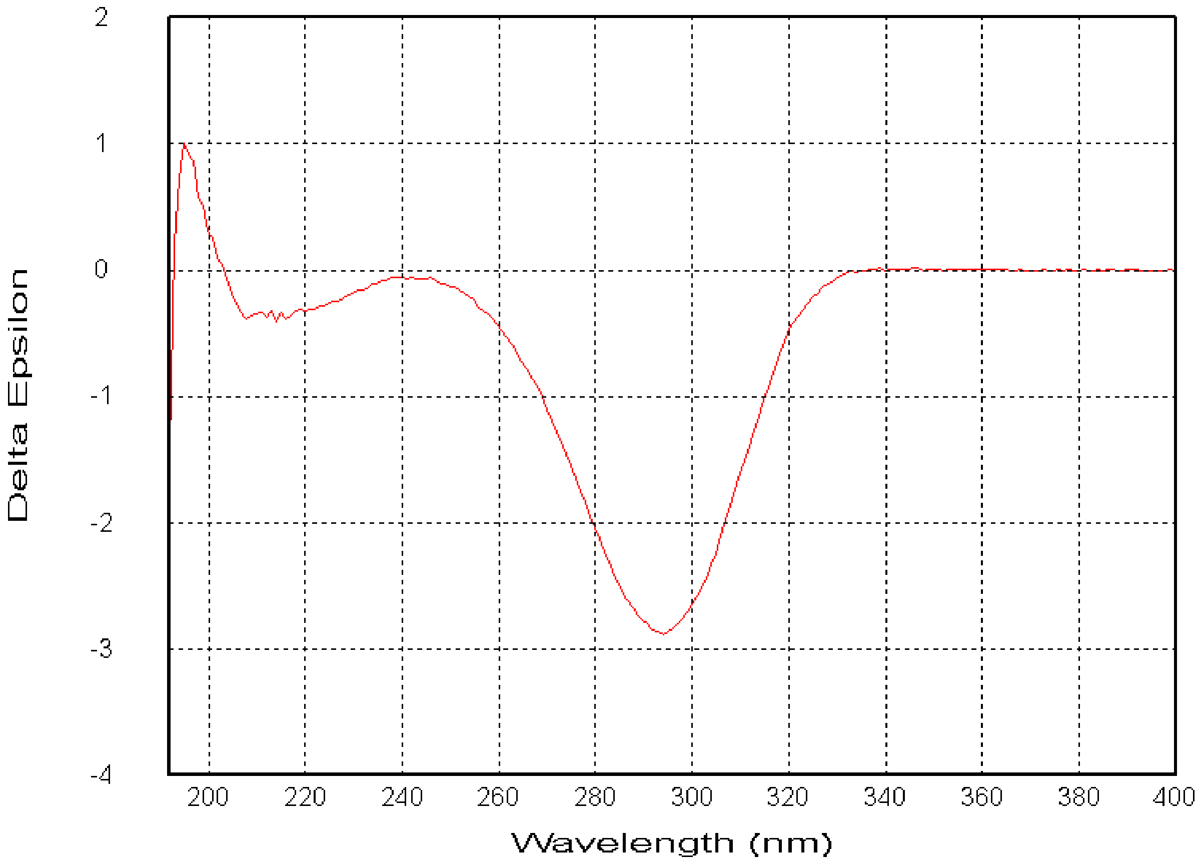

2
1
0
-1
-2
-3
-4
Delta Epsilon
200
220
240
260
280
300
320
340
360
380
400
Wavelength (nm)

**Figure S102.** $^1$H NMR spectrum of 7b ((S)-MTPA ester) measured at 700 MHz in $CDCl_3$

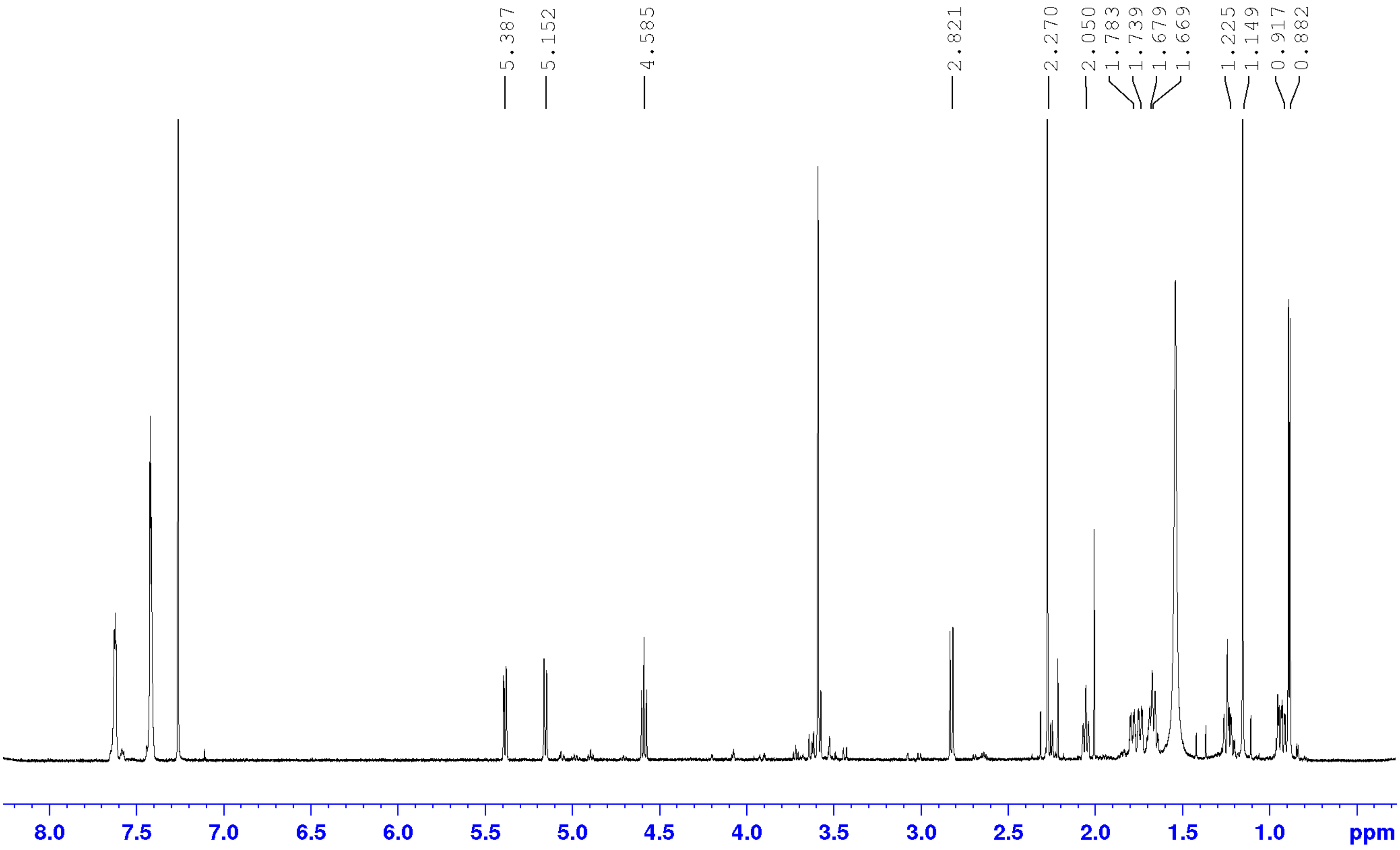

**Figure S103. $^{1}$H NMR spectrum of 7a ((R)-MTPA ester) measured at 700 MHz in $CDCl_3$**

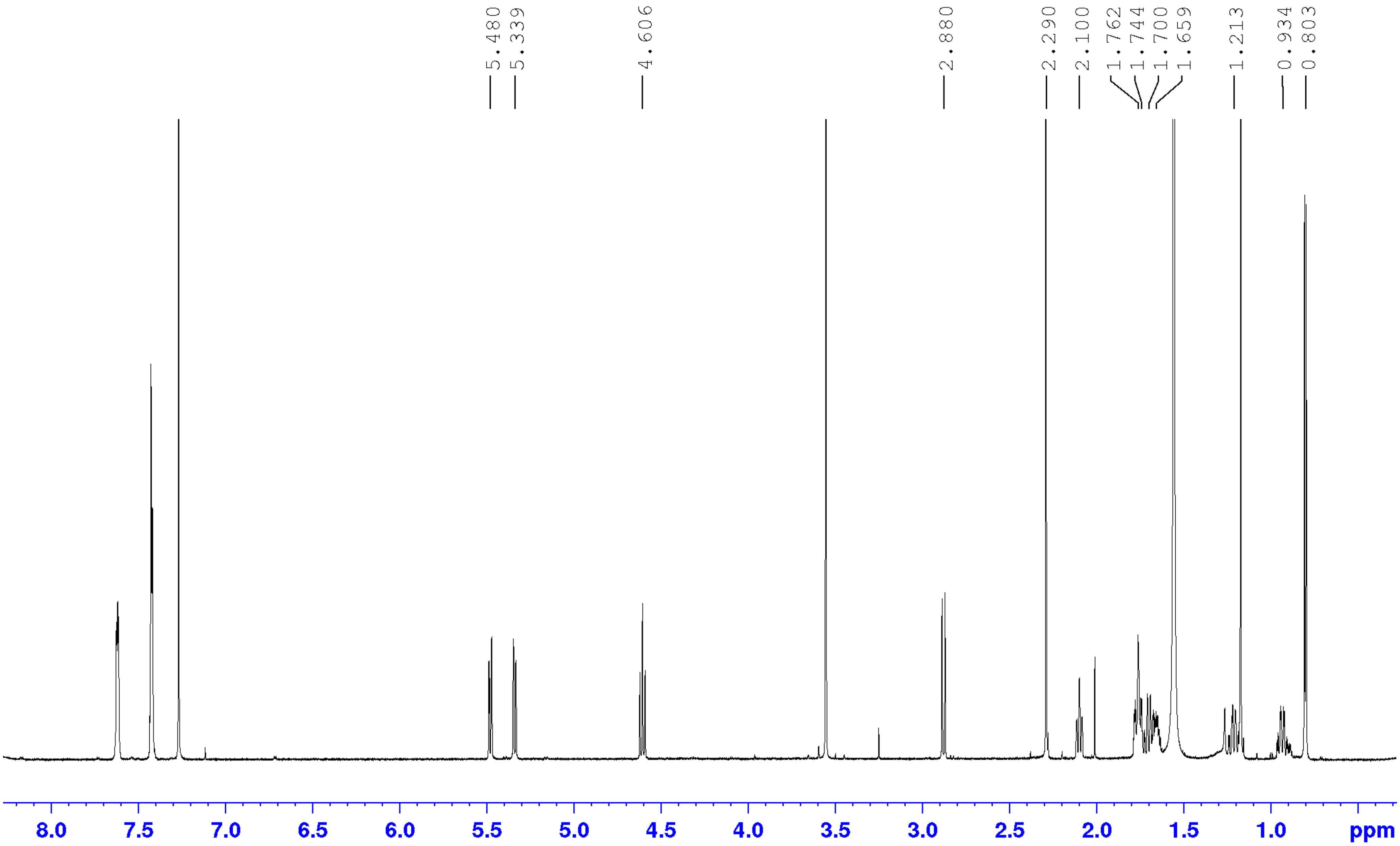

**Figure S104. HRESIMS for 7b ((S)-MTPA ester)**

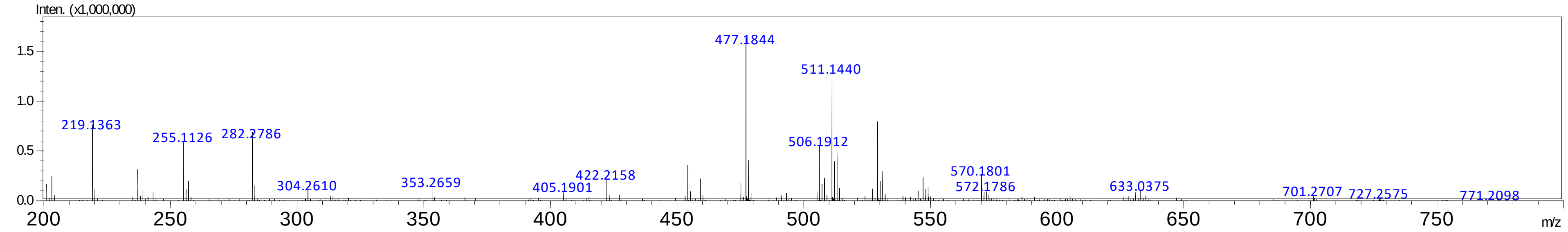


| | meas | calc | Δ (ppm) |
|---|---|---|---|
| | | | |
| $[M+Na]^+$ | 477,1844 | 477,1859 | -3,14 |

**Figure S105. HRESIMS for 7a ((R)-MTPA ester)**

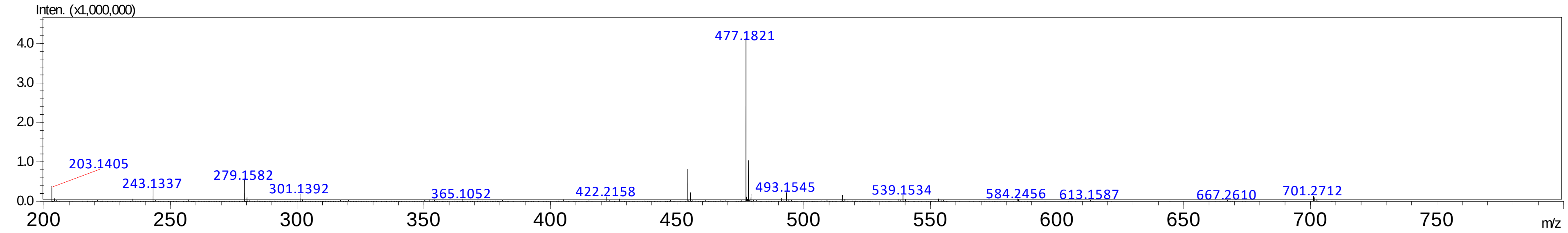


| | meas | calc | Δ (ppm) |
|---|---|---|---|
| | | | |
| $[M+Na]^+$ | 477,1821 | 477,1859 | -7,96 |

**Figure S106. ¹H NMR spectrum of 9b ((S)-MTPA ester) measured at 700 MHz in $CDCl_3$**

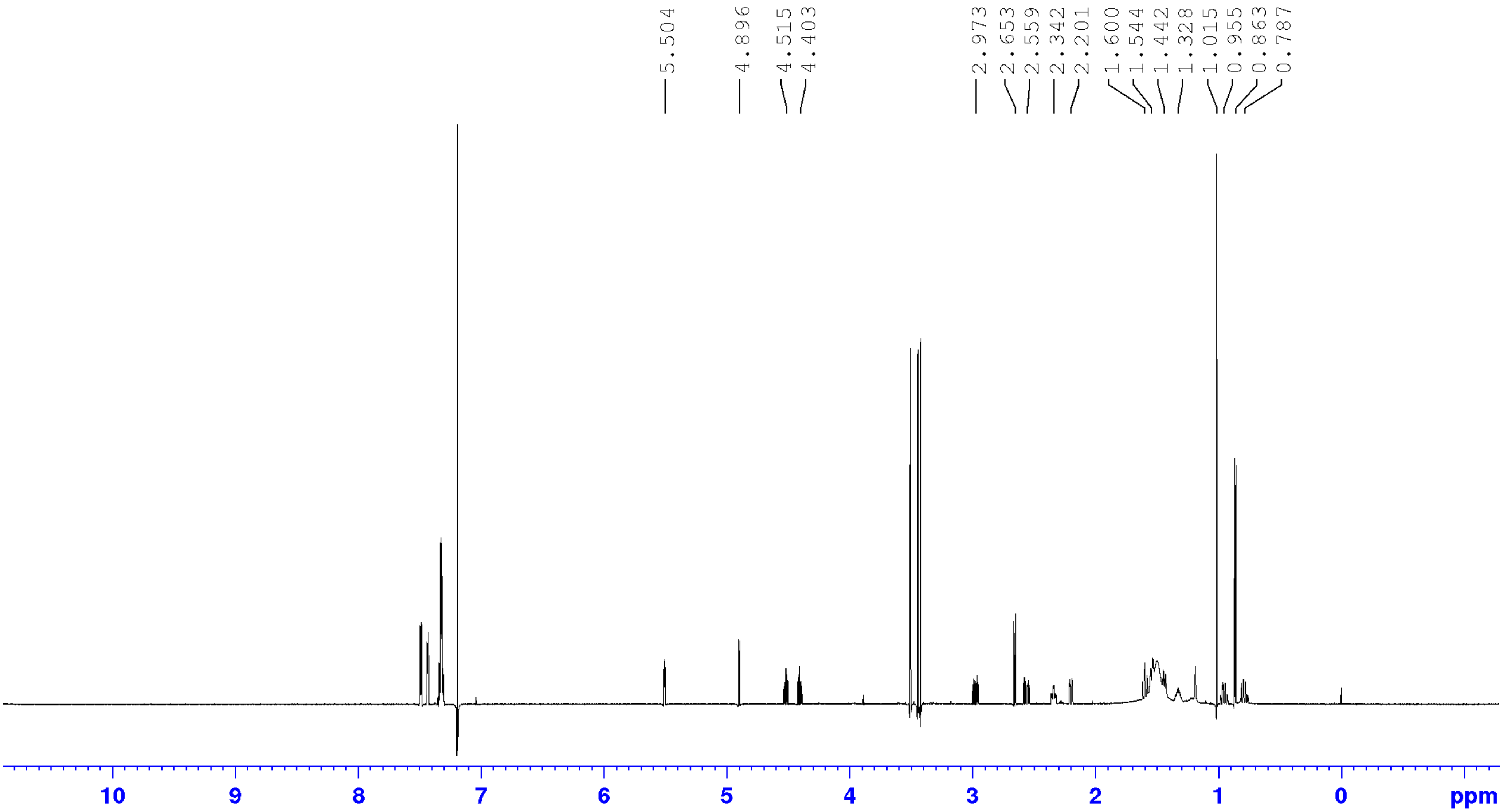

**Figure S107. ¹H NMR spectrum of 9a ((R)-MTPA ester) measured at 700 MHz in $CDCl_3$**

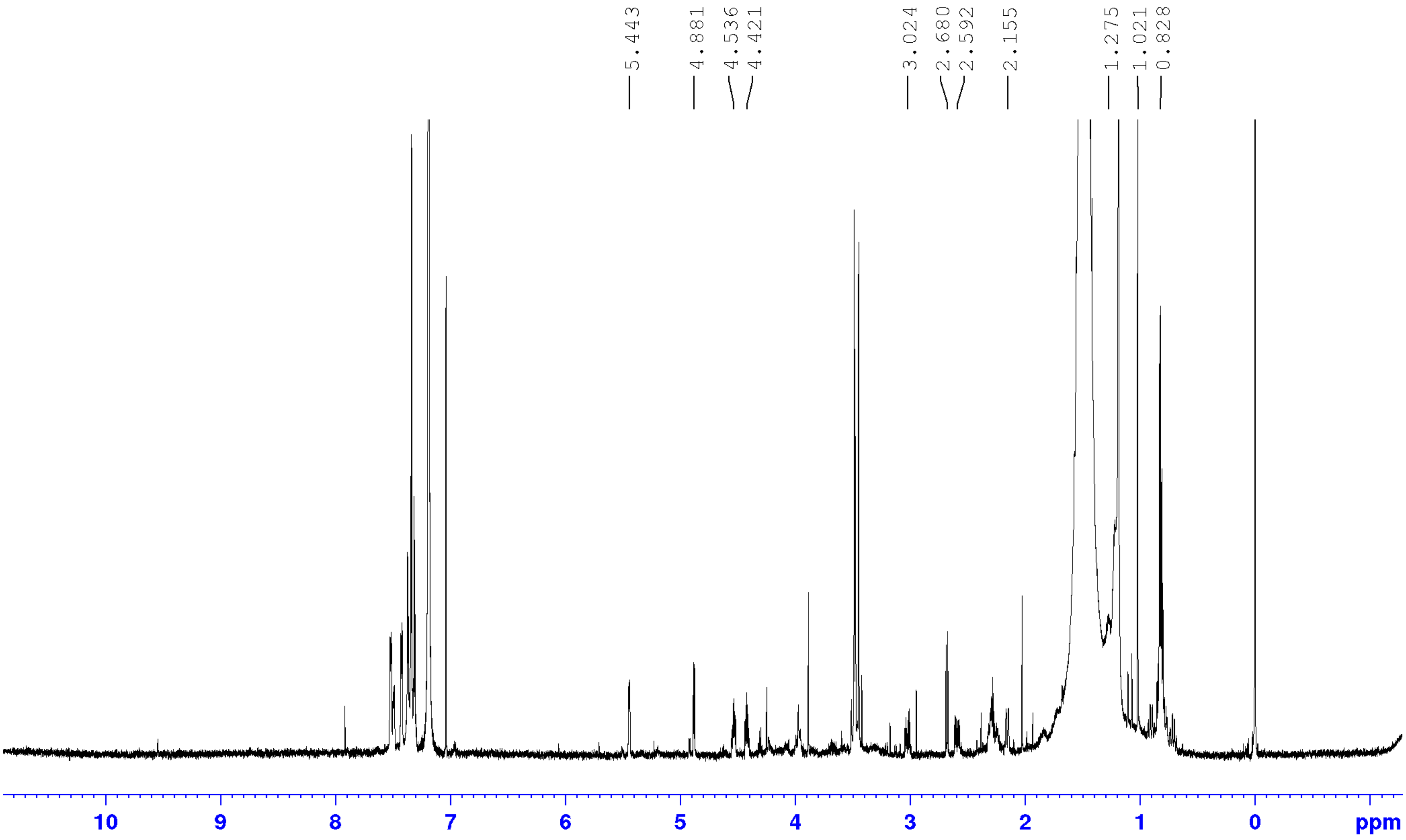

**Figure S108. ¹H NMR spectrum of 10b ((S)-MTPA ester) measured at 700 MHz in $CDCl_3$**

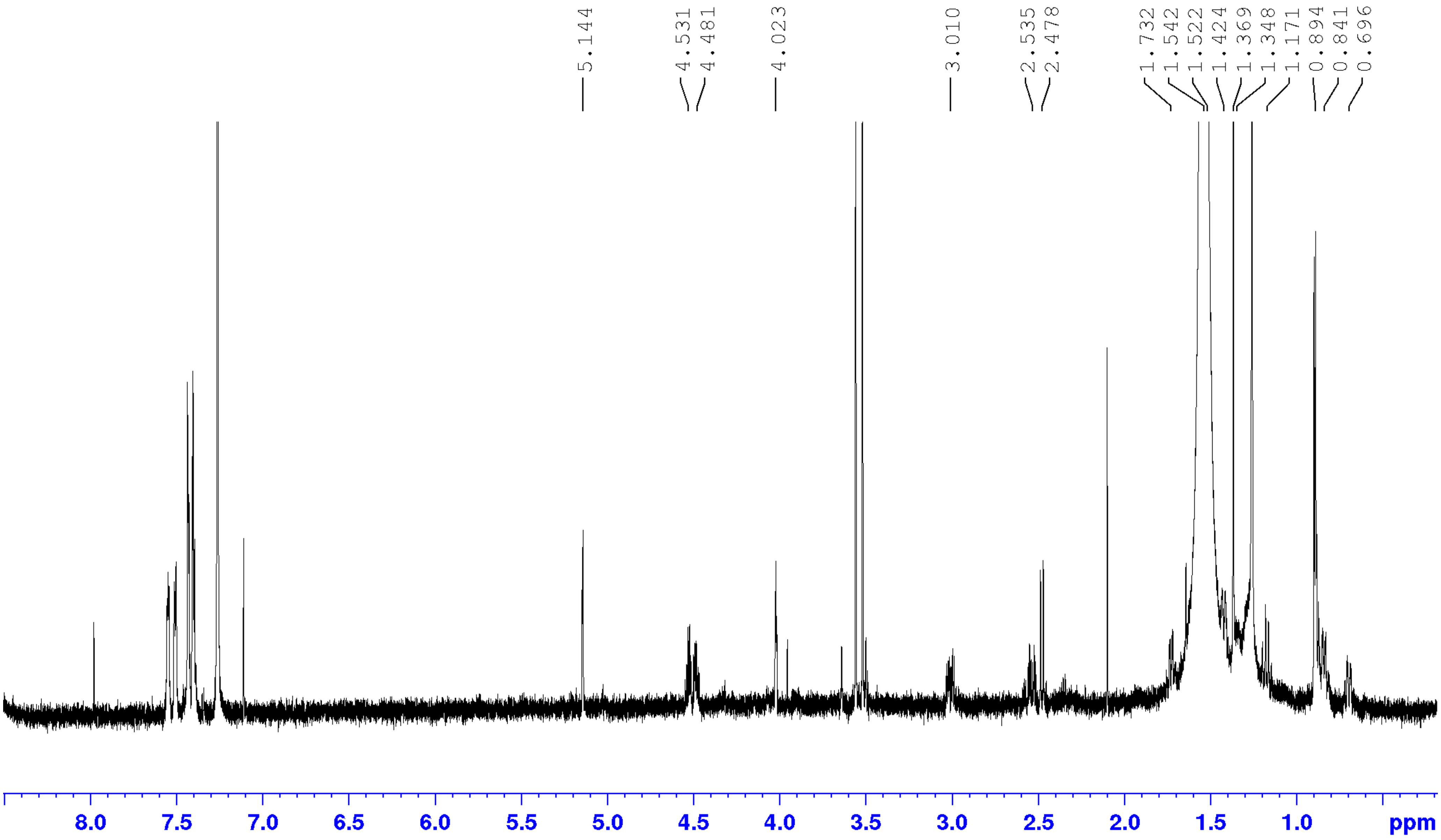

**Figure S109. HSQC spectrum of 10b ((S)-MTPA ester) measured at 700 MHz in $CDCl_3$**

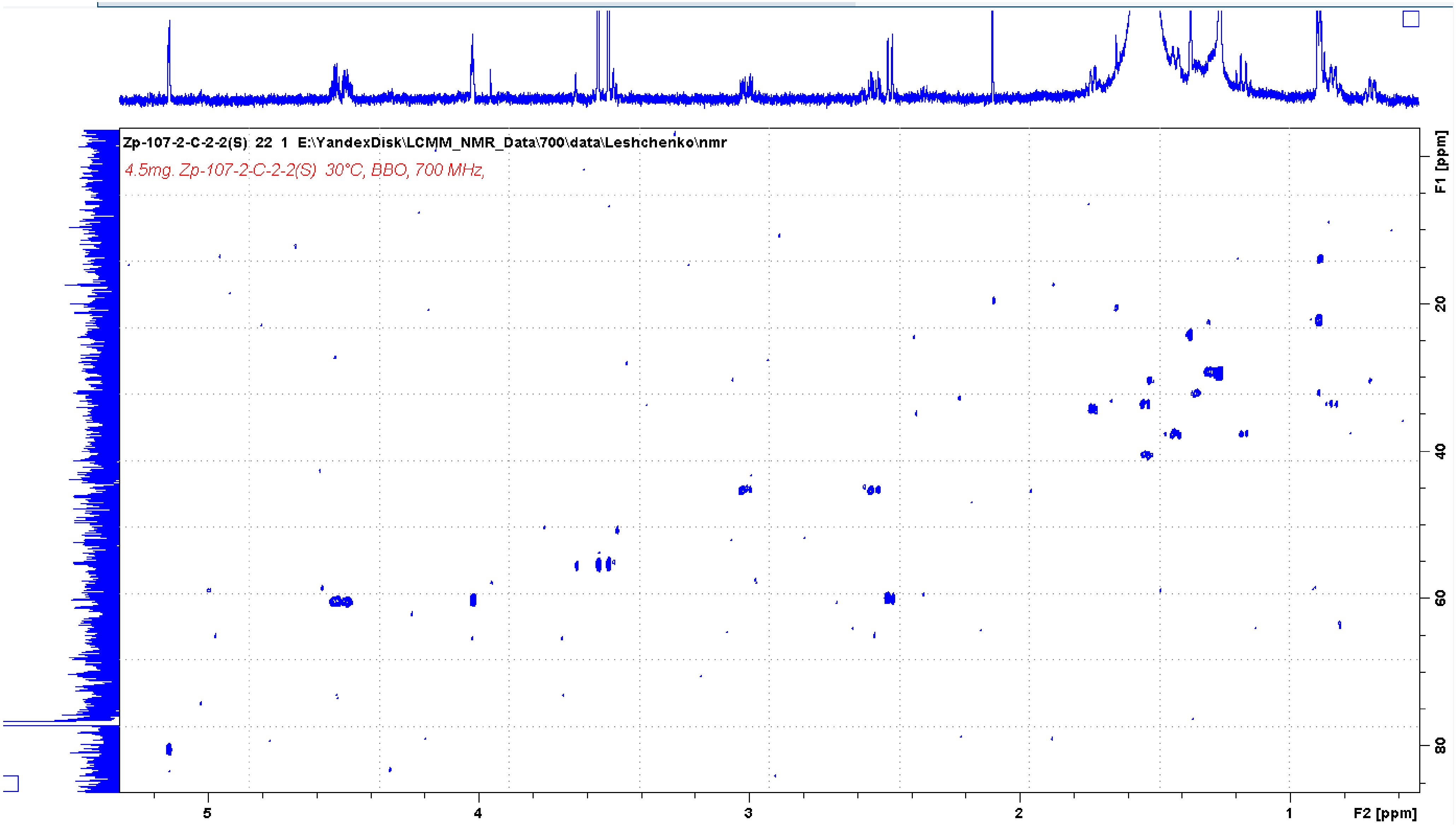

**Figure S110. $^1$H NMR spectrum of 10a ((R)-MTPA ester) measured at 700 MHz in $CDCl_3$**

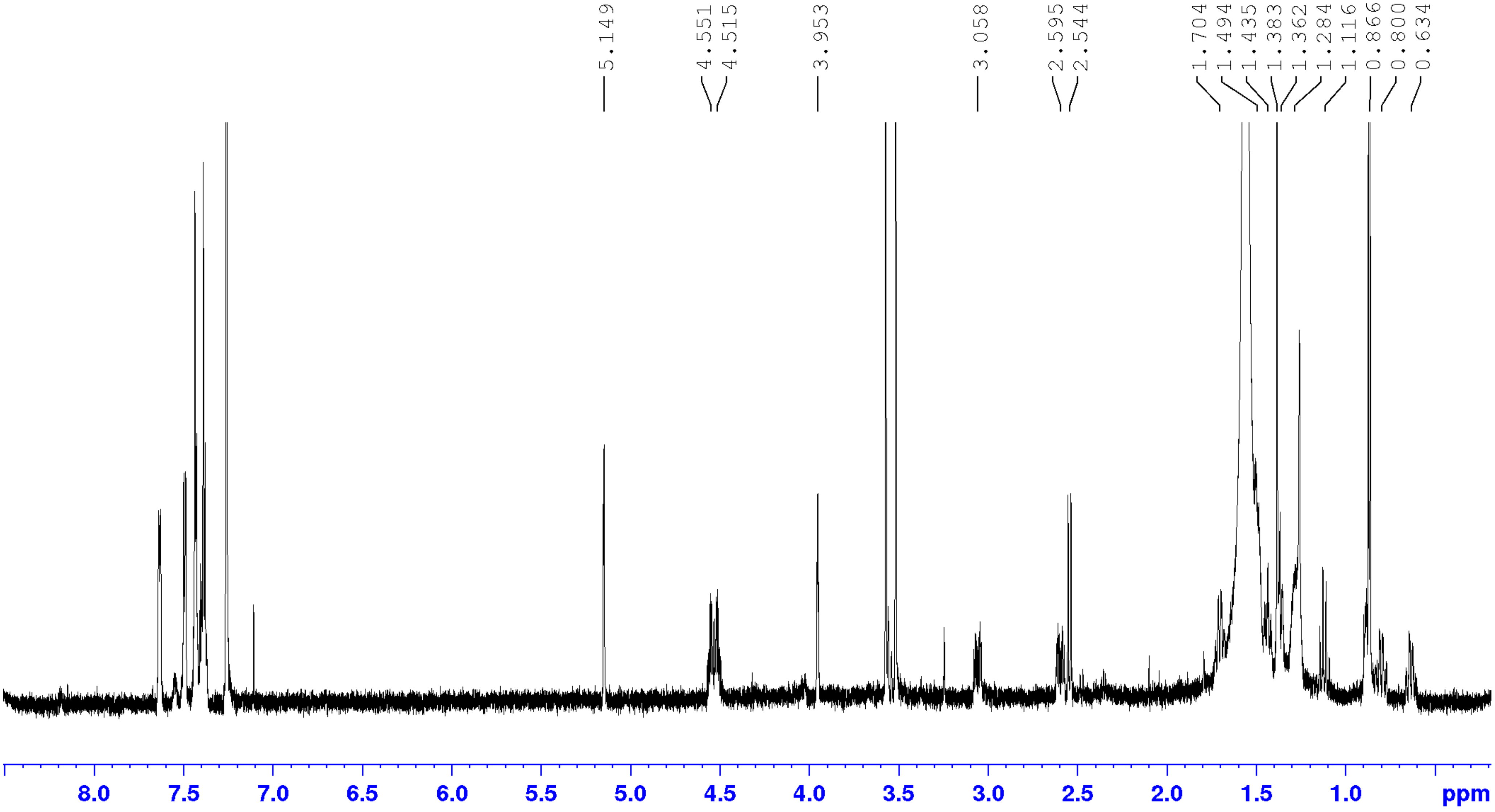

**Figure S111. HSQC spectrum of 10a ((R)-MTPA ester) measured at 700 MHz in $CDCl_3$**

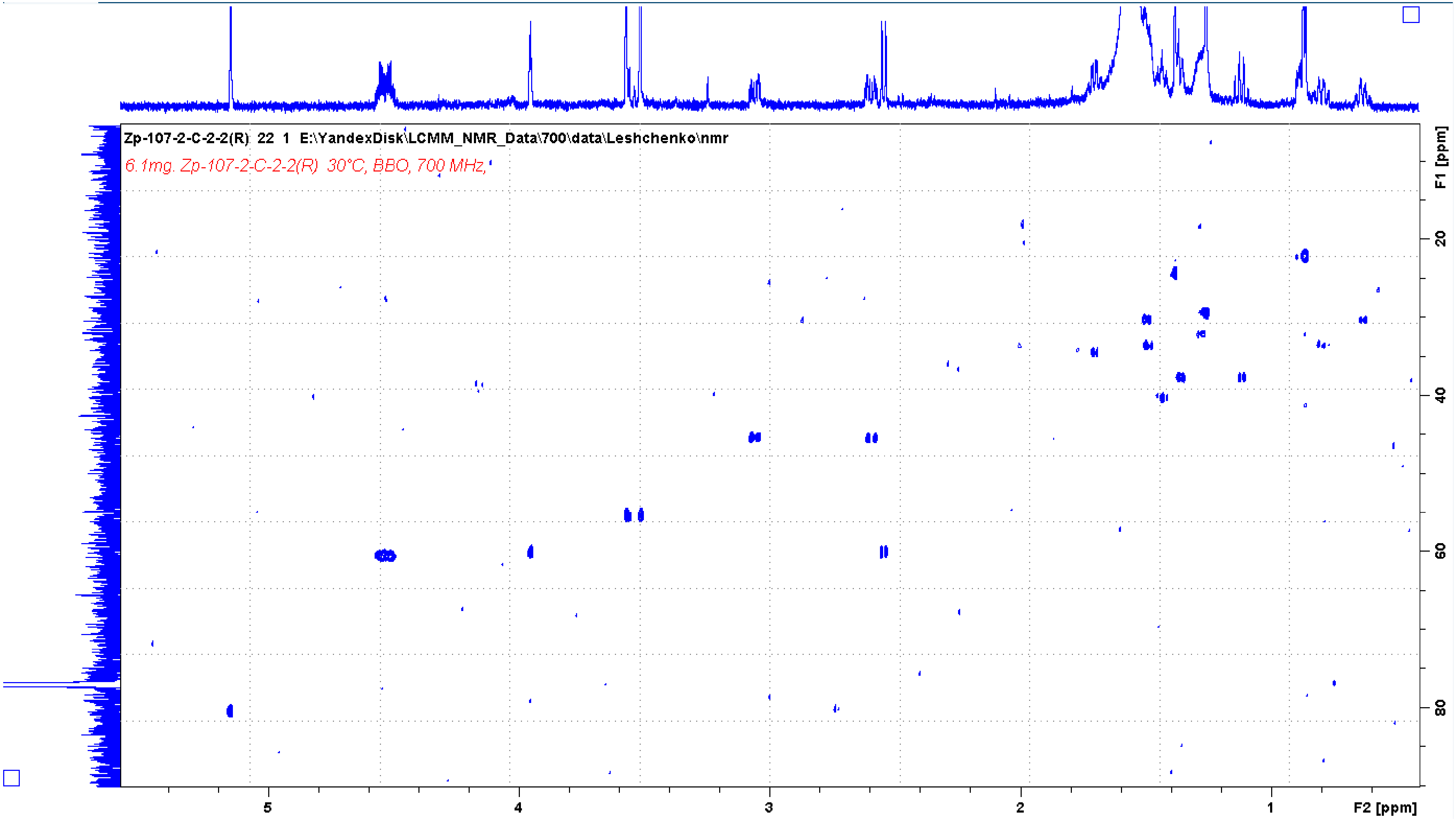

**Figure S112. HRESIMS for 10b ((S)-MTPA ester)**

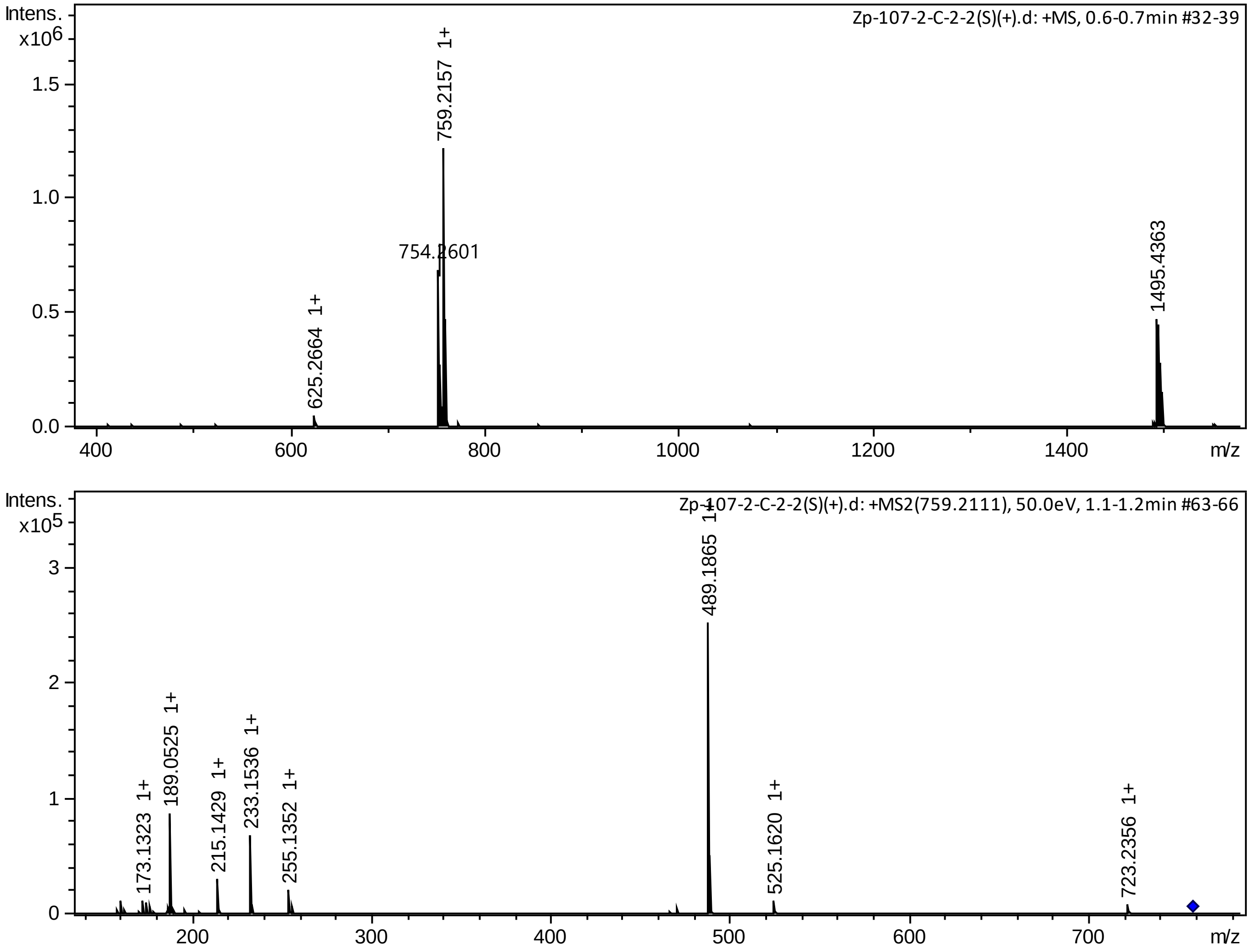

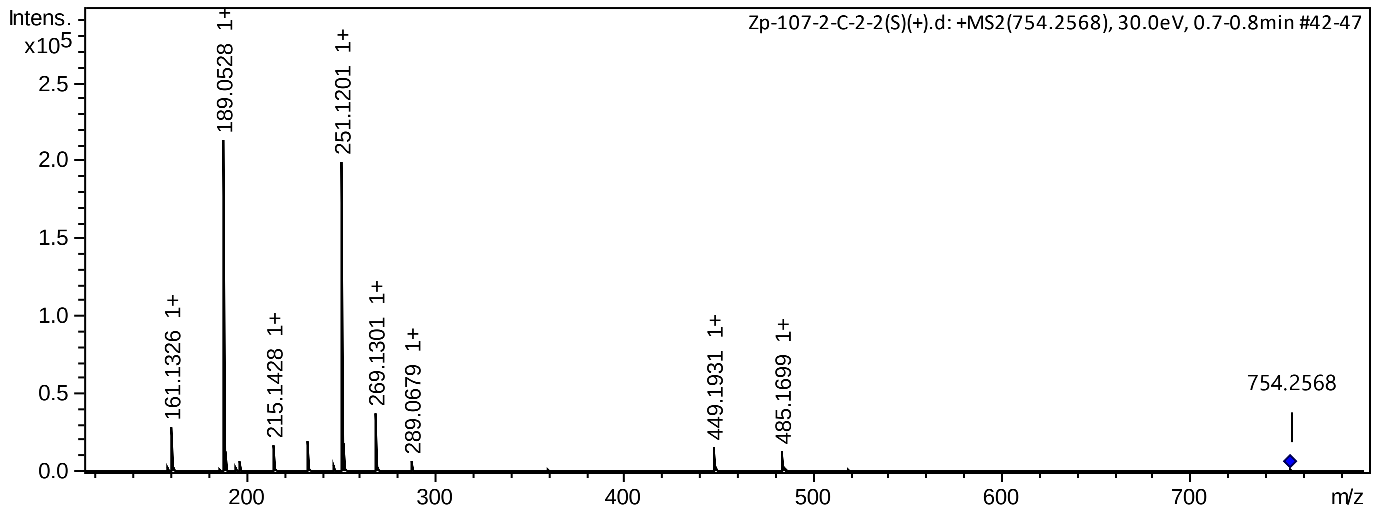


| | meas | calc | Δ (ppm) |
|---|---|---|---|
| $[M+Cl]^-$ | 771,1933 | 771,1932 | -0,1 |
| $[M+NH4]^+$ | 754,2601 | 754,2552 | -6,5 |
| $[M+Na]^+$ | 759,2157 | 759,213 | -3,6 |

**Figure S113. HRESIMS for 10a ((R)-MTPA ester)**

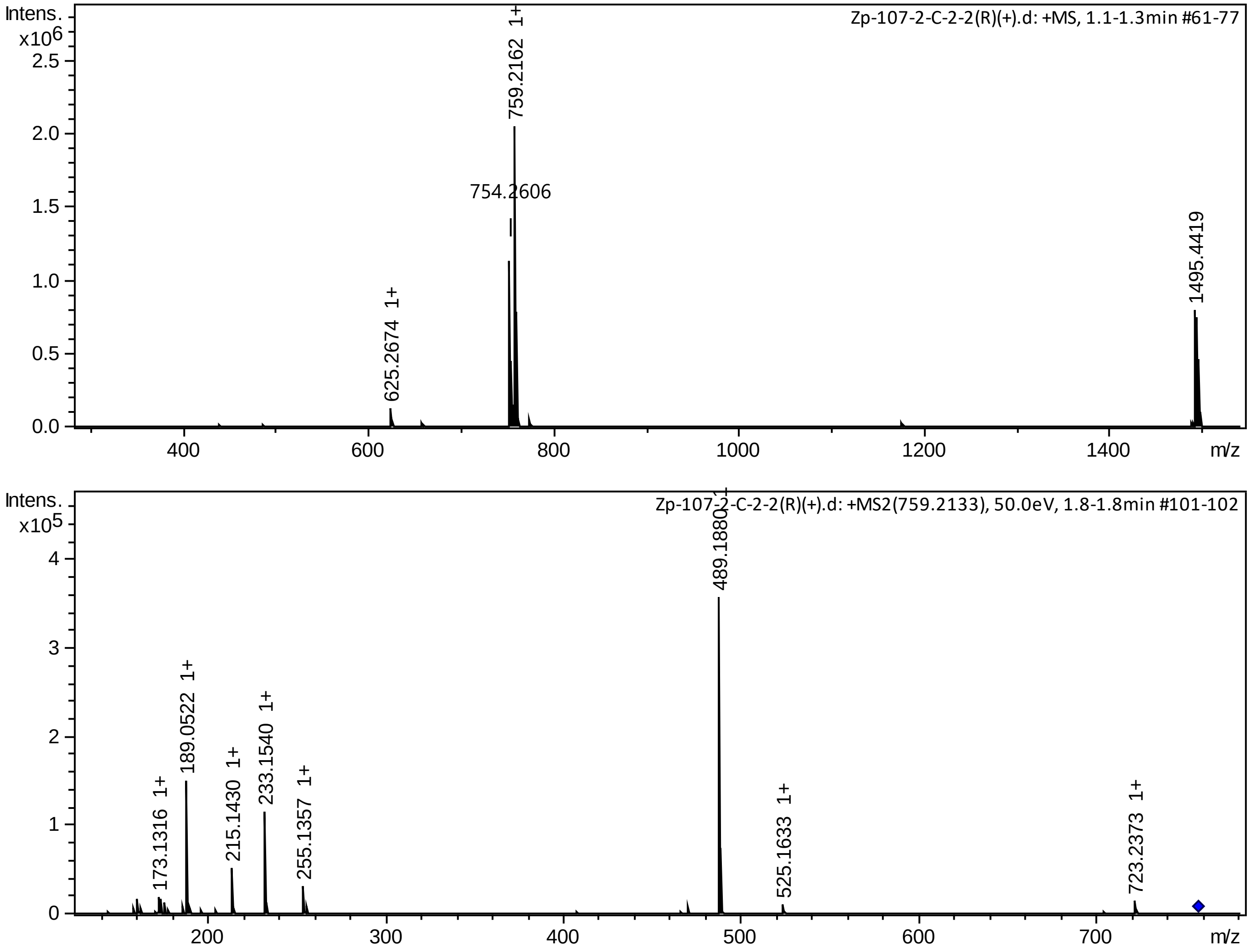

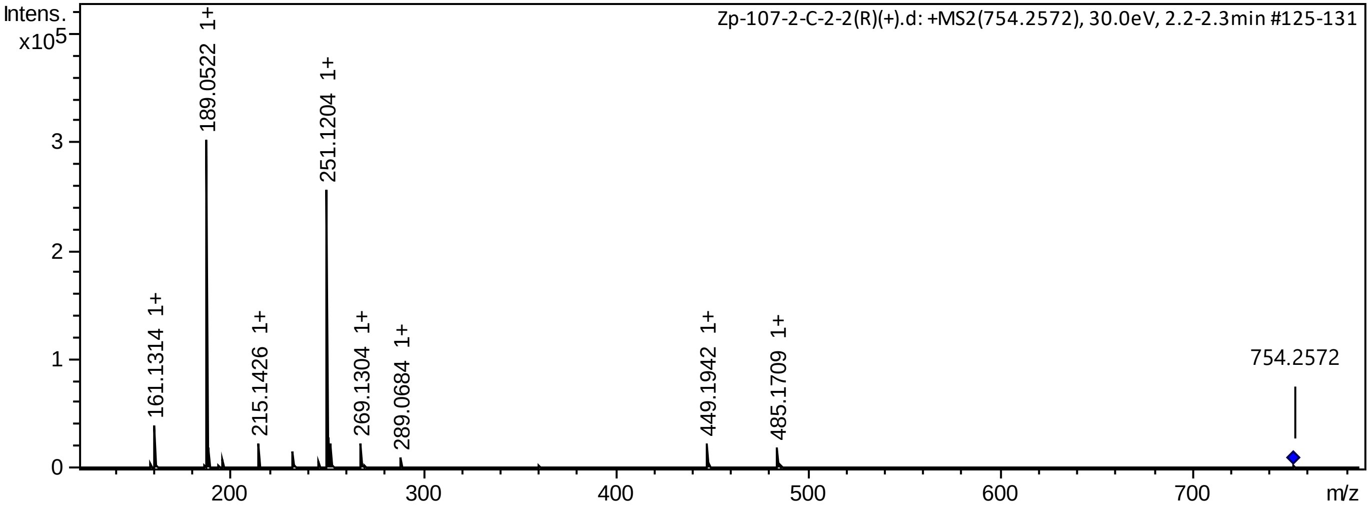


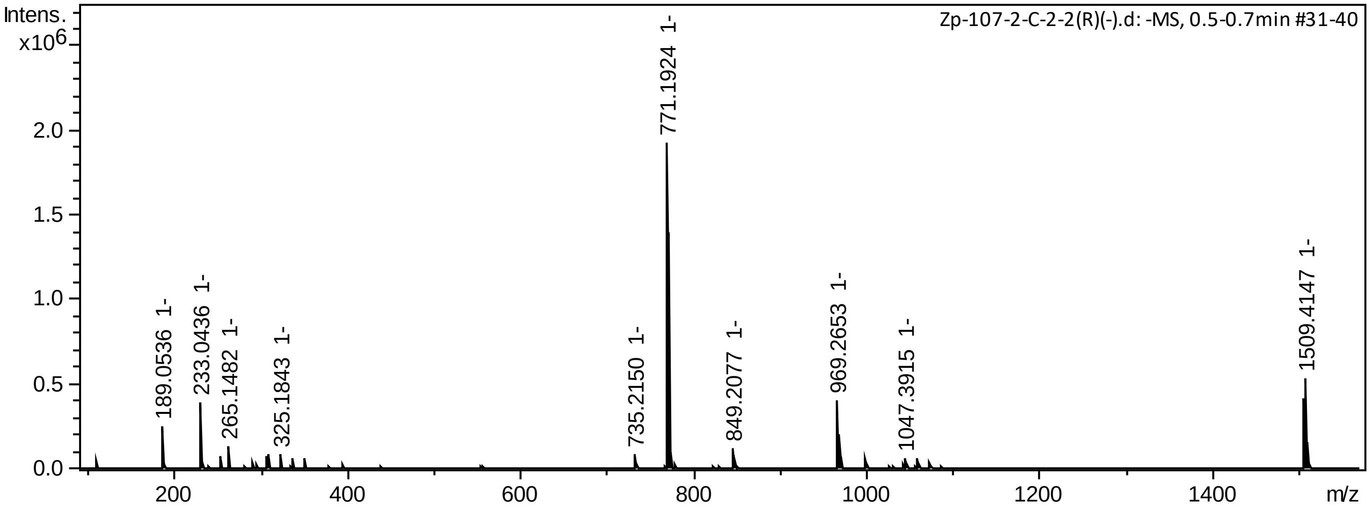


| | meas | calc | Δ (ppm) |
|---|---|---|---|
| $[M+Cl]^-$ | 771,1924 | 771,1932 | 1,0 |
| $[M+NH4]^+$ | 754,2606 | 754,2552 | -7,2 |
| $[M+Na]^+$ | 759,2162 | 759,213 | -4,2 |

**Figure S114. The main conformations of 1**

| | | |
|---|---|---|
| 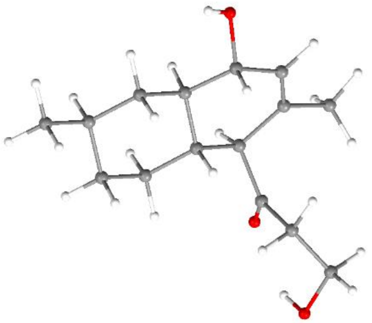<br>[ 0.05 ]  θ ≈ 0° | 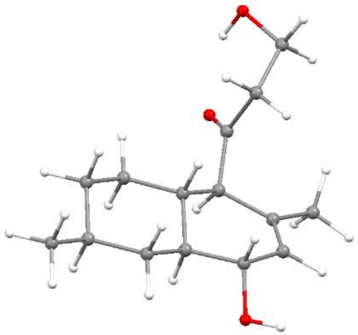<br>[ 0.03 ]  θ ≈ 0° | 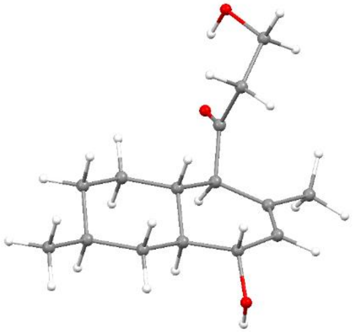<br>[ 0.19 ]  θ ≈ 0° |
| 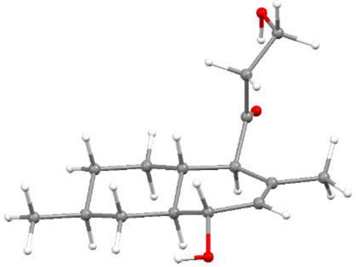<br>[ 0.01 ]  θ ≈ 0° | 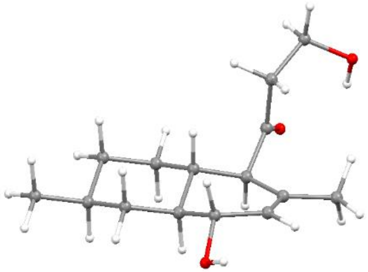<br>[ 0.02 ]  θ ≈ 0° | 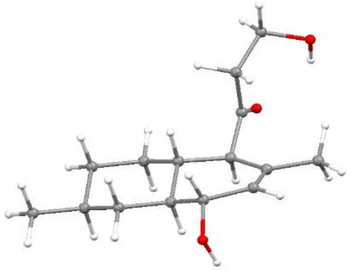<br>[ 0.07 ]  θ ≈ 0° |
| | | |
| 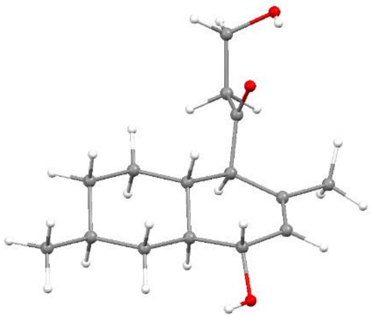<br>[ 0.07 ]  θ ≈ 180° | 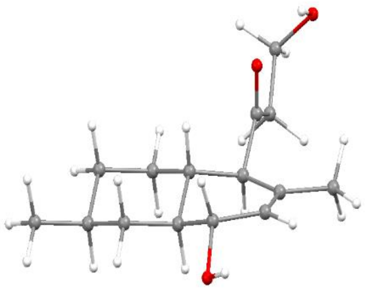<br>[ 0.05 ]  θ ≈ 180° | 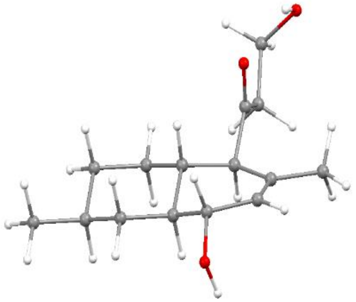<br>[ 0.24 ]  θ ≈ 180° |

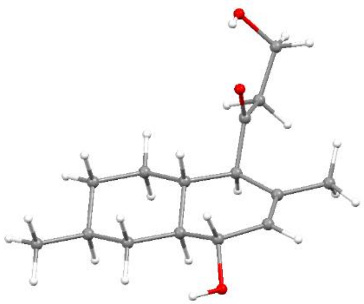

[ 0.06 ] $\theta \approx 180^{\circ}$

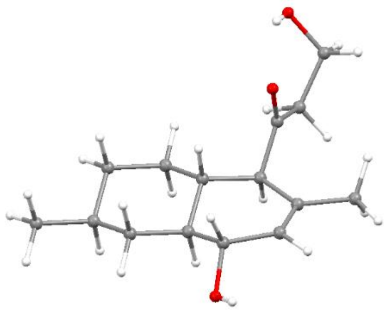

[ 0.04 ] $\theta \approx 180^{\circ}$

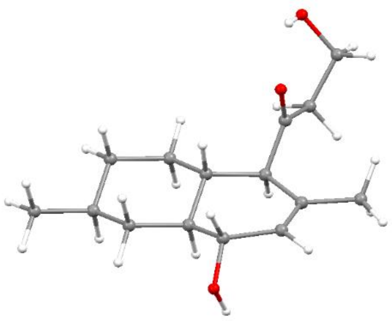

[ 0.17 ] $\theta \approx 180^{\circ}$

*the statistical weights are in square brackets

**Figure S115. ECD spectra, calculated for different conformations of 1.**

The ratio of intensities of bands at $\lambda \approx 210$ nm and $\lambda \approx 290$ nm in the total ECD spectrum is determined by the ratio, in which conformations $\theta \approx 0^{\circ}$ and $\theta \approx 180^{\circ}$ are presented in the sample. It is seen that theory based on PCM approach somewhat overestimates the percentage of $\theta \approx 180^{\circ}$ conformations. The same situation was obtained for compound **3** (see Figure S116)

We used the notation $\theta = \angle$O–C(3)–C(4) –H(4).

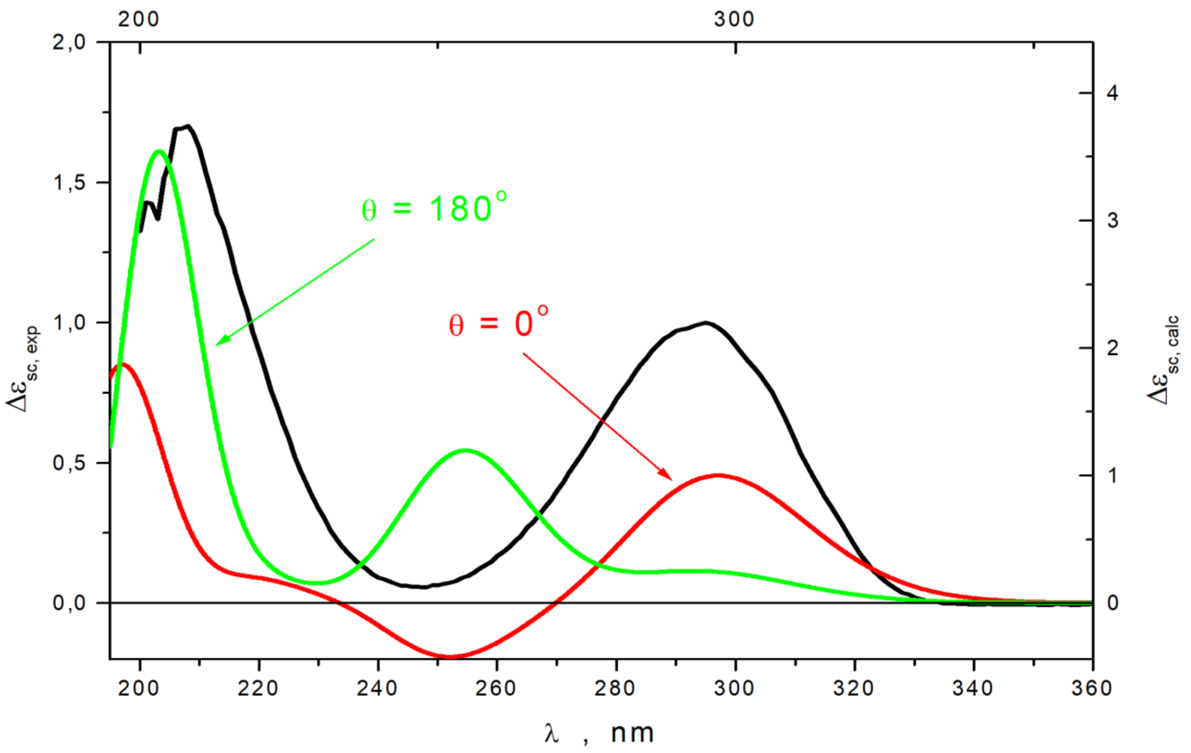

**Figure S116. ECD spectra, calculated for different conformations of 3.**

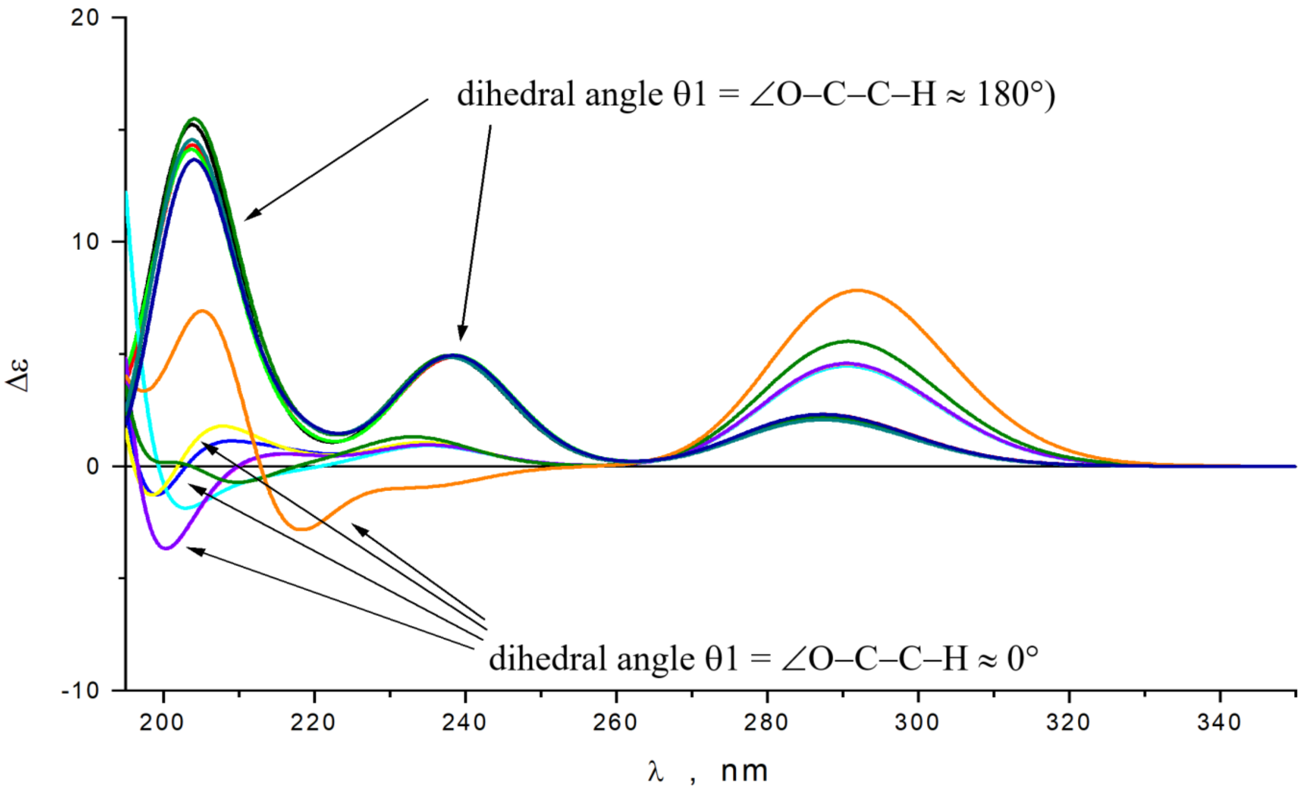


Δε

**Figure S117. ECD spectra, simulated for different relative amounts of conformations with dihedral angle θ1 = ∠O–C–C–H ≈ 180° and θ1 = ∠O–C–C–H ≈ 0°**

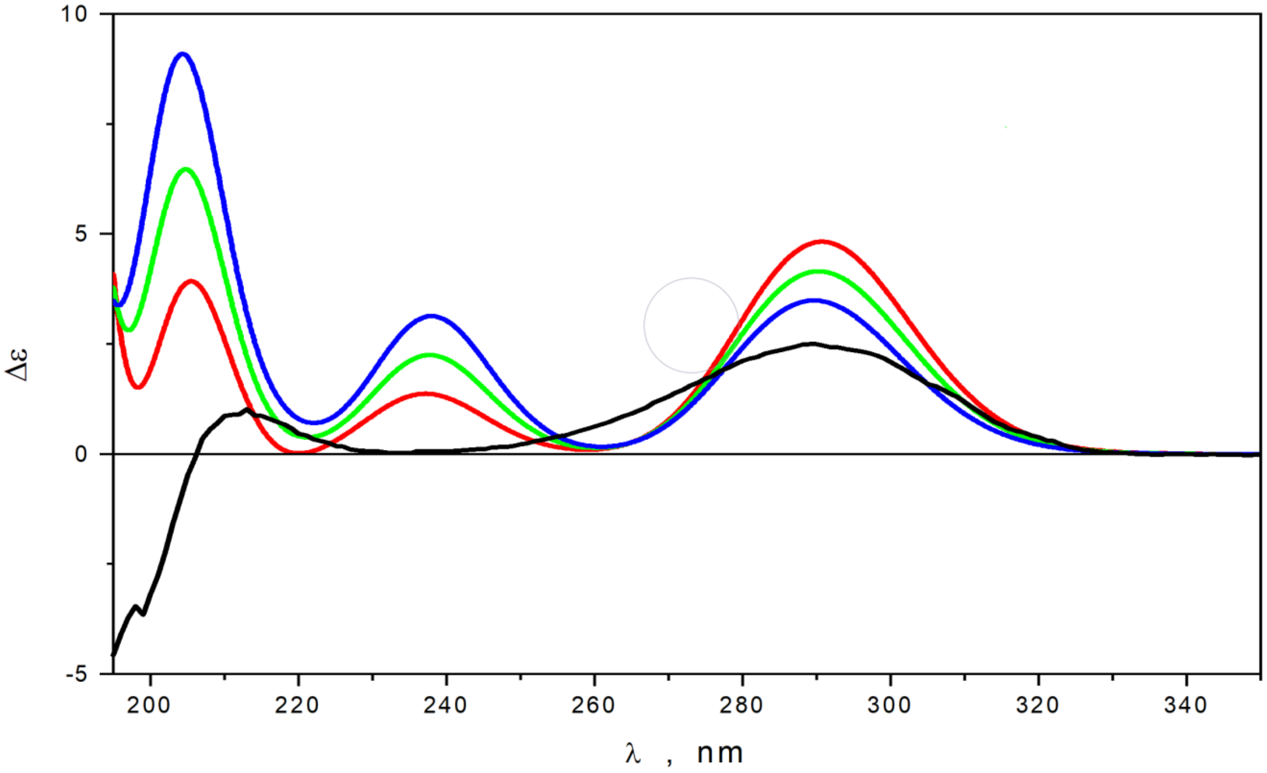


**Figure S118. HPLC-MS/MS Analysis**

The following mobile phases were used for the high pressure gradient: A, methanol+0.1% formic acid; B, water+0.1% formic acid. Eluent was pumped at a constant flow 0.2 mL/min with gradient (A:B, % by vol.): 80:20. Analysis time was 5 min. Injection volume was 5μL.

The MS parameters were as follows: temperature of the heat block, and desolvatation line was 200, and 250 °C, respectively. Flow rate of nebulizing and dry gases (N2) was 1.5 and 10 L/min, respectively. The capillary voltages were 4.5/-3.5 kV (for positive / negative ionization mode). The MS1 scan range was set at m/z 200-800. The MS2 scan range was set at m/z 100-800, CID energy was 50%

**Figure S119. Key HMBC (blue arrows) and 1H-1H COSY (bold lines) correlations of 1–10.**

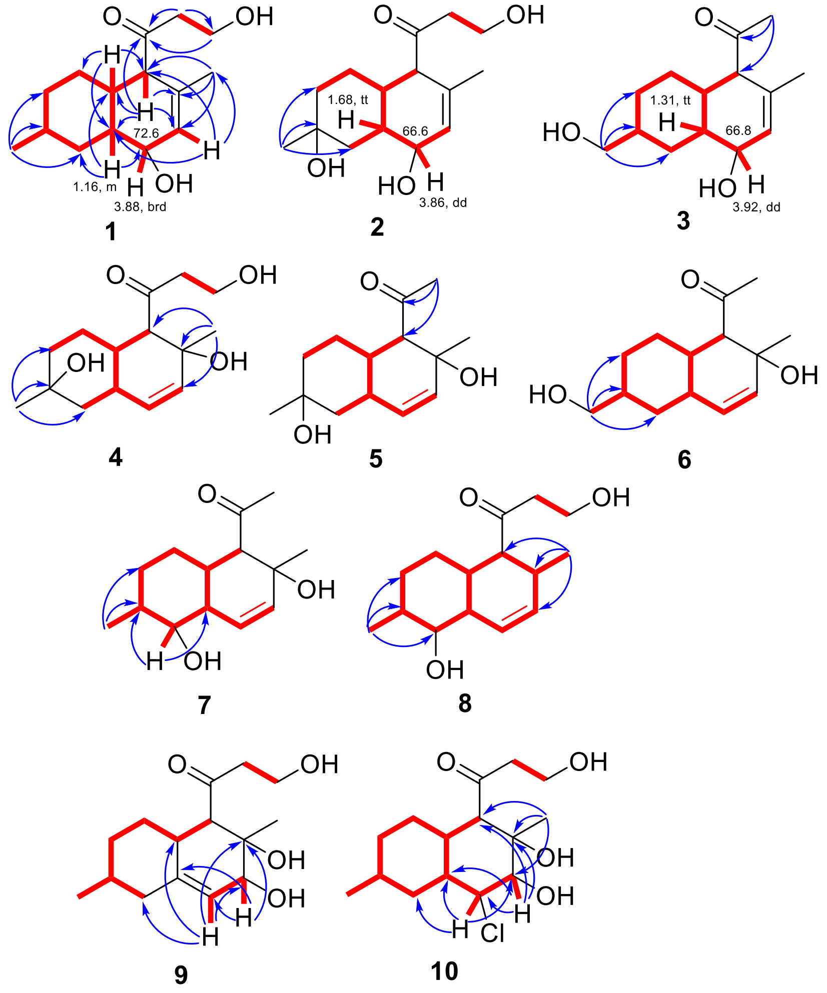

**Figure S120. HRESIMS for 9b ((S)-MTPA ester)**

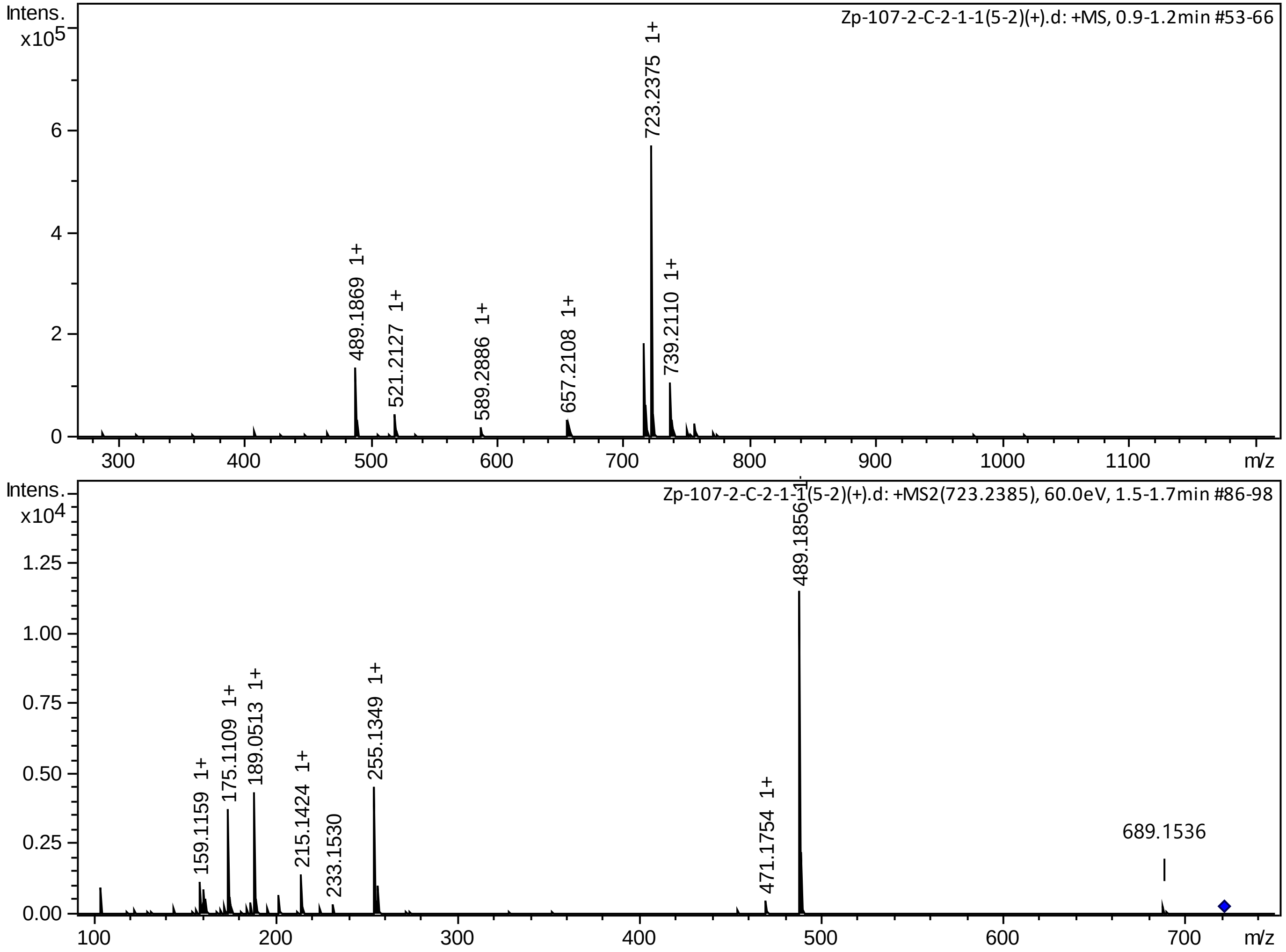


| | meas | calc | Δ (ppm) |
|---|---|---|---|
| [M+Cl]$^-$ | 735,2157 | 735,2165 | 1,1 |
| | | | |
| [M+Na]$^+$ | 723,2375 | 723,2363 | -1,7 |

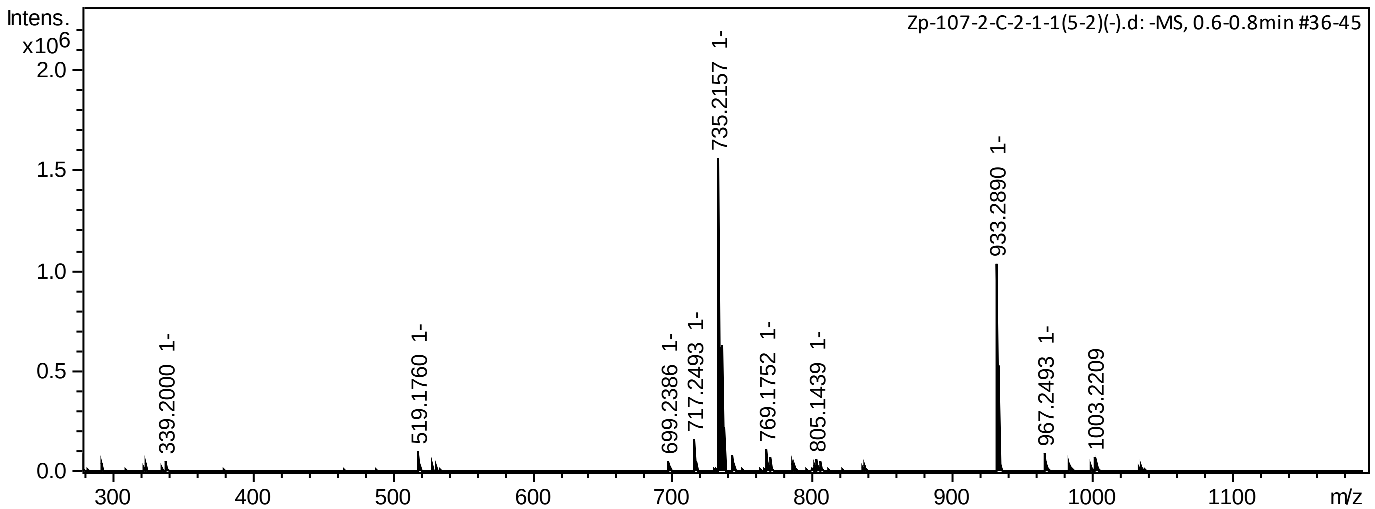
Intens.
x10^6
2.0
1.5
1.0
0.5
0.0
Zp-107-2-C-2-1-1(5-2)(-).d: -MS, 0.6-0.8min #36-45
339.2000 1-
519.1760 1-
699.2386 1-
717.2493 1-
735.2157 1-
769.1752 1-
805.1439 1-
933.2890 1-
967.2493 1-
1003.2209
300
400
500
600
700
800
900
1000
1100
m/z

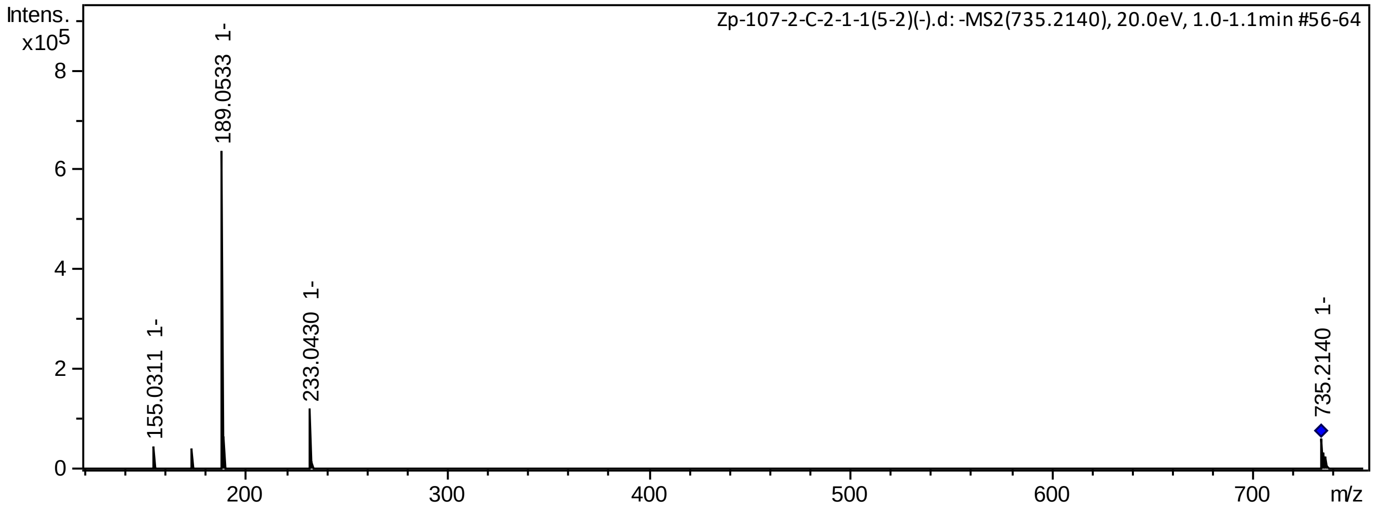
Intens.
x10^5
8
6
4
2
0
Zp-107-2-C-2-1-1(5-2)(-).d: -MS2(735.2140), 20.0eV, 1.0-1.1min #56-64
155.0311 1-
189.0533 1-
233.0430 1-
735.2140 1-
200
300
400
500
600
700
m/z

**Figure S121. HRESIMS for 9a ((R)-MTPA ester)**

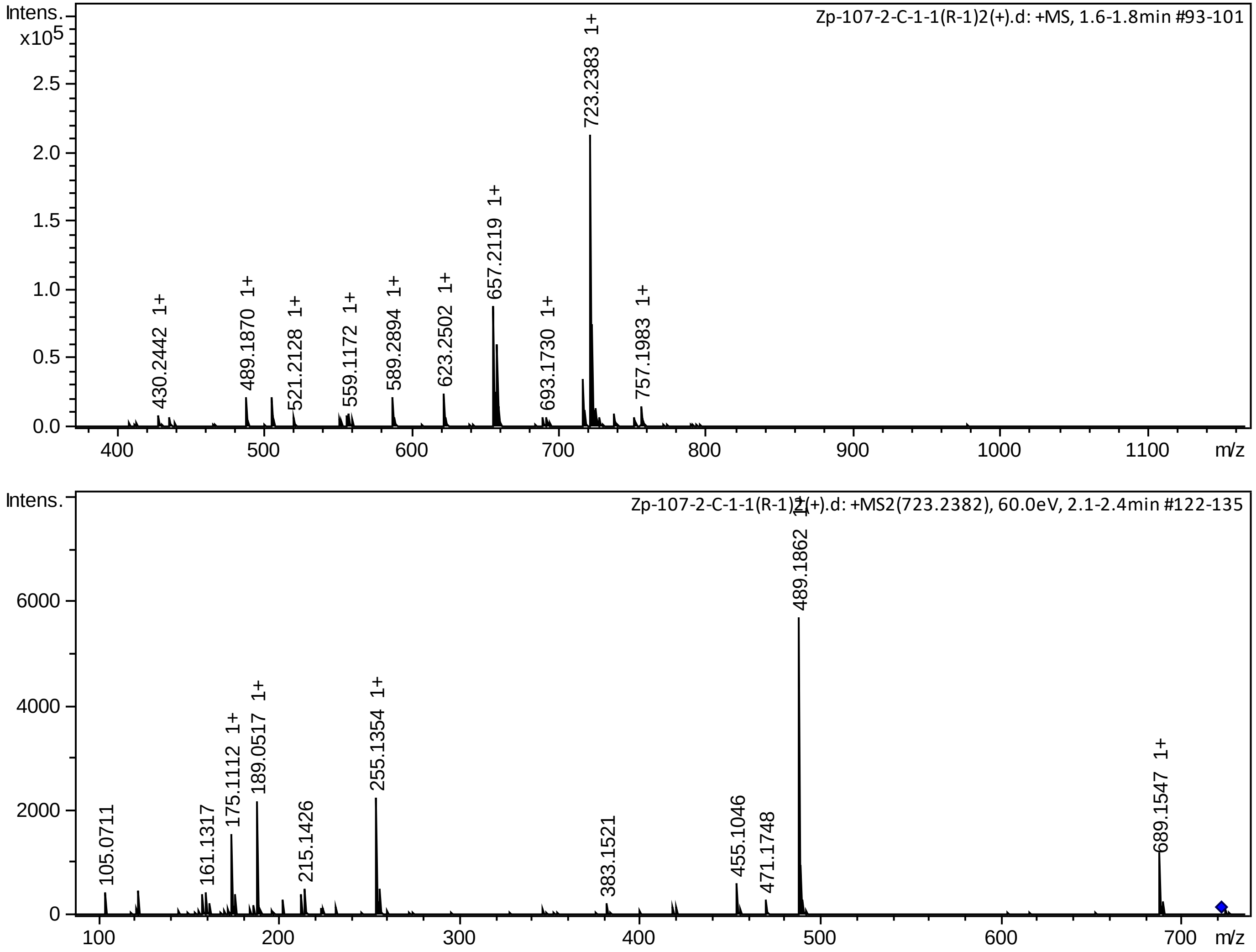

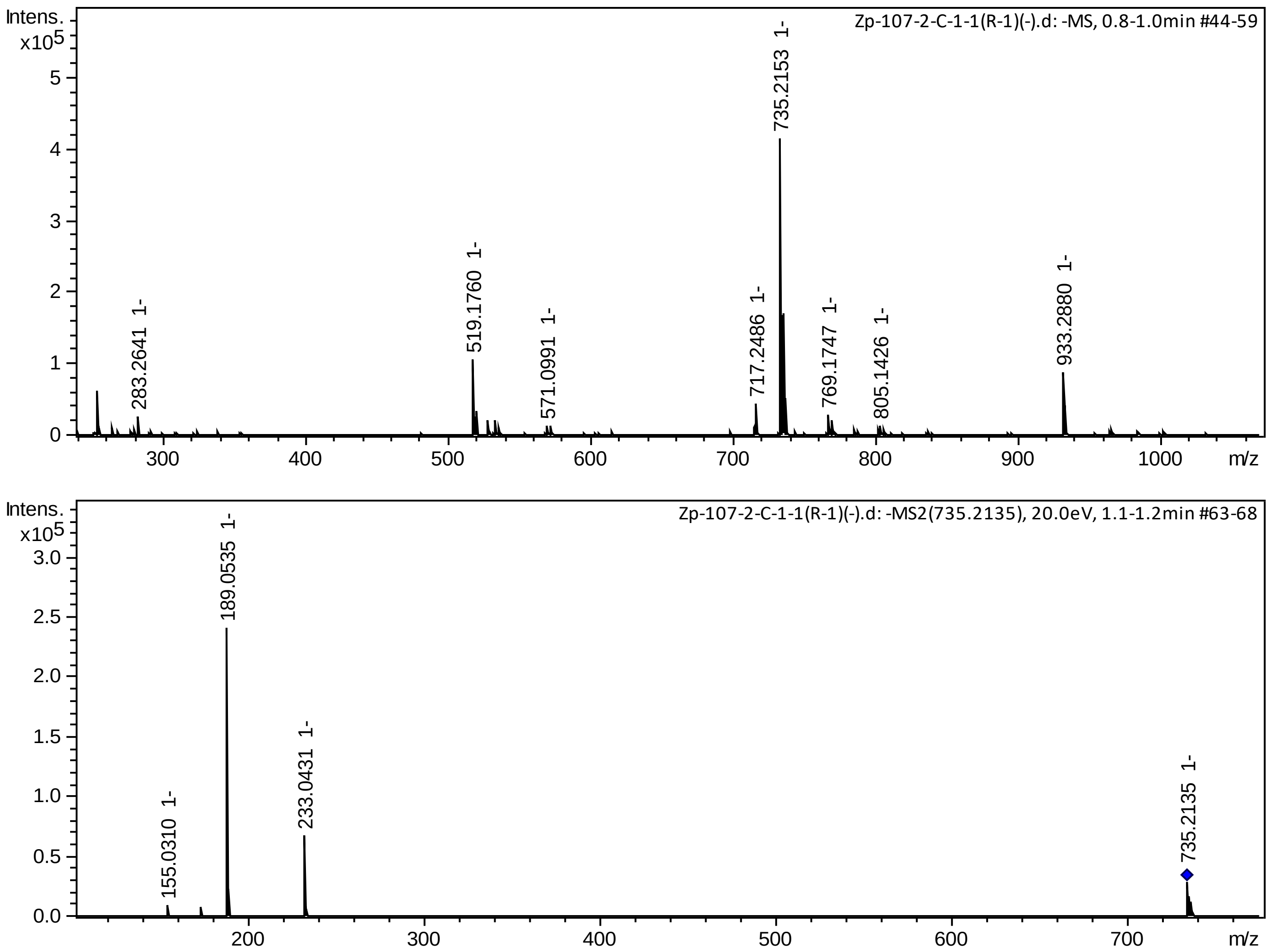


| | meas | calc | Δ (ppm) |
|---|---|---|---|
| [M+Cl]$^-$ | 735,2153 | 735,2165 | 1,6 |
| | | | |
| [M+Na]$^+$ | 723,2383 | 723,2363 | -2,8 |
